# Double Disguise: Camouflaging Photocages for Bioorthogonally Controlled Conditional Activation

**DOI:** 10.1021/jacs.5c15005

**Published:** 2025-10-14

**Authors:** Orsolya Ember, Krisztina Németh, Dóra Kern, Attila Kormos, Péter Kele, Márton Bojtár

**Affiliations:** † MTA—HUN-REN TTK Lendület “Momentum” Chemical Biology Research Group, Institute of Organic Chemistry, 280964 HUN-REN Research Centre for Natural Sciences, Magyar tudósok krt. 2, H-1117 Budapest, Hungary; ‡ Hevesy György PhD School of Chemistry, Eötvös Loránd University, Pázmány Péter sétány 1/A, 1117 Budapest, Hungary

## Abstract

Photocages provide excellent spatiotemporal control over biological processes; however, strategies to restrict their activation to specific conditions remain limited. In this work, we demonstrate that our recently introduced photocages based on the aminofluorone (rhodol) choromophore can be rendered completely inactive through a single substitution on the oxygen auxochrome. These ‘camouflaged’ photocages are entirely colorless, preventing photouncaging in their disabled state. Bioorthogonal removal of the camouflaging group restores the photoactivity of the cages, introducing an additional layer of control for photoassisted delivery systems. We further demonstrate this concept of substitution-dependent, bioorthogonally regulated photoactivation in a cellular context, where both a bioorthogonal reagent and light were required to release a potent topoisomerase inhibitor, SN38.

## Graphical abstract

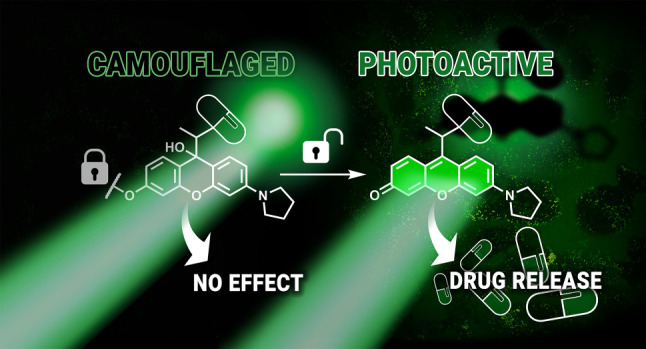

## Introduction

Photocages, also known as photoremovable protecting groups (PPGs) enable irreversible external activation of bioactive compounds with light, allowing spatiotemporal control over a multitude of biological processes.^1–4^ The approach to manipulate biological systems through photocaging has significantly advanced the field of chemical biology^5,6^ and enabled the emergence of light-activatable drugs.^4,7^ Among these, photoactivated chemotherapy (PACT) received considerable attention to increase the therapeutic indices of cytotoxic compounds and localize their action to the disease site.^8–10^ While this concept is certainly appealing in well-defined solid tumors within the reach of light irradiation, further increase of localization precision could significantly increase the potential of such approaches especially in the treatment of diffuse or dispersed tumors in target tissue (‘on-tissue and on-target’ instead of ‘on-tissue and off-target’).^11^ To address these challenges, the concept of conditional photoactivation has recently emerged as a promising strategy for adding an extra layer of control prior to light activation.^12–15^ In these conditionally activatable systems, the photocage is rendered unresponsive to light until a specific chemical/enzymatic transformation restores its photoresponsivity.^16–19^ This dual-controlled approach provides a higher degree of control and selectivity, particularly in therapeutic contexts.^13,15^ Examples of this strategy include photolabile protecting groups quenched by enzyme-cleavable moieties, where the photolability is suppressed until the quencher moiety is enzymatically removed.^20,21^ In these cases, however, the activation wavelength was limited to UV/blue light as only UV-absorbing coumarin chromophores were used. Controlling the photoresponsivity of photocages by bioorthogonal chemistry was recently reported by us^22^ and others,^23^ however, in these cases the inherent wavelength limitation of the bioorthogonal tetrazine unit as a quencher of photoresponsivity severely restricts the utilization of chromophores with absorption maxima above the UV/blue spectral range.^21,24,25^ This calls for versatile design strategies that enable the concept to be extended to a range of photocage scaffolds, particularly those with red-shifted absorption.^26–35^

During our recent efforts to convert xanthenium-based chromophores such as rhodamines to photocages (Figure 1a)^36^ we initially observed the formation of a colorless side-product having an exocyclic double bond. Formation of these species was problematic and the suppression of the ‘exo form’ proved instrumental to the success of our previous work. Nevertheless, we speculated that this exo form may have originated from a ‘leuco form’, a colorless adduct formed by water or other nucleophiles at position 9, a process akin to the intramolecular spirocyclization of xanthene-based fluorescent dyes.^37^

**Figure 1:**
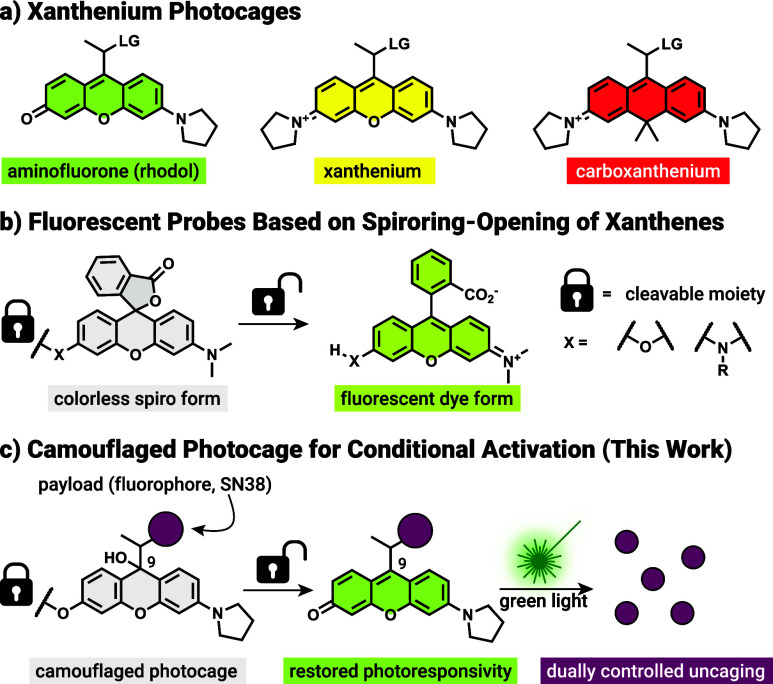
(a) Structures of the xanthenium photocages; LG = leaving group. (b) Spirocyclization-based fluoro- and chromogenic probes. (c) The camouflaged photocage concept.

In the design of chromo- and fluorogenic dyes based on the xanthene core, one of the most popular approaches is to rely on the spirocyclization of a nucleophilic substituent (e.g., COOH, CH_2_OH) on the appending ring.^38–41^ Stimuli-responsiveness is usually introduced via one or several cleavable motifs on the auxochrome(s), thereby modulating the electron density within their conjugated core or by altering the electronic properties of the intramolecular nucleophile responsible for spirocycle closure (Figure 1b).^42,43^

As most photocages, including the structurally analogous xanthenium derivatives lack suitable intramolecular nucleophiles we opted to employ intermolecular nucleophile addition in the design. We hypothesized that similarly to the spirocyclization process, the equilibrium between the leuco and colored species can be shifted toward the leuco form in a substitution-dependent manner, allowing us to effectively mask the absorption of the photocages (‘camouflaging’). Removal of the masking substituent restores the original equilibrium favoring the colored ‘oxo form’, thus reinstating the photoresponsivity of the photocage (Figure 1c).

Here we show that this novel chromogenicity mechanism can be exploited to disable the photoresponsivity of a rhodol-based photocage via a simple substitution. The *O*-substituted camouflaged derivatives are mainly present in their colorless leuco form, rendering the conjugates nonactivatable by light. The rhodol chromophore was further modified with a cleavable *trans*-cyclooctene (TCO) substituent, enabling bioorthogonally controlled conditioned activation via an inverse electron-demand Diels–Alder (IEDDA) reaction with tetrazines.^44^ Unlike earlier strategies that relied on direct quenching of the chromophore by tetrazines,^22–24^ our approach redefines the role of the tetrazine enabling conditional activation to be extended into the red-shifted range. This novel strategy offers dual control over payload liberation: first through selective chemical activation by a bioorthogonal trigger, and then through subsequent release via visible (green) light irradiation. The applicability of this sequential AND-type photouncaging scheme was demonstrated by a conditionally activatable drug release system in live cells.

## Results and Discussion

We first examined the effects of pH and substitution on the equilibrium between various states of photocage **1** based on the rhodol scaffold. To these ends, we have synthesized a series of model compounds featuring a simple carbamate-linked dimethylamine payload with (**2**–**5**) or without (**1-NMe**_**2**_) various substitutions as ‘camouflaging groups’ (Figure 2). Ether or carbamate-type masking groups were introduced to the reduced form of the key xanthene intermediates (Schemes S1 and S2). Attempts to functionalize the oxygen atom of the visible light absorbing ‘oxo form’ failed repeatedly highlighting the synthetic significance of the redox states of the cores. Acetylation yielded only degradation products while silyl-protected derivatives decomposed to the parent photocage. UV/vis absorption measurements conducted across a range of pH values revealed that the rhodol photocage with the model payload (**1-NMe**_**2**_, Figure 2a) exists in a pH-dependent equilibrium among three distinct states: the highly photolabile oxo form, predominant at physiological pH; the cationic ‘iminium form’, favored under acidic conditions; and the colorless, 9-hydroxy adduct leuco form, which was only observable under highly basic conditions (e.g., in 0.5 M NaOH; see Figure S2 in the SI for the corresponding spectra). According to the pH-dependent titration, p*K*_oxo_, defined as the pH value at which the iminium and oxo forms are in equal concentrations based on absorbance data, was determined to be 4.93, indicating 99.7% predominance of the oxo form at pH 7.4.

**Figure 2:**
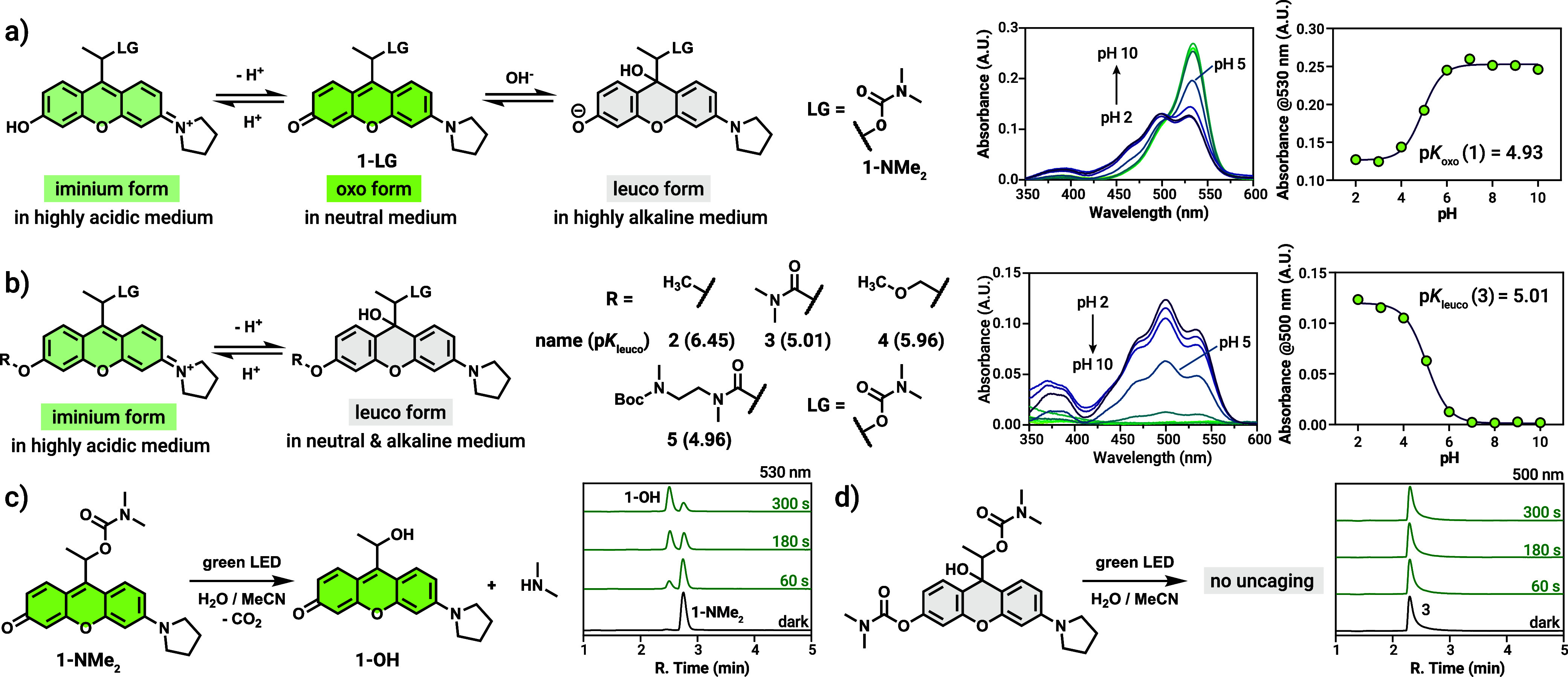
(a) Structure and various forms of **1-NMe**_**2**_ with the respective pH-dependent absorption spectra and absorbance values at 530 nm. (b) Structure and various forms of compounds **2**-**5** and their respective p*K*_leuco_ values together with the pH-dependent absorption spectra and absorbance values at 500 nm for **3**. (c) Uncaging scheme and partial chromatograms of **1-NMe**_**2**_ upon green light irradiation. (d) Uncaging scheme and partial chromatograms of **3** upon green light irradiation. For the full 1D and 2D uncaging data see Section 4.3 in the SI.

Introducing a substituent at the oxygen auxochrome had a dramatic effect on the pH-dependent distribution of the three forms. Substitution at the phenolic oxygen effectively blocks the formation of the oxo tautomer, thereby restricting the equilibrium to the leuco and iminium forms. (Figures 2b and S4–S8 in the SI). Comparison of the respective p*K*_leuco_ values (defined as the pH value at which the iminium and leuco forms are in equal concentrations) suggests that *O*-substituted derivatives are locked mainly in their leuco forms at physiological pH. Notably, *O*-carbamate derivatives displayed the lowest p*K*_leuco_ values (e.g., 5.01 for compound **3**, corresponding to 99.6% leuco at pH 7.4), indicating an almost exclusive presence of the leuco form at physiological pH conditions.

To link the absorption profiles of the parent and substituted photocages with photoresponsivity under physiological conditions, compounds **1**–**5** bearing the same dimethylamine leaving group were irradiated with green light (549 nm, 72 mW output power, 0 to 5 min). As expected, **1-NMe**_**2**_ released dimethylamine rapidly upon irradiation in water (containing 25% MeCN), confirmed by ^1^H NMR studies (Figure S9 in the SI) along with the expected photosolvolysis side-product, **1-OH** (Figure 2c; see Figure S10 in the SI for the full 1D and 2D uncaging data). Satisfyingly, none of the camouflaged rhodols (**2**-**5**) uncaged their payload using the same irradiation as confirmed by HPLC-MS studies as we found no photosolvolysis byproducts (Figures 2d and S11–S14 in the SI). Uncaging experiments performed at acidic pH (∼0.1% HCOOH, pH 3.0) revealed that neither the iminium form of **1-NMe**_**2**_ nor **3** are photoresponsive, indicating that the camouflaging works even in case of the absorbing species at lower pH values (Figures S15 and S16 in the SI). While the mechanism for the quenched activity in the iminium form is unclear, the low fluorescence of **3** at pH 3.0 (Figure S3 in the SI) indicates efficient nonproductive de-excitation pathways, possibly due to the electron-withdrawing nature of the carbamate masking unit. Interestingly, the ether derivative **2** underwent slow photooxidation based on the HPLC-MS data (see Figure S11 in the SI for the uncaging data and the suggested structure).

Following establishment of the substitution-dependent camouflaging concept, we wished to explore bioorthogonally triggered restoration of the photoresponsivity. Bioorthogonal click-to-release (c2r) systems based on *trans*-cyclooctene (TCO) derivatives have recently entered Phase I and II trials in various cancer indications, therefore represent an ideal platform for multimodal targeting applications.^45–47^ Building on our recent work with bioorthogonally generated quinone methides,^48^ we sought to evaluate whether c2r-based chromogenic and fluorogenic systems containing a phenolic leaving group (as found in rhodol-type dyes) are feasible, and whether the release occurs within a reasonable time frame. To this end, we first synthesized a fluorescent probe based on a rhodol fluorophore, **Rho**. The bioorthogonally activatable probe, **rTCO-Rho** features an *N*,*N*′-dimethylethylenediamine self-immolative linker that connects the releasing TCO (rTCO) moiety (via carbamate) to the key oxygen of the dye unit, which results in quenched fluorescence (Scheme 1, see Scheme S3 in the SI for the synthesis). Reaction of **rTCO-Rho** with recently reported bis­(3-hydroxypyridin-2-yl) tetrazine (**Tz**) as a bioorthogonal trigger^49^ gave rise to remarkably increased fluorescence intensity, confirming the release of the dye within a reasonable time frame (∼40-fold increase within 1 h, see Scheme 1a for the reaction cascade and Figures S21 and S22 in the SI for the UV/vis and fluorescence data). Next, we applied this design strategy to the rhodol photocage (**1**) to access conditionally activatable **rTCO-1** derivatives. To allow real-time tracking of the bioorthogonal activation (via UV/vis) and the subsequent photolysis steps (via fluorescence), we loaded a coumarin derivative (**Cou**) as fluorogenic payload. The fluorescence of the payload is quenched in compound **1-Cou** due to Förster’s resonance energy transfer (FRET), enabling direct monitoring of the uncaging via fluorescence. To obtain **rTCO-1-Cou**, first the reduced form of the rhodol precursor was equipped with the coumarin payload through a carbamate bond. Bioorthogonal click-to-release unit (**rTCO** was introduced) via the same self-immolative linker in the final steps of the synthesis, prior to the oxidation of the rhodol core to yield the bioorthogonally activatable construct **rTCO-1-Cou** (see Scheme S4 in the SI for the synthesis). Notable to mention that we have successfully devised a synthetic protocol, where the product is accessed purely in its leuco form simply by applying a quick liquid–liquid phase extraction step.

**Scheme 1:**
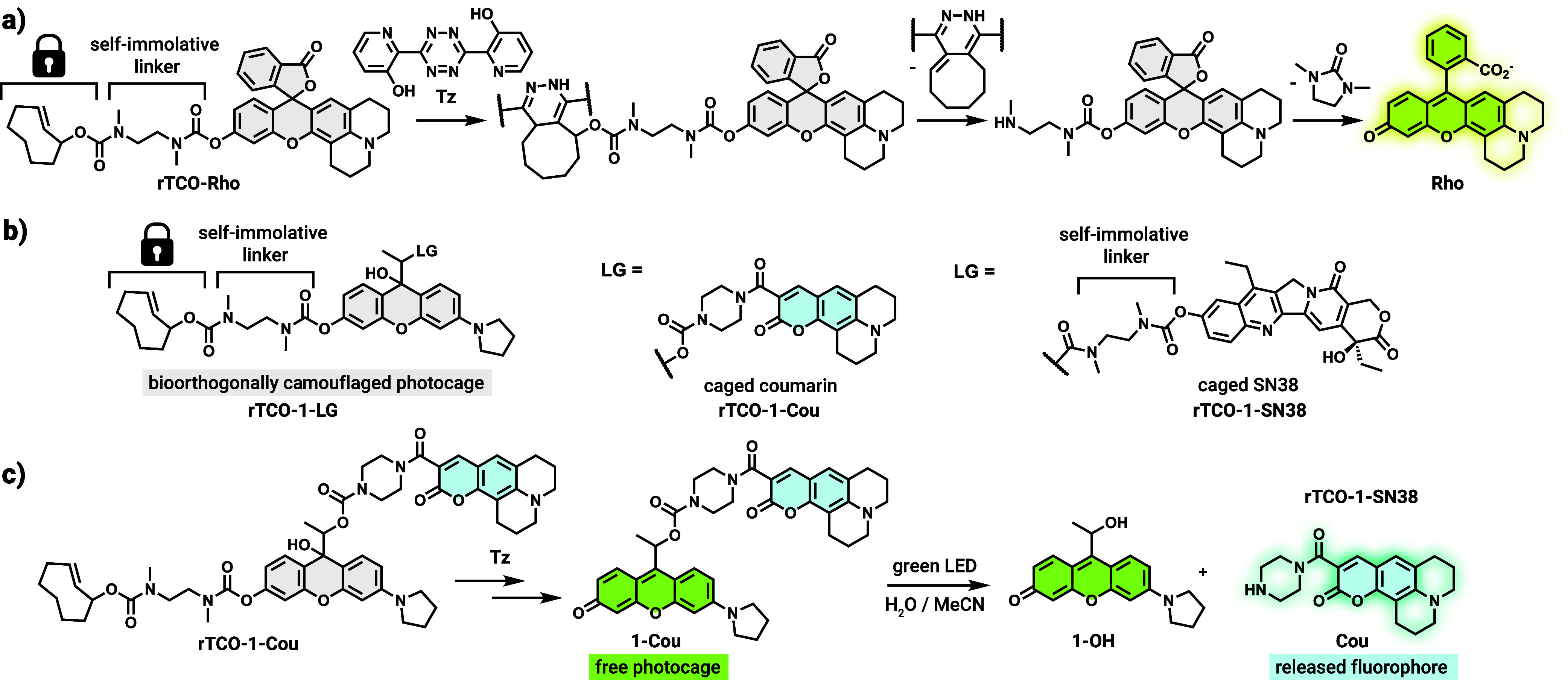
(a) Structure and c2r Reaction Sequence of **rTCO-Rho** with **Tz**; (b) Structure of the Bioorthogonally Camouflaged Rhodols; (c) c2r Reaction and Photo-Uncaging Sequence of **rTCO-1-Cou**

Next, we assessed the release kinetics of **rTCO-1-Cou** using UV/vis and HPLC-MS measurements (Figure 3). Upon reaction with the bioorthogonal counterpart tetrazine (**Tz**, 5 equiv), the construct underwent a click-to-release cascade, leading to the formation of the **1-Cou** (Scheme 1c) as indicated by the appearance of a distinct absorption peak at 530 nm characteristics of the oxo tautomer (Figure 3a). Upon full restoration of the oxo form, the sample was diluted and irradiated with green light (549 nm, 72 mW output power) for 6 min. Photolysis was followed by the fluorescence emission of the liberated coumarin payload **Cou** (λ_ex_: 425 nm, Figure 3b). Since absorption-based monitoring of the bioorthogonal activation is only suitable to detect the end point of the multistep process, i.e., the formation of the green-light absorbing oxo form of **1-Cou**, we complemented the monitoring with HPLC-MS analysis under identical reaction conditions to gain deeper insight into the stepwise activation cascade (Figure 3c; for complete 2D contour chromatograms with *m*/*z* values, see Figure S25 in the SI). As expected, multiple intermediates and byproducts could be identified by their respective UV/vis absorption profiles and masses through the reaction sequence. Remarkably, the click reaction proceeded almost instantaneously, and subsequent release and self-immolation reactions reached near completion within just 3 h. It is important to note that the acidic conditions of the HPLC-MS eluent system (0.1% HCOOH in water/MeCN) promote nearly instantaneous conversion of the leuco form to the colored iminium form, preventing detection of the unsubstituted leuco intermediate in this setup.

**Figure 3:**
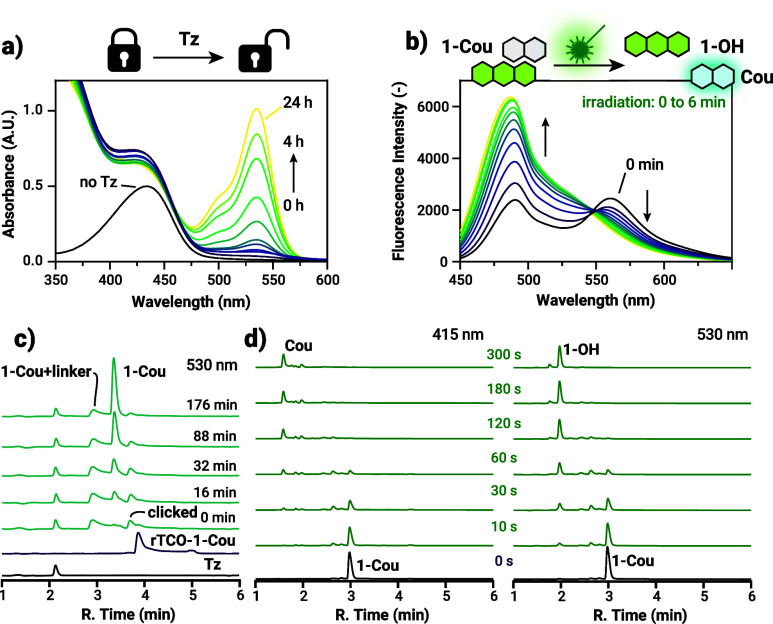
(a) UV/vis measurements of the release kinetics of **rTCO-1-Cou** upon reaction with **Tz**. (b) Fluorescence spectra (λ_ex_ = 425 nm) of the photouncaging of the released **1-Cou** upon irradiation with green light. (c) HPLC chromatograms of the c2r reaction of **rTCO-1-Cou** with **Tz**. (d) HPLC chromatograms of the photouncaging of **1-Cou** upon irradiation with green light.

To gain further insight into the kinetics of the leuco-oxo transformation, the parent rhodol photocage (**1-NMe**_**2**_) was first dissolved in a 1:1 water/methanol mixture containing 0.5 M NaOH to promote formation of the colorless leuco form, confirmed by ^1^H NMR earlier. This basic solution was then diluted with a HEPES buffer solution, and the pH was adjusted to 7.0 with aqueous HCl. Leuco-oxo conversion was then monitored with UV/vis spectroscopy (Figure S29 in the SI).

At neutral pH, the conversion was found to be nearly complete within 2 h (τ_1/2_ ∼ 31 min). The IEDDA reaction was ruled out as the rate-limiting step, since release times remained independent of **rTCO-1-Cou** concentration, as confirmed by UV/vis-monitored c2r experiments (Figures S31 and S32 in the SI). These suggest that overall kinetics are governed by subsequent first-order processes i.e., the release, self-immolation, and leuco-oxo transformation steps.

We also explored the use of a release tetrazine (rTz) in place of rTCO, a design that could extend this chromogenic approach to TCO-pretargeted bioorthogonal applications.^50^ To this end, a photocaged construct bearing a release tetrazine (**rTz-1-Cou**; see Scheme S4 in the SI) was synthesized and evaluated. Its reaction with a complementary TCO successfully generated the activated PPG–payload, **1-Cou**, although with significantly lower rate and efficiency compared to rTCO-based systems (Figure S33).

With bioorthogonal chemistry-based drug delivery approaches in clinical trials for oncological applications, we speculated that this novel, dual activation mechanism is especially appealing to increase the localization precision of photoactivated chemotherapy. In our recent work we have demonstrated that xanthenium photocages can significantly decrease the activity of a highly potent topoisomerase inhibitor, SN38, which can be reinstated after a brief light irradiation.^36^ To demonstrate the viability of dual activation as means for precisely controlled drug activation system, we designed and synthesized prodrug **rTCO-1-SN38**, an rTCO-camouflaged construct that releases SN38 upon tetrazine-triggered activation and subsequent irradiation with green light. Synthesis of the prodrug was accomplished through the previously described protocol using the reduced form of the photocage (Scheme S5 in the SI). Note that in case of SN38 a self-immolative unit was also necessary for payload installation to avoid physiologically labile carbonate bonds.

The activation reaction cascade was first monitored by the established LC-MS protocol allowing us to track the formation of key intermediates (clicked product, **1-SN38**-linker conjugate, see SI Figure S26 for the structures) as well as **1-SN38** (Figure 4; for complete 2D contour chromatograms with *m*/*z* values, see Figure S26 in the SI). Similarly to the click-to-release reaction yielding **1-Cou**, the IEDDA reaction between **rTCO-1-SN38** and **Tz** was virtually instantaneous, while the release required a few hours to reach completion. To assess the activation kinetics under biologically relevant conditions, prodrug **rTCO-1-SN38** was treated with 5 equiv of **Tz** in McCoy’s 5A medium at 37 °C. In this case, however, the inherent fluorescence of the released photocage **1-SN38** (‘oxo-form’) was used to monitor the release process, as this method also reflects the ‘leuco’ to ‘oxo’ transformation (Figure 4b). The fluorescence intensity reached a plateau after approximately 5–6 h.

**Figure 4:**
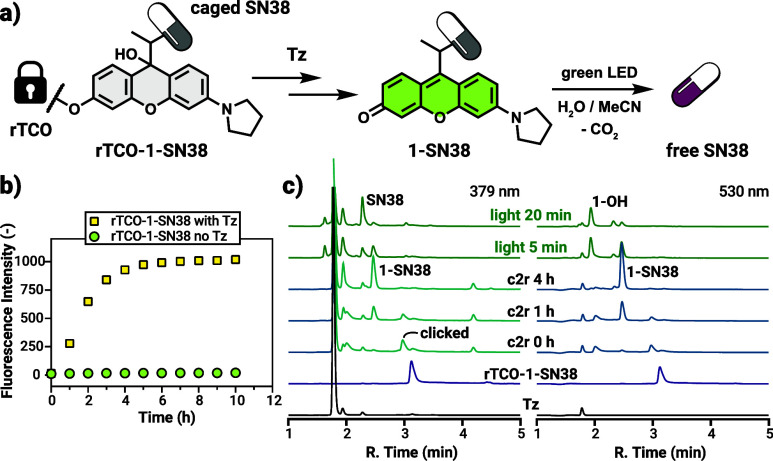
(a) Scheme of the c2r reaction and photouncaging sequence of **rTCO-1-SN38**. (b) Fluorescence measurements of the release kinetics of **rTCO-1-SN38** upon reaction with **Tz** in McCoy’s 5A medium at 37 °C (λ_ex_ = 535 nm, λ_em_ = 575 nm; mean values shown of 3 parallel experiments, SD below the dot marker range). (c) HPLC chromatograms of the c2r reaction of **rTCO-1-SN38** with **Tz** and subsequent irradiation with green light, monitored at the absorption maximum of SN38 and the photocage.

The sequential activation process was further evaluated in a more challenging cellular setting using live SK-OV-3 cells. The cells were incubated with prodrug **rTCO-1-SN38** in the presence or absence of 5 equiv of tetrazine **Tz** for 24 h, followed by confocal microscopy to visualize the fluorescence of the released **1-SN38** prodrug (λ_exc_: 552 nm, λ_det_: 565–615 nm) (Figure 5a). A marked increase in fluorescence was evident from the images both inside the cells (∼14-fold) and in the extracellular medium (∼74-fold, Figure S35 in the SI), clearly indicating the release of unmasked **1-SN38**. We also investigated the subcellular distribution of the photocaged prodrug together with its TCO-camouflaged conjugate **rTCO-1-SN38**. Fluorescence colocalization experiments were performed in SK-OV-3 cells (Figures S36–S38 in the SI). The cells were costained either with LysoTracker Deep Red or MitoTracker Deep Red. The TCO-camouflaged prodrug was imaged both in the absence and presence of 5 equiv of tetrazine (**Tz**). Confocal fluorescence microscopy images revealed strong colocalization of both the parent PPG-SN38 conjugate (**1-SN38**) and its camouflaged derivative (**rTCO-1-SN38**, at higher laser intensities) with LysoTracker, while no significant colocalization with MitoTracker was observed. This suggests that **1-SN38** can cross the cell membrane and localizes primarily in the lysosomes, however, as **rTCO-1-SN38** is not emissive in its leuco form present at neutral pH, the faint lysosomal signal of the iminium form is not conclusive of its true localization.

**Figure 5:**
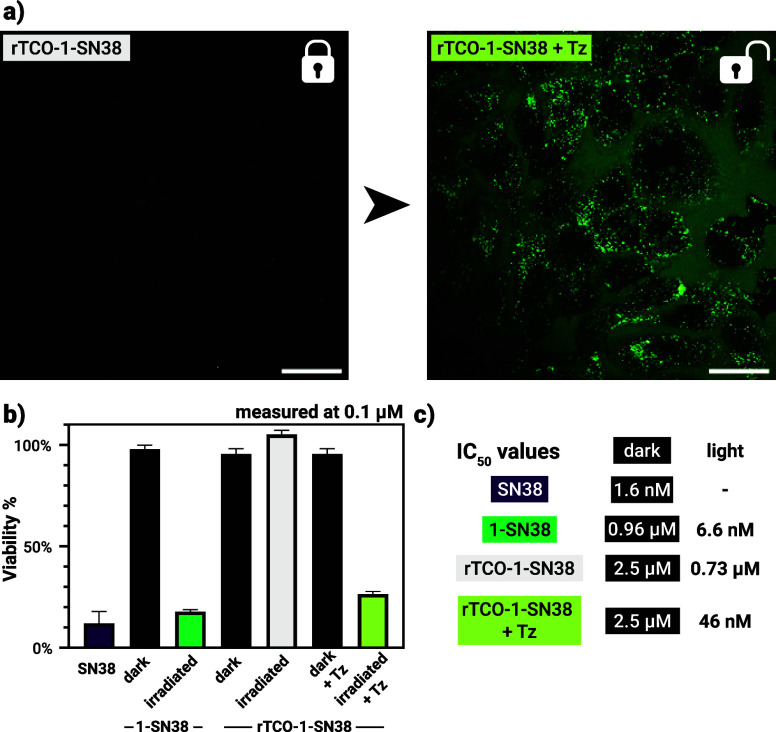
(a) Confocal microscopy images of **rTCO-1-SN38** before and after the addition of **Tz**; scale bar: 50 μm. (b) Viabilities of SK-OV-3 cells after 72 h of treatment with various compounds/conditions. For statistical analysis, see Table S1 in the SI. (c) IC_50_ values of various compounds/conditions.

To further evaluate the feasibility of the dual activation strategy in a biological context, we conducted cell viability assays using SK-OV-3 cells (Figure 5b,c, Section 6 in the SI). The cells were treated either with the photoactivatable conjugate **1-SN38** or the rTCO-camouflaged prodrug **rTCO-1-SN38** under various conditions, that is, in the presence/absence of tetrazine (**Tz**) and with or without 10 min of green light irradiation. Cells were incubated for 72 h, and viability was assessed using an MTT assay. While SN38 effectively halted proliferation (viability ∼ 12%), none of the caged drugs affected cellular viability in the dark at 0.1 μM. Most importantly, the rTCO-camouflaged prodrug was nontoxic at this concentration even if the cells were irradiated, suggesting long-term stability and lack of photoresponsivity in a cellular context. Together with the observed (at least partial) accumulation in acidic lysosomes, this also confirms that neither the ‘leuco’ nor the iminium form of the camouflaged derivative can release its payload with green light. Upon irradiation of either **1-SN38** and **rTCO-1-SN38** in the presence of **Tz**, the viabilities decreased as expected (∼18 and 26%, respectively), indicating efficient liberation of toxic SN38. Importantly, while the low pH of the lysosomes certainly affects the uncaging efficiency (see above), at pH ∼ 5 about 54% of **1-SN38** is still in the photoactive oxo form. We have also determined concentration-dependent viabilities for all cases (Figures S39–S41 in the SI), which yielded dark IC_50_ values of 0.96 and 2.5 μM for **1-SN38** and **rTCO-1-SN38**, respectively. For comparison, the IC_50_ of free SN38 was determined to be 1.6 nM, consistent with published data.^51^ Green light irradiation of **1-SN38** and **rTCO-1-SN38** in the presence of **Tz** restored the biological activity of SN38, as suggested by the respective IC_50_ values of 6.6 nM and 46 nM. While the latter is well above the activity of SN38, it still indicates successful uncaging when both the chemical trigger and light are present. In contrast, when cells treated with **rTCO-1-SN38** and light in the absence of tetrazine, only a minimal decrease in the IC_50_ value was observed (0.73 μM), suggesting practically no uncaging. No toxicity for **Tz** was observed either with or without green light irradiation below micromolar concentrations.

## Conclusions

In summary, we have introduced a novel chromogenic design strategy suitable to modulate the photoresponsivity of recently developed xanthenium photocages. Substitution at the oxygen auxochrome of the aminofluorone (rhodol) photocage results in diminished photoresponsivity due to interconversion into a colorless leuco form. This camouflaging strategy allows the extension of the conditional photoactivation concept both in terms of core scaffolds and cleavable quencher motifs by incorporating bioorthogonally cleavable systems into photocages. Introduction of a release TCO moiety, connected to the chromophore via a carbamate-based self-immolative linker enables bioorthogonal control over photoresponsivity. Treatment of such constructs with a tetrazine to trigger click-to-release reaction successfully restored the photolability of the photocage by allowing the formation of the photoresponsive species under mild, physiological conditions. We applied this strategy to the design of a dually controlled prodrug, providing a further layer of control to photouncaging. Detailed monitoring of the cascade reactions in vitro and in cellulo revealed efficient restoration of photoresponsivity of the rhodol photocage upon tetrazine triggering. Experimental evidence suggests that both triggers were necessary to release the free drug (SN38), thereby providing dual—chemical and physical—control over the payload release. Although the current activation method requires hours for completion, very recent developments in the field of bioorthogonal bond cleavage chemistries allow for rapid release (within minutes), enabling truly pretargeted systems for precise control.^52^

While this proof-of-concept study applied bioorthogonal click-to-release reactions and a tetrazine reagent as a trigger, the general design strategy is not restricted to bioorthogonal conditions as enzyme-activatable moieties could also be leveraged for photocage activation. In this context, overexpressed cancer-cell specific enzymes or enzymes in the tumor microenvironment can all be utilized for precise, on-tissue and on-target drug delivery purposes in light-accessible malignancies. Altogether, these results establish a versatile platform for conditionally activatable photocages, offering an additional layer of control over light-triggered systems in chemical biology and precision medicine.

## Supplementary Material



## Notes

Experimental details, synthetic procedures, characterization data, spectroscopic characterization, uncaging experiments, details on the imaging experiments, further images, and viability assessment (PDF)
