# Supplementary material for: Double Disguise: Camouflaging Photocages for Bioorthogonally Controlled Conditional Activation
Source: J Am Chem Soc. 2025 Oct 14;147(42):38889–96. doi: 10.1021/jacs.5c15005 (PMC12550854; doi:10.1021/jacs.5c15005)
Supplement: Supplementary file 1 [file ja5c15005_si_001.pdf]

Supporting Information (SI)

Double Disguise: Camouflaging Photocages for Bioorthogonally Controlled Conditional Activation

Orsolya Ember,[a, b] Krisztina Németh,[a] Dóra Kern,[a, b] Attila Kormos,[a] Péter Kele[a,*], Márton Bojtár[a*]

[a] MTA – HUN-REN TTK Lendület "Momentum" Chemical Biology Research Group, Institute of Organic Chemistry, HUN-REN Research Centre for Natural Sciences. Magyar tudósok krt. 2. H-1117, Budapest, Hungary. e-mail: bojtar.marton@ttk.hu, kele.peter@ttk.hu

[b] Hevesy György PhD School of Chemistry, Eötvös Loránd University, Pázmány Péter sétány 1/A, 1117 Budapest

Contents

1. MATERIALS AND METHODS 2

2. SYNTHESIS OF THE COMPOUNDS 3

2.1 Synthetic Overview 3
2.2 Synthesis of the Photocage Precursors 6
2.3 Synthesis of the Model Rhodol Compounds 8
2.4 Synthesis of the rTCO-Rho Fluorogenic Dye 14
2.5 Synthesis of the Coumarin Conjugates 14
2.6 Synthesis of the SN38 Conjugates 20

3. SPECTROSCOPIC PROPERTIES OF THE COMPOUNDS 23

3.1 Molar Absorption Coefficients 23
3.2 Absorption and Emission Spectra of the Various Forms 23
3.3 pH-Dependent Absorption Spectra 24

4. UNCAGING STUDIES 26

4.1 Experimental Details 26
4.2 NMR Studies 27
4.3 HPLC Chromatograms of the Irradiation Experiments 28
4.4 Click-to-Release Experiments of rTCO-Rhod 38
4.5 Dark Stability of the rTCO-Compounds 40
4.6 HPLC Chromatograms of the Click-to-Release Experiments 42
4.7 Uncaging Experiments Followed by Optical Spectroscopy Methods 45

5. FLUORESCENCE MICROSCOPY 48

5.1 Cell Sample Preparation for Fluorescence Imaging Experiments 48
5.2 Live Cell Fluorescence Imaging 48
5.3 Colocalization Studies 49

6. VIABILITY STUDIES 52

6.1 Experimental Details 52
6.2 Viability Curves and IC50 Determination 52

7. NMR SPECTRA AND HPLC-MS DATA 54

8. REFERENCES 109

1. Materials and Methods

All starting materials were purchased from commercial suppliers (Sigma Aldrich, Fluorochem, Merck, Alfa Aesar, Acros, Doug Discovery, Molar Chemicals) and used without further purification. Reaction monitoring was performed by analytical thin-layer chromatography (TLC) on Merck silica gel 60 F254 precoated aluminum plates. Flash column chromatography was performed using a Teledyne ISCO CombiFlash automated system with RediSep Gold columns.

NMR spectra were recorded on a Varian Inova 500 MHz or a Varian Inova 300 MHz spectrometer. Chemical shifts (δ) are reported in parts per million (ppm) relative to residual solvent signals or TMS. Coupling constants are given in hertz (Hz).

Analytical reversed-phase HPLC-UV/Vis-MS analyses were performed on a Shimadzu LC-MS-2020 system equipped with a Gemini C18 column (100 × 2.0 mm, 5 μm, 110 Å). Detection was achieved using a diode array detector (190-800 nm) and an electrospray ionization mass spectrometer (ESI-MS). Two LC-MS gradient elution methods were employed, both using the same mobile phase system and conditions. Eluent A consisted of 94.9% H_2O, 5.0% MeCN, and 0.1% HCOOH; eluent B consisted of 94.9% MeCN, 5.0% H_2O, and 0.1% HCOOH. The flow rate was set to 0.8 mL/min, and the column temperature was maintained at 40 °C.

The longer gradient method (8.5 min) used the following profile: 0.00 min – 0% B, 6.00 min – 100% B, 7.00 min – 100% B, 7.50 min – 0% B, 8.50 min – 0% B.

The shorter gradient method (5.0 min) used the following profile: 0.00 min – 0% B, 2.50 min – 100% B, 3.13 min – 100% B, 3.75 min – 0% B, 5.00 min – 0% B.

Semi-preparative HPLC was conducted using a Wufeng Chrom LC100 system with a Gemini C18 column (150 × 21 mm, 5 μm, 110 Å).

Fluorescence spectra were recorded on a Jasco FP-8300 spectrofluorometer. UV/Vis spectra were collected using a Jasco V-750 spectrophotometer. Quartz cuvettes with 1 cm pathlength were used.

High-resolution mass spectrometric measurements were performed using a Sciex TripleTOF 5600+ hybrid Quadrupole-TOF LC/MS/MS system in positive electrospray mode.

Note about the characterization of the compounds

In the case of complicated/non-resolvable ^{13}C spectra, we provided HPLC-UV-MS data with 2D contour plots and total ion chromatograms. Usually, in the case of substituted rhodol compounds, due to the dynamic nature of the intermolecular addition reaction multiple smaller peaks can be observed in the HPLC chromatograms with the same *m*/*z* values (sometimes also complicated by the separation of the diastereomers). During the course of our experiments, the regular maintenance of the HPLC-MS system was performed responsible for the alteration of the retention times of various compounds in different experiments.

2. Synthesis of the Compounds

2.1 Synthetic Overview

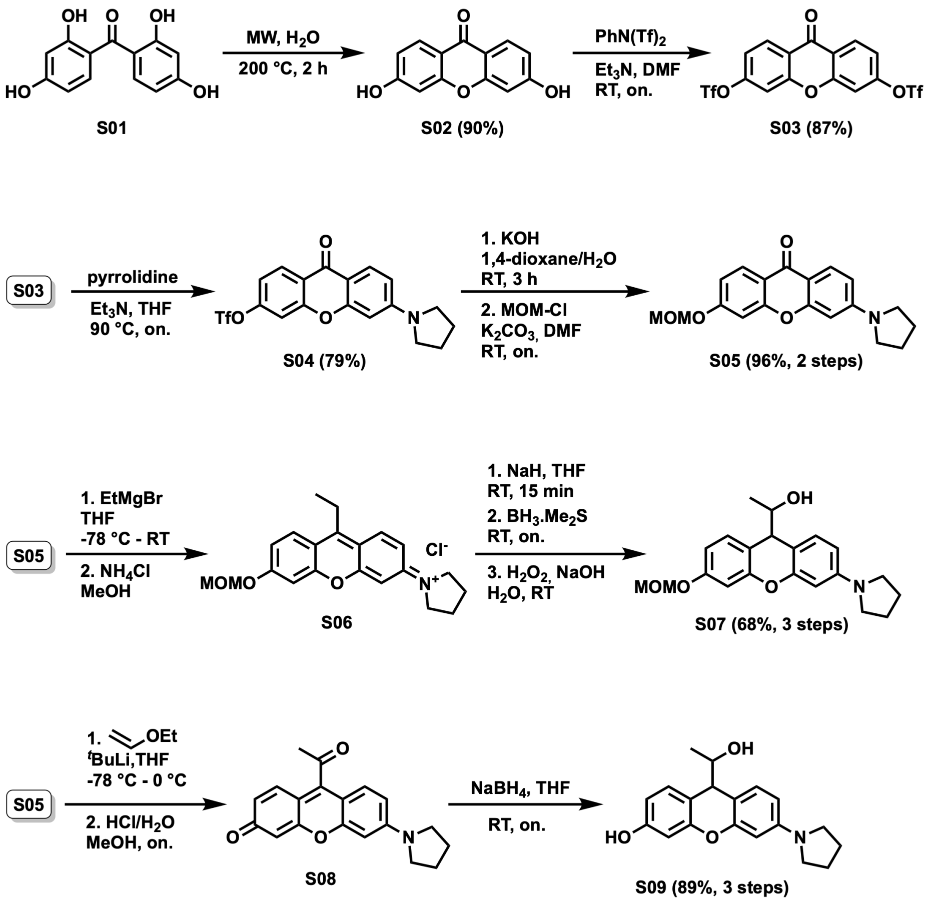

Scheme S1. Synthesis of the photocage precursors.

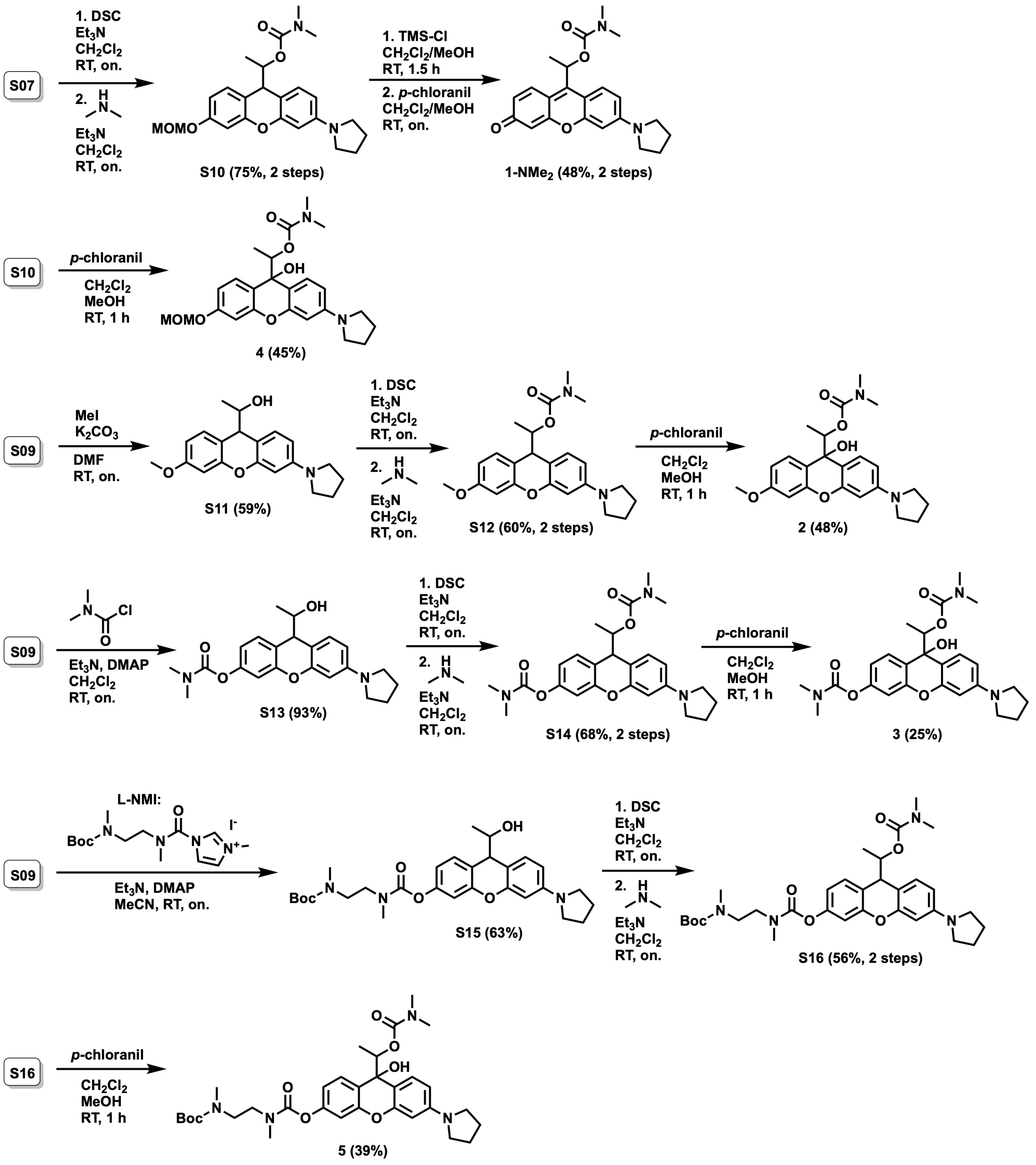

Scheme S2. Synthesis of the model rhodol compounds.

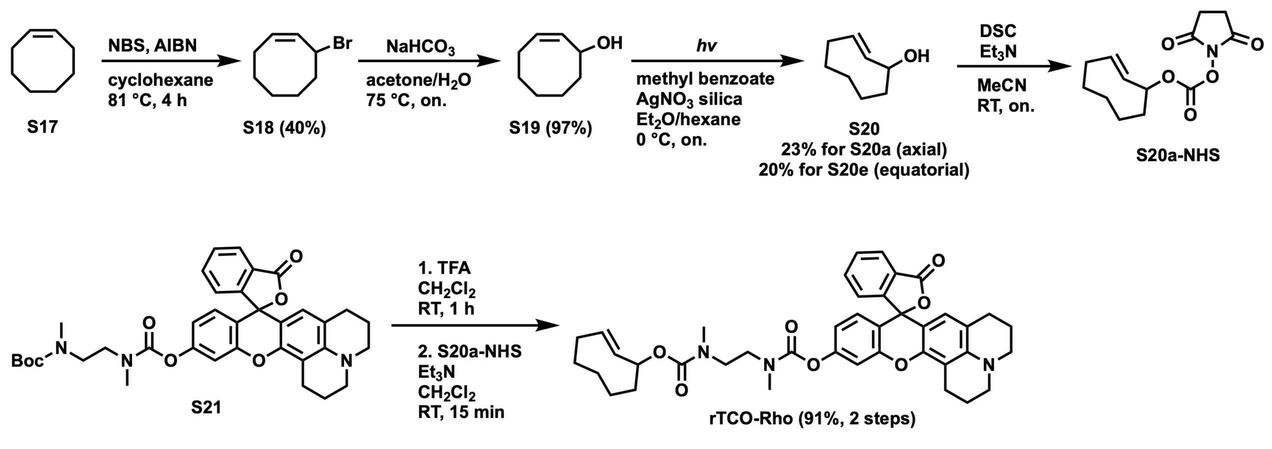

Scheme S3. Synthesis of ***rTCO-Rho****.*

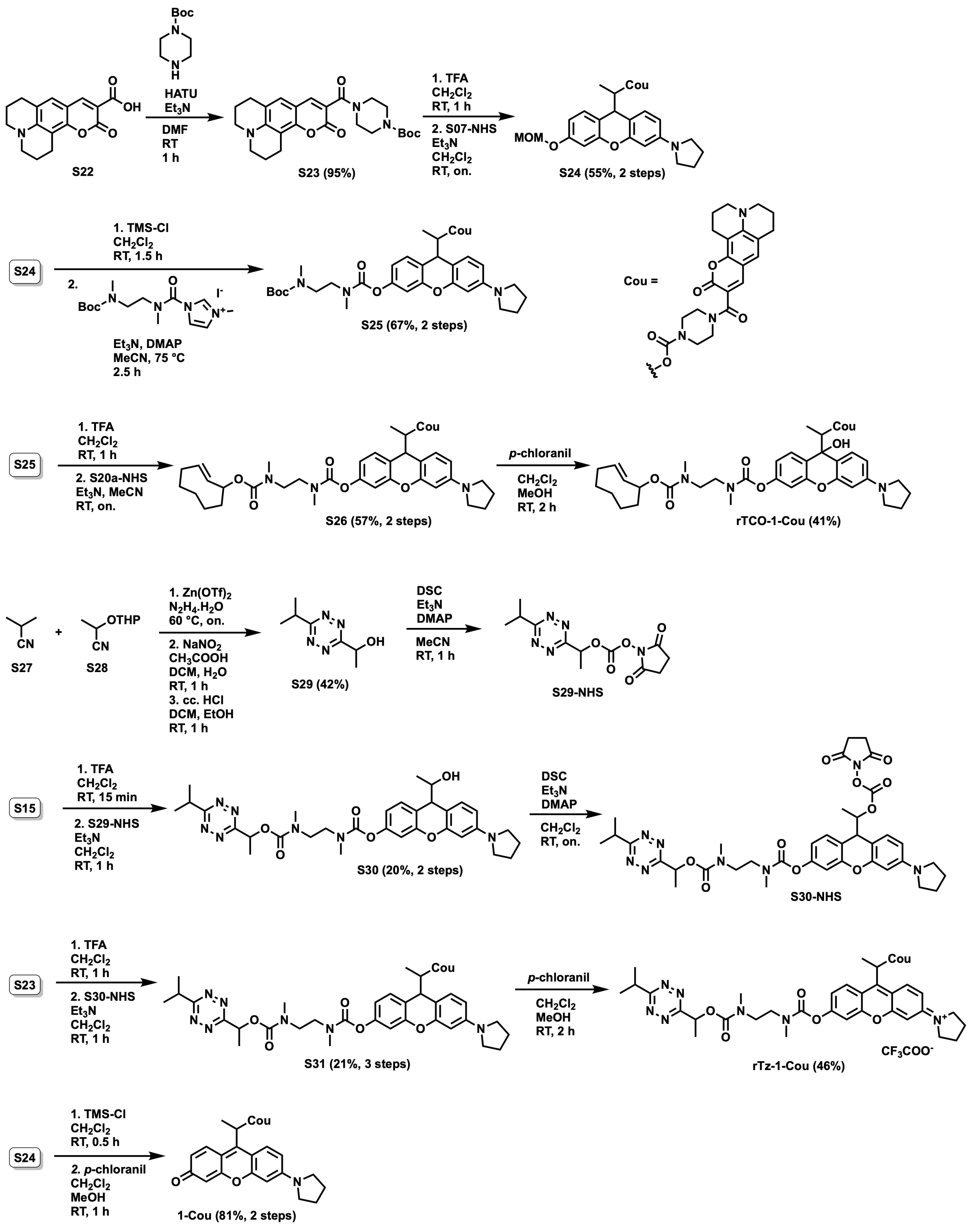

*Scheme S4. Synthesis of the fluorogenic rhodol compounds **rTCO-1-Cou**, **rTz-1-Cou**, and **1-Cou**.*

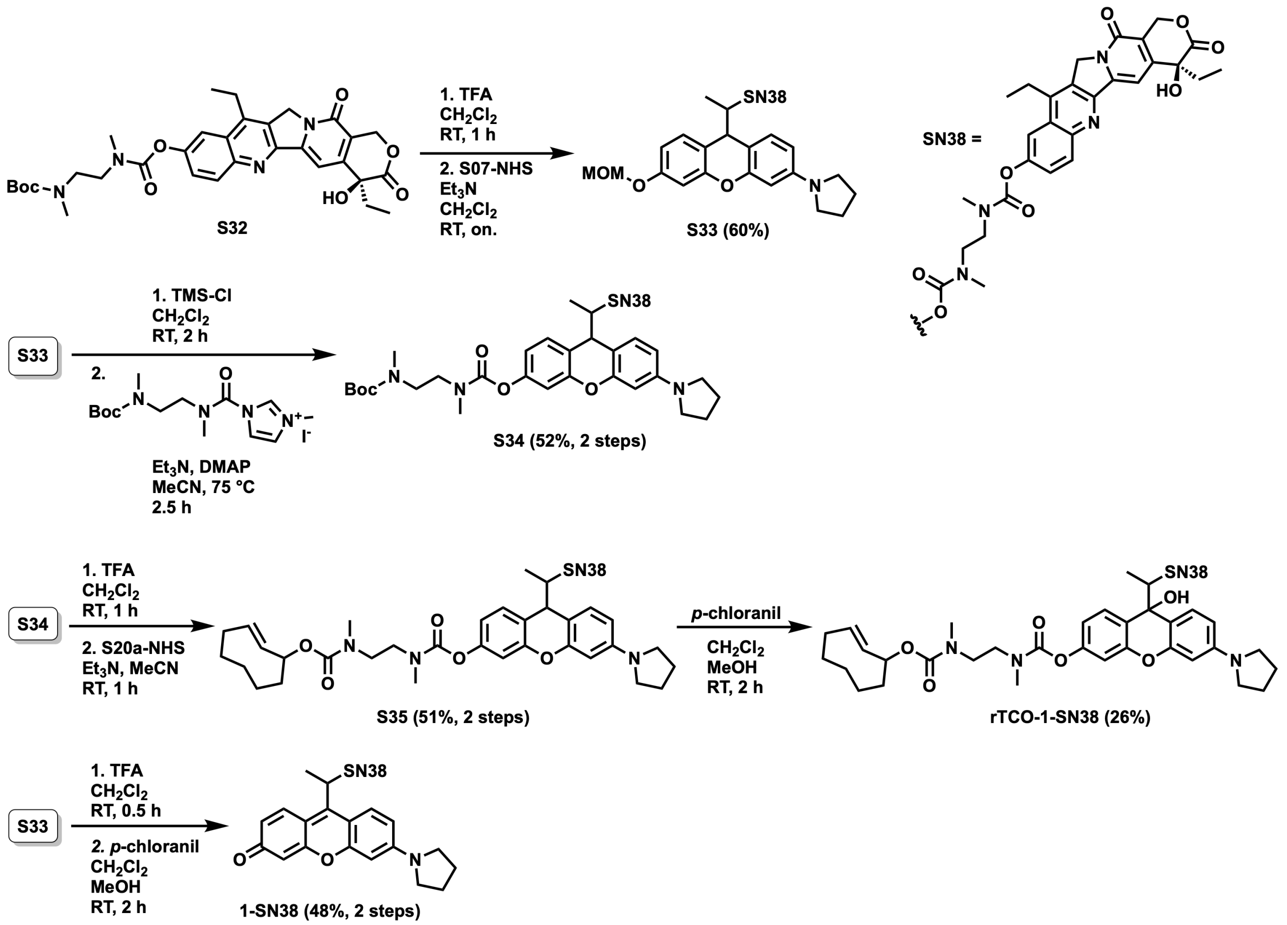

*Scheme S5. Synthesis of the drug-containing rhodol compounds **rTCO-1-SN38** and **1-SN38**.*

2.2 Synthesis of the Photocage Precursors

Compound **S05** [1] and 1-((2-((*tert*-butoxycarbonyl)(methyl)amino)ethyl)(methyl)carbamoyl)-3-methyl-1*H*-imidazol-3-ium iodide (**L-NMI**) [2] were synthesized according to reported procedures.

1-(3-(Methoxymethoxy)-6-(pyrrolidin-1-yl)-9H-xanthen-9-yl)ethan-1-ol (S07)

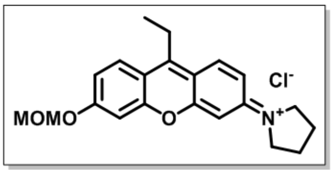

Grignard reaction (step 1): In a previously dried round-bottom flask, 3-(methoxymethoxy)-6-(pyrrolidin-1-yl)-9*H*-xanthen-9-one (**S05**) (565 mg, 1.74 mmol, 1.0 equiv.) was dissolved in anhydrous THF (10 mL) under Ar atmosphere, and the solution was cooled to -78 °C. EtMgBr (1 M in THF, 5.21 mL, 5.21 mmol, 3.0 equiv.) was added dropwise at this temperature. After stirring for 15 minutes, the reaction mixture was allowed to warm to room temperature and stirred for an additional hour. Upon verification of complete conversion using LC-MS, quenching was performed by the slow addition of saturated aqueous NH_4Cl (30 mL), followed by extraction with CH_2Cl_2 (3 × 50 mL). The combined organic layers were washed with brine, dried over anhydrous $MgSO_4$, and concentrated under reduced pressure. The resulting dark pink oil (**S06**) was used in subsequent steps without further purification.

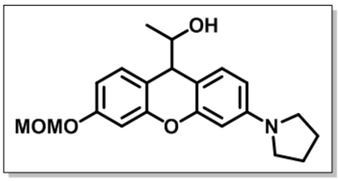

Hydroboration and oxidation step (steps 2 and 3): The crude ethyl-pyronine intermediate (**S06**) (calculated: 586 mg, 1.74 mmol, 1.0 equiv.) was dissolved in anhydrous THF (10 mL), and NaH (60% in mineral oil, 350 mg, 8.75 mmol, 5.0 equiv.) was added under Ar atmosphere. After stirring for 15 minutes, the solution was added through a syringe filter into a previously dried round-bottom flask, and the NaH was washed with THF (5 mL). The solution was cooled to 0 °C, and borane-dimethyl sulfide complex (1.65 mL, 17.4 mmol, 10 equiv.) was added dropwise. The reaction mixture was allowed to warm to room temperature and stirred overnight. After LC-MS analysis confirmed complete conversion, the reaction was quenched by the addition of methanol (15 mL) and 3 M aqueous NaOH (10 mL), followed by the slow addition of 30% hydrogen peroxide (15 mL). The mixture was extracted with CH_2Cl_2 (3 × 60 mL). The combined organic phases were washed with aqueous sodium thiosulfate (2 × 60 mL), dried over

anhydrous $MgSO_4$, and concentrated under reduced pressure. The crude product was purified by RP flash chromatography (eluent: 0.1% TFA in H_2O/MeCN, gradient from 5% to 100% MeCN) to afford compound **S07** as a pink solid.

Yield: 420 mg (68% for the 3 steps)

For the characterization data, please refer to our previous paper. [1]

9-(1-Hydroxyethyl)-6-(pyrrolidin-1-yl)-9H-xanthen-3-ol (S09)

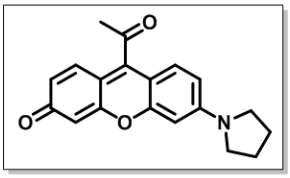

Umpolung acylation (step 1): To a previously dried round-bottom flask, anhydrous THF (7.5 mL) and ethyl vinyl ether (1.82 mL, 19.1 mmol, 7.5 equiv.) were added under Ar atmosphere and cooled to -78 °C. tert-Butyllithium (1.7 M in pentane, 7.50 mL, 12.8 mmol, 5.0 equiv.) was added dropwise, and the solution was stirred at -78 °C for 20 min. The mixture was then warmed to 0 °C in an ice-water bath and stirred for 5 min before being cooled again to -78 °C. A suspension of 3-(methoxymethoxy)-6-(pyrrolidin-1-yl)-9*H*-xanthen-9-one (**S05**) (830 mg, 2.55 mmol, 1.0 equiv.) in dry THF (10 mL) was added dropwise. After stirring for 45 min at -78 °C, the reaction was allowed to warm to room temperature and stirred for an additional 15 min. After the confirmation of complete conversion via LC-MS, the reaction was quenched with MeOH (30 mL), followed by the addition of saturated aqueous NH_4Cl (50 mL). The quenched mixture was extracted with CH_2Cl_2 (3 × 60 mL), and the combined organic phases were washed with brine (2 × 40 mL), dried over anhydrous $MgSO_4$, and concentrated under reduced pressure. The residue was dissolved in MeOH (50 mL), and concentrated HCl (15 mL) was added; then the mixture was stirred overnight at room temperature to complete hydrolysis. After confirming completion by LC-MS, the reaction was diluted with water and concentrated $NaHCO_3$ solution to adjust the pH in the 7-9 range and extracted with CH_2Cl_2 (5 × 50 mL). The combined organic phases were dried over anhydrous $MgSO_4$ and concentrated under reduced pressure. The resulting dark purple solid (**S08**) was used in the next step without further purification.

^{1}H NMR (500 MHz, $CDCl_3$) δ 7.13 – 7.08 (m, 2H, contained in this multiplet: 7.11 (d, *J* = 9.1 Hz, 1H), 7.09 (d, *J* = 9.5 Hz, 1H)), 6.63 (dd, *J* = 9.5, 2.0 Hz, 1H), 6.52 (dd, *J* = 8.9, 2.3 Hz, 1H), 6.41 (d, *J* = 2.3 Hz, 1H), 6.38 (d, *J* = 2.0 Hz, 1H), 3.45 – 3.37 (m, 4H), 2.65 (s, 3H), 2.12 – 2.05 (m, 4H).

^{13}C NMR (126 MHz, $CDCl_3$) δ 202.40, 185.08, 158.78, 155.50, 152.30, 148.16, 129.18, 128.29, 127.56, 111.46, 111.20, 106.37, 105.98, 97.75, 48.26, 32.91, 25.47.

HRMS: $[M+H]^+$: calcd for $[C_{19}H_{18}NO_3]^+$: 308.1286, found: 308.1299.

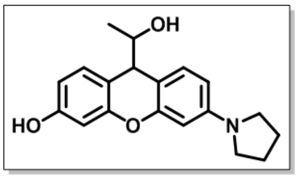

Double reduction (step 2): Crude intermediate **S08** (calculated: 784 mg, 2.55 mmol, 1.0 equiv.) was dissolved in EtOH (70 mL), cooled to 0 °C, and $NaBH_4$ (965 mg, 25.5 mmol, 10 equiv.) was added. The reaction mixture was stirred overnight at room temperature until complete conversion was confirmed by LC-MS. The reaction was quenched by the addition of water (50 mL). The aqueous solution was extracted with CH_2Cl_2 (3 × 50 mL), and the combined organic phases were washed with water (50 mL), then dried over anhydrous $MgSO_4$. The solution was concentrated under reduced pressure to yield a light pink solid (**S09**), which was used in subsequent steps without further purification.

Yield: 710 mg (89% for the 2 steps)

^{1}H NMR (500 MHz, $CDCl_3$) δ 7.09 (d, *J* = 8.3 Hz, 1H), 7.06 (d, *J* = 8.2 Hz, 1H), 6.59 (d, *J* = 2.4 Hz, 1H), 6.54 (dd, *J* = 8.2, 2.5 Hz, 1H), 6.34 (dd, *J* = 8.2, 2.5 Hz, 1H), 6.31 (d, *J* = 2.4 Hz, 1H), 3.87 – 3.77 (m, 2H), 3.35 – 3.23 (m, 4H), 2.06 – 1.95 (m, 4H), 0.98 (d, *J* = 5.7 Hz, 3H).

^{13}C NMR (126 MHz, $CDCl_3$) δ 155.75, 153.82, 153.78, 148.41, 130.06, 129.91, 115.40, 110.43, 108.46, 107.63, 103.73, 99.18, 73.69, 47.89, 45.61, 25.60, 18.76.

HRMS: $[M+H]^+$: calcd for $[C_{19}H_{22}NO_3]^+$: 312.1599, found: 312.1613.

2.3 Synthesis of the Model Rhodol Compounds

1-(3-(Methoxymethoxy)-6-(pyrrolidin-1-yl)-9H-xanthen-9-yl)ethyl dimethylcarbamate (S10)

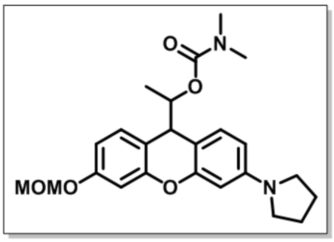

Activation with DSC (step 1): Compound **S07** (60 mg, 0.17 mmol, 1.0 equiv.) was dissolved in CH_2Cl_2 (stabilized with amylene, 1.5 mL), then *N,N'*-disuccinimidyl carbonate (0.17 g, 0.68 mmol, 4.0 equiv.) and Et_3N (0.14 mL, 1.0 mmol, 6.0 equiv.) were added to the solution. The reaction mixture was stirred overnight at room temperature. After confirming the complete conversion with LC-MS, the reaction mixture was diluted with CH_2Cl_2 (stabilized with amylene, 50 mL), and it was extracted with concentrated $NaHCO_3$ solution (5 × 30 mL). The organic layer was dried over anhydrous $MgSO_4$, and the solution was concentrated under reduced pressure to yield mixed carbonate **S07-NHS**, which was used in the subsequent steps without further purification.

Carbamate bond formation (step 2): Activated compound **S07-NHS** (calculated: 84 mg, 0.17 mmol, 1.0 equiv.) was dissolved in CH_2Cl_2 (stabilized with amylene, 1.5 mL), then dimethylamine solution (2 M in THF, 0.17 mL, 0.34 mmol, 2.0 equiv.) and Et_3N (0.12 mL, 0.85 mmol, 5.0 equiv.) were added. The solution was stirred overnight at room temperature. After LC-MS analysis confirmed the completion of the reaction, the solution was concentrated under reduced pressure, and the crude product was purified by HPLC (eluent: 0.1% TFA in H_2O/MeCN, gradient from 5% to 100% MeCN).

Yield: 54 mg (75% for the 2 steps)

NMR note: signal duplication due to diastereomers and restricted rotation

^{1}H NMR (500 MHz, $CDCl_3$) δ 7.18 – 6.91 (m, 2H, contained in this multiplet: 7.16 (d, *J* = 8.3 Hz, 1H, 7.08 (d, *J* = 8.3 Hz, 1H), 7.03 (d, *J* = 8.4 Hz, 1H), 6.95 (d, *J* = 8.3 Hz, 1H)), 6.74 – 6.71 (m, 1H, contained in this multiplet: 6.73 (d, *J* = 2.3 Hz, 1H), 6.71 (d, *J* = 2.3 Hz, 1H)), 6.70 – 6.65 (m, 1H, contained in this multiplet: 6.69 (dd, *J* = 8.3, 2.3 Hz, 1H), 6.68 (dd, *J* = 8.3, 2.3 Hz, 1H)), 6.31 – 6.25 (m, 1H, contained in this multiplet: 6.28 (dd, *J* = 8.4, 2.3 Hz, 1H), 6.27 (dd, *J* = 8.4, 2.3 Hz, 1H)), 6.23 – 6.19 (m, 1H, contained in this multiplet: 6.22 (d, *J* = 2.5 Hz, 1H), 6.20 (d, *J* = 2.4 Hz, 1H)), 5.14 – 5.08 (m, 2H, contained in this multiplet: 5.12 (s, 2H), 5.11 (s, 2H)), 4.88 – 4.78 (m, 1H), 4.09 (d, J = 3.6 Hz, 1H), 3.47 – 3.39 (m, 3H, contained in this multiplet: 3.44 (s, 3H), 3.43 (s, 3H)), 3.26 – 3.17 (m, 4H), 2.91 – 2.82 (m, 6H, contained in this multiplet: 2.861 (s, 6H), 2.856 (s, 6H)), 2.00 – 1.89 (m, 4H), 0.88 – 0.81 (m, 3H, contained in this multiplet: 0.85 (d, *J* = 6.5 Hz, 3H), 0.84 (d, *J* = 6.4 Hz, 3H)).

^{13}C NMR (126 MHz, $CDCl_3$) δ 157.38, 157.35, 156.29, 156.26, 153.82, 153.59, 153.32, 153.07, 145.85, 145.48, 130.94, 130.48, 130.45, 130.12, 115.21, 114.30, 113.77, 113.68, 111.82, 111.52, 110.78, 110.43, 104.25, 104.05, 102.97, 102.64, 94.73, 94.69, 76.72, 76.70, 56.14, 51.89, 51.30, 42.42, 42.40, 36.49, 35.95, 25.10, 25.05, 15.13, 15.08.

HRMS: $[M+H]^+$: calcd for $[C_{24}H_{31}N_2O_5]^+$: 427.2227, found: 427.2227.

1-(3-Oxo-6-(pyrrolidin-1-yl)-3H-xanthen-9-yl)ethyl dimethylcarbamate (1-NMe$_2$)

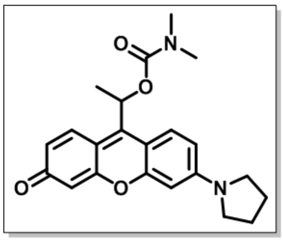

Deprotection (step 1): Compound **S10** (28 mg, 66 µmol, 1.0 equiv.) was dissolved in CH_2Cl_2/MeOH 1:1 (1 mL), then TMS-Cl (100 µL, 0.79 mmol, 12 equiv.) was added, and the reaction mixture was stirred at room temperature for 1.5 hours. The volatiles were removed under reduced pressure, and the crude product was used in the subsequent step without further purification.

Oxidation (step 2): The crude intermediate (calculated: 25 mg, 66 µmol, 1.0 equiv.) was dissolved in CH_2Cl_2/MeOH 1:1 (1 mL), then *p*-chloranil (32 mg, 0.13 mmol, 2.0 equiv.) was added. The reaction mixture was stirred overnight at room temperature. After confirming the completion of the reaction with LC-MS, the solvents were evaporated under reduced pressure, and the product was purified by flash chromatography on silica (eluent: CH_2Cl_2/MeOH 0% to 15%).

Yield: 12 mg (48% for the 2 steps)

Oxo form: ^{1}H NMR (500 MHz, CD_3CN) δ 8.03 (d, *J* = 9.3 Hz, 1H), 7.96 (d, *J* = 9.8 Hz, 1H), 6.67 (dd, *J* = 9.3, 2.4 Hz, 1H), 6.49 – 6.44 (m, 2H, contained in this multiplet: 6.47 (q, J = 7.0 Hz, 1H), 6.46 (dd, *J* = 9.9, 2.0 Hz, 1H)), 6.40 (d, *J* = 2.4 Hz, 1H), 6.10 (d, *J* = 2.0 Hz, 1H), 3.43 – 3.38 (m, *J* = 6.5 Hz, 4H), 2.97 (s, 3H), 2.78 (s, 3H), 2.05 – 2.00 (m, 4H), 1.74 (d, *J* = 7.0 Hz, 3H).
^{13}C NMR (75 MHz, CD_3CN) δ 184.50, 159.93, 156.50, 156.09, 152.91, 151.21, 129.56, 129.15, 128.22, 113.66, 111.95, 108.59, 105.21, 97.74, 69.13, 48.82, 36.66, 36.36, 25.97, 21.51.

Leuco form: ^{1}H NMR (500 MHz, CD_3OD-D_2O 3:1, with 0.5 M NaOD) δ 7.41 – 7.10 (m, 2H, contained in this multiplet: 7.39 (d, *J* = 8.6 Hz, 1H), 7.29 (d, *J* = 8.6 Hz, 1H), 7.23 (d, *J* = 8.6 Hz, 1H), 7.13 (d, *J* = 8.6 Hz, 1H)), 6.54 – 6.44 (m, 2H, contained in this multiplet: 6.52 (dd, *J* = 8.7, 2.4 Hz, 1H), 6.50 (dd, *J* = 8.8, 2.3 Hz, 1H), 6.49 (dd, *J* = 8.8, 1.9 Hz, 1H), 6.47 (dd, *J* = 8.7, 2.2 Hz, 1H)), 6.37 – 6.34 (m, 1H, contained in this multiplet: 6.360 (d, *J* = 2.2 Hz, 1H), 6.356 (d, *J* = 2.2 Hz, 1H)), 6.27 – 6.24 (m, 1H, contained in this multiplet: 6.260 (d, *J* = 2.1 Hz, 1H), 6.256 (d, *J* = 2.2 Hz, 1H)), 5.04 – 4.97 (m, 1H, contained in this multiplet: 5.01 (q, J = 6.3 Hz, 1H), 5.00 (q, J = 6.4 Hz, 1H)), 3.33 – 3.27 (m, 4H), 2.91 (s, 3H), 2.87 (s, 3H), 2.09 – 2.00 (m, 4H), 0.86 – 0.80 (m, 3H, contained in this multiplet: 0.85 (d, *J* = 6.2 Hz, 3H), 0.84 (d, *J* = 6.2 Hz, 3H)).

HRMS: $[M+H]^+$: calcd for $[C_{22}H_{25}N_2O_4]^+$: 381.1808, found: 381.1810.

1-(9-Hydroxy-3-(methoxymethoxy)-6-(pyrrolidin-1-yl)-9H-xanthen-9-yl)ethyl dimethylcarbamate (4)

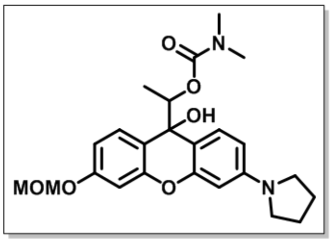

Compound **S10** (32 mg, 75 µmol, 1.0 equiv.) was dissolved in CH_2Cl_2/MeOH 1:1 (1 mL), then *p*-chloranil (28 mg, 0.11 mmol, 1.5 equiv.) was added. The reaction mixture was stirred for 1 hour at room temperature. Once complete conversion was verified by LC-MS, the reaction mixture was diluted with CH_2Cl_2 and extracted with concentrated $NaHCO_3$ solution. The organic layer was dried over anhydrous $MgSO_4$, and the solvents were evaporated under reduced pressure. The crude product was purified by flash chromatography on silica (eluent: CH_2Cl_2/MeOH 0% to 15%).

Yield: 15 mg (45%)

^{1}H NMR (300 MHz, CD_3CN) δ 7.63 – 7.23 (m, 2H), 6.89 – 6.81 (m, 1H), 6.80 – 6.72 (m, 1H), 6.50 – 6.43 (m, 1H), 6.27 – 6.19 (m, 1H), 5.23 – 5.18 (m, 2H), 5.00 – 4.80 (m, 1H), 3.47 – 3.43 (m, 3H), 3.32 – 3.24 (m, 4H), 2.91 – 2.87 (m, 1H), 2.81 – 2.72 (m, 6H), 2.05 – 1.98 (m, 4H), 0.90 – 0.80 (m, 3H).

HRMS: $[M+H]^+$: calcd for $[C_{24}H_{31}N_2O_6]^+$: 443.2176, found: 443.2176; $[M-H_2O+H]^+$: calcd for $[C_{24}H_{29}N_2O_5]^+$: 425.2070, found: 425.2074.

1-(3-Methoxy-6-(pyrrolidin-1-yl)-9H-xanthen-9-yl)ethan-1-ol (S11)

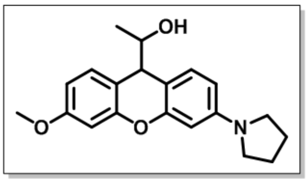

Compound **S09** (50 mg, 0.16 mmol, 1.0 equiv.) and anhydrous K_2CO_3 (55 mg, 0.40 mmol, 2.5 equiv.) were dissolved in dry DMF (1 mL). A solution of MeI (18 µL, 0.29 mmol, 1.8 equiv.) in dry DMF (1 mL) was added dropwise, and the reaction mixture was stirred overnight at room temperature. After establishing complete conversion via LC-MS, the solvent was evaporated under reduced pressure, and the crude product was purified by HPLC (eluent: 0.1% TFA in H_2O/MeCN, gradient from 5% to 100% MeCN).

Yield: 31 mg (59%)

^{1}H NMR (500 MHz, $CDCl_3$) δ 7.22 – 7.09 (m, 2H, contained in this multiplet: 7.20 (d, *J* = 8.3 Hz, 1H), 7.16 (d, *J* = 8.2 Hz, 1H), 7.14 (d, *J* = 8.2 Hz, 1H), 7.11 (d, *J* = 8.2 Hz, 1H)), 6.70 – 6.65 (m, 2H), 6.50 – 6.41 (m, 2H, contained in this multiplet: 6.48 (dd, *J* = 8.4, 2.1 Hz, 1H), 6.46 (dd, *J* = 8.5, 2.4 Hz, 1H), 6.44 (dd, *J* = 8.9, 2.4 Hz, 2H)), 3.89 – 3.81 (m, 5H), 3.41 – 3.30 (m, 4H), 2.11 – 2.02 (m, 4H), 1.03 – 0.94 (m, 3H, contained in this multiplet: 0.99 (d, *J* = 5.6 Hz, 3H), 0.98 (d, *J* = 5.6 Hz, 1H)).

^{13}C NMR (126 MHz, $CDCl_3$) δ 159.71, 159.67, 153.94, 153.81, 153.65, 153.50, 147.58, 147.53, 130.18, 130.17, 129.91, 129.88, 115.18, 114.51, 110.94, 110.13, 109.62, 109.60, 108.61, 108.42, 101.83, 101.62, 100.44, 100.39, 73.60, 73.59, 55.56, 49.08, 48.94, 45.66, 45.57, 25.46, 25.45, 18.85, 18.75.

HRMS: $[M+H]^+$: calcd for $[C_{20}H_{24}NO_3]^+$: 326.1756, found: 326.1768.

1-(3-Methoxy-6-(pyrrolidin-1-yl)-9H-xanthen-9-yl)ethyl dimethylcarbamate (S12)

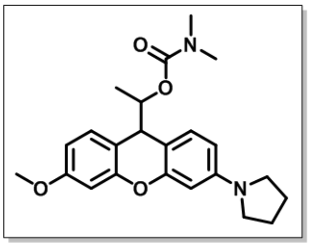

Activation with DSC (step 1): Compound **S11** (30 mg, 92 µmol, 1.0 equiv.) was dissolved in CH_2Cl_2 (stabilized with amylene, 1 mL), then *N,N'*-disuccinimidyl carbonate (94 mg, 0.37 mmol, 4.0 equiv.) and Et_3N (77 µL, 0.55 mmol, 6.0 equiv.) were added to the solution. The reaction mixture was stirred overnight at room temperature. After confirming full conversion via LC-MS, the reaction mixture was diluted with CH_2Cl_2 (stabilized with amylene, 50 mL), and it was extracted with concentrated $NaHCO_3$ solution (5 × 30 mL). The organic layer was dried over anhydrous $MgSO_4$, and the solution was concentrated under reduced pressure to yield mixed carbonate **S11-NHS**, which was used in the subsequent steps without further purification.

Carbamate bond formation (step 2): Activated compound **S11-NHS** (calculated: 43 mg, 92 µmol, 1.0 equiv.) was dissolved in CH_2Cl_2 (stabilized with amylene, 1 mL), then dimethylamine solution (2 M in THF, 92 µL, 0.18 mmol, 2.0 equiv.) and Et_3N (64 µL, 0.46 mmol, 5.0 equiv.) were added. The solution was stirred overnight at room temperature. Upon confirmation of the full conversion by LC-MS analysis, the solution was concentrated under reduced pressure, and the crude product was purified by HPLC (eluent: 0.1% TFA in H_2O/MeCN, gradient from 5% to 100% MeCN).

Yield: 22 mg (60% for the 2 steps)

NMR note: signal multiplication due to diastereomers and restricted rotation:

^{1}H NMR (500 MHz, $CDCl_3$) δ 7.27 – 7.04 (m, 2H), 6.74 – 6.60 (m, 4H), 4.94 – 4.86 (m, 1H), 4.21 – 4.13 (m, 1H), 3.83 – 3.77 (m, 3H), 3.53 – 3.41 (m, 4H), 2.90 (s, 6H), 2.16 – 2.06 (m, 4H), 0.89 (d, J = 6.3 Hz, 3H).

^{13}C NMR (126 MHz, $CDCl_3$) δ 159.83, 156.34, 153.39, 131.05, 130.94, 130.60, 130.45, 130.12, 113.79, 112.90, 110.04, 109.86, 101.60, 101.30, 55.55, 52.38, 51.79, 49.72, 42.40, 25.09, 25.04, 15.11.

HRMS: $[M+H]^+$: calcd for $[C_{23}H_{29}N_2O_4]^+$: 397.2121, found: 397.2121.

1-(9-Hydroxy-3-methoxy-6-(pyrrolidin-1-yl)-9H-xanthen-9-yl)ethyl dimethylcarbamate (2)

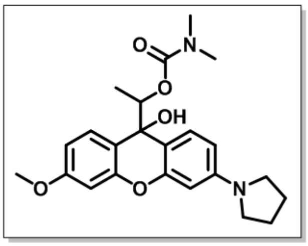

Compound **S12** (22 mg, 55 µmol, 1.0 equiv.) was dissolved in CH_2Cl_2/MeOH 1:1 (1 mL), then *p*-chloranil (20 mg, 83 µmol, 1.5 equiv.) was added. The reaction mixture was stirred for 1 hour at room temperature. After confirming the completion of the reaction with LC-MS, the reaction mixture was diluted with CH_2Cl_2 (50 mL) and extracted with concentrated $NaHCO_3$ solution (2 × 25 mL). After solvent removal under reduced pressure, the crude material was dissolved in MeCN (5 mL), and one drop of TFA was added to convert the compound into its iminium form. This protonated species was purified by HPLC (eluent: 0.1% TFA in H_2O/MeCN, gradient from 5% to 100% MeCN). The product was extracted from the aqueous solution with CH_2Cl_2 (50 mL) and washed with concentrated $NaHCO_3$ solution (2 × 25 mL) to yield the leuco form of the compound. After evaporating the solvents under reduced pressure, a light red solid was obtained.

Yield: 11 mg (48%)

Leuco form: ^{1}H NMR (500 MHz, $CDCl_3$) δ 7.64 – 7.57 (m, 1H, contained in this multiplet: 7.61 (d, *J* = 8.7 Hz, 1H), 7.58 (d, *J* = 8.6 Hz, 1H)), 7.54 – 7.48 (m, 1H, contained in this multiplet: 7.52 (d, *J* = 8.6 Hz, 1H), 7.49 (d, *J* = 8.6 Hz, 1H)), 6.76 – 6.70 (m, 1H, contained in this multiplet: 6.73 (dd, *J* = 8.5, 2.5 Hz, 1H), 6.72 (dd, *J* = 8.5, 2.6 Hz, 1H)), 6.66 – 6.61 (m, 1H, contained in this multiplet: 6.64 (d, *J* = 2.7 Hz, 1H), 6.62 (d, *J* = 2.6 Hz, 1H)), 6.45 – 6.40 (m, 1H, contained in this multiplet: 6.43 (dd, *J* = 8.6, 2.1 Hz, 1H), 6.42 (dd, *J* = 8.5, 2.2 Hz, 1H)), 6.27 – 6.23 (m, 1H, contained in this multiplet: 6.26 (d, *J* = 2.2 Hz, 1H), 6.25 (d, *J* = 2.2 Hz, 1H)), 5.11 – 5.04 (m, 1H, contained in this multiplet: 5.08 (q, *J* = 6.6 Hz, 1H), 5.07 (q, *J* = 6.5 Hz, 1H)), 3.84 – 3.82 (m, 3H, contained in this multiplet: 3.83 (s, 3H), 3.82 (s, 3H)), 3.34 – 3.28 (m, 4H), 2.95 – 2.90 (m, 6H), 2.05 – 1.99 (m, 4H), 0.82 (d, *J* = 6.4 Hz, 3H).

Iminium form: ^{1}H NMR (500 MHz, CD_3CN) δ 8.44 (d, *J* = 9.2 Hz, 1H), 8.40 (d, *J* = 9.8 Hz, 1H), 7.28 – 7.17 (m, 3H), 6.75 (d, *J* = 2.2 Hz, 1H), 6.61 (q, *J* = 7.1 Hz, 1H), 4.02 (s, 3H), 3.78 – 3.73 (m, 2H), 3.68 – 3.63 (m, 2H), 3.01 (s, 3H), 2.75 (s, 3H), 2.14 – 2.09 (m, 4H), 1.83 (d, *J* = 7.0 Hz, 3H).

^{13}C NMR (126 MHz, CD_3CN) δ 168.33, 160.68, 159.81, 158.06, 157.32, 156.00, 131.36, 130.43, 119.76, 117.39, 116.15, 114.06, 101.58, 97.96, 69.74, 57.72, 51.03, 50.83, 25.86, 25.56, 21.57.

HRMS: $[M+H]^+$: calcd for $[C_{23}H_{29}N_2O_5]^+$: 413.2070, found: 413.2072; $[M-H_2O+H]^+$: calcd for $[C_{23}H_{27}N_2O_4]^+$: 395.1965, found: 395.1971.

9-(1-Hydroxyethyl)-6-(pyrrolidin-1-yl)-9H-xanthen-3-yl dimethylcarbamate (S13)

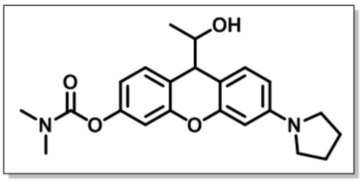

Compound **S09** (50 mg, 0.16 mmol, 1.0 equiv.) and DMAP (1.0 mg, 8.0 µmol, 0.05 equiv.) were dissolved in CH_2Cl_2 (2 mL), then dimethylcarbamoyl chloride (19 mg, 0.18 mmol, 1.1 equiv.) and Et_3N (100 µL, 0.72 mmol, 4.5 equiv.) were added. The reaction mixture was stirred overnight at room temperature. Once the complete conversion was verified by LC-MS, the solvent was removed under reduced pressure. The crude material was purified by HPLC (eluent: 0.1% TFA in H_2O/MeCN, gradient from 5% to 100% MeCN) to yield a brown solid (**S13**).

Yield: 57 mg (93%)

NMR note: signal duplication due to diastereomers.

^1H NMR (500 MHz, $CDCl_3$) δ 7.25 – 7.15 (m, 1H, contained in this multiplet: 7.23 (d, *J* = 8.3 Hz, 1H), 7.18 (d, *J* = 8.3 Hz, 1H)), 7.10 – 7.00 (m, 1H, contained in this multiplet: 7.08 (d, *J* = 8.3 Hz, 1H), 7.03 (d, *J* = 8.3 Hz, 1H)), 6.86 – 6.84 (m, 1H, contained in this multiplet: 6.853 (d, J = 2.3 Hz, 1H), 6.849 (d, J = 2.2 Hz, 1H)), 6.83 – 6.78 (m, 1H, contained in this multiplet: 6.812 (dd, *J* = 8.1, 2.6 Hz, 1H), 6.807 (dd, *J* = 8.1, 2.4 Hz, 1H)), 6.38 – 6.32 (m, 1H, contained in this multiplet: 6.35 (dd, *J* = 7.8, 2.1 Hz, 1H), 6.34 (dd, *J* = 7.9, 2.2 Hz, 1H)), 6.32 – 6.27 (m, 1H, contained in this multiplet: 6.30 (d, *J* = 1.8 Hz, 1H), 6.29 (d, *J* = 1.9 Hz, 1H)), 3.86 – 3.72 (m, 2H), 3.31 – 3.23 (m, 4H), 3.08 (s, 3H), 2.99 (s, 3H), 2.21 (s, 1H), 2.03 – 1.96 (m, 4H), 0.99 – 0.92 (m, 3H, contained in this multiplet: 0.97 (d, *J* = 6.2 Hz, 3H), 0.94 (d, *J* = 6.2 Hz, 3H)).

^{13}C NMR (126 MHz, $CDCl_3$) δ 155.09, 155.04, 153.58, 153.57, 153.31, 153.29, 150.99, 150.93, 148.08, 148.08, 129.98, 129.97, 129.78, 129.68, 120.64, 119.64, 116.29, 110.19, 109.96, 109.38, 109.02, 107.94, 107.83, 99.55, 99.41, 73.34, 73.09, 48.17, 48.13, 46.18, 45.85, 36.78, 36.54, 25.48, 18.76, 18.53.

HRMS: $[M+H]^+$: calcd for $[C_{22}H_{27}N_2O_4]^+$: 383.1965, found: 383.1970.

9-(1-((Dimethylcarbamoyl)oxy)ethyl)-6-(pyrrolidin-1-yl)-9H-xanthen-3-yl dimethylcarbamate (S14)

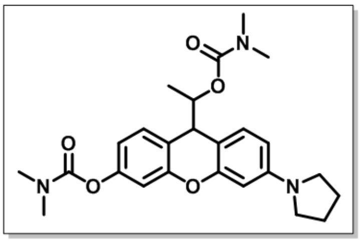

Activation with DSC (step 1): Compound **S13** (57 mg, 0.15 mmol, 1.0 equiv.) was dissolved in CH_2Cl_2 (stabilized with amylene, 1 mL), then *N,N'*-disuccinimidyl carbonate (0.15 g, 0.60 mmol, 4.0 equiv.) and Et_3N (0.12 mL, 0.89 mmol, 6.0 equiv.) were added to the solution. The reaction mixture was stirred overnight at room temperature. After LC-MS analysis confirmed complete conversion, the reaction mixture was diluted with CH_2Cl_2 (stabilized with amylene, 50 mL) and it was extracted with concentrated $NaHCO_3$ solution (5 × 30 mL). The organic layer was dried over anhydrous $MgSO_4$, and the solution was concentrated under reduced pressure to yield mixed carbonate **S13-NHS**, which was used in the subsequent steps without further purification.

Carbamate bond formation (step 2): Activated compound **S13-NHS** (calculated: 78 mg, 0.15 mmol, 1.0 equiv.) was dissolved in CH_2Cl_2 (stabilized with amylene, 1.5 mL), then dimethylamine solution (2 M in THF, 0.15 mL, 0.30 mmol, 2.0 equiv.) and Et_3N (0.10 mL, 0.74 mmol, 5.0 equiv.) were added. The solution was stirred overnight at room temperature. After confirming the complete conversion with LC-MS, the solution was concentrated under reduced pressure, and the crude product was purified by HPLC (eluent: 0.1% TFA in H_2O/MeCN, gradient from 5% to 100% MeCN).

Yield: 46 mg (68% for the 2 steps)

^1H NMR (500 MHz, $CDCl_3$) δ 7.31 – 7.27 (m, 1H, contained in this multiplet: 7.29 (d, *J* = 8.2 Hz, 1H), 7.28 (d, *J* = 8.9 Hz, 1H)), 7.20 – 7.11 (m, 1H, contained in this multiplet: 7.17 (d, *J* = 8.2 Hz, 1H), 7.14 (d, *J* = 8.3 Hz, 1H)), 6.92 – 6.78 (m, 4H), 4.95 – 4.85 (m, 1H, contained in this multiplet: 4.92 (qd, J = 6.3, 3.9 Hz), 4.88 (qd,

J = 6.3, 3.9 Hz)), 4.26 – 4.19 (m, 1H, contained in this multiplet: 4.24 (d, *J* = 3.2 Hz, 1H), 4.22 (d, *J* = 3.5 Hz, 1H)), 3.61 – 3.49 (m, 4H), 3.08 (s, 3H), 2.99 (s, 3H), 2.88 (s, 6H), 2.22 – 2.10 (m, 4H), 0.91 (d, J = 6.3 Hz, 3H).

^{13}C NMR (126 MHz, $CDCl_3$) δ 156.21, 154.91, 154.87, 153.70, 153.27, 152.98, 152.49, 151.32, 131.21, 130.85, 130.08, 129.87, 118.30, 117.61, 117.18, 116.87, 110.16, 109.98, 76.51, 76.47, 54.33, 53.68, 42.80, 42.76, 36.77, 36.54, 36.44, 35.83, 24.76, 24.70, 15.20, 15.18.

HRMS: $[M+H]^+$: calcd for $[C_{25}H_{32}N_3O_5]^+$: 454.2336, found: 454.2327.

9-(1-((Dimethylcarbamoyl)oxy)ethyl)-9-hydroxy-6-(pyrrolidin-1-yl)-9H-xanthen-3-yl dimethylcarbamate (3)

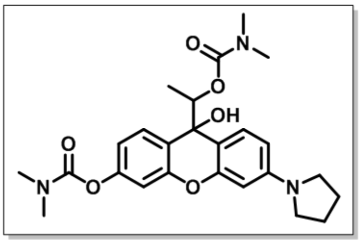

Compound **S14** (46 mg, 0.10 mol, 1.0 equiv.) was dissolved in CH_2Cl_2/MeOH 1:1 (1 mL), then *p*-chloranil (37 mg, 0.15 mmol, 1.5 equiv.) was added. The reaction mixture was stirred for 1 hour at room temperature. Following confirmation of complete conversion via LC-MS, the reaction mixture was diluted with CH_2Cl_2 (50 mL) and extracted with concentrated $NaHCO_3$ solution (2 × 25 mL). After solvent removal under reduced pressure, the crude material was dissolved in MeCN (5 mL), and one drop of TFA was added to convert the compound into its iminium form. This protonated species was purified by HPLC (eluent: 0.1% TFA in H_2O/MeCN, gradient from 5% to 100% MeCN). The product was extracted from the aqueous solution with CH_2Cl_2 (50 mL) and washed with concentrated $NaHCO_3$ solution (2 × 25 mL) to yield the leuco form of the compound. After evaporating the solvents under reduced pressure, a brown solid was obtained.

Yield: 12 mg (25%)

Leuco form: ^{1}H NMR (500 MHz, $CDCl_3$) δ 7.72 – 7.63 (m, 1H, contained in this multiplet: 7.70 (d, *J* = 8.5 Hz, 1H), 7.66 (d, *J* = 8.3 Hz, 1H)), 7.54 – 7.45 (m, 1H, contained in this multiplet: 7.51 (d, *J* = 8.6 Hz, 1H), 7.48 (d, *J* = 8.6 Hz, 1H)), 6.96 – 6.88 (m, 2H), 6.46 – 6.39 (m, 1H, contained in this multiplet: 6.43 (dd, *J* = 8.3, 2.6 Hz, 1H), 6.42 (dd, *J* = 8.5, 2.5 Hz, 1H)), 6.27 – 6.21 (m, 1H, contained in this multiplet: 6.24 (d, *J* = 2.1 Hz, 1H), 6.23 (d, *J* = 2.1 Hz, 1H)), 5.11 – 5.03 (m, 1H, contained in this multiplet: 5.07 (d, *J* = 6.8 Hz, 1H), 5.06 (d, *J* = 6.8 Hz, 1H)), 3.34 – 3.27 (m, 4H), 3.10 (s, 3H), 3.02 (s, 3H), 2.96 – 2.86 (m, 6H), 2.04 – 1.97 (m, 4H), 0.87 – 0.80 (m, 3H, contained in this multiplet: 0.84 (d, *J* = 6.4 Hz, 3H), 0.83 (d, *J* = 6.5 Hz, 1H)).

^{13}C NMR (75 MHz, CD_3CN) δ 156.82, 156.78, 155.34, 155.32, 152.97, 152.86, 152.82, 152.73, 152.57, 152.56, 149.93, 149.83, 129.31, 129.06, 128.95, 128.81, 123.54, 123.28, 117.34, 112.15, 110.12, 109.82, 108.86, 108.78, 98.37, 98.08, 79.35, 70.98, 48.42, 36.93, 36.79, 36.62, 36.01, 26.09, 15.78, 15.72.

Iminium form: ^{1}H NMR (500 MHz, $CDCl_3$) δ 8.42 (d, *J* = 9.9 Hz, 1H), 8.37 (d, *J* = 9.2 Hz, 1H), 7.59 (d, *J* = 2.3 Hz, 1H), 7.40 (dd, *J* = 9.2, 2.3 Hz, 1H), 7.32 (dd, *J* = 9.9, 2.1 Hz, 1H), 6.74 (d, *J* = 2.1 Hz, 1H), 6.61 (q, *J* = 7.1 Hz, 1H), 3.95 – 3.81 (m, 2H), 3.78 – 3.70 (m, 2H), 3.15 (s, 3H), 3.06 (s, 3H), 3.02 (s, 3H), 2.82 (s, 3H), 2.25 – 2.12 (m, 4H), 1.88 (d, *J* = 7.0 Hz, 3H).

HRMS: $[M+H]^+$: calcd for $[C_{25}H_{32}N_3O_6]^+$: 470.2285, found: 470.2286; $[M-H_2O+H]^+$: calcd for $[C_{25}H_{30}N_3O_5]^+$: 452.2179, found: 452.2184.

tert-Butyl (9-(1-hydroxyethyl)-6-(pyrrolidin-1-yl)-9H-xanthen-3-yl) ethane-1,2-diylbis(methylcarbamate) (S15)

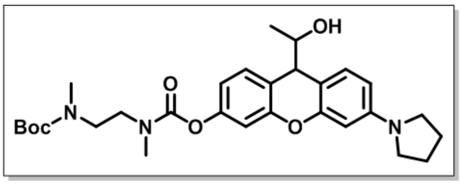

Compound **S09** (50 mg, 0.16 mmol, 1.0 equiv.) and DMAP (1.0 mg, 8.0 μmol, 0.05 equiv.) were dissolved in MeCN (2 mL), then **L-NMI** [2] (75 mg, 0.18 mmol, 1.1 equiv.) and Et_3N (0.11 mL, 0.80 mmol, 5.0 equiv.) were added. The reaction mixture was stirred at room temperature overnight. After confirming the completion of the reaction with LC-MS, the solvent was removed under reduced pressure, and the crude product was purified by HPLC (eluent: 0.1% TFA in H_2O/MeCN, gradient from 5% to 100% MeCN) to yield a brown solid.

Yield: 53 mg (63%)

^{1}H NMR (500 MHz, $CDCl_3$) δ 7.25 – 7.03 (m, 2H, contained in this multiplet: 7.23 (d, J = 8.3 Hz, 1H), 7.17 (d, J = 8.2 Hz, 1H), 7.12 (d, J = 8.3 Hz, 1H), 7.05 (d, J = 8.3 Hz, 1H)), 6.88 – 6.78 (m, 2H), 6.49 – 6.36 (m, 2H, contained in this multiplet: 6.46 (dd, J = 8.2, 2.2 Hz, 1H), 6.45 (dd, J = 8.2, 2.1 Hz, 1H), 6.42 (d, J = 2.3 Hz, 1H), 6.39 (d, J = 2.1 Hz, 1H), 3.87 – 3.74 (m, 2H), 3.59 – 3.40 (m, 4H), 3.35 – 3.27 (m, 4H), 3.12 – 2.87 (m, 6H), 2.06 – 1.98 (m, 4H), 1.49 – 1.40 (m, 9H), 0.97 – 0.90 (m, 3H).

^{13}C NMR (126 MHz, $CDCl_3$) δ 154.67, 153.50, 153.42, 153.24, 153.11, 150.82, 147.18, 147.10, 130.24, 130.00, 129.88, 129.77, 116.47, 116.46, 116.31, 116.29, 111.05, 110.24, 109.88, 109.00, 108.78, 100.67, 73.29, 73.12, 49.41, 49.22, 47.09, 45.81, 35.34, 34.71, 28.47, 25.29, 25.27, 18.61, 18.51.

HRMS: $[M+H]^+$: calcd for $[C_{29}H_{40}N_3O_6]^+$: 597.3282, found: 597.3274.

tert-Butyl (9-(1-((dimethylcarbamoyl)oxy)ethyl)-6-(pyrrolidin-1-yl)-9H-xanthen-3-yl) ethane-1,2-diylbis(methylcarbamate) (S16)

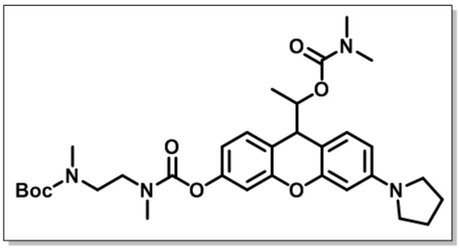

Activation with DSC (step 1): Compound **S15** (84 mg, 0.16 mmol, 1.0 equiv.) was dissolved in CH_2Cl_2 (stabilized with amylene, 1.5 mL), then *N,N'*-disuccinimidyl carbonate (0.16 g, 0.64 mmol, 4.0 equiv.) and Et_3N (0.13 mL, 0.96 mmol, 6.0 equiv.) were added to the solution. The reaction mixture was stirred overnight at room temperature. After establishing complete conversion through LC-MS, the reaction mixture was diluted with CH_2Cl_2 (stabilized with amylene, 50 mL), and it was extracted with concentrated $NaHCO_3$ solution (5 × 30 mL). The organic layer was dried over anhydrous $MgSO_4$, and the solution was concentrated under reduced pressure to yield mixed carbonate **S15-NHS**, which was used in the subsequent steps without further purification.

Carbamate bond formation (step 2): Activated compound **S24** (calculated: 0.11 g, 0.16 mmol, 1.0 equiv.) was dissolved in CH_2Cl_2 (stabilized with amylene, 1.5 mL), then dimethylamine solution (2 M in THF, 0.16 mL, 0.32 mmol, 2.0 equiv.) and Et_3N (0.11 mL, 0.80 mmol, 5.0 equiv.) were added. The solution was stirred overnight at room temperature. Upon verification of complete conversion using LC-MS, the solution was concentrated under reduced pressure, and the crude product was purified by HPLC (eluent: 0.1% TFA in H_2O/MeCN, gradient from 5% to 100% MeCN).

Yield: 53 mg (56% for the 2 steps)

^{1}H NMR (500 MHz, $CDCl_3$) δ 7.31 – 7.27 (m, 1H, contained in this multiplet: 7.29 (d, J = 8.2 Hz, 1H), 7.28 (d, J = 8.9 Hz, 1H)), 7.19 – 7.11 (m, 1H, contained in this multiplet: 7.17 (d, J = 8.2 Hz, 1H), 7.14 (d, J = 8.3 Hz, 1H)), 6.90 – 6.79 (m, 4H), 4.95 – 4.84 (m, 1H, contained in this multiplet: 4.91 (qd, J = 6.3, 3.9 Hz, 1H), 4.89 (qd, J = 6.3, 3.9 Hz, 1H)), 4.26 – 4.19 (m, 1H, contained in this multiplet: 4.24 (d, J = 3.2 Hz, 1H), 4.22 (d, J = 3.5 Hz, 1H)), 3.61 – 3.48 (m, 4H), 3.08 (s, 3H), 2.99 (s, 3H), 2.88 (s, 6H), 2.16 (d, J = 11.2 Hz, 4H), 0.91 (d, J = 6.3 Hz, 3H).
HRMS: $[M+H]^+$: calcd for $[C_{32}H_{45}N_4O_7]^+$: 597.3282, found: 597.3274.

tert-Butyl (9-(1-((dimethylcarbamoyl)oxy)ethyl)-9-hydroxy-6-(pyrrolidin-1-yl)-9H-xanthen-3-yl) ethane-1,2-diylbis(methylcarbamate) (5)

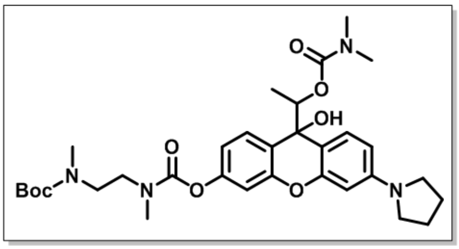

Compound **S16** (53 mg, 89 µmol, 1.0 equiv.) was dissolved in CH_2Cl_2/MeOH 1:1 (1 mL), then *p*-chloranil (24 mg, 98 µmol, 1.1 equiv.) was added. The reaction mixture was stirred for 1.5 hours at room temperature. After confirming the completion of the reaction with LC-MS, the reaction mixture was diluted with CH_2Cl_2 (50 mL) and extracted with concentrated $NaHCO_3$ solution (2 × 25 mL). After solvent removal under reduced pressure, the crude material was dissolved in MeCN (5 mL), and one drop of TFA was added to convert the compound into its iminium form. This protonated species was purified by HPLC (eluent: 0.1% TFA in H_2O/MeCN, gradient from 5% to 100% MeCN). The product was extracted from the aqueous solution with CH_2Cl_2 (50 mL) and washed with concentrated $NaHCO_3$ solution (2 × 25 mL) to yield the leuco form of the compound. After evaporating the solvents under reduced pressure, a purple solid (**5**) was obtained.

Yield: 21 mg (39%)

^{1}H NMR (500 MHz, CD_3CN) δ 7.71 – 7.44 (m, 1H), 7.42 – 7.26 (m, 1H), 7.00 – 6.81 (m, 2H), 6.53 – 6.42 (m, 1H), 6.31 – 6.19 (m, 1H), 5.02 – 4.80 (m, 1H), 3.61 – 3.40 (m, 4H), 3.34 – 3.26 (m, 4H), 3.12 – 2.89 (m, 6H), 2.82 – 2.71 (m, 6H), 2.04 – 1.97 (m, 4H), 1.47 – 1.38 (m, 9H), 0.93 – 0.83 (m, 3H).

HRMS: $[M+H]^+$: calcd for $[C_{32}H_{45}N_4O_8]^+$: 613.3231, found: 613.3241; $[M-H_2O+H]^+$: calcd for $[C_{32}H_{43}N_4O_7]^+$: 595.3126, found: 595.3134.

2.4 Synthesis of the rTCO-Rho Fluorogenic Dye

Compound **S21** [3] and (*E*)-cyclooct-2-en-1-yl (2,5-dioxopyrrolidin-1-yl) carbonate (**S20a-NHS**) [4] were synthesized according to published procedures.

(E)-Cyclooct-2-en-1-yl (3'-oxo-2,3,6,7-tetrahydro-1H,3'H,5H-spiro[chromeno[2,3-f]pyrido[3,2,1-ij]quinoline-9,1'-isobenzofuran]-12-yl) ethane-1,2-diylbis(methylcarbamate) (rTCO-Rho)

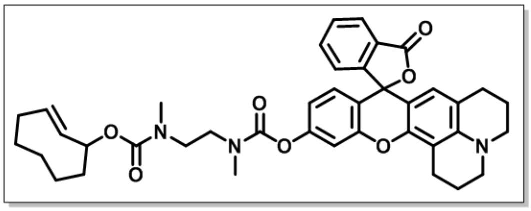

Deprotection (step 1): To a solution of **S21** [3] (63 mg, 0.10 mmol, 1.0 equiv.) in CH_2Cl_2 (2 mL), TFA (1 mL) was added, and the solution was stirred at room temperature for 1 h. The solvent was removed in vacuo, and the residue was dissolved in CH_2Cl_2 (2 mL). The solvent was then evaporated five times, and the crude product was dried in high vacuum.

Carbamate bond formation (step 2): The crude deprotected linker-rhodol and **S20a-NHS** [4] (27 mg, 0.10 mmol, 1.0 equiv.) were dissolved in CH_2Cl_2 (2 mL), and triethylamine (0.07 mL, 0.5 mmol, 5 equiv.) in CH_2Cl_2 (1 mL) was added dropwise. After stirring at room temperature for 15 min, the reaction mixture was purified by flash chromatography on silica (eluent: CH_2Cl_2/MeOH 0% to 10%).

Yield: 62 mg (91% for the 2 steps)

^{1}H NMR (500 MHz, $CDCl_3$) δ 7.99 (d, *J* = 7.5 Hz, 1H), 7.64 (t, *J* = 7.2 Hz, 1H), 7.59 (t, *J* = 7.3 Hz, 1H), 7.18 (d, *J* = 7.2 Hz, 1H), 7.09 (d, *J* = 2.5 Hz, 1H), 6.76 – 6.68 (m, 2H), 6.13 (s, 1H), 5.83 – 5.71 (m, 1H), 5.59 – 5.50 (m, 1H), 5.42 – 5.34 (m, 1H), 3.67 – 3.45 (m, 4H), 3.17 (t, *J* = 5.6 Hz, 2H), 3.14 (t, *J* = 5.6 Hz, 2H), 3.12 – 2.96 (m, 6H), 2.91 (t, *J* = 6.1 Hz, 2H), 2.60 – 2.46 (m, 2H), 2.45 – 2.34 (m, 1H), 2.08 – 1.94 (m, 5H), 1.91 – 1.79 (m, 3H), 1.72 – 1.64 (m, 2H), 1.54 – 1.39 (m, 1H), 1.12 – 0.98 (m, 1H), 0.84 – 0.73 (m, 1H).

^{13}C NMR (126 MHz, $CDCl_3$) δ 169.79, 154.15, 153.34, 152.47, 147.92, 144.73, 134.85, 131.92, 131.62, 129.56, 128.92, 127.31, 124.95, 124.89, 124.38, 118.08, 116.98, 110.36, 107.38, 105.02, 84.56, 74.76, 50.01, 49.56, 48.07, 47.17, 46.90, 46.31, 41.01, 40.95, 36.15, 36.02, 29.27, 27.48, 24.34, 21.93, 21.35, 21.13.

HRMS: $[M+H]^+$: calcd for $[C_{40}H_{44}N_3O_7]^+$: 678.3173, found: 678.3145

2.5 Synthesis of the Coumarin Conjugates

Compounds **S22** [5], **S29** [6], and **S29-NHS** [7] were synthesized according to published procedures.

tert-Butyl 4-(11-oxo-2,3,6,7-tetrahydro-1H,5H,11H-pyrano[2,3-f]pyrido[3,2,1-ij]quinoline-10-carbonyl)piperazine-1-carboxylate (S23)

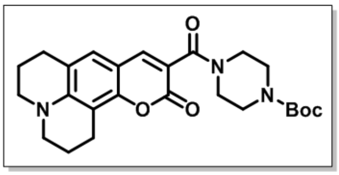

Compound **S22** [5] (100 mg, 351 μmol, 1.0 equiv.), 1-Boc-piperazine (131 mg, 701 μmol, 2.0 equiv.), and HATU (200 mg, 526 μmol, 1.5 equiv.) were dissolved in dry DMF (2 mL), then Et_3N (195 μL, 1.40 mmol, 4.0 equiv.) was added. The reaction mixture was stirred for 1 hour at room temperature. After the completion of the reaction was confirmed by LC-MS, the reaction mixture was diluted with water (10 mL), 10% citric acid (15 mL), and brine (15 mL). This aqueous solution was extracted with EtOAc (3 × 25 mL). The combined organic layers were washed with concentrated Na_2CO_3 solution (2 × 25 mL) and brine (4 × 25 mL). The organic layer was dried over anhydrous $MgSO_4$, and the solvent was removed under reduced pressure. The crude product was purified by flash chromatography on silica (eluent: CH_2Cl_2/MeOH 0% to 15%) to yield a bright yellow solid.

Yield: 151 mg (95%)

^{1}H NMR (500 MHz, $CDCl_3$) δ 7.74 (s, 1H), 6.85 (s, 1H), 3.71 – 3.59 (m, 2H), 3.50 – 3.41 (m, 4H), 3.36 – 3.29 (m, 2H), 3.29 – 3.23 (m, 4H), 2.82 (t, J = 6.5 Hz, 2H), 2.71 (t, J = 6.1 Hz, 2H), 1.96 – 1.87 (m, 4H), 1.42 (s, 9H).

^{13}C NMR (126 MHz, $CDCl_3$) δ 165.86, 159.62, 154.73, 152.38, 147.41, 145.87, 126.01, 119.25, 114.33, 107.65, 106.07, 80.32, 50.15, 49.75, 28.38, 27.48, 21.27, 20.33, 20.13.

HRMS: $[M+H]^+$: calcd for $[C_{25}H_{32}N_3O_5]^+$: 454.2336, found: 454.2340.

1-(3-(Methoxymethoxy)-6-(pyrrolidin-1-yl)-9H-xanthen-9-yl)ethyl 4-(11-oxo-2,3,6,7-tetrahydro-1H,5H,11H-pyrano[2,3-f]pyrido[3,2,1-ij]quinoline-10-carbonyl)piperazine-1-carboxylate (S24)

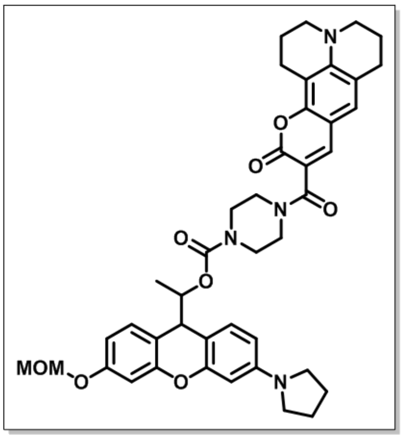

Deprotection (step 1): Compound **S23** (130 mg, 287 µmol, 1.0 equiv.) was treated with TFA (250 µL, 3.25 mmol, 11 equiv.) in CH_2Cl_2 (1 mL). The reaction mixture was stirred at room temperature for 1 hour. After confirming full conversion by LC-MS, the volatiles were removed under reduced pressure, and the deprotected compound was used in the subsequent step without further purification.

Carbamate bond formation (step 2): Deprotected **S23** (calculated as trifluoroacetate salt: 134 mg, 287 µmol, 1.0 equiv.) and compound **S07-NHS** (0.14 g, 0.28 mmol, 1.0 equiv.) were dissolved in CH_2Cl_2 (2 mL), then Et_3N (0.24 mL, 1.7 mmol, 6.1 equiv.) was added. The reaction mixture was stirred at room temperature overnight, until the complete conversion of the starting materials was verified by LC-MS. The solvent was removed under reduced pressure, and the crude product was purified by HPLC (eluent: 0.1% TFA in H_2O/MeCN, gradient from 5% to 100% MeCN).

Yield: 113 mg (55% for the 2 steps)

^{1}H NMR (500 MHz, CD_3CN) δ 7.68 (s, 1H), 7.24 – 7.12 (m, 2H), 6.93 (s, 1H), 6.81 – 6.54 (m, 4H), 5.16 (s, 2H), 4.93 – 4.80 (m, 1H), 4.47 (s, 8H), 4.11 – 4.03 (m, 1H, contained in this multiplet: 4.08 (d, J = 3.8 Hz, 1H), 4.07 (d, J = 3.9 Hz, 1H)), 3.45 – 3.36 (m, 7H), 3.28 – 3.24 (m, 4H), 2.75 (t, J = 6.2 Hz, 2H), 2.70 (t, J = 6.1 Hz, 2H), 2.09 – 2.01 (m, 4H), 1.93 – 1.84 (m, 4H), 0.95 (d, J = 6.4 Hz, 3H).

^{13}C NMR (75 MHz, CD_3CN) δ 166.65, 160.23, 159.65, 158.23, 158.15, 155.14, 154.34, 154.28, 154.20, 154.12, 153.09, 148.15, 145.32, 131.57, 131.40, 131.29, 126.91, 120.22, 118.58, 116.14, 115.76, 115.29, 114.76, 112.67, 112.45, 108.17, 106.56, 104.84, 104.55, 95.36, 95.33, 77.32, 56.38, 52.58, 52.19, 50.67, 50.19, 49.88, 44.50, 44.17, 43.20, 28.01, 25.49, 25.41, 21.91, 20.99, 20.78, 16.51, 16.48.

HRMS: $[M+H]^+$: calcd for $[C_{42}H_{47}N_4O_8]^+$: 735.3388, found: 735.3372.

1-(3-(((2-((tert-Butoxycarbonyl)(methyl)amino)ethyl)(methyl)carbamoyl)oxy)-6-(pyrrolidin-1-yl)-9H-xanthen-9-yl)ethyl 4-(11-oxo-2,3,6,7-tetrahydro-1H,5H,11H-pyrano[2,3-f]pyrido[3,2,1-ij]quinoline-10-carbonyl)piperazine-1-carboxylate (S25)

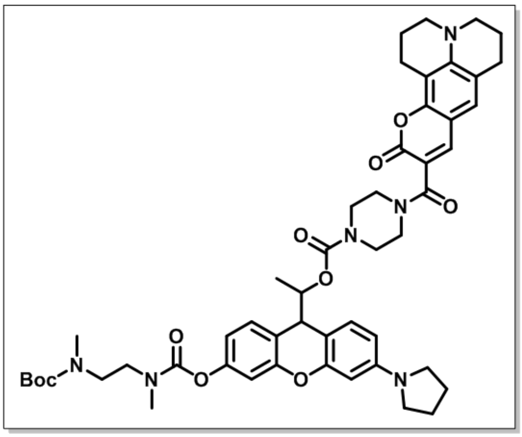

Deprotection (step 1): Compound **S24** (110 mg, 150 µmol, 1.0 equiv.) was treated with TMS-Cl (100 µL, 788 µmol, 5.3 equiv.) in CH_2Cl_2/MeOH 1:1 solvent mixture (1.5 mL). The reaction mixture was stirred at room temperature for 1.5 hours. The volatiles were removed under reduced pressure, and the crude product was used in the subsequent step without further purification.

Carbamate bond formation (step 2): The crude intermediate (calculated: 104 mg, 150 µmol, 1.0 equiv.) and DMAP (1 mg, 8 µmol, 0.05 equiv.) were dissolved In MeCN (1.5 mL), then **L-NMI** [2] (80 mg, 0.19 mmol, 1.3 equiv.) and Et_3N (105 µL, 750 µmol, 5.0 equiv.) were added. The reaction mixture was stirred at 75 °C for 2.5 hours. After LC-MS analysis confirmed completion, the solvent was removed under reduced pressure, and the crude product was purified by HPLC (eluent: 0.1% TFA in H_2O/MeCN, gradient from 5% to 100% MeCN) to yield a yellow solid.

Yield: 91 mg (67% for the 2 steps)

^{1}H NMR (500 MHz, CD_3CN) δ 7.70 (s, 1H), 7.33 – 7.20 (m, 1H, contained in this multiplet: 7.29 (d, *J* = 8.0 Hz, 1H), 7.24 (d, *J* = 8.3 Hz, 1H)), 7.14 – 7.04 (m, 1H, contained in this multiplet: 7.12 (d, *J* = 8.4 Hz, 1H), 7.08 (d, *J* = 8.4 Hz, 1H)), 6.99 (s, 1H), 6.89 – 6.79 (m, 2H), 6.40 (dd, *J* = 8.5, 2.6 Hz, 1H), 6.30 – 6.20 (m, 1H), 4.95 – 4.83 (m, 1H), 4.15 – 4.05 (m, 1H), 3.75 – 3.38 (m, 8H), 3.37 – 3.20 (m, 12H), 3.10 – 2.83 (m, 6H), 2.81 (t, *J* = 6.3 Hz, 2H), 2.75 (t, *J* = 6.2 Hz, 2H), 2.03 – 1.97 (m, 4H), 1.97 – 1.89 (m, 4H), 1.49 – 1.35 (m, 9H), 1.03 – 0.94 (m, 3H).

HRMS: $[M+H]^+$: calcd for $[C_{50}H_{61}N_6O_{10}]^+$: 905.4443, found: 905.4432.

(E)-1-(3-(((2-(((Cyclooct-2-en-1-yloxy)carbonyl)(methyl)amino)ethyl)(methyl)carbamoyl)oxy)-6-(pyrrolidin-1-yl)-9H-xanthen-9-yl)ethyl 4-(11-oxo-2,3,6,7-tetrahydro-1H,5H,11H-pyrano[2,3-f]pyrido[3,2,1-ij]quinoline-10-carbonyl)piperazine-1-carboxylate (S26)

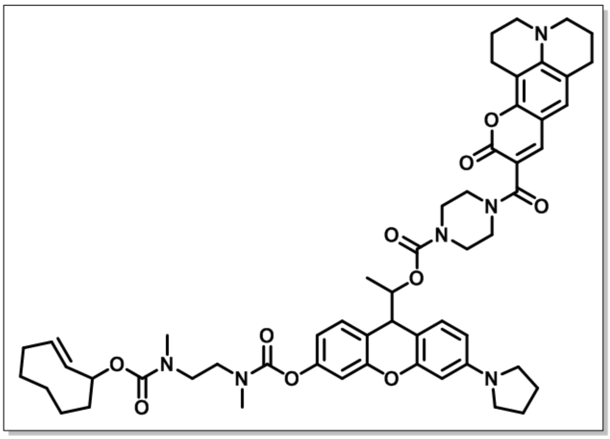

Deprotection (step 1): Compound **S25** (91 mg, 0.10 mmol, 1.0 equiv.) was dissolved in CH_2Cl_2 (1 mL), and TFA (200 µL) was added. The reaction mixture was stirred at room temperature for 1 hour. The volatiles were evaporated under reduced pressure, and the crude intermediate was used in the subsequent step without further purification.

Carbamate bond formation (step 2): Deprotected **S25** (calculated as trifluoroacetate salt: 92 mg, 0.10 mmol, 1.0 equiv.) was dissolved in MeCN (2 mL), then **S20a-NHS** [4] (29 mg, 0.11 mmol, 1.1 equiv.) and Et_3N (70 µL, 0.50 mmol, 5.0 equiv.) were added. The reaction mixture was stirred at room temperature overnight. After the complete conversion of the starting materials was confirmed with LC-MS, the solvent was removed under reduced pressure. The crude material was purified by HPLC (eluent: 0.1% HCOOH in H_2O/MeCN, gradient from 5% to 100% MeCN). The purified product was extracted from the aqueous solution by extraction with CH_2Cl_2 (2 × 25 mL), and the organic layer was washed with concentrated $NaHCO_3$ solution (30 mL), then the solvent was evaporated to yield product **S26**.

Yield: 55 mg (57% for the 2 steps)

^{1}H NMR (500 MHz, CD_2Cl_2) δ 7.72 (s, 1H), 7.29 – 7.13 (m, 1H, contained in this multiplet: 7.27 (d, *J* = 6.2 Hz, 1H), 7.15 (d, *J* = 7.3 Hz, 1H)), 7.13 – 6.97 (m, 1H, contained in this multiplet: 7.11 (d, *J* = 8.4 Hz, 1H), 7.00 (d, *J* = 8.3 Hz, 1H)), 6.92 (s, 1H), 6.87 – 6.78 (m, 2H), 6.36 (dd, *J* = 8.3, 2.3 Hz, 1H), 6.29 – 6.20 (m,

1H), 5.87 – 5.72 (m, 1H), 5.60 – 5.44 (m, 1H), 5.36 – 5.33 (m, 1H), 5.00 – 4.89 (m, 1H), 4.20 – 4.08 (m, 1H), 3.80 – 3.42 (m, 10H), 3.39 – 3.23 (m, 10H), 3.12 – 2.92 (m, 6H), 2.86 (t, *J* = 6.1 Hz, 2H), 2.76 (t, *J* = 6.1 Hz, 2H), 2.49 – 2.30 (m, 1H), 2.07 – 1.93 (m, 11H), 1.90 – 1.79 (m, 1H), 1.72 – 1.59 (m, 2H), 1.56 – 1.42 (m, 1H), 1.18 – 1.05 (m, 1H), 0.96 (d, *J* = 6.2 Hz, 3H), 0.86 – 0.74 (m, 1H).

HRMS: $[M+H]^+$: calcd for $[C_{54}H_{65}N_6O_{10}]^+$: 957.4756, found: 957.4750.

(E)-1-(3-(((2-(((Cyclooct-2-en-1-yloxy)carbonyl)(methyl)amino)ethyl)(methyl)carbamoyl)oxy)-9-hydroxy-6-(pyrrolidin-1-yl)-9H-xanthen-9-yl)ethyl 4-(11-oxo-2,3,6,7-tetrahydro-1H,5H,11H-pyrano[2,3-f]pyrido[3,2,1-ij]quinoline-10-carbonyl)piperazine-1-carboxylate (rTCO-1-Cou)

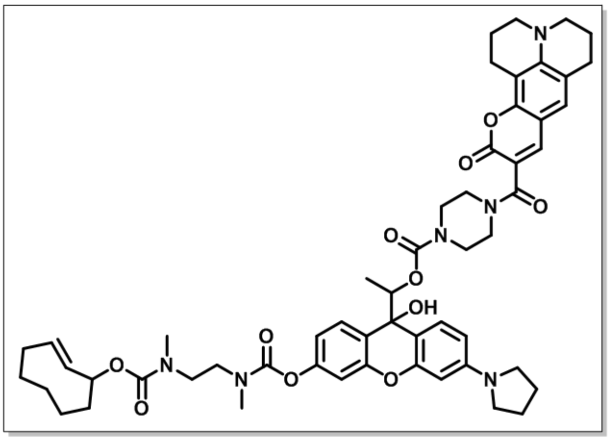

Compound **S26** (55 mg, 57 µmol, 1.0 equiv.) was dissolved in CH_2Cl_2/MeOH 1:1 solvent mixture (2 mL), then *p*-chloranil (21 mg, 86 µmol, 1.5 equiv.) was added, and the reaction mixture was stirred at room temperature for 2 hours. Upon confirmation of full conversion via LC-MS analysis, the solvents were removed under reduced pressure, and the crude material was purified by HPLC (eluent: 0.1% HCOOH in H_2O/MeCN, gradient from 5% to 100% MeCN). The purified product was extracted from the aqueous solution with CH_2Cl_2 (2 × 25 mL), and the organic layer was washed with concentrated $NaHCO_3$ solution (30 mL), then the solvent was evaporated to yield a yellow solid.

Yield: 23 mg (41%)

^{1}H NMR (500 MHz, $CDCl_3$) δ 7.79 (s, 1H), 7.72 – 7.57 (m, 1H), 7.51 – 7.38 (m, 1H), 6.96 – 6.83 (m, 3H), 6.41 (dd, *J* = 8.5, 2.0 Hz, 1H), 6.26 – 6.15 (m, 1H), 5.84 – 5.72 (m, 1H), 5.59 – 5.48 (m, 1H), 5.41 – 5.32 (m, 1H), 5.10 (q, *J* = 6.1 Hz, 1H), 3.78 – 3.41 (m, 10H), 3.39 – 3.22 (m, 10H), 3.15 – 2.95 (m, 6H), 2.87 (t, *J* = 5.9 Hz, 2H), 2.75 (t, *J* = 6.0 Hz, 2H), 2.51 – 2.29 (m, 1H), 2.11 – 1.92 (m, 10H), 1.90 – 1.80 (m, 1H), 1.73 – 1.62 (m, 2H), 1.52 – 1.43 (m, 1H), 1.31 – 1.26 (m, 1H), 1.15 – 1.02 (m, 1H), 0.91 (d, *J* = 6.0 Hz, 3H), 0.85 – 0.75 (m, 1H).

HRMS: $[M-H_2O+H]^+$: calcd for $[C_{54}H_{63}N_6O_{10}]^+$: 955.4600, found: 955.4595.

9-(1-Hydroxyethyl)-6-(pyrrolidin-1-yl)-9H-xanthen-3-yl (1-(6-isopropyl-1,2,4,5-tetrazin-3-yl)ethyl) ethane-1,2-diylbis(methylcarbamate) (S30)

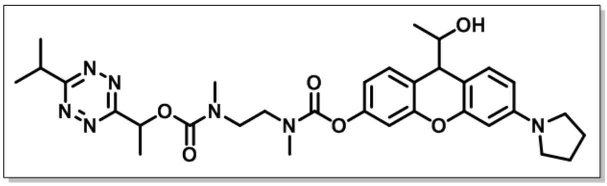

Deprotection (step 1): Compound **S15** (149 mg, 283 µmol, 1.0 equiv.) was dissolved in CH_2Cl_2 (4 mL), followed by the addition of TFA (2 mL) and a few drops of water. The reaction mixture was stirred at room temperature for 15 min. Upon completion, the solvent was evaporated in vacuo. The crude product was used immediately without further purification.

Carbamate bond formation (step 2): The crude deprotected **S15** (0.283 mmol, 1.0 equiv.) was dissolved in dry CH_2Cl_2 (stabilized with amylene) and added dropwise to a stirred solution of crude **S29-NHS** [7] (calculated: 88 mg, 0.28 mmol, 1.0 equiv.) and Et_3N (100 µL, 0.714 mmol, 2.5 equiv.) in dry CH_2Cl_2 (stabilized with amylene). The mixture was stirred at room temperature for 1 hour and the reaction was monitored by LC-MS. Upon completion, the solvent was removed in vacuo, and the crude product was purified by HPLC (eluent: 0.1% TFA in H_2O/MeCN, gradient from 5% to 100% MeCN).

Yield: 35 mg (20% for the 2 steps)

^{1}H NMR (500 MHz, $CDCl_3$) δ 7.25 – 7.14 (m, 1H), 7.11 – 6.99 (m, 1H), 6.92 – 6.75 (m, 2H), 6.36 – 6.29 (m, 1H), 6.29 – 6.26 (m, 1H), 6.28 – 6.17 (m, 1H), 3.88 – 3.76 (m, 2H), 3.68 – 3.49 (m, 4H), 3.45 – 3.34 (m, 1H),

3.32 – 3.22 (m, 4H), 3.18 – 2.95 (m, 6H), 2.04 – 1.95 (m, 4H), 1.86 – 1.72 (m, 3H), 1.54 – 1.10 (m, 6H), 1.02 – 0.90 (m, 3H).

HRMS: $[M+H]^+$: calcd for $[C_{32}H_{42}N_7O_6]^+$: 620.3191, found: 620.3194.

1-(3-(((2-(((1-(6-Isopropyl-1,2,4,5-tetrazin-3-yl)ethoxy)carbonyl)(methyl)amino)ethyl)(methyl)carbamoyl)oxy)-6-(pyrrolidin-1-yl)-9H-xanthen-9-yl)ethyl 4-(11-oxo-2,3,6,7-tetrahydro-1H,5H,11H-pyrano[2,3-f]pyrido[3,2,1-ij]quinoline-10-carbonyl)piperazine-1-carboxylate (S31)

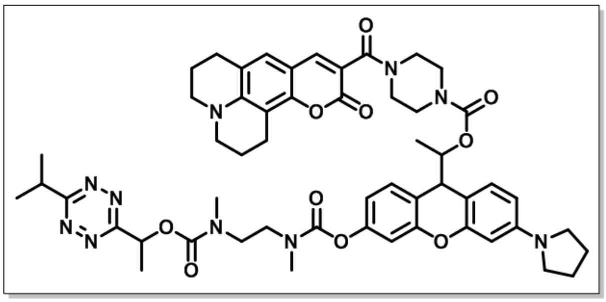

DSC activation (step 1): Compound **S30** (35 mg, 56 µmol, 1.0 equiv.), *N,N'*-disuccinimidyl carbonate (72 mg, 0.28 mmol, 5.0 equiv.), DMAP (4 mg, 0.03 mmol, 0.5 equiv.) and Et_3N (39 µL, 0.28 mmol, 5.0 equiv.) were dissolved in dry CH_2Cl_2 (stabilized with amylene) and the mixture was stirred at room temperature. The reaction was monitored by TLC (CH_2Cl_2/MeOH 9:1). Upon completion (1.5 hours), the solvent was evaporated under reduced pressure. The residue was redissolved in ethyl acetate, and the organic layer was washed with water five times, then with concentrated $NaHCO_3$ solution also five times. The organic phase was dried on anhydrous $MgSO_4$, then the solvent was evaporated in vacuo. The crude product (**S30-NHS**) was used immediately without further purification.

Carbamate bond formation (step 2): Crude **S30-NHS** (calculated: 43 mg, 56 µmol, 1.0 equiv.) was dissolved in dry CH_2Cl_2 (stabilized with amylene). Triethylamine was added (23 µL, 0.17 mmol, 3.0 equiv.), then the solution of crude deprotected **S23** (56 µmol, 1.0 equiv.) in dry CH_2Cl_2 (stabilized with amylene) was also added dropwise. The reaction mixture was monitored by LC-MS and was stirred at room temperature until completion (1 hour). Then the solvent was removed in vacuo, and the crude product was purified by HPLC (eluent: 0.1% TFA in H_2O/MeCN, gradient from 5% to 100% MeCN).

Yield: 12 mg (21% for the 2 steps)

^1H NMR (500 MHz, $CDCl_3$) δ 7.84 – 7.75 (m, 1H), 7.14 – 6.77 (m, 5H), 6.36 – 6.15 (m, 3H), 5.03 – 4.93 (m, 1H), 4.21 – 4.08 (m, 1H), 3.79 – 3.36 (m, 13H), 3.34 – 3.25 (m, 8H), 3.19 – 2.96 (m, 6H), 2.88 (t, *J* = 5.4 Hz, 2H), 2.76 (t, *J* = 6.0 Hz, 2H), 2.06 – 1.94 (m, 8H), 1.86 – 1.76 (m, 3H), 1.52 (d, *J* = 6.6 Hz, 6H), 1.02 – 0.92 (m, 3H).

HRMS: $[M+2H]^{2+}$: calcd for $[C_{53}H_{64}N_{10}O_{10}]^{2+}$: 500.2397, found: 500.2399.

1-(6-(((2-(((1-(6-Isopropyl-1,2,4,5-tetrazin-3-yl)ethoxy)carbonyl)(methyl)amino)ethyl)(methyl)carbamoyl)oxy)-9-(1-((4-(11-oxo-2,3,6,7-tetrahydro-1H,5H,11H-pyrano[2,3-f]pyrido[3,2,1-ij]quinoline-10-carbonyl)piperazine-1-carbonyl)oxy)ethyl)-3H-xanthen-3-ylidene)pyrrolidin-1-ium trifluoroacetate (rTz-1-Cou)

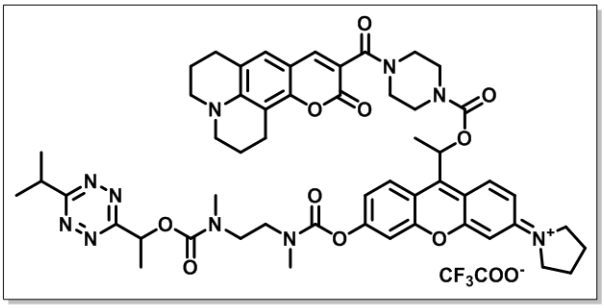

Compound **S31** (12 mg, 12 µmol, 1.0 equiv.) was dissolved in CH_2Cl_2/MeOH 1:1 (1 mL), then *p*-chloranil (12 mg, 48 µmol, 4.0 equiv.) was added. The mixture was stirred at room temperature, and the reaction was monitored by LC-MS. Upon completion (2 hours), the solvent was removed in vacuo. The crude product was purified by HPLC (eluent: 0.1% TFA in H_2O/MeCN, gradient from 5% to 100% MeCN). The product was further filtered on neutral aluminum oxide in a Pasteur pipette (eluent: CH_2Cl_2/MeOH 0% to 5%) to remove the residual hydroquinone.

Yield: 5.5 mg (46%)

Iminium form: ^1H NMR (500 MHz, CD_3CN) δ 8.55 – 8.46 (m, 1H), 8.42 (d, *J* = 9.7 Hz, 1H), 7.71 (s, 1H), 7.63 – 7.49 (m, 1H), 7.47 – 7.35 (m, 1H), 7.30 (dd, *J* = 9.8, 1.6 Hz, 1H), 6.99 (s, 1H), 6.83 (d, *J* = 2.2 Hz, 1H), 6.69

(q, J = 6.5 Hz, 1H), 6.21 – 6.07 (m, 1H), 3.89 – 3.81 (m, 2H), 3.76 – 3.49 (m, 10H), 3.44 – 3.25 (m, 8H), 3.19 – 2.90 (m, 6H), 2.82 – 2.69 (m, 4H), 2.19 – 2.13 (m, 4H), 2.12 – 2.08 (m, 2H), 1.94 – 1.91 (m, 2H), 1.88 (d, J = 6.9 Hz, 3H), 1.79 – 1.70 (m, 3H, contained in this multiplet: 1.77 (d, J = 6.5 Hz, 3H), 1.74 (d, J = 6.6 Hz, 3H)), 1.48 – 1.37 (m, 6H).

HRMS: $[M+2H]^{2+}$: calcd for $[C_{53}H_{62}N_{10}O_{10}]^{2+}$: 499.2319, found: 499.2330.

1-(3-Oxo-6-(pyrrolidin-1-yl)-3H-xanthen-9-yl)ethyl 4-(11-oxo-2,3,6,7-tetrahydro-1H,5H,11H-pyrano[2,3-f]pyrido[3,2,1-ij]quinoline-10-carbonyl)piperazine-1-carboxylate (1-Cou)

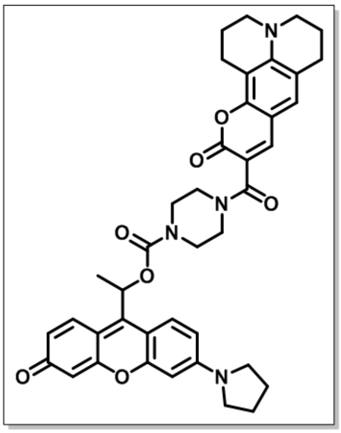

Deprotection (step 1): Compound **S24** (30 mg, 41 µmol, 1.0 equiv.) was treated with TMS-Cl (100 µL, 788 µmol, 19 equiv.) in CH_2Cl_2/MeOH 1:1 solvent mixture (1 mL). The reaction mixture was stirred at room temperature for 30 min. The volatiles were removed under reduced pressure, and the crude product was used in the subsequent step without further purification.

Oxidation (step 2): Deprotected **S24** (calculated: 28 mg, 41 µmol, 1.0 equiv.) was dissolved in CH_2Cl_2/MeOH 1:1 solvent mixture (1 mL), then *p*-chloranil (11 mg, 45 µmol, 1.1 equiv.) was added, and the reaction mixture was stirred at room temperature for 1 hour. After confirming the completion of the reaction with LC-MS, the solvents were removed under reduced pressure, and the crude material was purified by flash chromatography on silica (eluent: CH_2Cl_2/MeOH 0% to 15%) to yield a red solid.

Yield: 23 mg (81%)

^{1}H NMR (500 MHz, $CDCl_3$+CD_3OD) δ 7.98 – 7.82 (m, 2H), 7.74 (s, 1H), 6.85 (s, 1H), 6.67 – 6.59 (m, 2H), 6.55 (q, J = 7.1 Hz, 1H), 6.43 (d, J = 2.1 Hz, 1H), 6.38 (d, J = 2.0 Hz, 1H), 3.86 – 3.31 (m, 12H), 3.29 – 3.23 (m, 4H), 2.80 (t, J = 5.2 Hz, 2H), 2.70 (t, J = 6.0 Hz, 2H), 2.10 – 2.03 (m, 4H), 1.96 – 1.87 (m, 4H), 1.77 (d, J = 6.5 Hz, 3H).

HRMS: $[M+H]^{+}$: calcd for $[C_{40}H_{41}N_4O_7]^{+}$: 689.2969, found: 689.2973.

2.6 Synthesis of the SN38 Conjugates

Compound **S32** was synthesized according to a reported procedure. [1]

(S)-4,11-Diethyl-4-hydroxy-3,14-dioxo-3,4,12,14-tetrahydro-1H-pyrano[3',4':6,7]indolizino[1,2-b]quinolin-9-yl (1-(3-(methoxymethoxy)-6-(pyrrolidin-1-yl)-9H-xanthen-9-yl)ethyl) ethane-1,2-diylbis(methylcarbamate) (S33)

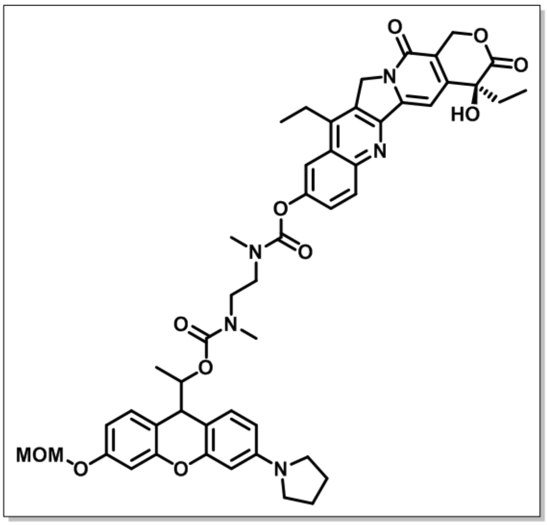

Deprotection (step 1): Compound **S32** (139 mg, 229 μmol, 1.0 equiv.) was dissolved in CH_2Cl_2 (1.125 mL), then TFA (375 μL) was added. The reaction mixture was stirred at room temperature for 1 hour. After the completion of the reaction was confirmed with LC-MS, the volatiles were removed under reduced pressure.

Carbamate bond formation (step 2): Deprotected **S32** (calculated as trifluoroacetate salt: 142 mg, 229 μmol, 1.0 equiv.) and compound **S07-NHS** (0.14 g, 0.28 mmol, 1.2 equiv.) were dissolved in CH_2Cl_2 (2 mL), then Et_3N (0.24 mL, 1.7 mmol, 7.5 equiv.) was added. The reaction mixture was stirred at room temperature overnight. After establishing complete conversion through LC-MS, the solvent was evaporated under reduced pressure. The crude material was purified by HPLC (eluent: 0.1% TFA in H_2O/MeCN, gradient from 5% to 100% MeCN).

Yield: 121 mg (60% for the 2 steps)

^{1}H NMR (500 MHz, $CDCl_3$) δ 8.23 – 8.09 (m, 1H), 7.89 – 7.43 (m, 3H), 7.24 – 6.90 (m, 2H), 6.83 – 6.24 (m, 4H), 5.70 (d, *J* = 16.3 Hz, 1H), 5.28 (d, *J* = 16.0 Hz, 1H), 5.24 – 5.06 (m, 4H), 5.04 – 4.85 (m, 1H), 4.23 – 4.06 (m, 1H), 3.76 – 3.58 (m, 4H), 3.50 – 3.41 (m, 4H), 3.36 – 3.28 (m, 3H), 3.23 – 2.90 (m, 8H), 2.08 – 1.97 (m, 4H), 1.95 – 1.81 (m, 2H), 1.38 – 1.20 (m, 3H), 1.01 (t, J = 7.3 Hz, 3H), 0.96 – 0.82 (m, 3H).

HRMS: $[M+H]^+$: calcd for $[C_{49}H_{54}N_5O_{11}]^+$: 888.3814, found: 888.3798.

1-(3-(((2-((tert-Butoxycarbonyl)(methyl)amino)ethyl)(methyl)carbamoyl)oxy)-6-(pyrrolidin-1-yl)-9H-xanthen-9-yl)ethyl ((S)-4,11-diethyl-4-hydroxy-3,14-dioxo-3,4,12,14-tetrahydro-1H-pyrano[3',4':6,7]indolizino[1,2-b]quinolin-9-yl) ethane-1,2-diylbis(methylcarbamate) (S34)

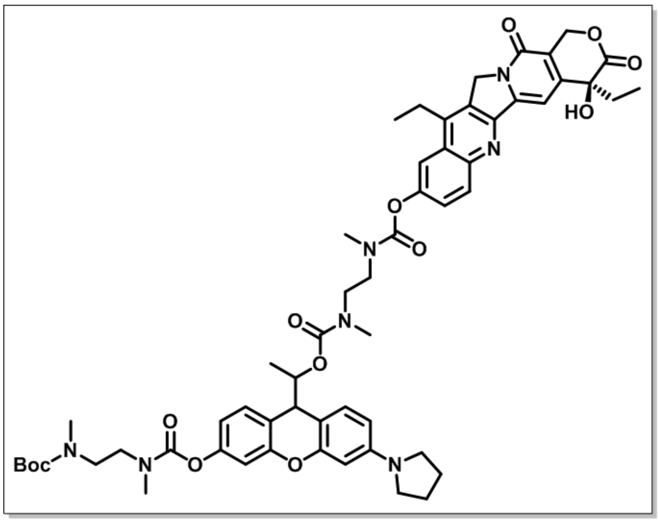

Deprotection (step 1): Compound **S33** (120 mg, 135 μmol, 1.0 equiv.) was treated with TMS-Cl (100 μL, 788 μmol, 5.8 equiv.) in CH_2Cl_2/MeOH 1:1 solvent mixture (1.5 mL). The reaction mixture was stirred at room temperature for 2 hours. The volatiles were removed under reduced pressure, and the crude product was used in the subsequent step without further purification.

Carbamate bond formation (step 2): Deprotected **S33** (calculated: 114 mg, 135 μmol, 1.0 equiv.) and DMAP (1 mg, 8 μmol, 0.06 equiv.) were dissolved in MeCN (1.5 mL), then **L-NMI** [2] (72 mg, 0.17 mmol, 1.3 equiv.) and Et_3N (94 μL, 0.68 mmol, 5.0 equiv.) were added. The reaction mixture was stirred at 75 °C for 2.5 hours. After LC-MS analysis confirmed complete conversion, the solvent was removed under reduced pressure, and the crude product was purified by HPLC (eluent: 0.1% TFA in H_2O/MeCN, gradient from 5% to 100% MeCN) to yield a red solid.

Yield: 75 mg (52% for the 2 steps)

^{1}H NMR (500 MHz, CD_2Cl_2) δ 8.28 – 8.06 (m, 1H), 7.98 – 7.76 (m, 1H), 7.68 – 7.51 (m, 2H), 7.42 – 7.03 (m, 2H), 6.99 – 6.83 (m, 2H), 6.62 – 6.36 (m, 2H), 5.71 (d, J = 16.2 Hz, 1H), 5.37 – 5.28 (m, 3H), 5.08 – 4.93 (m,

1H), 4.31 – 4.18 (m, 1H), 3.88 – 3.44 (m, 8H), 3.43 – 3.30 (m, 4H), 3.25 – 2.93 (m, 14H), 2.14 – 2.04 (m, 4H), 2.00 – 1.87 (m, 2H), 1.54 – 1.45 (m, 9H), 1.43 – 1.30 (m, 3H), 1.05 (t, J = 7.3 Hz, 3H), 1.02 – 0.89 (m, 3H).

HRMS: $[M+H]^+$: calcd for $[C_{57}H_{68}N_7O_{13}]^+$: 1058.4869, found: 1058.4838.

(E)-Cyclooct-2-en-1-yl (9-(1-(((2-(((((S)-4,11-diethyl-4-hydroxy-3,14-dioxo-3,4,12,14-tetrahydro-1H-pyrano[3',4':6,7]indolizino[1,2-b]quinolin-9-yl)oxy)carbonyl)(methyl)amino)ethyl)(methyl)carbamoyl)oxy)ethyl)-6-(pyrrolidin-1-yl)-9H-xanthen-3-yl) ethane-1,2-diylbis(methylcarbamate) (S35)

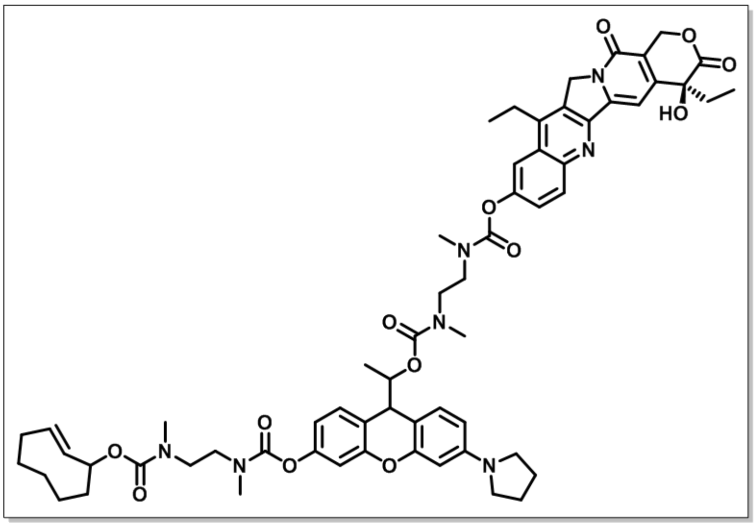

Deprotection (step 1): Compound **S34** (75 mg, 71 µmol, 1.0 equiv.) was dissolved in CH_2Cl_2 (1 mL), and TFA (200 µL) was added. The reaction mixture was stirred at room temperature for 1 hour. The volatiles were evaporated under reduced pressure, and the crude intermediate was used in the subsequent step without further purification.

Carbamate bond formation (step 2): Deprotected **S34** (calculated as trifluoroacetate salt: 76 mg, 71 µmol, 1.0 equiv.) was dissolved in MeCN (2 mL), then **S20a-NHS** [4] (21 mg, 78 µmol, 1.1 equiv.) and Et_3N (79 µL, 0.57 mmol, 8.0 equiv.) were added. The reaction mixture was stirred at room temperature for 1 hour. After the complete conversion of the starting materials was confirmed with LC-MS, the solvent was removed under reduced pressure. The crude material was purified by HPLC (eluent: 0.1% HCOOH in H_2O/MeCN, gradient from 5% to 100% MeCN). The purified product was obtained from the aqueous solution by extraction with CH_2Cl_2 (2 × 25 mL), and the organic layer was washed with concentrated $NaHCO_3$ solution (30 mL), then the solvent was evaporated to yield product **S35**.

Yield: 40 mg (51% for the 2 steps)

^1H NMR (500 MHz, $CDCl_3$) δ 8.26 – 8.09 (m, 1H), 7.92 – 7.38 (m, 3H), 7.37 – 6.93 (m, 2H), 6.93 – 6.76 (m, 2H), 6.38 – 6.03 (m, 2H), 5.84 – 5.69 (m, 2H, contained in this multiplet: 5.73 (d, *J* = 16.3 Hz, 1H)), 5.60 – 5.47 (m, 1H), 5.41 – 5.34 (m, 1H), 5.30 (d, J = 15.7 Hz, 1H), 5.26 – 5.17 (m, 2H), 5.05 – 4.89 (m, 1H), 4.27 – 4.13 (m, 1H), 3.93 – 3.43 (m, 8H), 3.30 – 2.92 (m, 18H), 2.49 – 2.29 (m, 1H), 2.14 – 1.74 (m, 12H), 1.52 – 1.42 (m, 1H), 1.40 – 1.32 (m, 3H), 1.12 – 1.06 (m, 1H), 1.03 (t, J = 7.3 Hz, 3H), 0.96 – 0.85 (m, 3H), 0.84 – 0.73 (m, 1H).

HRMS: $[M+H]^+$: calcd for $[C_{61}H_{72}N_7O_{13}]^+$: 1110.5182, found: 1110.5177.

(E)*-Cyclooct-2-en-1-yl (9-(1-(((2-(((((S)-4,11-diethyl-4-hydroxy-3,14-dioxo-3,4,12,14-tetrahydro-1H-pyrano[3',4':6,7]indolizino[1,2-b]quinolin-9-yl)oxy)carbonyl)(methyl)amino)ethyl)(methyl)carbamoyl)oxy)ethyl)-9-hydroxy-6-(pyrrolidin-1-yl)-9H-xanthen-3-yl) ethane-1,2-diylbis(methylcarbamate) (rTCO-1-SN38)

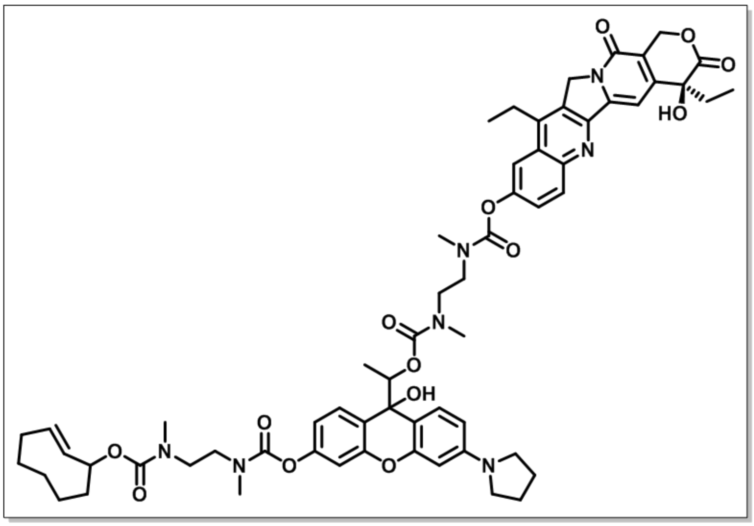

Compound **S35** (40 mg, 36 µmol, 1.0 equiv.) was dissolved in CH_2Cl_2/MeOH 1:1 mixture (1 mL), then *p*-chloranil (13 mg, 54 µmol, 1.5 equiv.) was added, and the reaction mixture was stirred at room temperature for 2 hours. After confirming the completion of the reaction with LC-MS, the solvents were removed under reduced pressure, and the crude material was purified by HPLC (eluent: 0.1% HCOOH in H_2O/MeCN, gradient from 5% to 100% MeCN). The purified product was extracted from the aqueous solution by extraction with CH_2Cl_2 (2 × 25 mL), and the organic layer was washed with concentrated $NaHCO_3$ solution (30 mL), then the solvent was evaporated to yield a red solid.

Yield: 11 mg (26%)

^{1}H NMR (500 MHz, $CDCl_3$) δ 8.21 – 8.06 (m, 1H), 7.90 – 7.77 (m, 1H), 7.74 – 7.65 (m, 1H), 7.64 – 7.44 (m, 3H), 6.96 – 6.84 (m, 2H), 6.44 – 5.98 (m, 2H), 5.83 – 5.74 (m, 1H), 5.71 (d, J = 16.3 Hz, 1H), 5.60 – 5.48 (m, 1H), 5.41 – 5.10 (m, 5H), 3.88 – 3.40 (m, 8H), 3.33 – 2.88 (m, 18H), 2.48 – 2.32 (m, 1H), 2.12 – 1.79 (m, 10H), 1.70 – 1.61 (m, 2H), 1.41 – 1.29 (m, 5H), 1.13 – 1.06 (m, 1H), 1.03 (t, *J* = 7.1 Hz, 3H), 0.90 – 0.76 (m, 4H).

HRMS: $[M-H_2O+2H]^{2+}$: calcd for $[C_{61}H_{71}N_7O_{13}]^{2+}$: 554.7549, found: 554.7547.

(S)*-4,11-Diethyl-4-hydroxy-3,14-dioxo-3,4,12,14-tetrahydro-1H-pyrano[3',4':6,7]indolizino[1,2-b]quinolin-9-yl (1-(3-oxo-6-(pyrrolidin-1-yl)-3H-xanthen-9-yl)ethyl) ethane-1,2-diylbis(methylcarbamate) (1-SN38)

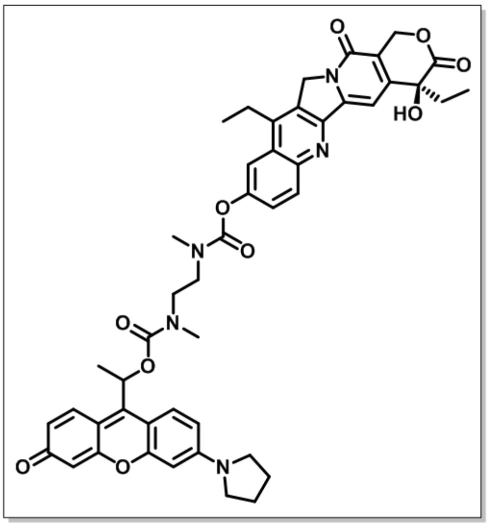

Deprotection (step 1): Compound **S33** (20 mg, 23 µmol, 1.0 equiv.) was treated with TFA (375 µL) in CH_2Cl_2 (1.5 mL). The reaction mixture was stirred at room temperature for 30 min. The volatiles were removed under reduced pressure, and the crude product was used in the subsequent step without further purification.

Oxidation (step 2): Deprotected **S33** (calculated as trifluoroacetate salt: 22 mg, 23 µmol, 1.0 equiv.) was dissolved in CH_2Cl_2/MeOH 1:1 solvent mixture (1 mL), then *p*-chloranil (6.8 mg, 28 µmol, 1.2 equiv.) was added and the reaction mixture was stirred at room temperature for 1 hour. Upon verification of full conversion by LC-MS, the solvents were removed under reduced pressure, and the crude material was purified by flash chromatography on silica (eluent: CH_2Cl_2/MeOH 0% to 15%) to yield a red solid.

Yield: 9.2 mg (48%)

^{1}H NMR (500 MHz, $CDCl_3$) δ 8.24 – 7.70 (m, 4H), 7.66 – 7.60 (m, 1H), 7.59 – 7.42 (m, 1H), 6.73 – 6.51 (m, 3H), 6.47 – 5.94 (m, 2H), 5.75 (d, *J* = 16.2 Hz, 1H), 5.34 – 5.19 (m, 3H), 4.18 – 3.51 (m, 4H), 3.49 – 3.23 (m, 4H), 3.20 – 2.98 (m, 8H), 2.13 – 1.99 (m, 4H), 1.96 – 1.87 (m, 2H), 1.85 – 1.71 (m, 3H), 1.42 – 1.30 (m, 3H), 1.04 (t, *J* = 6.9 Hz, 3H).

HRMS: $[M+H]^+$: calcd for $[C_{47}H_{48}N_5O_{10}]^+$: 842.3395, found: 842.3404.

3. Spectroscopic Properties of the Compounds

3.1 Molar Absorption Coefficients

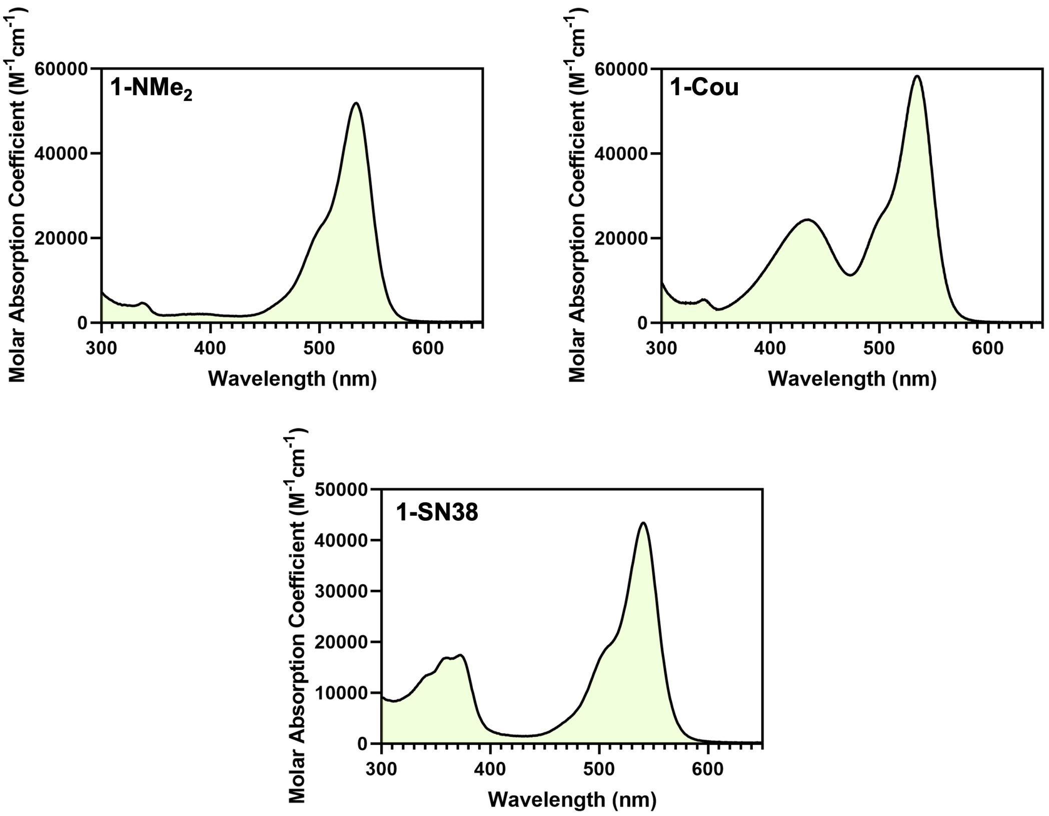

*Figure S1. Absorption spectra of compounds derived from photocage **1** measured in HEPES (pH 7.4, containing 25% MeCN)*

3.2 Absorption and Emission Spectra of the Various Forms

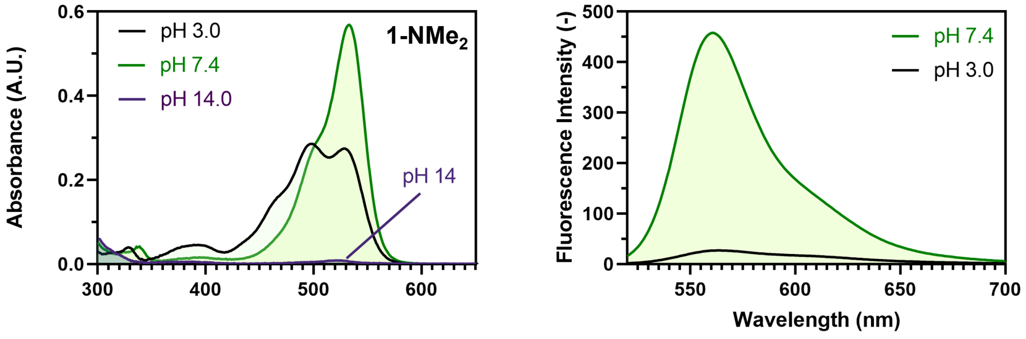

*Figure S2. Absorption and emission spectra of compound **1-NMe₂** (10 μM) measured in HEPES (pH 7.4, containing 1% MeCN, oxo form) and 0.1% HCOOH in water (pH 3.0, containing 1% MeCN, iminium form). Excitation wavelength: 506 nm*

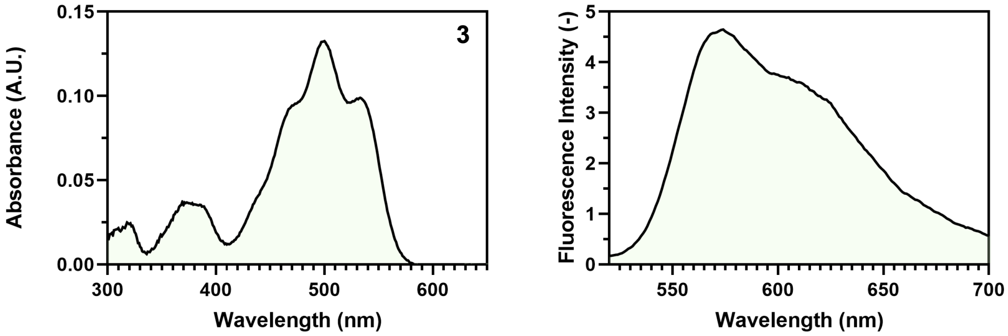

*Figure S3. Absorption and emission spectra of compound **3** measured in 0.1% HCOOH in water (pH 3.0, containing 1% MeCN, iminium form). Excitation wavelength: 506 nm.*

3.3 pH-Dependent Absorption Spectra

The absorption spectra of **1-NMe₂** and substituted rhodol compounds **2**, **3**, **4**, and **5** were measured across a pH range of 2.0 to 10.0 in one-unit increments. Samples were prepared by dissolving each compound at a final concentration of 10 μM in Britton–Robinson buffer solutions containing 10% MeCN. Following preparation, solutions were incubated at room temperature in the dark to allow for complete pH-dependent interconversion. UV/Vis absorbance spectra were subsequently recorded over the 300–700 nm wavelength range. The pK_{oxo} and pK_{leuco} values were determined by plotting the absorbance values at the respective absorption maxima of each compound as a function of pH. The resulting data were fitted using a sigmoidal model in Prism 8.0 (4PL, X is log(concentration)) to extract the p*K* values corresponding to the oxo and leuco forms.

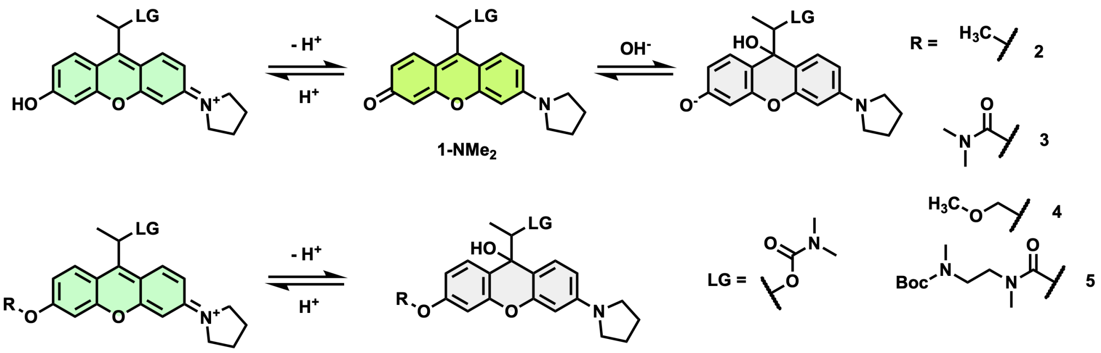

*Scheme S6. Structure and various forms of compound **1**-**5***

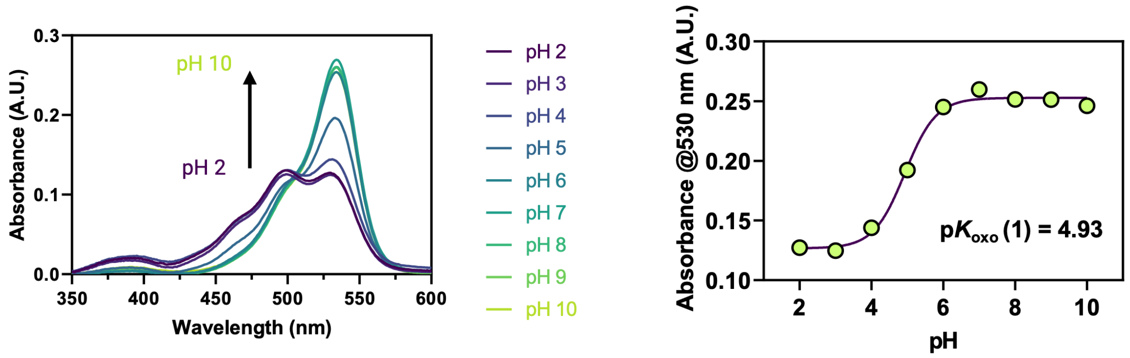

*Figure S4. pH-dependent absorption spectra of **1-NMe₂** and the absorbance values at 530 nm*

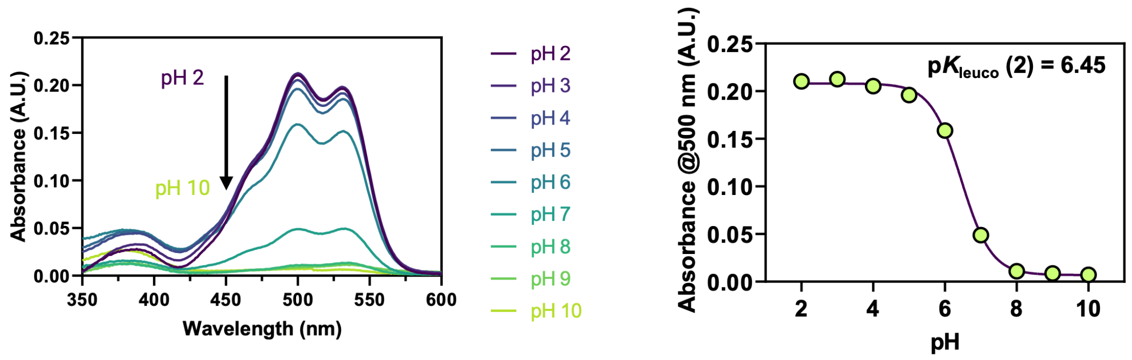

*Figure S5. pH-dependent absorption spectra of **2** and the absorbance values at 530 nm*

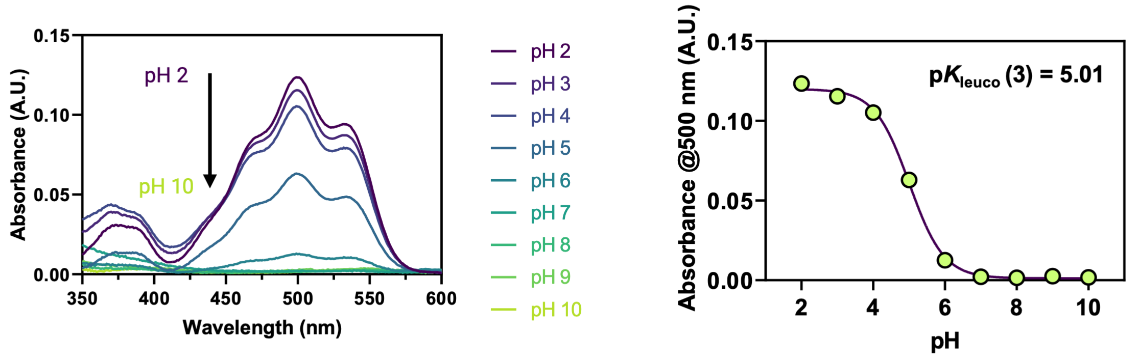

*Figure S6. pH-dependent absorption spectra of **3** and the absorbance values at 530 nm*

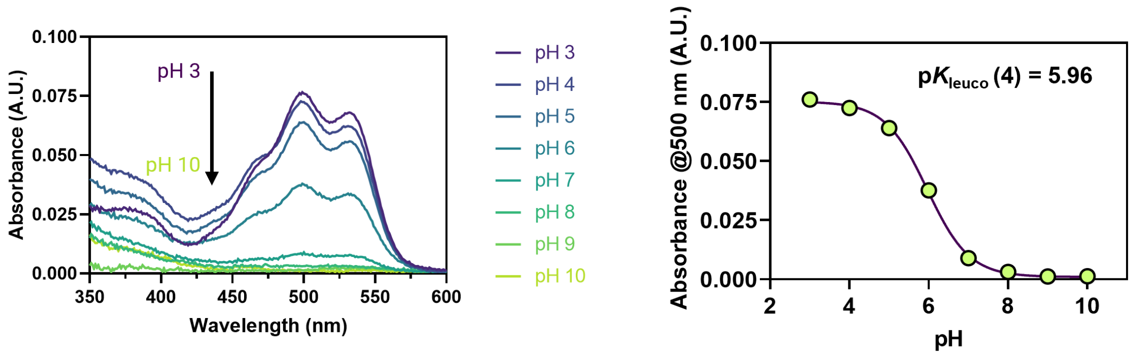

*Figure S7. pH-dependent absorption spectra of **4** and the absorbance values at 530 nm*

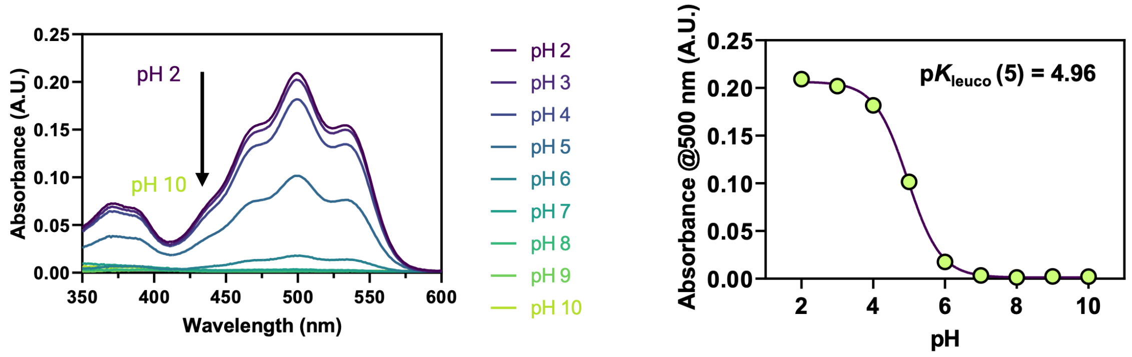

*Figure S8. pH-dependent absorption spectra of **5** and the absorbance values at 530 nm*

4. Uncaging Studies

4.1 Experimental Details

4.1.1 Irradiation Studies

The uncaging experiments were performed using a custom-made LED panel with commercial light sources. The specifications were as follows: input power: 4 W; λ_{max} = 549 nm; half-width: 16 nm; output power: 72 mW.

In the ^{1}H NMR experiments, a solution of **1-NMe$_2$** (approx. 1 mM) was prepared in D_2O/CD_3CN 3:1 solvent mixture (V_{total} = 500 µL). The sample was subjected to green light irradiation for defined durations (0 min, 30 min, 60 min). ^{1}H NMR spectra were recorded at each time point to monitor the uncaging reaction.

In the HPLC-MS experiments, sample solutions used for the irradiation studies contained 50 µM of the target compound in a 9:1 mixture of HEPES buffer and MeCN (V_{total} = 1 mL). The HEPES buffer solution (10 mM, pH 7.4) was freshly prepared by dilution from a 1 M stock solution. Samples were irradiated for defined durations, with continuous water cooling applied to the light source to prevent overheating. After irradiation, the samples were analyzed by an HPLC-UV/Vis-MS system. The chromatograms at 254 nm and at the absorption maxima of the photoremovable protecting group or its payload were compared. The possible photoproducts were identified by their corresponding *m*/*z* values and absorption profiles. Further irradiation experiments were conducted under acidic conditions using compounds **1-NMe$_2$** and **3**. Sample solutions (50 µM) of each compound were prepared in an aqueous HCOOH solution (pH 3.0) containing 10% MeCN (V_{total} = 1000 µL). The samples were subjected to green light irradiation for defined durations, and LC-MS analyses were carried out at each time point.

4.1.2 Click-to-Release Experiments

Click-to-release reactions were monitored by LC-MS analyses. The bioorthogonally activatable photocages **rTCO-1-Cou** and **rTCO-1-SN38** were each reacted with **Tz** in a 3:1 mixture of HEPES buffer and MeCN. Reaction mixtures (1 mL) contained the bioorthogonally activatable photocage (20 µM) and **Tz** (100 µM, 5 equiv.). The species of the cascade reaction were identified by their corresponding *m*/*z* values. After the click-to-release reaction, the activated **1-SN38** photocage was irradiated with green light for defined durations, and the photolysis was monitored by LC-MS. The bioorthogonal activation of **rTCO-1-Cou** was further monitored by UV/Vis spectroscopy. The reaction was carried out in a 3:1 mixture of HEPES buffer and MeCN (V_{total} = 2500 µL), containing **rTCO-1-Cou** (20 µM) and **Tz** (100 µM, 5 equiv.). The mixture was incubated at 37 °C, and the formation of the activated rhodol chromophore was detected by recording UV/Vis spectra over time. Similarly, the bioorthogonal activation of rTz-1-Cou was also monitored by UV/Vis spectroscopy. The reaction was carried out in a 3:1 mixture of HEPES buffer and MeCN (V_{total} = 2500 µL), containing rTz-1-Cou (20 µM) and TCO-halo (100 µM, 5 equiv., see the structure in Figure S34). The mixture was incubated at 37 °C, and the formation of the activated rhodol chromophore was detected by recording UV/Vis spectra over time.

4.1.3 Leuco-to-Oxo Form Conversion Monitoring

The leuco-to-oxo form conversion of **1-NMe$_2$** was monitored using UV/Vis spectroscopy. A basic stock solution of **1-NMe$_2$** (0.5 mM in water/MeOH 1:1 solvent mixture, containing 0.5 M NaOH) was prepared, yielding a colorless solution. This stock solution was subsequently diluted with HEPES buffer to a final concentration of 5 µM, and the pH was adjusted to 7.0 using 2 M HCl. The interconversion process was monitored over a 5 h period by UV/Vis spectroscopy.

4.2 NMR Studies

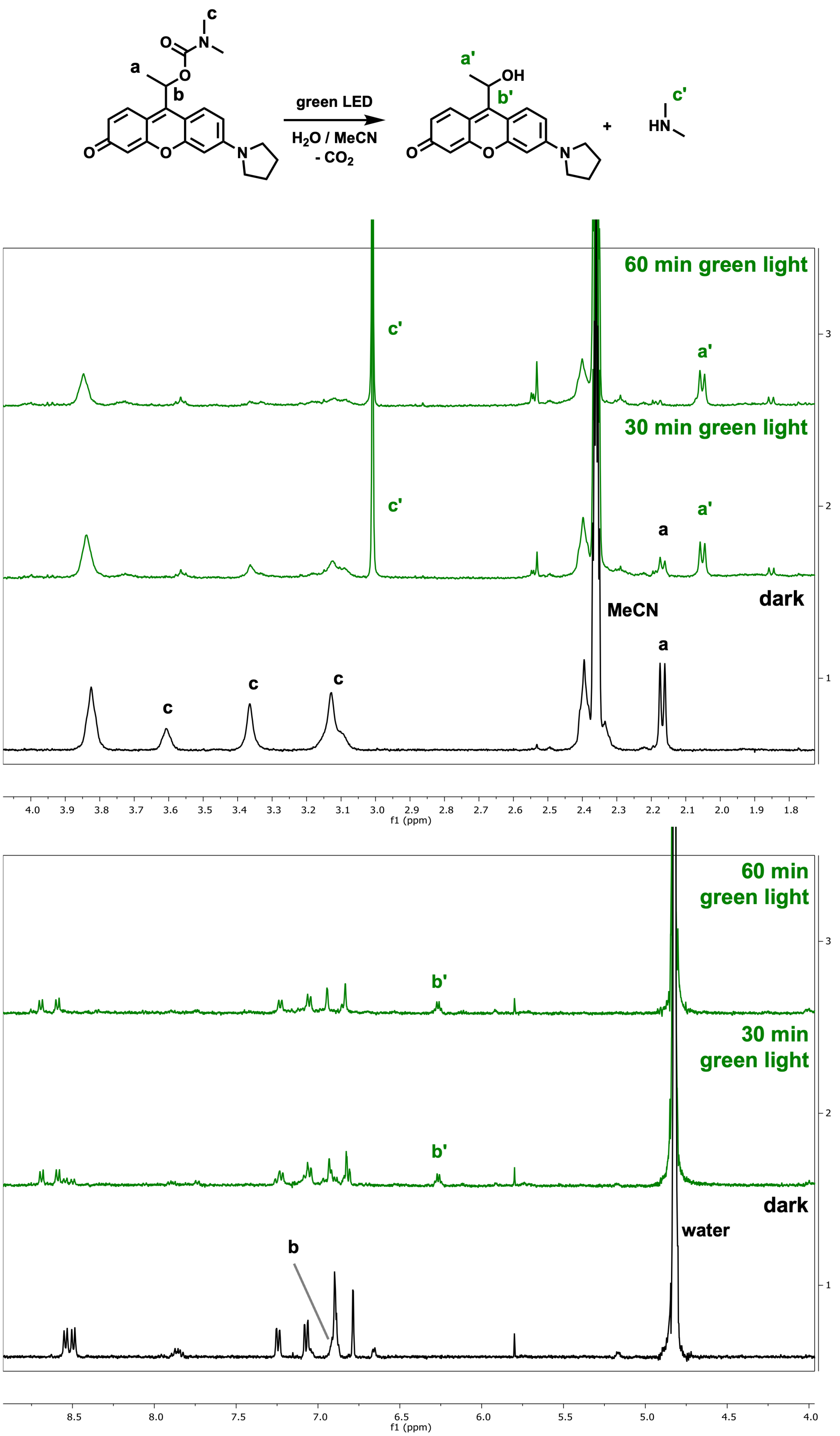

Figure S9. [1]*H-NMR studies of the uncaging of* ***1-NMe₂*** *(~1 mM in D_2O - CD_3CN 3:1)*

4.3 HPLC Chromatograms of the Irradiation Experiments

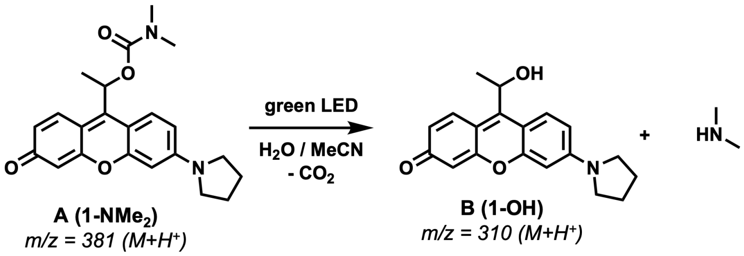

A (1-NMe$_2$) *m/z = 381 (M+H$^+$)*

B (1-OH) *m/z = 310 (M+H$^+$)*

irradiation in HEPES/MeCN 9:1 (chromatograms @530 nm):

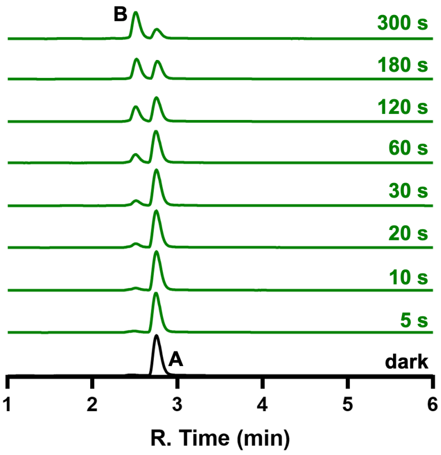

irradiation in HEPES/MeCN 9:1 (2D contour chromatograms):

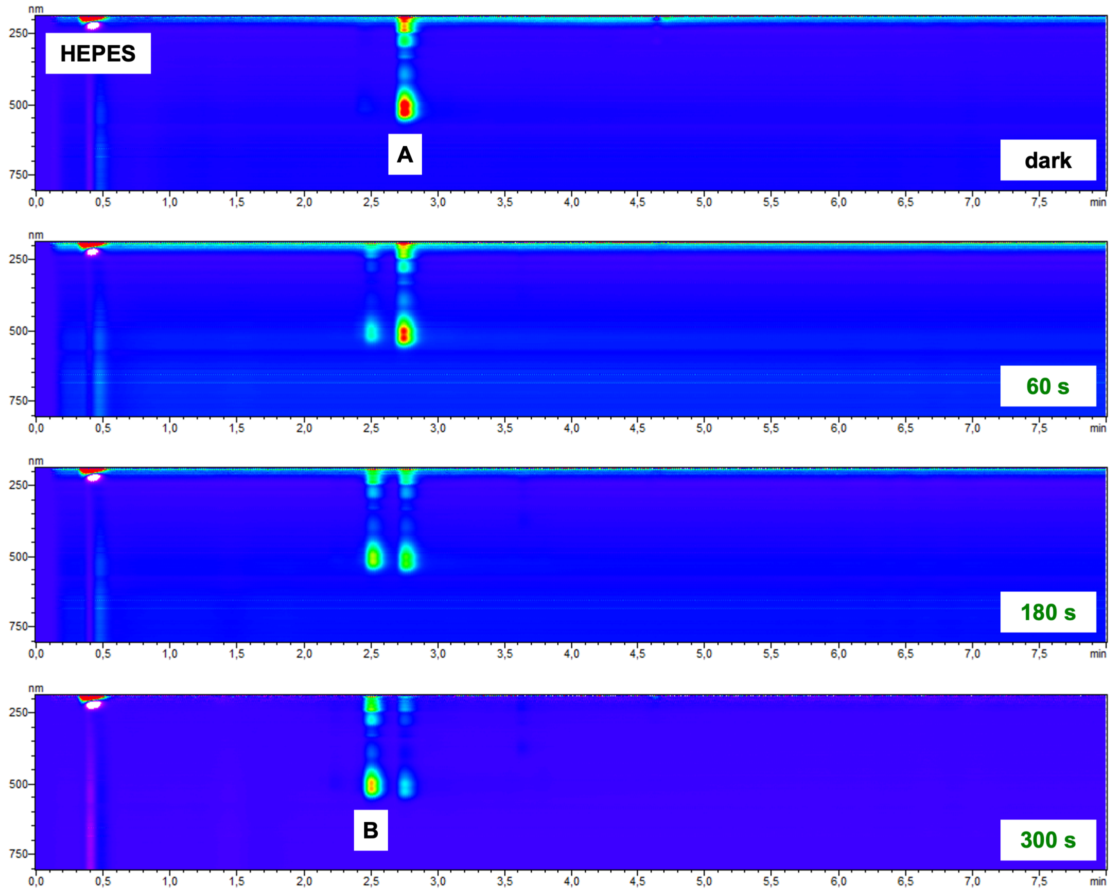

*Figure S10. Chromatograms of the photolysis of **1-NMe$_2$** as followed by HPLC-UV/Vis-MS instrument. The disappearance of the starting material (A) and the appearance of 1-OH (B) was monitored at 530 nm.*

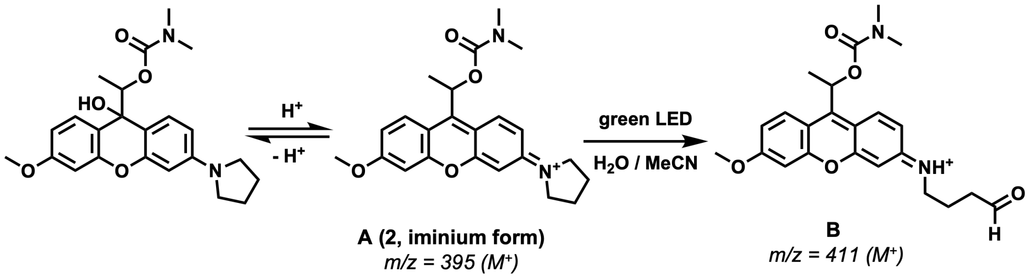

irradiation in HEPES/MeCN 9:1 (chromatograms @530 nm):

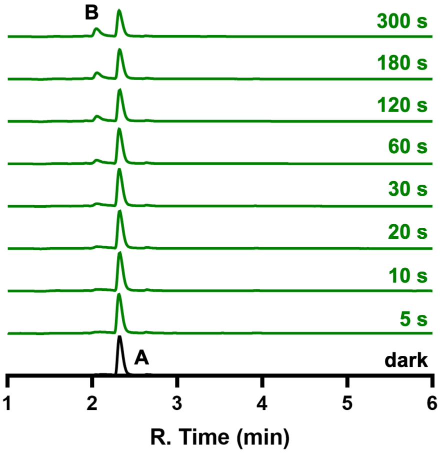

irradiation in HEPES/MeCN 9:1 (2D contour chromatograms):

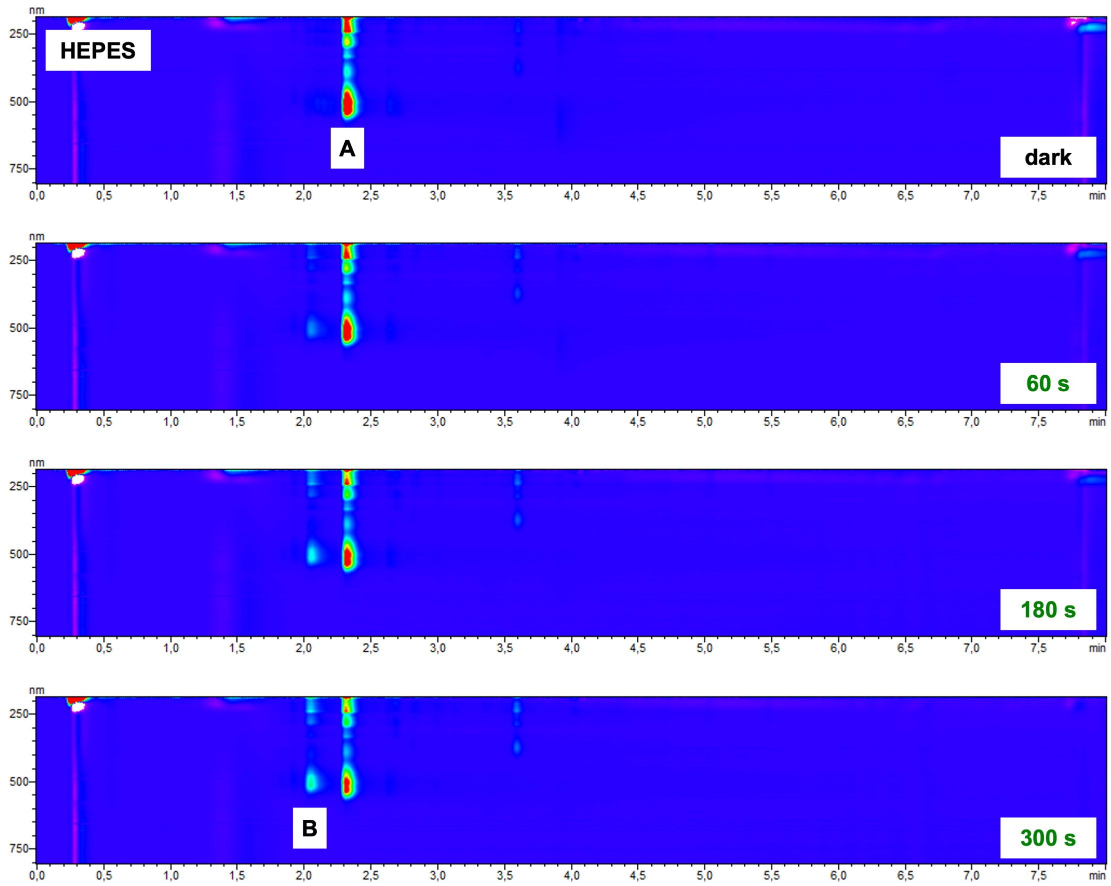

Figure S11. Chromatograms of the green light irradiation studies of ***2*** *as followed by HPLC-UV/Vis-MS instrument. The appearance of photooxidation product B (suggested structure) and the disappearance of the starting material A was monitored at 530 nm.*

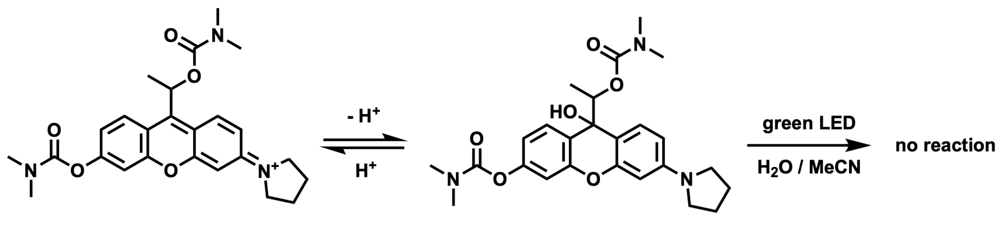

A (3, iminium form)
m/z = 452 (M+)

irradiation in HEPES/MeCN 9:1 (chromatograms @500 nm):

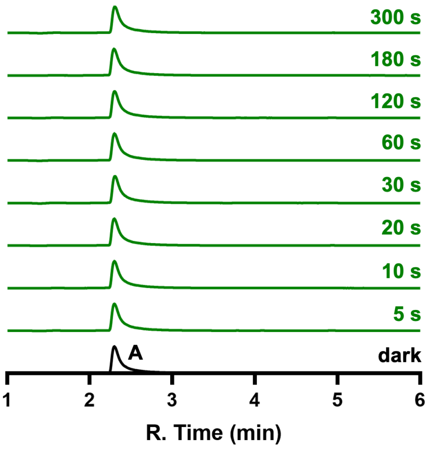

irradiation in HEPES/MeCN 9:1 (2D contour chromatograms):

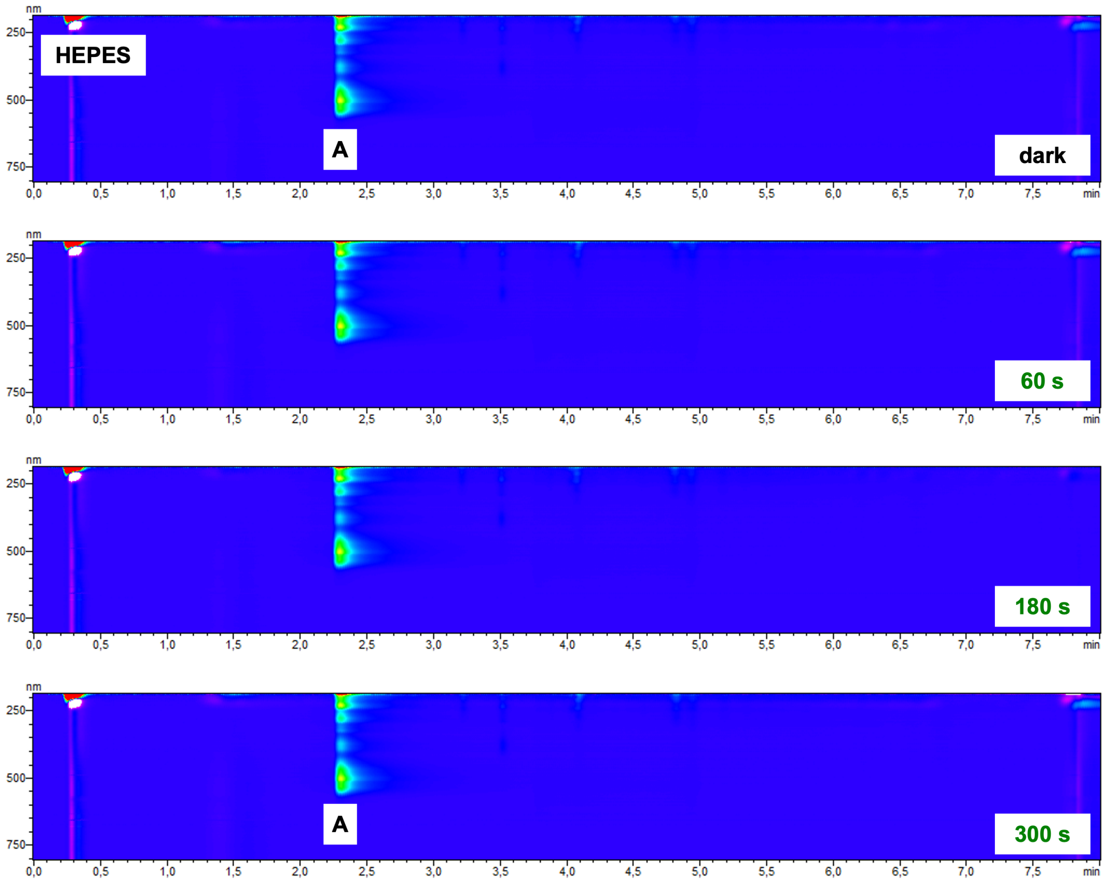

*Figure S12. Chromatograms of the green light irradiation studies of **3** as followed by HPLC-UV/Vis-MS instrument. No photoreactivity was observed.*

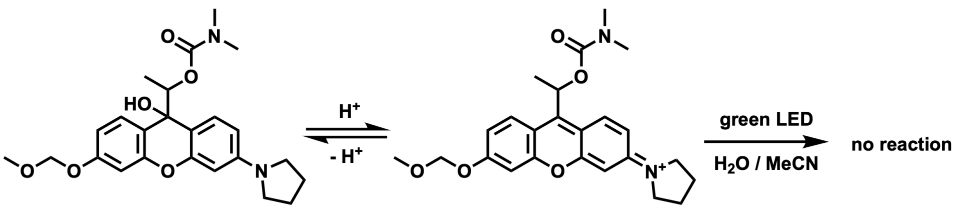

A (4, iminium form)
m/z = 425 (M+)

irradiation in HEPES/MeCN 9:1 (chromatograms @500 nm):

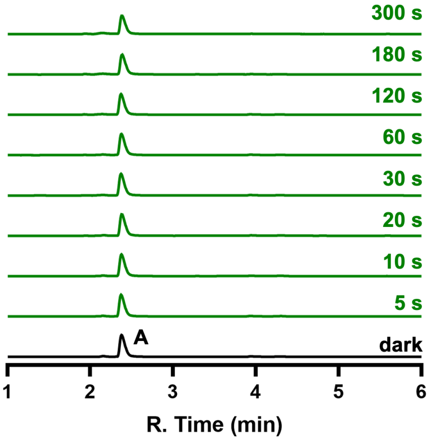

irradiation in HEPES/MeCN 9:1 (2D contour chromatograms):

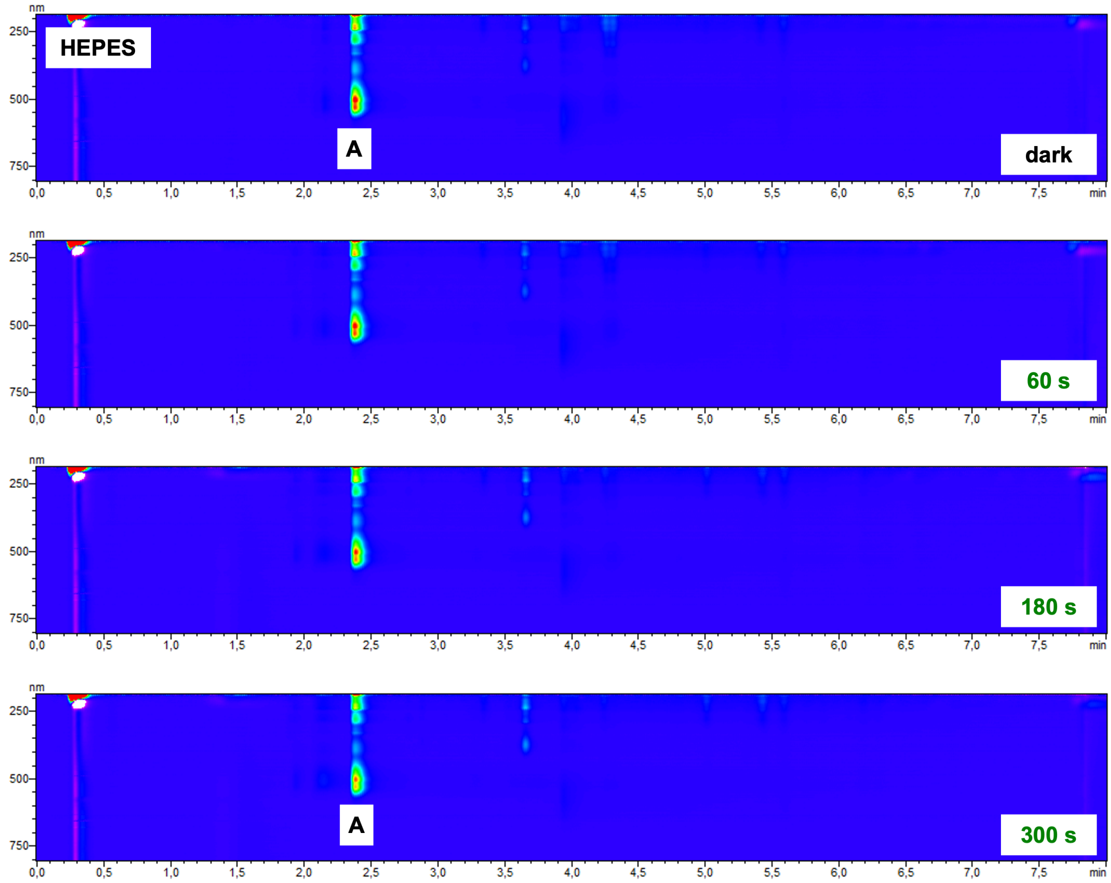

*Figure S13 Chromatograms of the green light irradiation studies of **4** as followed by HPLC-UV/Vis-MS instrument. No photoreactivity was observed.*

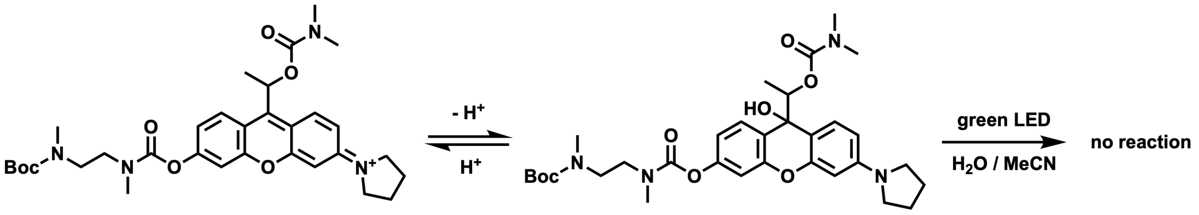

A (5, iminium form)
m/z = 595 (M+)

irradiation in HEPES/MeCN 9:1 (chromatograms @500 nm):

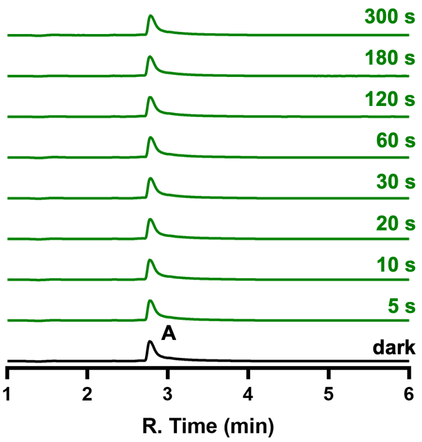

irradiation in HEPES/MeCN 9:1 (2D contour chromatograms):

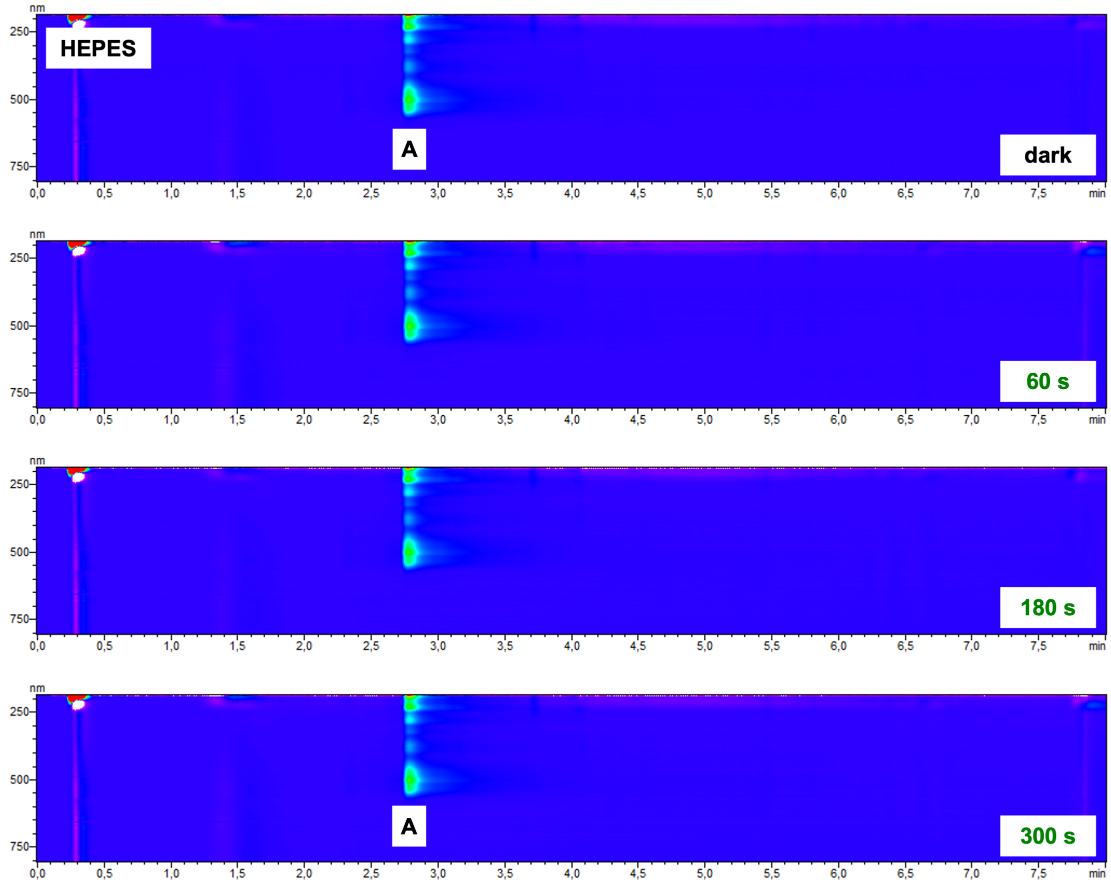

*Figure S14. Chromatograms of the green light irradiation studies of **5** as followed by HPLC-UV/Vis-MS instrument. No photoreactivity was observed*

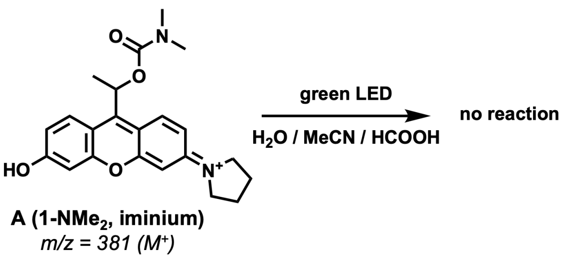

irradiation in aq. HCOOH/MeCN 9:1 (chromatograms @530 nm):

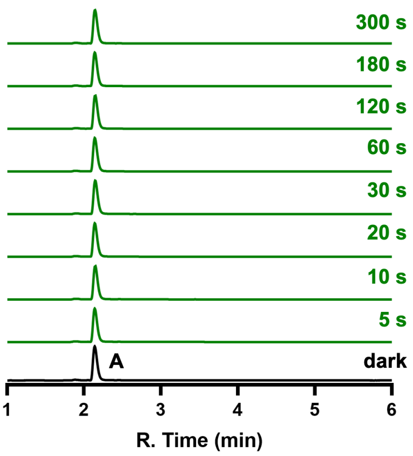

irradiation in aq. HCOOH/MeCN 9:1 (2D contour chromatograms):

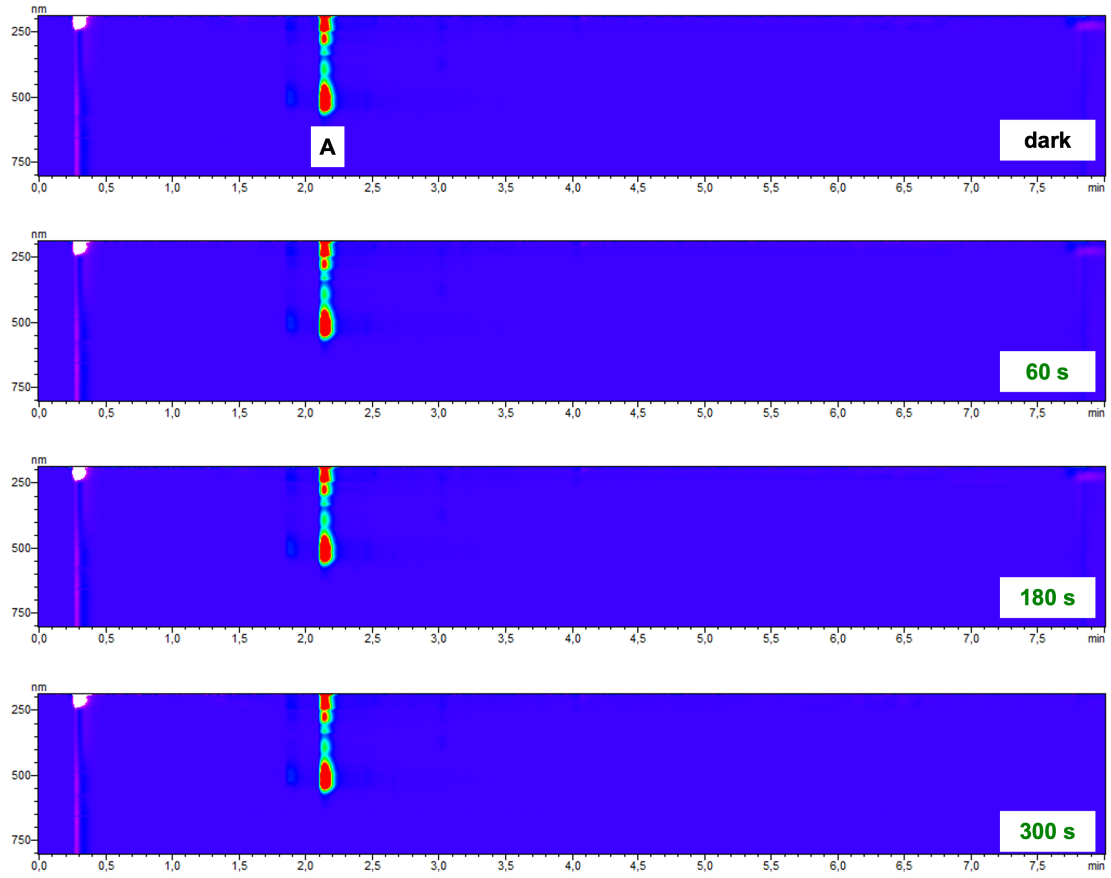

*Figure S15. Chromatograms of the green light irradiation studies of **1-NMe₂** in acidic medium as followed by HPLC-UV/Vis-MS instrument. No photoreactivity was observed*

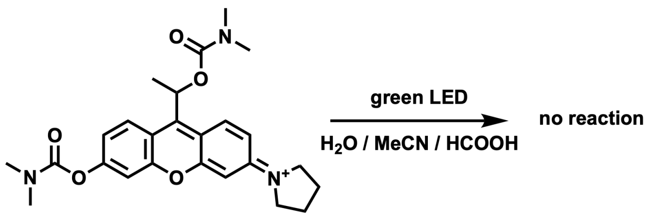

A (3, iminium form)
m/z = 452 (M$^+$*)*

irradiation in aq. HCOOH/MeCN 9:1 (chromatograms @500 nm):

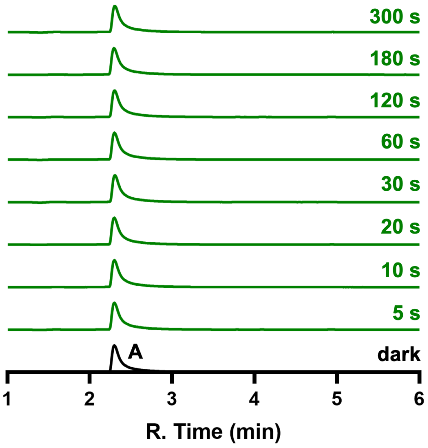

irradiation in aq. HCOOH/MeCN 9:1 (2D contour chromatograms):

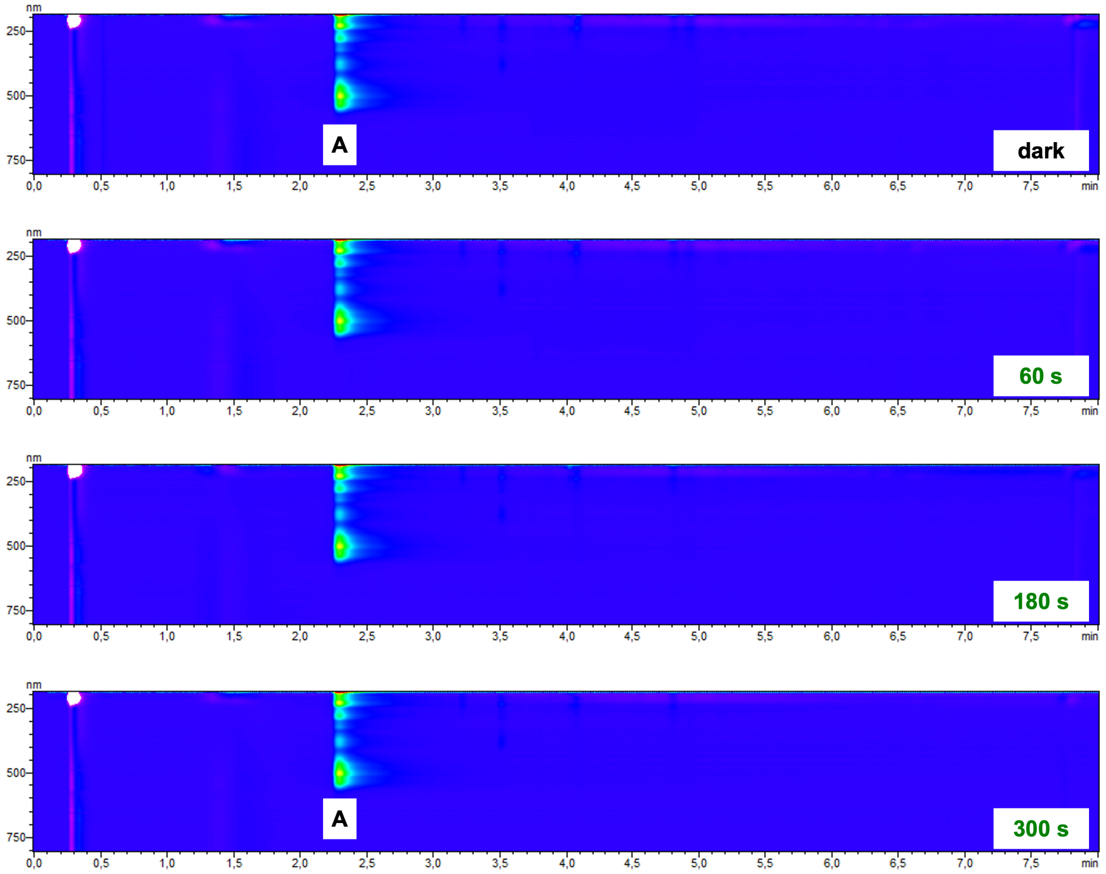

Figure S16. Chromatograms of the green light irradiation studies of ***3*** *in acidic medium as followed by HPLC-UV/Vis-MS instrument. No photoreactivity was observed*

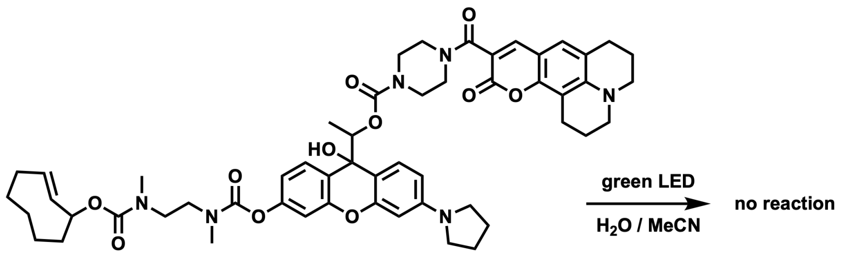

A (rTCO-1-Cou)
m/z = 955 (iminium, (M-OH)$^+$)

irradiation in HEPES/MeCN 9:1:

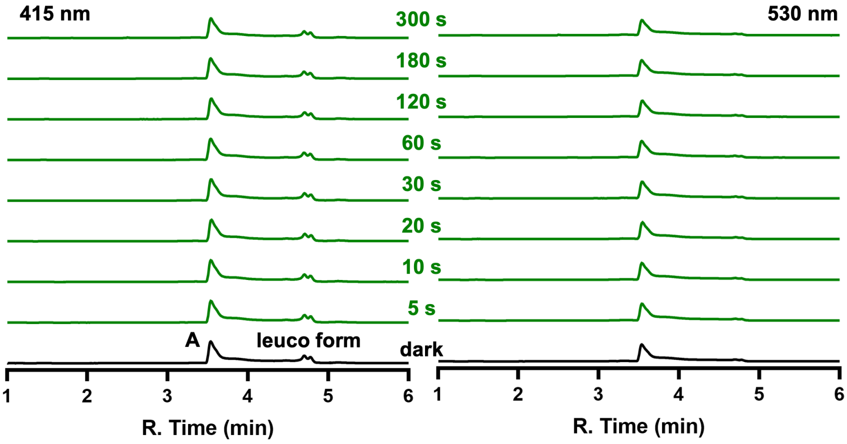

irradiation in HEPES/MeCN 9:1 (2D contour chromatograms):

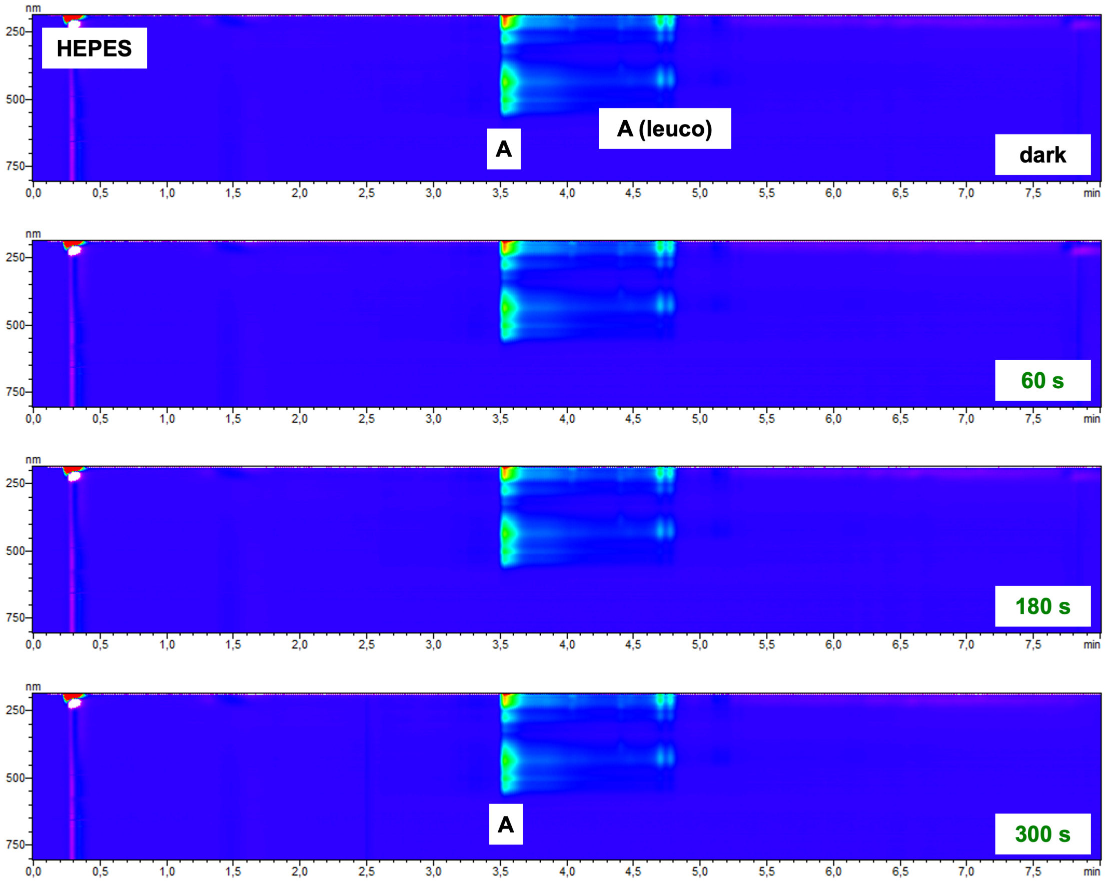

*Figure S17. Chromatograms of the green light irradiation studies of **rTCO-1-Cou** as followed by HPLC-UV/Vis-MS instrument. No photoreactivity was observed.*

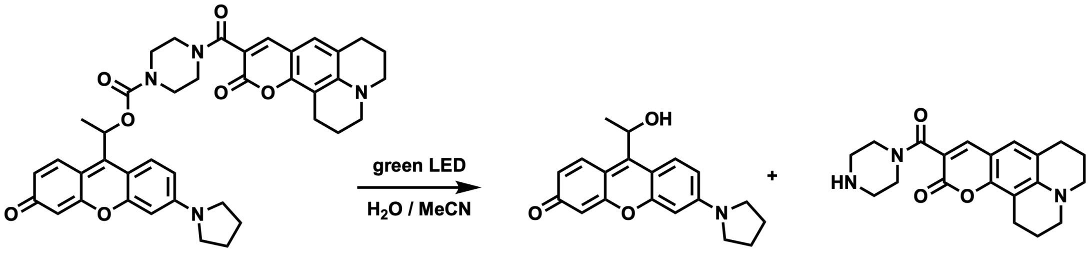

A (1-Cou)
m/z = 689 (M+H)⁺

B (1-OH)
m/z = 310 (M+H)⁺

C (Cou)
m/z = 354 (M+H)⁺

irradiation in HEPES/MeCN 9:1:

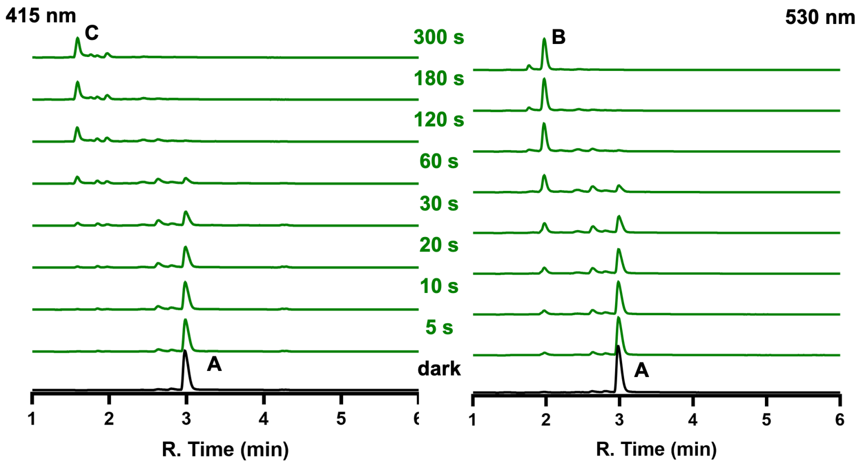

irradiation in HEPES/MeCN 9:1 (2D contour chromatograms):

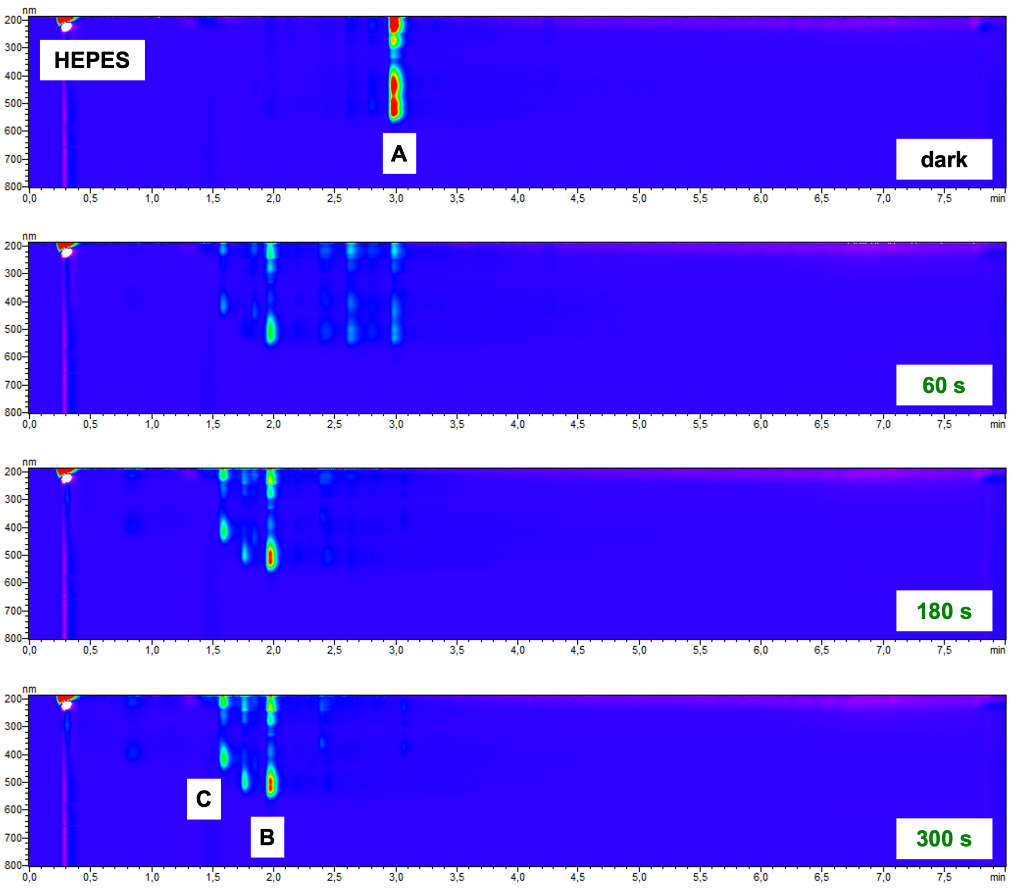

*Figure S18. Chromatograms of the photolysis of **1-Cou** as followed by HPLC-UV/Vis-MS instrument. The appearance of the **Cou** payload (C) and the decrease of **1-Cou** (A) was monitored at the absorption maxima of each compound (415 nm and 530 nm, respectively).*

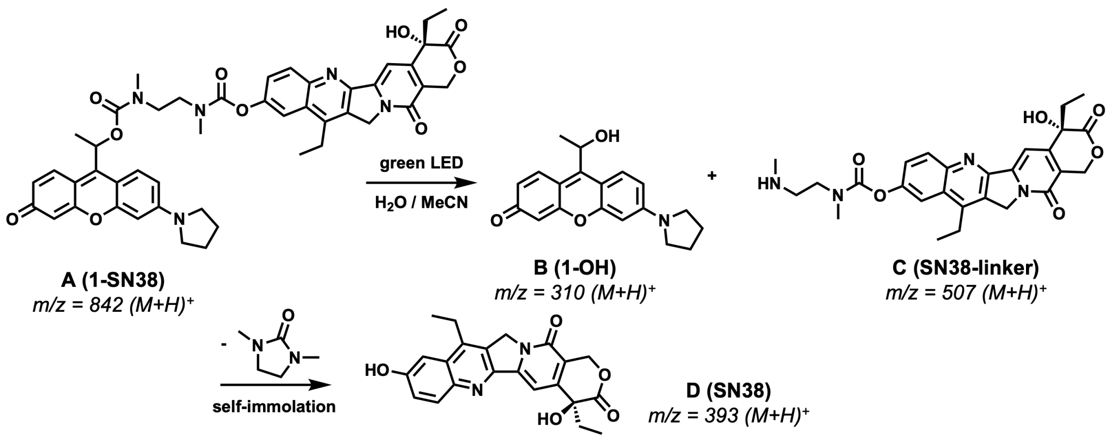

irradiation in HEPES/MeCN 9:1:

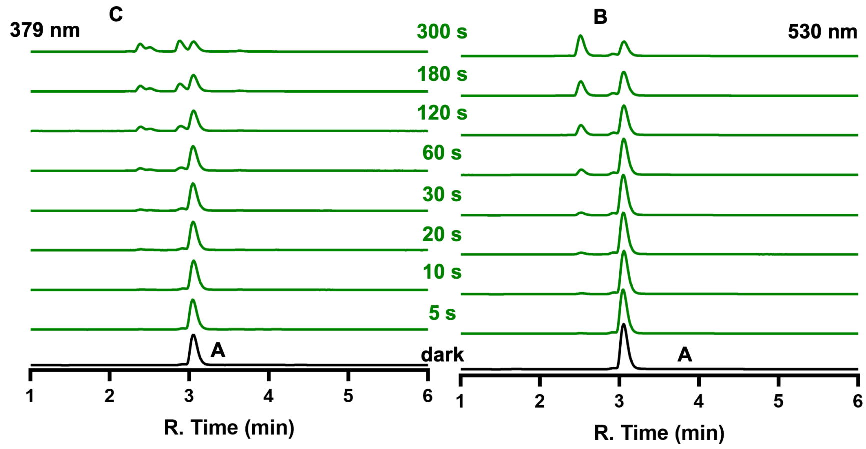

irradiation in HEPES/MeCN 9:1 (2D contour chromatograms):

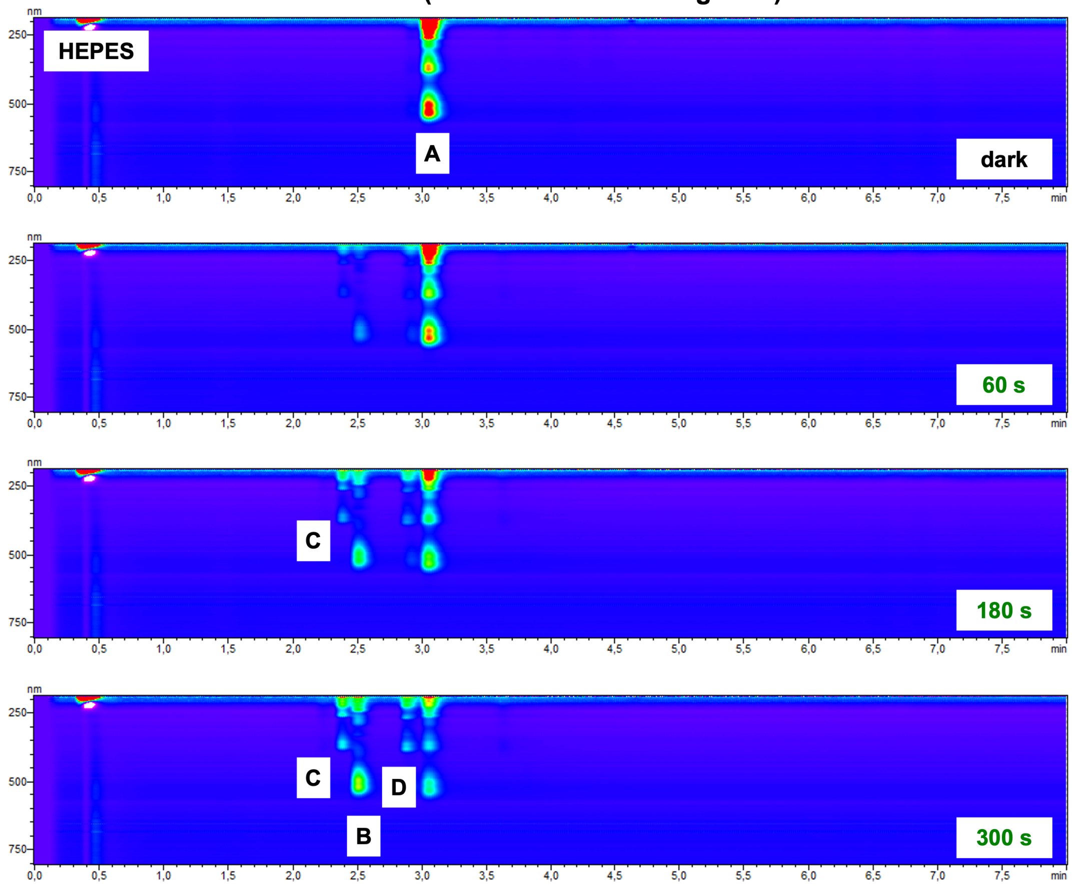

*Figure S19. Chromatograms of the photolysis of **1-SN38** as followed by HPLC-UV/Vis-MS instrument. The appearance of the SN38 compounds (C and D) and the decrease of **1-SN38** (A) was monitored at the absorption maxima of each compound (379 nm and 530 nm, respectively).*

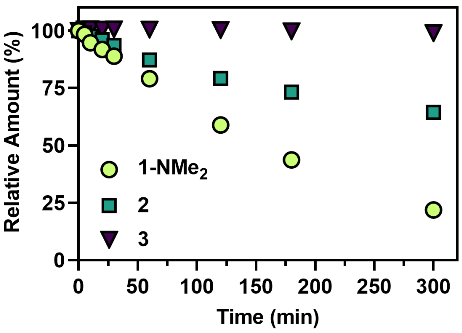

*Figure S20. Relative amounts of **1-NMe₂**, **2** and **3** upon green light irradiation based on the HPLC-UV/Vis integrals*

4.4 Click-to-Release Experiments of rTCO-Rhod

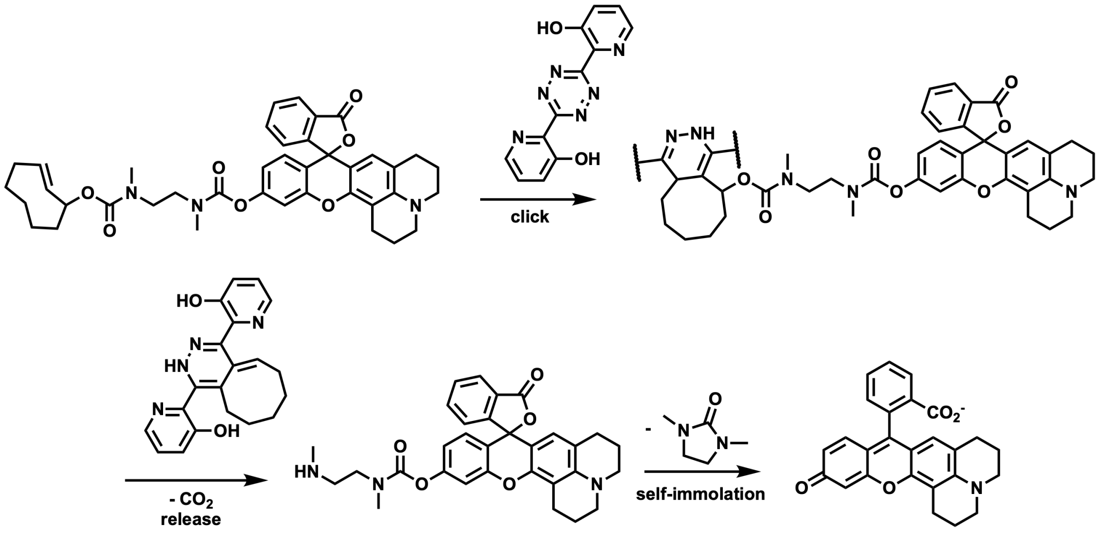

*Scheme S7 Click-to-release reaction cascade of **rTCO-Rho** with **Tz***

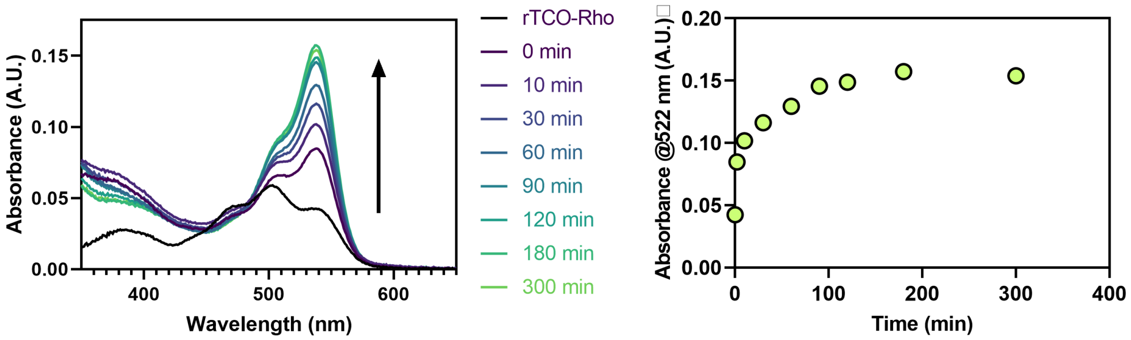

*Figure S21. Absorption spectra of the click-to-release reaction of **rTCO-Rho** (50 µM) with **Tz** (1.2 equiv.) in HEPES buffer (10 mM, pH 7.4, containing 25% MeCN). Absorption was monitored after dilution to 2.5 µM in PBS*

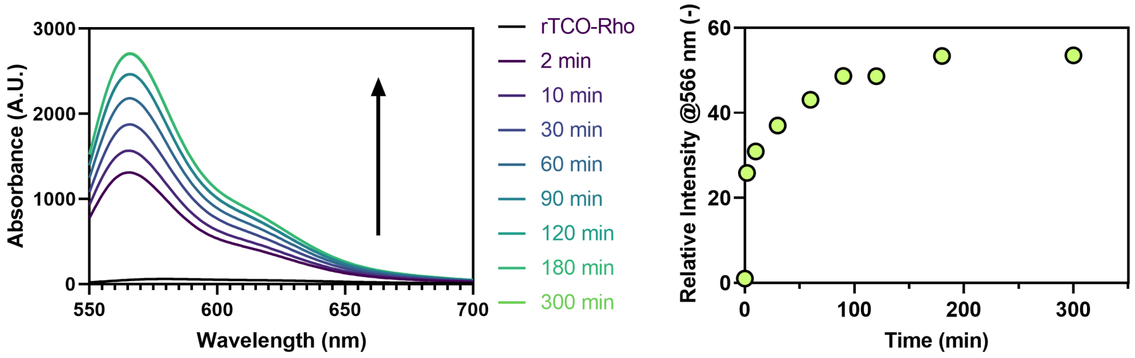

*Figure S22. Fluorescence spectra of the click-to-release reaction of **rTCO-Rho** (50 μM) with **Tz** (1.2 equiv.) in HEPES buffer (10 mM, pH 7.4, containing 25% MeCN). Fluorescence was monitored after dilution to 2.5 μM in PBS. Excitation wavelength: 530 nm.*

4.5 Dark Stability of the rTCO-Compounds

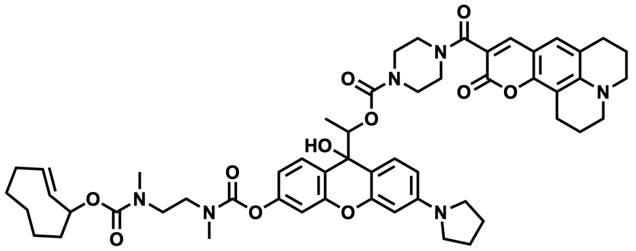

rTCO-1-Cou
m/z = 955 (iminium, (M-OH)⁺)

dark stability in HEPES/MeCN 3:1 (chromatograms @500 nm):

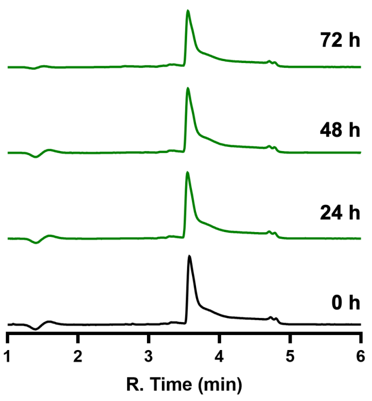

dark stability in HEPES/MeCN 3:1 (2D contour chromatograms):

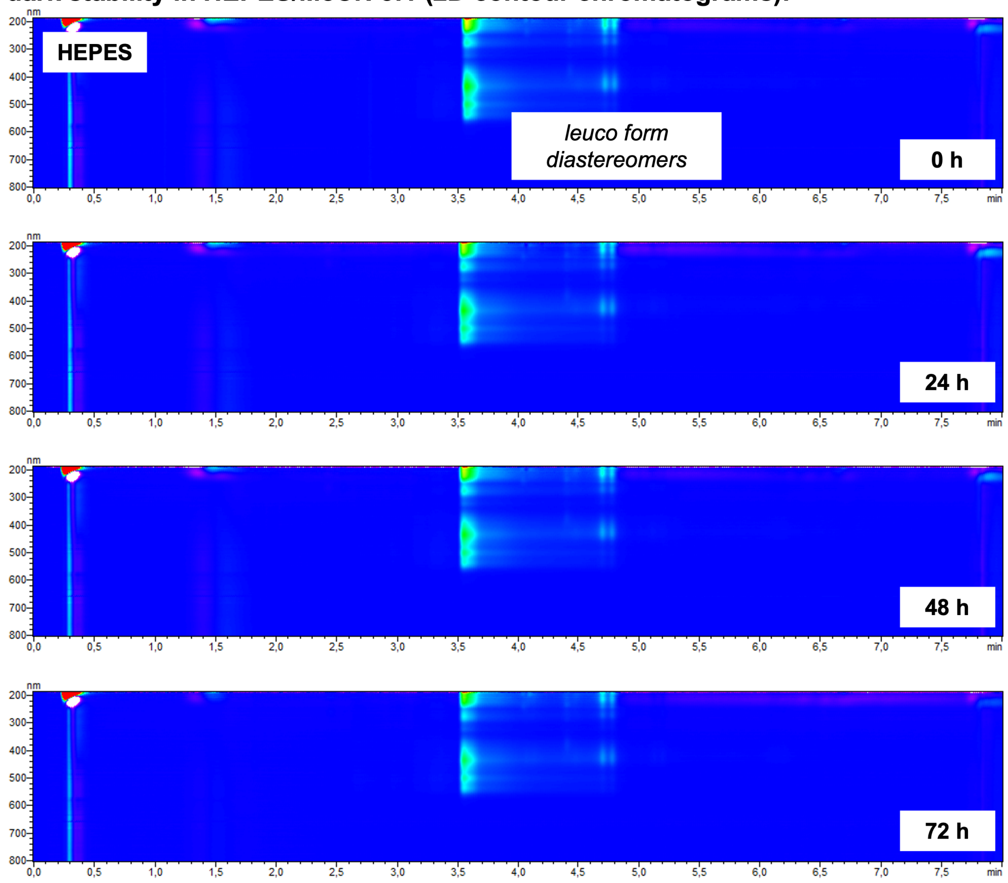

*Figure S23. Chromatograms of the dark stability studies of **rTCO-1-Cou** as followed by HPLC-UV/Vis-MS instrument. No degradation was observed.*

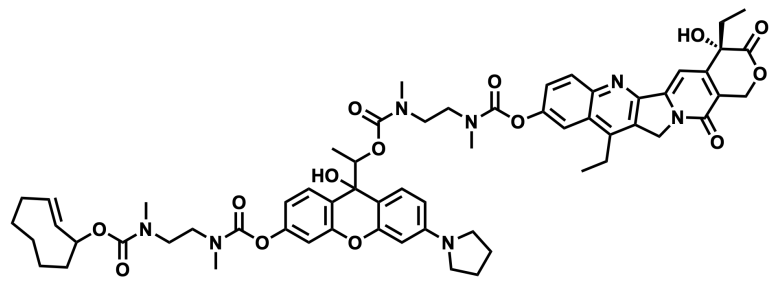

rTCO-1-SN38
m/z = 555 (iminium, (M-OH+H)$^{2+}$)

dark stability in HEPES/MeCN 3:1 (chromatograms @500 nm):

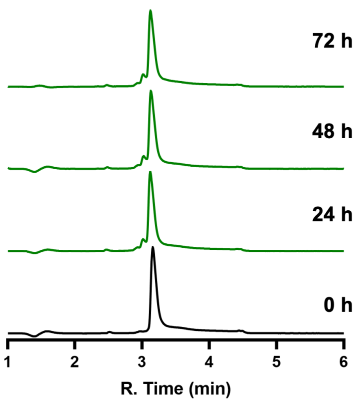

dark stability in HEPES/MeCN 3:1 (2D contour chromatograms):

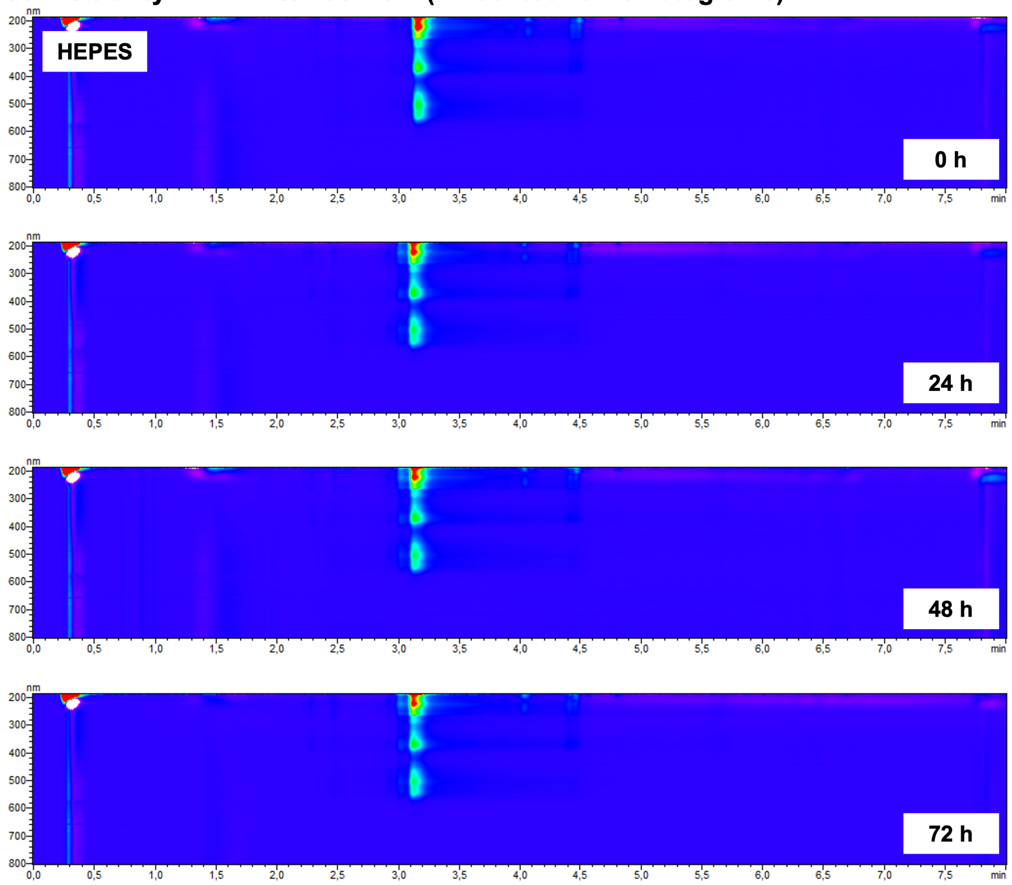

*Figure S24. Chromatograms of the dark stability studies of **rTCO-1-SN38** as followed by HPLC-UV/Vis-MS instrument. No degradation was observed.*

4.6 HPLC Chromatograms of the Click-to-Release Experiments

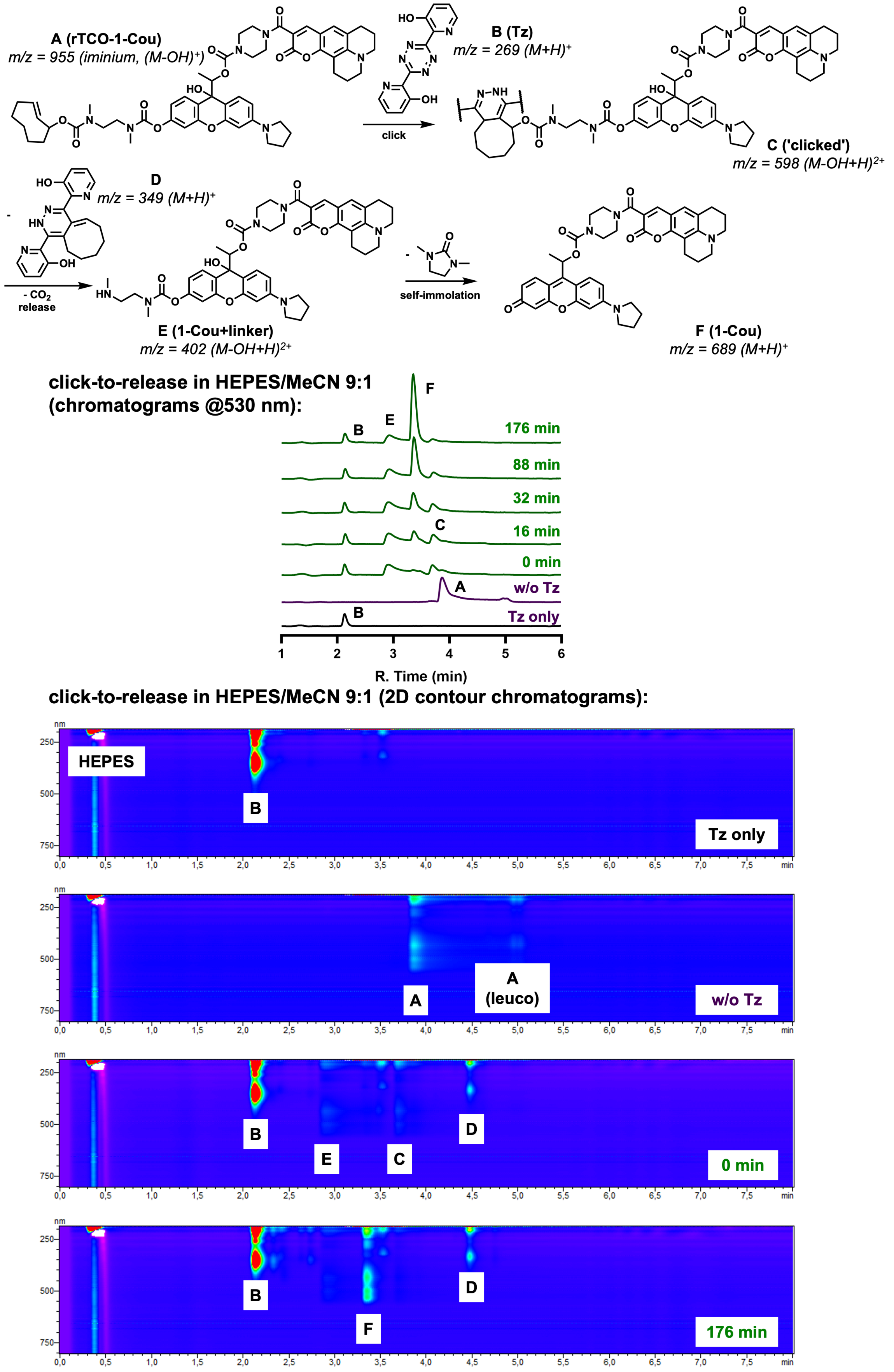

*Figure S25. Chromatograms of the click-to-release cascade reaction of **rTCO-1-Cou** (A) and **Tz** (B). The disappearance of the starting material (A) and the appearance of the intermediates (C and E) and the activated **1-Cou** (F) were monitored at 530 nm.*

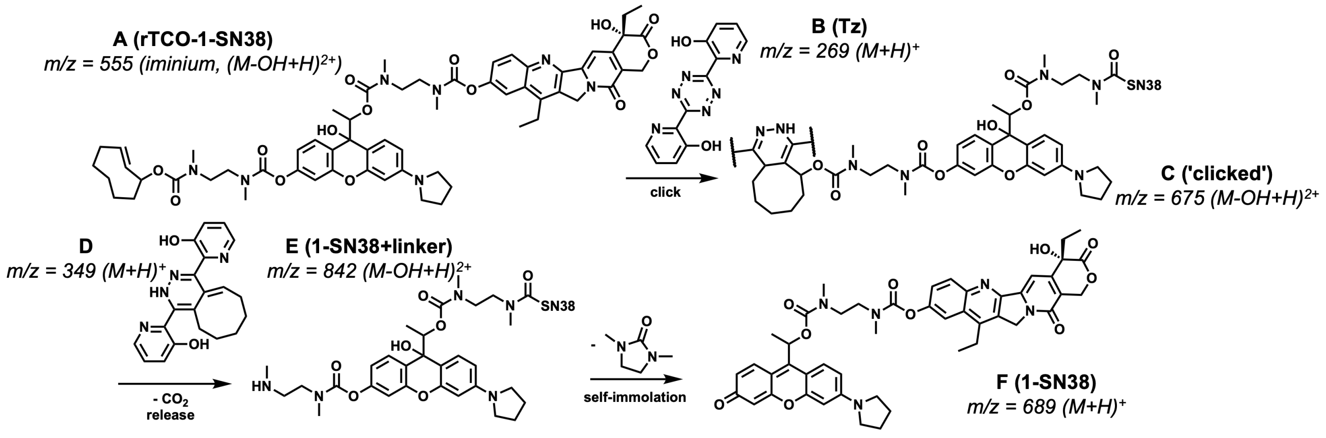

click-to-release in HEPES/MeCN 9:1:

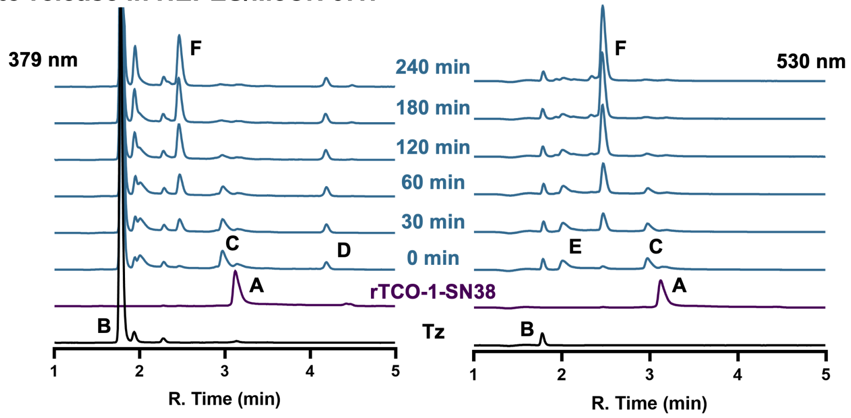

click-to-release in HEPES/MeCN 9:1 (2D contour chromatograms):

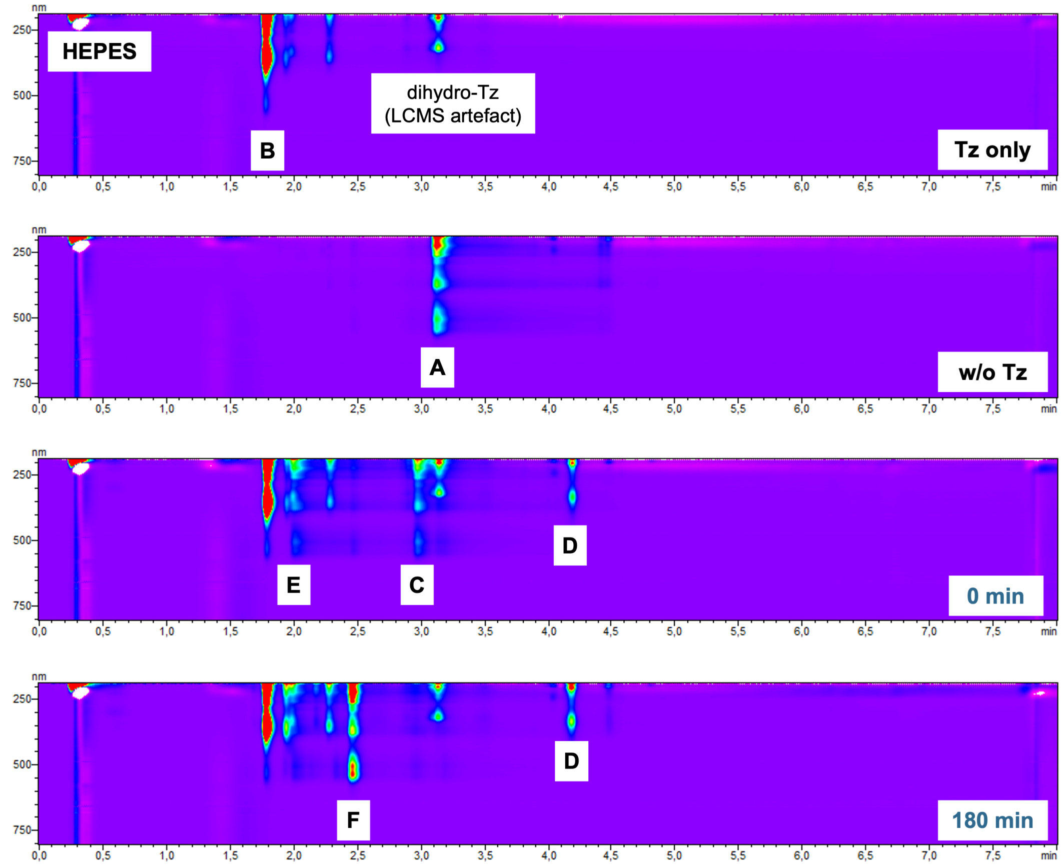

*Figure S26. Chromatograms of the click-to-release cascade reaction of **rTCO-1-SN38** (A) and **Tz** (B). The disappearance of the starting material (A) and the appearance of the intermediates (C and E) and the activated **1-SN38** (F) were monitored at 379 nm.*

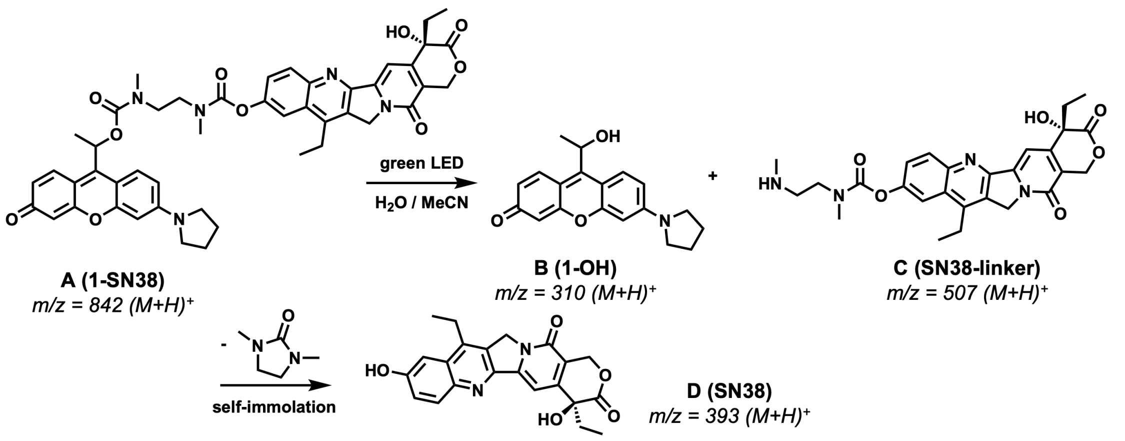

irradiation in HEPES/MeCN 9:1:

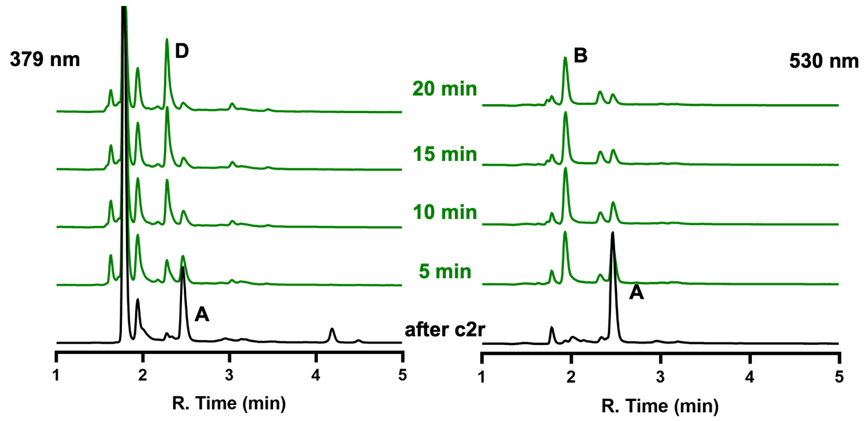

irradiation in HEPES/MeCN 9:1 (2D contour chromatograms):

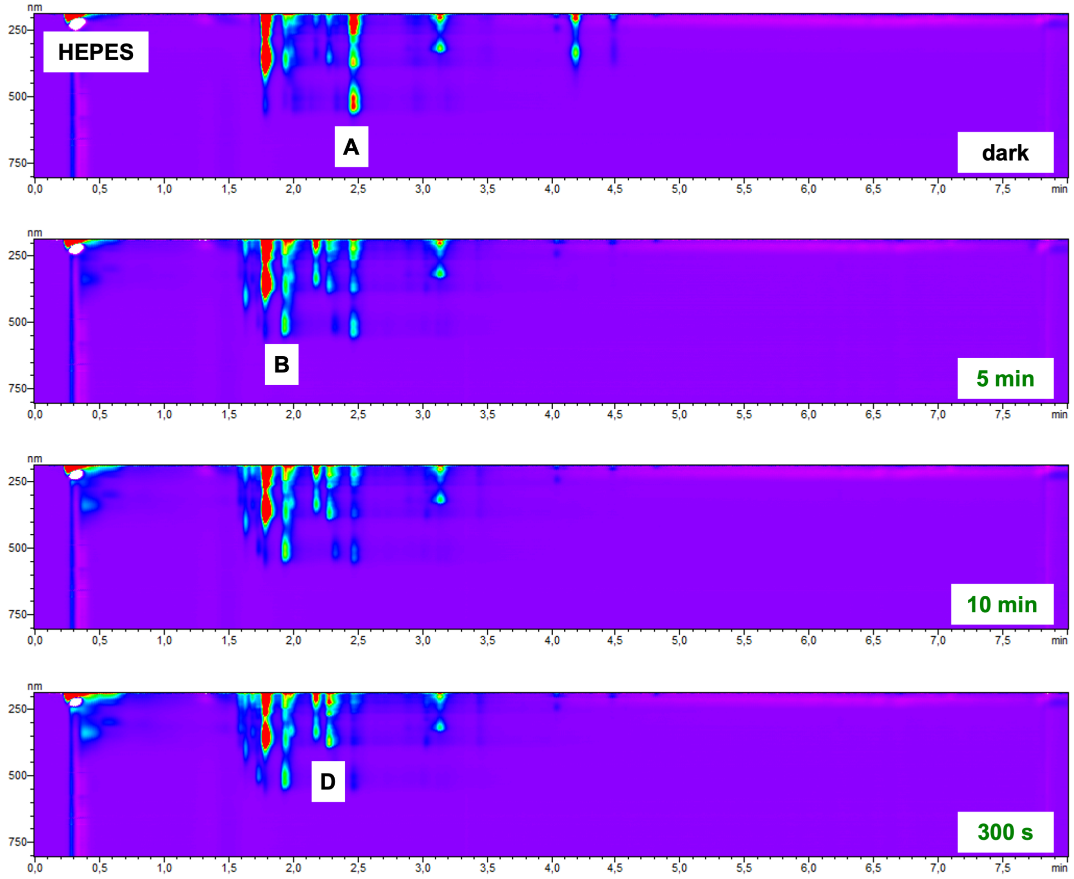

*Figure S27. Chromatograms of the photolysis of **1-SN38** from the c2r reaction mixture as followed by HPLC-UV/Vis-MS instrument. The appearance of the SN38 compounds (C and D) and the decrease of **1-SN38** (A) was monitored at the absorption maxima of each compound (379 nm and 530 nm, respectively).*

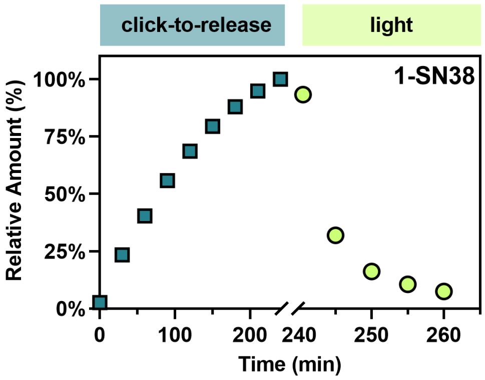

*Figure S28. Relative amounts of **1-SN38** during the c2r reaction cascade of **rTCO-1-SN38** and **Tz** and upon green light irradiation (same reaction mixture) based on the HPLC-UV/Vis integrals. Note the different scaling of the Time axis.*

4.7 Uncaging Experiments Followed by Optical Spectroscopy Methods

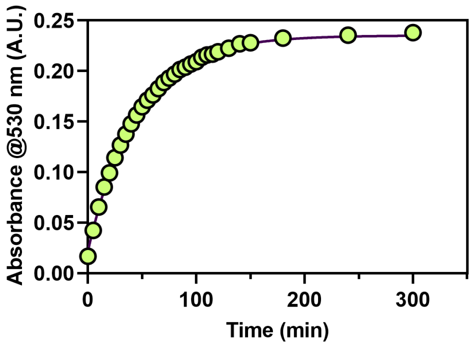

Figure S29. Absorption spectra of the leuco-oxo transformation after diluting the basic stock solution to 5 μM and setting the pH to 7.0.

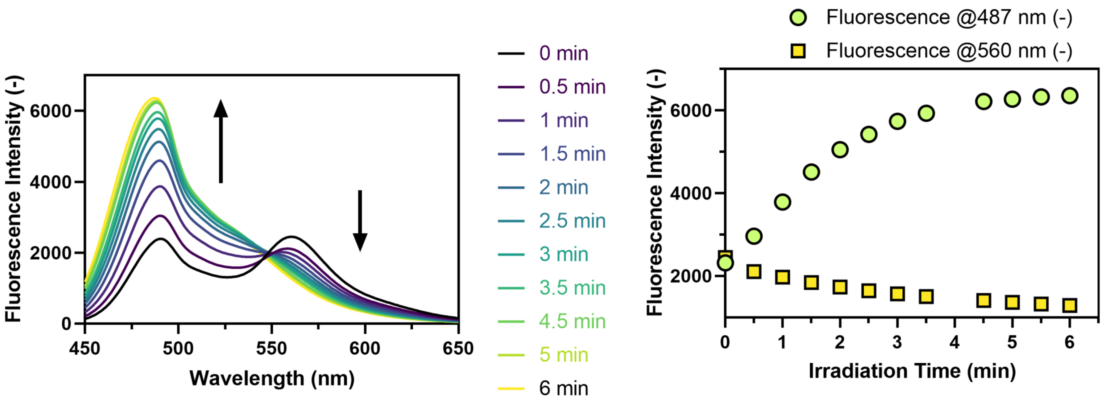

*Figure S30. Fluorescence spectra (excitation wavelength: 425 nm) of the photo-uncaging of the released **1-Cou** upon irradiation with green light, measured after confirming successful c2r reaction of **rTCO-1-Cou** with **Tz**.*

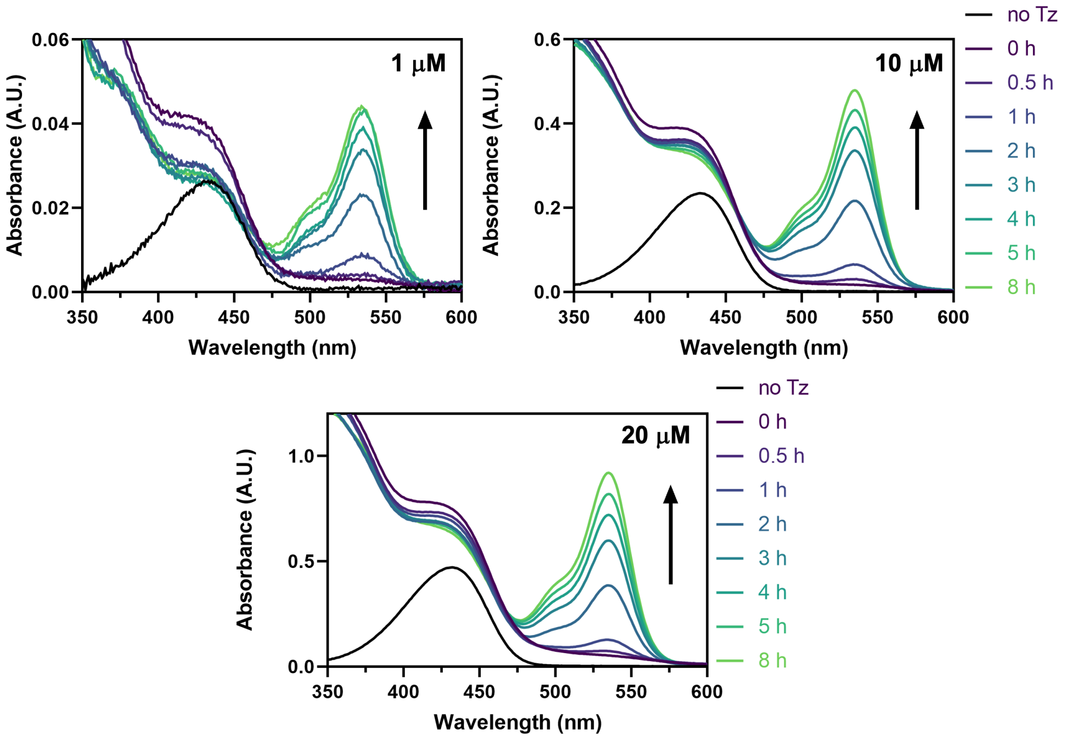

*Figure S31. Absorption spectra of the click-to-release reaction of **rTCO-1-Cou** upon reaction with **Tz** at various concentrations measured in HEPES buffer (pH 7.4, 10 mM, containing 25% MeCN)*

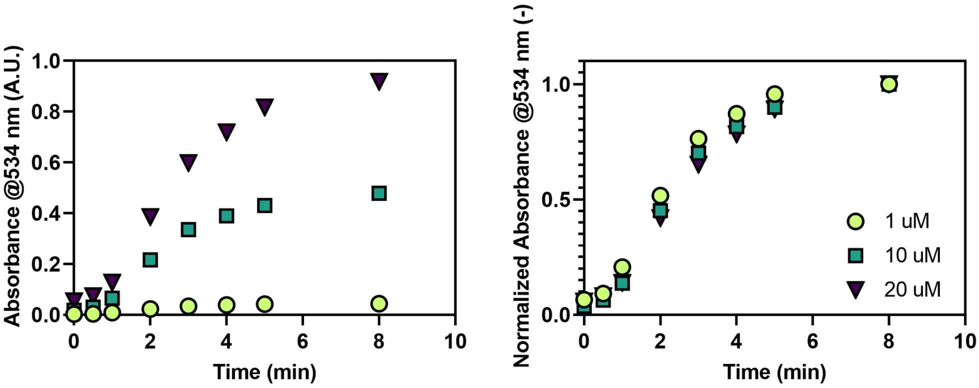

*Figure S32. Time-dependent absorption (left) and normalized absorption profile (right) of the click-to-release reaction of **rTCO-1-Cou** upon reaction with **Tz** at various concentrations measured in HEPES buffer (pH 7.4, 10 mM, containing 25% MeCN)*

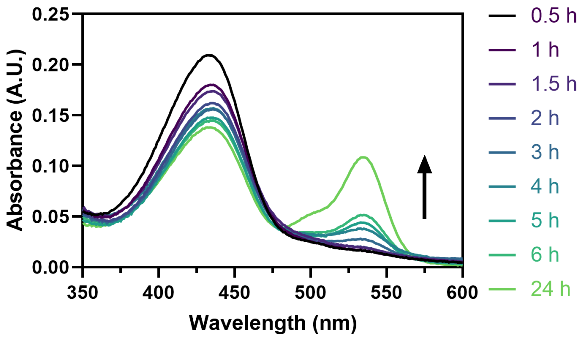

*Figure S33. Absorption spectra of the click-to-release reaction of **rTz-1-Cou** (10 μM) upon reaction with **TCO-halo** at various concentrations measured in HEPES buffer (pH 7.4, 10 mM, containing 25% MeCN)*

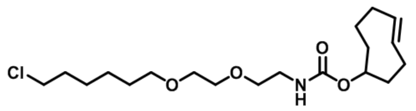

TCO-halo

Figure S34. Structure of ***TCO-halo*** [8]

5. Fluorescence Microscopy

5.1 Cell Sample Preparation for Fluorescence Imaging Experiments

SK-OV-3 cells were cultured in McCoy's 5A (Modified) Medium (Gibco 22330021) supplemented with 10% FBS (Gibco A5256801) and 1% penicillin-streptomycin (Gibco 15140122). Cells were maintained at 37 °C in a 5% CO_2 atmosphere and subcultured every 3-4 days using trypsin, for a maximum of 20 passages.

5.2 Live Cell Fluorescence Imaging

SK-OV-3 cells (8,000 cells/well) were transferred into µ-Slide 8-well chambered coverslips (Ibidi 80827) and incubated for 40 h at 37 °C in a 5% CO_2 atmosphere. Cells were then treated with either 1 µM **1-SN38** or 10 µM **rTCO-1-SN38**, either in the presence or absence of **Tz** (50 µM, 5 equiv.). For co-treatment, **rTCO-1-SN38** and **Tz** were pre-incubated in McCoy's 5A medium at room temperature for 24 hours before administration. After 1 h of treatment, the cells were subjected to confocal microscopy analyses.

Confocal images were acquired using a Leica TCS SP8 STED 3x microscope with a 552 nm excitation laser. Images were acquired with a Leica HC PL APO CS2 40x/1.30 oil immersion objective, and fluorescence was detected in the 565-615 nm range using HyD detector. Image processing was carried out with ImageJ software.

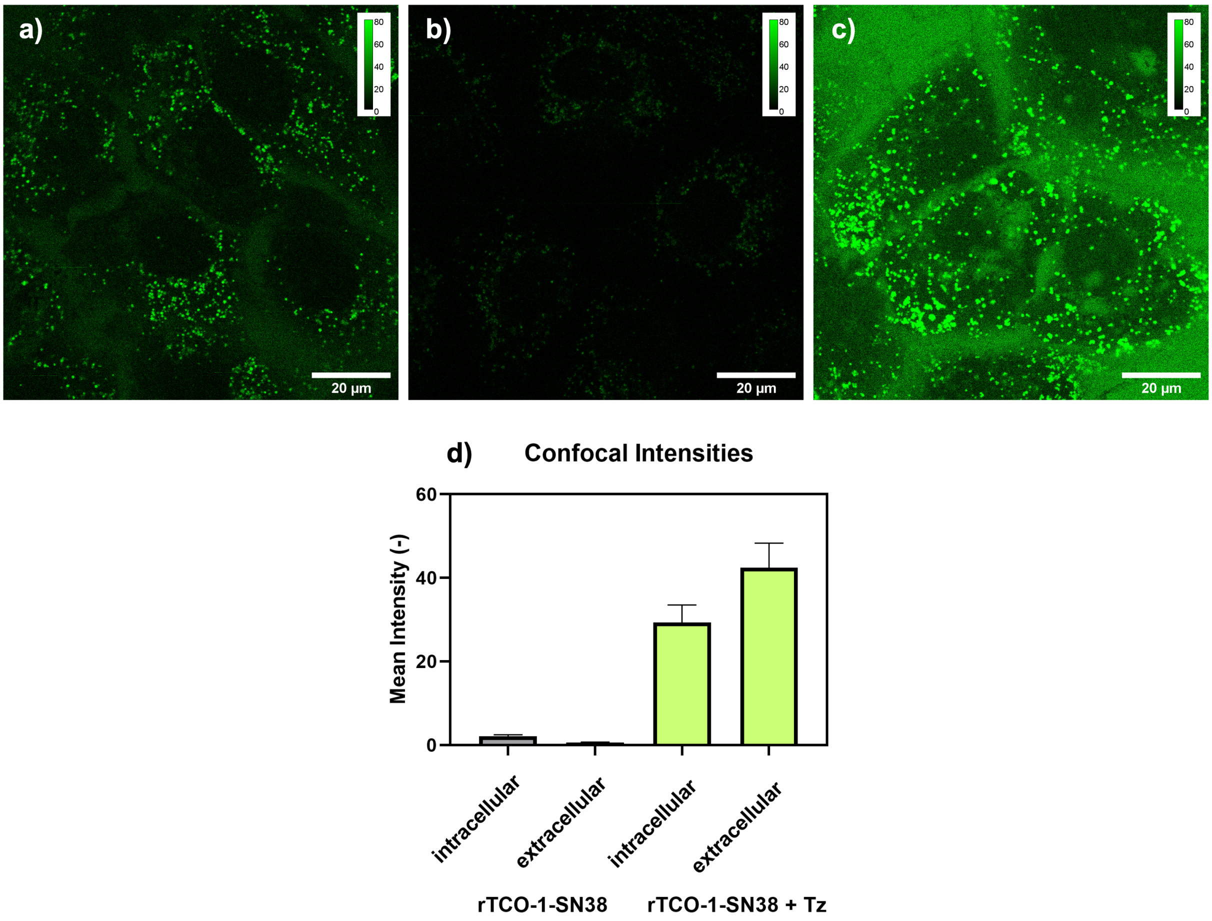

Figure S35. Zoomed-in confocal microscopy images of live SK-OV-3 cells treated with (a) ***1-SN38*** *(b)* ***rTCO-1-SN38*** *and (c)* ***rTCO-1-SN38*** *treated with* ***Tz*** *together with the measured fluorescence intensities of pixels (5 ROIs selected for each measurement)*

5.3 Colocalization Studies

SK-OV-3 cells (8,000 cells/well) were transferred into µ-Slide 8-well chambered coverslips (Ibidi 80827) and incubated for 40 h at 37 °C in a 5% CO_2 atmosphere. Cells were then treated with either 1 µM **1-SN38** or 10 µM **rTCO-1-SN38**, in the presence or absence of **Tz** (50 µM, 5 equiv.), in combination with 1 nM LysoTracker Deep Red (Invitrogen L12492) or 1 nM MitoTracker Deep Red (Invitrogen M46753) for 30 min. Following treatment, cells were subjected to confocal microscopy with a Leica TCS SP8 STED 3x microscope. The images were acquired with a Leica HC PL APO CS2 40x/1.30 oil immersion objective using dual-channel detection (channel 1 and 2: HyD detector). Excitation/emission settings were as follows: green channel (for **1-SN38** and **rTCO-1-SN38**), excitation at 552 nm and emission acquired at the 565-615 nm range; red channel (for organelle trackers), excitation at 638 nm and emission acquired at the 650-800 nm range. Image processing was performed with ImageJ software.

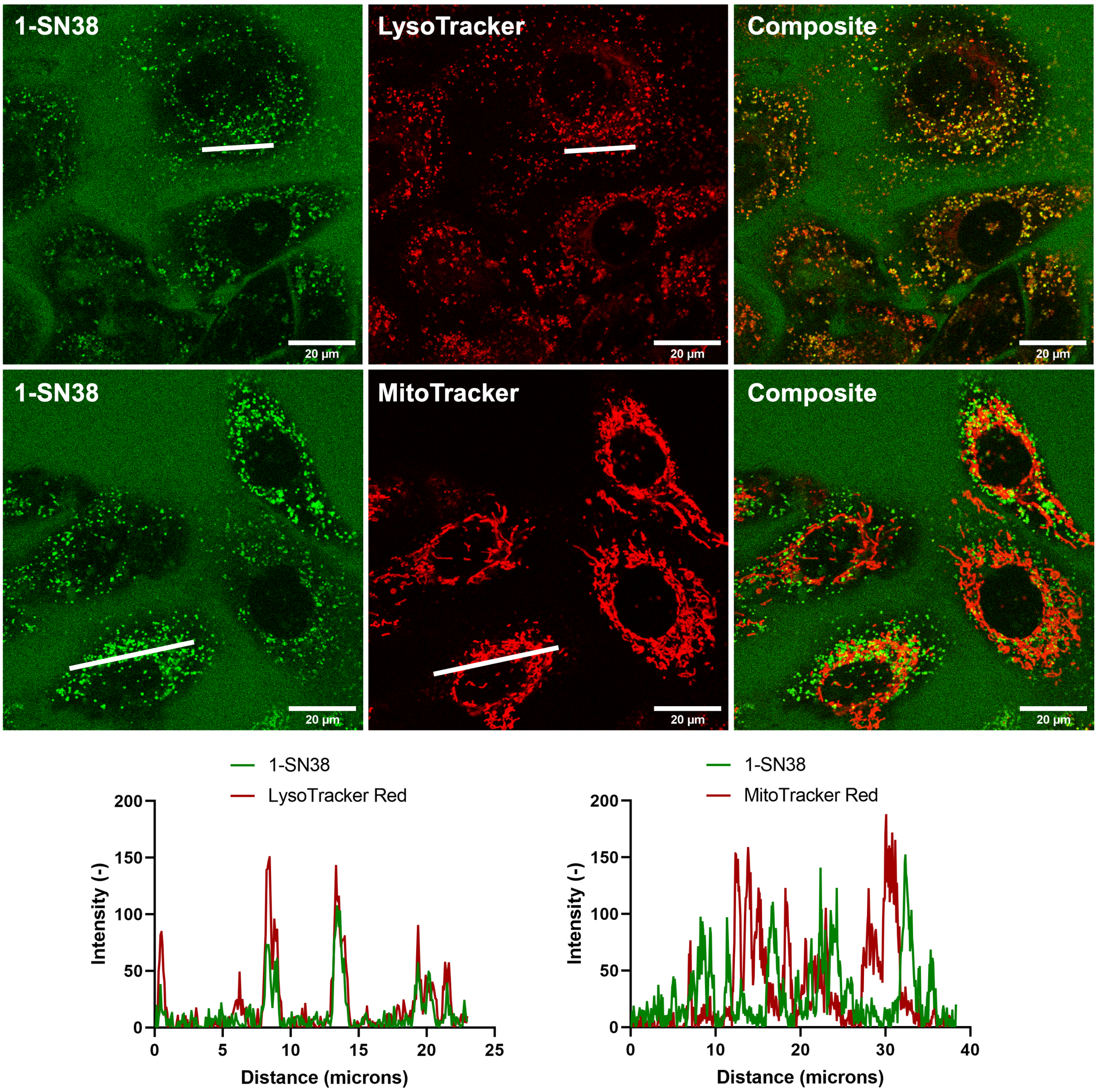

*Figure S36. Confocal microscopy images of SK-OV-3 cells treated with 1 µM **1-SN38** and 1 nM LysoTracker Deep Red or MitoTracker Deep Red. The white lines are plotted on the graphs to show the colocalization of **1-SN38** with the trackers.*

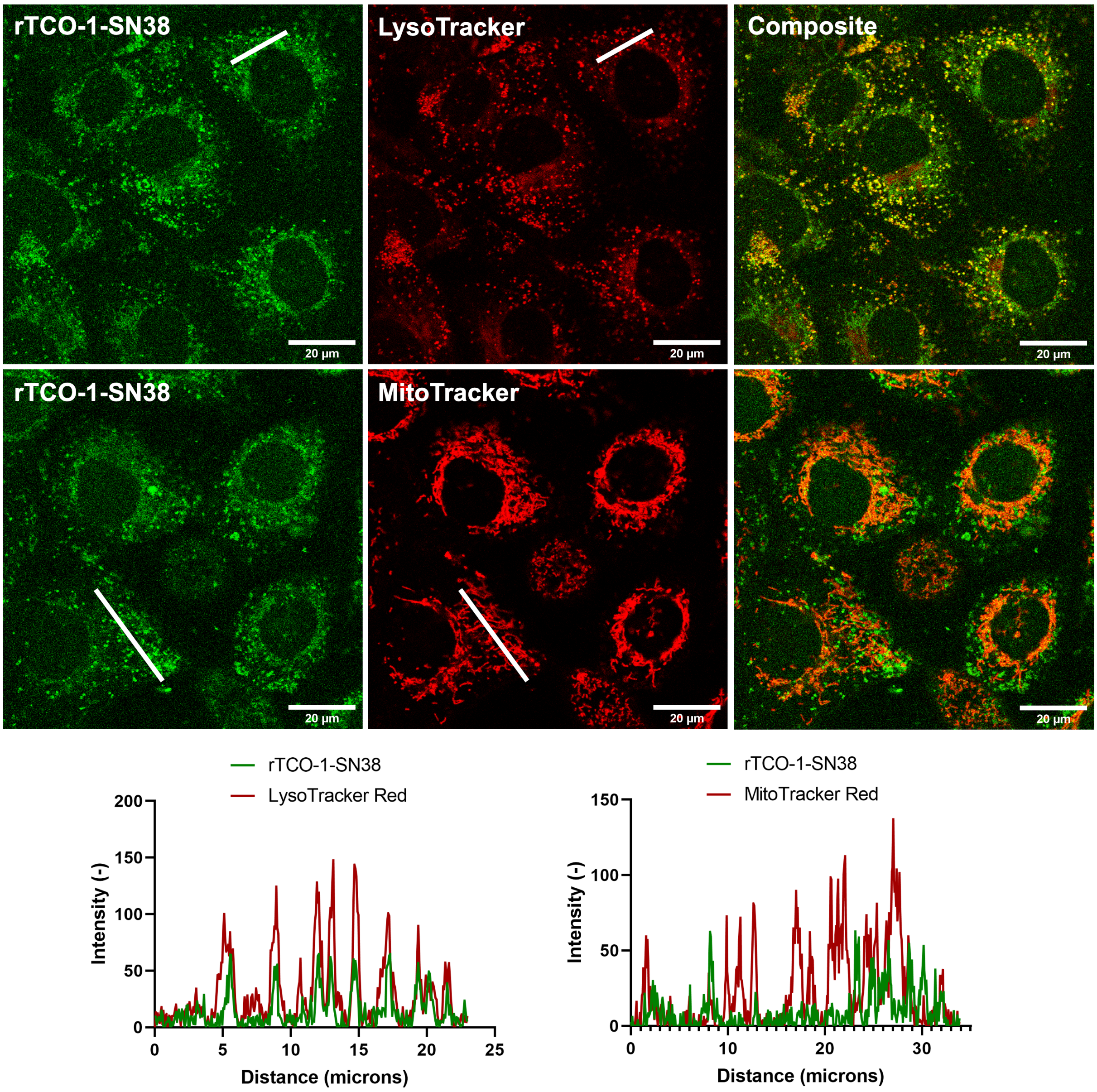

*Figure S37. Confocal microscopy images of SK-OV-3 cells treated with 10 µM **rTCO-1-SN38** and 1 nM LysoTracker Deep Red or MitoTracker Deep Red. The white lines are plotted on the graphs to show the colocalization of **rTCO-1-SN38** with the trackers.*

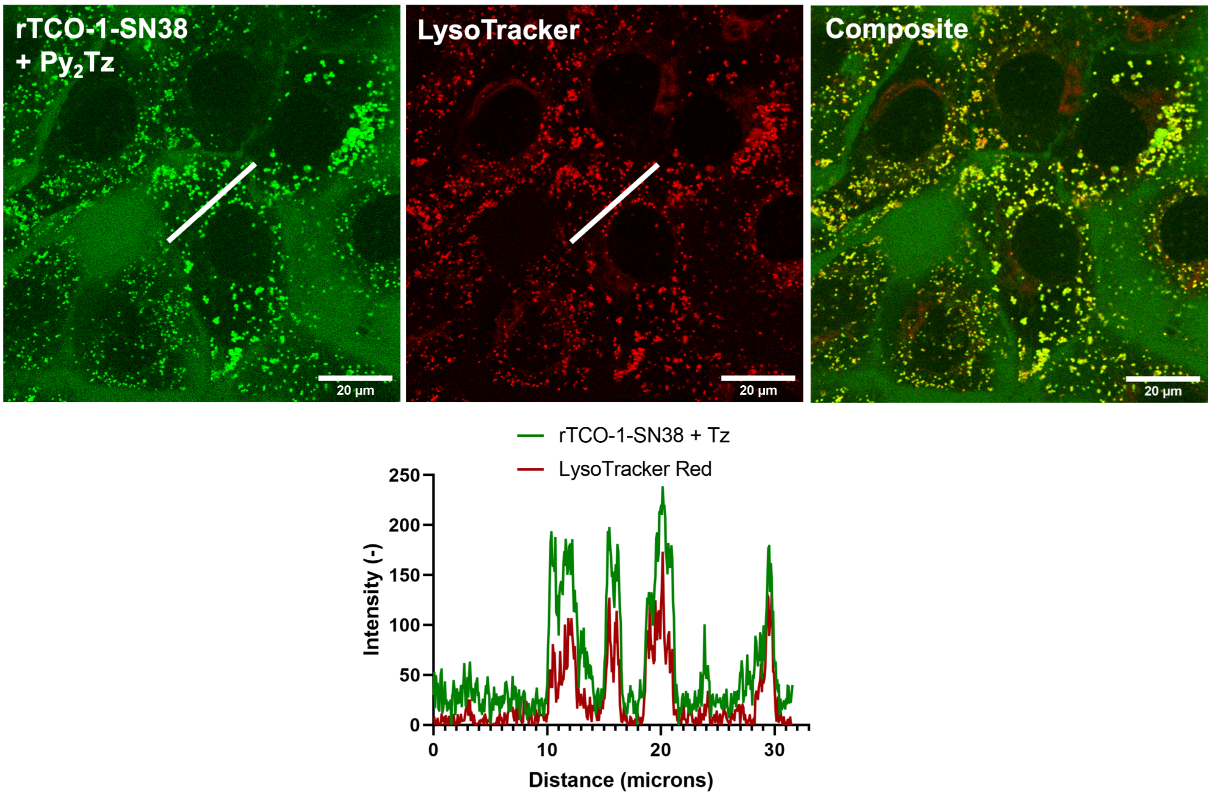

*Figure S38. Confocal microscopy images of SK-OV-3 cells treated with 10 μM **rTCO-1-SN38** and 50 μM **Tz** together with 1 nM LysoTracker Deep Red. The white lines are plotted on the graphs to show the colocalization with LysoTracker Deep Red.*

6. Viability Studies

6.1 Experimental Details

A viability test was performed to evaluate the toxicity of **SN38**, **1-SN38** (in the dark or after light irradiation), and **rTCO-1-SN38** (in the presence or absence of 5 equivalents of **Tz**; in the dark or after light irradiation) on SK-OV-3 cells. Cells were transferred into a 48-well plate (4,500 cells/well) and incubated for 24 h at 37 °C in a 5% CO_2 atmosphere. Cells were maintained in McCoy's 5A (Modified) Medium (Gibco 22330021), supplemented with 10% FBS (Gibco A5256801) and 1% penicillin-streptomycin (Gibco 15140122). The compounds were applied at concentrations ranging from 10^{-12} to 10^{-5} M and incubated for 4 hours. Following treatment, cells were either exposed to light irradiation or kept in the dark. After irradiation or dark incubation, cells were maintained at 37 °C in 5% CO_2 atmosphere for an additional 72 hours.

Cell viability was assessed using the MTT assay. After the incubation period, culture medium was replaced with 0.5 mg/mL MTT (3-(4,5-dimethylthiazol-2-yl)-2,5-diphenyltetrazolium bromide; Thermo Scientific L11939) solution in complete DMEM (Dulbecco's modified Eagle's medium (Gibco 41965039), supplemented with 10% FBS (Gibco A5256801), 1% penicillin-streptomycin (Gibco 15140122), 1% GlutaMAX (Gibco 35050061) and 1% sodium pyruvate (Gibco 11360070)), followed by a 120-minute incubation at 37 °C in the dark. Formazan crystals were solubilized in 250 µL DMSO, and absorbance was measured at 540 nm using a Biotek Synergy 2 Cytation 3 imaging plate reader with Gen5 software (version 3.08, Biotek, Winooski, VT, USA). Cell viability was expressed as a ratio of readings (n = 3) relative to untreated control cells. Welch's *t*-test was applied as statistical analysis to compare data using GraphPad Prism software (ver. 8.0.1). Significance levels are shown as *p*-values and presented as heat map.

6.2 Viability Curves and IC50 Determination

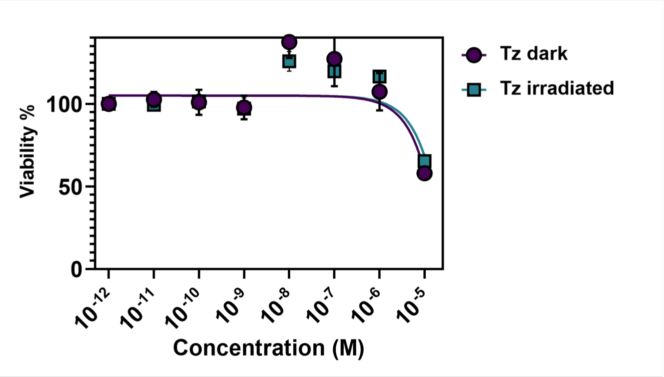

Figure S39. Concentration-dependent effects of ***Tz*** *on normalized viabilities together with their IC50 curves. Irradiation time: 10 min green light*

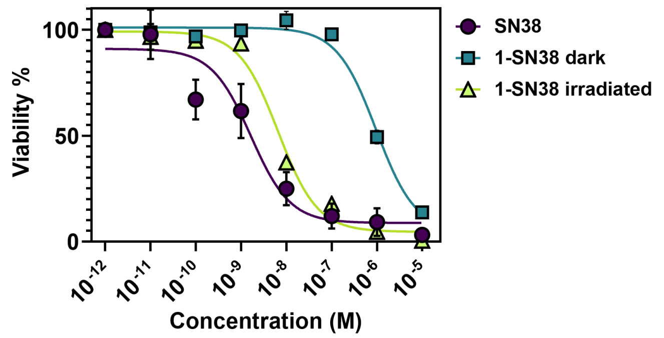

Figure S40. Concentration-dependent effects of ***SN38*** *and* ***1-SN38*** *with or without irradiation on normalized viabilities together with their IC50 curves. Irradiation time: 10 min green light*

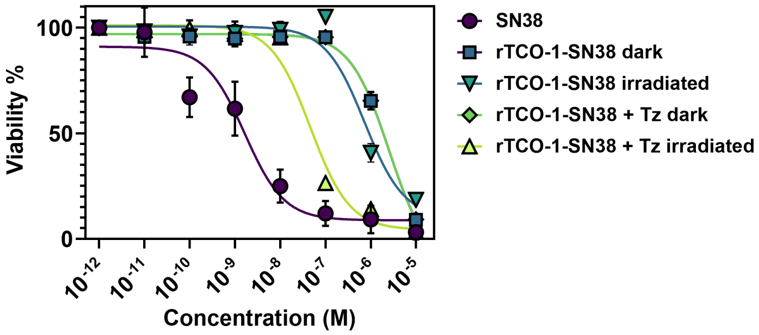

Figure S41. Concentration-dependent effects of ***SN38*** *and* ***rTCO-1-SN38*** *with or without irradiation with or without* ***Tz*** *(5 equiv.) on normalized viabilities together with their IC50 curves. Irradiation time: 10 min green light*

significance level
not significant (n.s.)
p<0.05
p<0.01
p<0.001
p<0.0001

p	1-SN38 dark	1-SN38 light	rTCO-1-SN38 no Tz dark	rTCO-1-SN38 no Tz light	rTCO-1-SN38 with Tz dark	rTCO-1-SN38 with Tz light
**SN38**	p<0.001	not significant	p<0.001	p<0.001	p<0.001	p<0.05
**1-SN38 dark**		p<0.0001	not significant		not significant	
**1-SN38 light**				p<0.0001		p<0.01
**rTCO-1-SN38 no Tz dark**				p<0.05	not significant	
**rTCO-1-SN38 no Tz light**						p<0.0001
**rTCO-1-SN38 with Tz dark**						p<0.0001

Table S1 Comparison based on significance of the normalized viabilitiy values of the 100 nM treatment using various compounds/conditions.

7. NMR Spectra and HPLC-MS Data

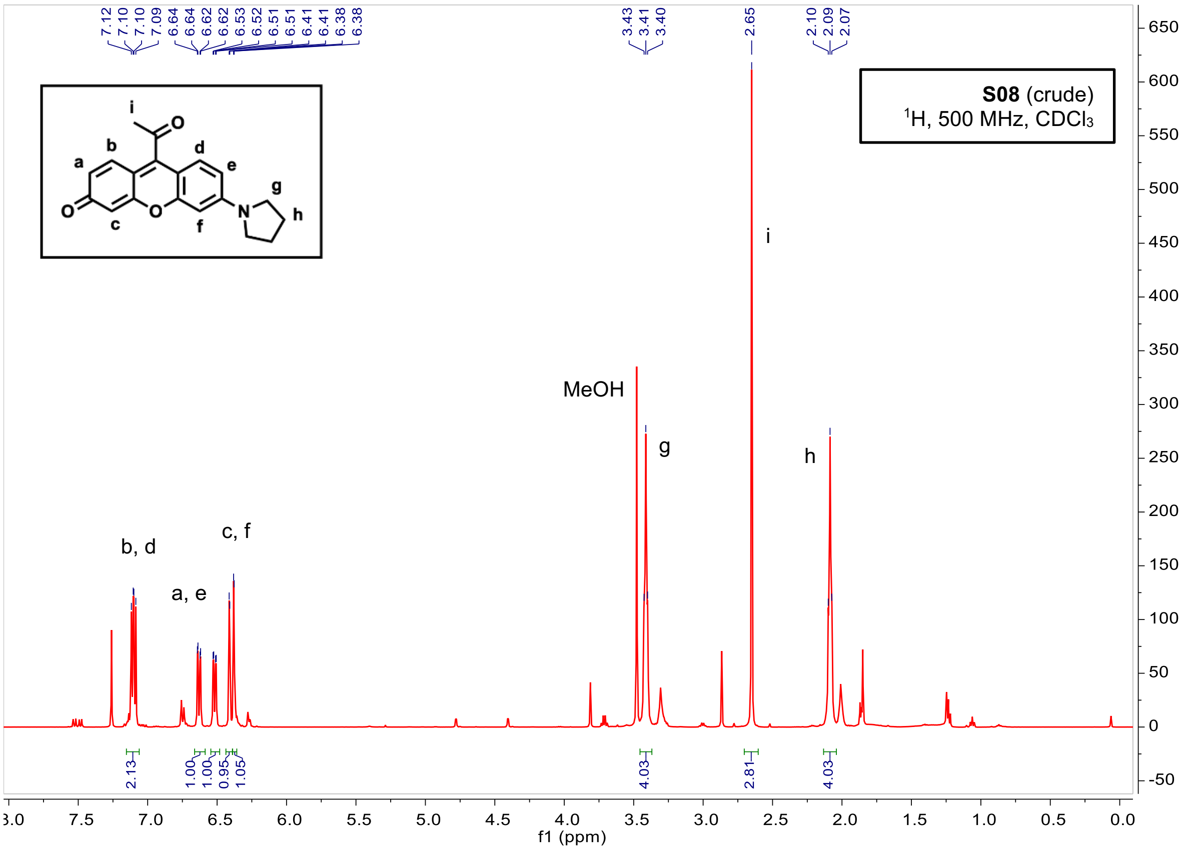

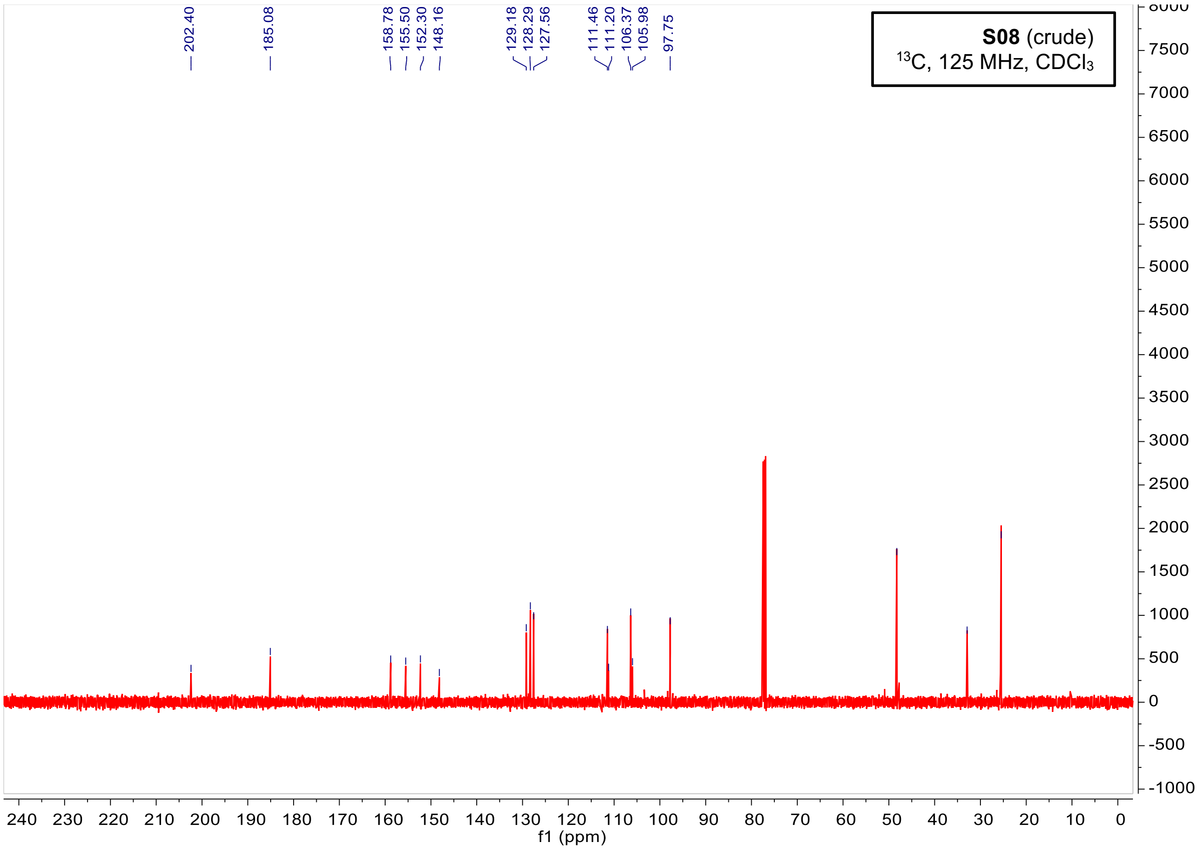

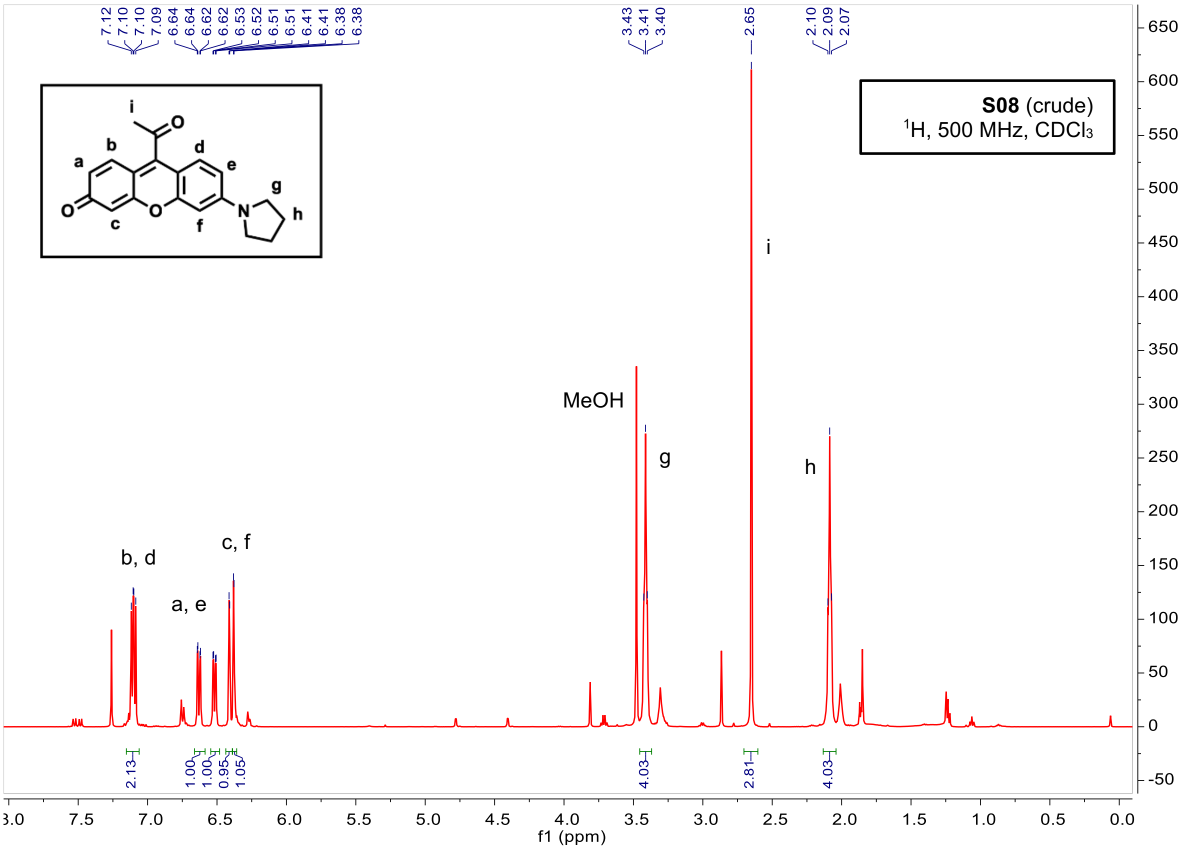
S08 (crude)
1H, 500 MHz, CDCl3
MeOH
b, d
a, e
c, f
g
i
h
f1 (ppm)

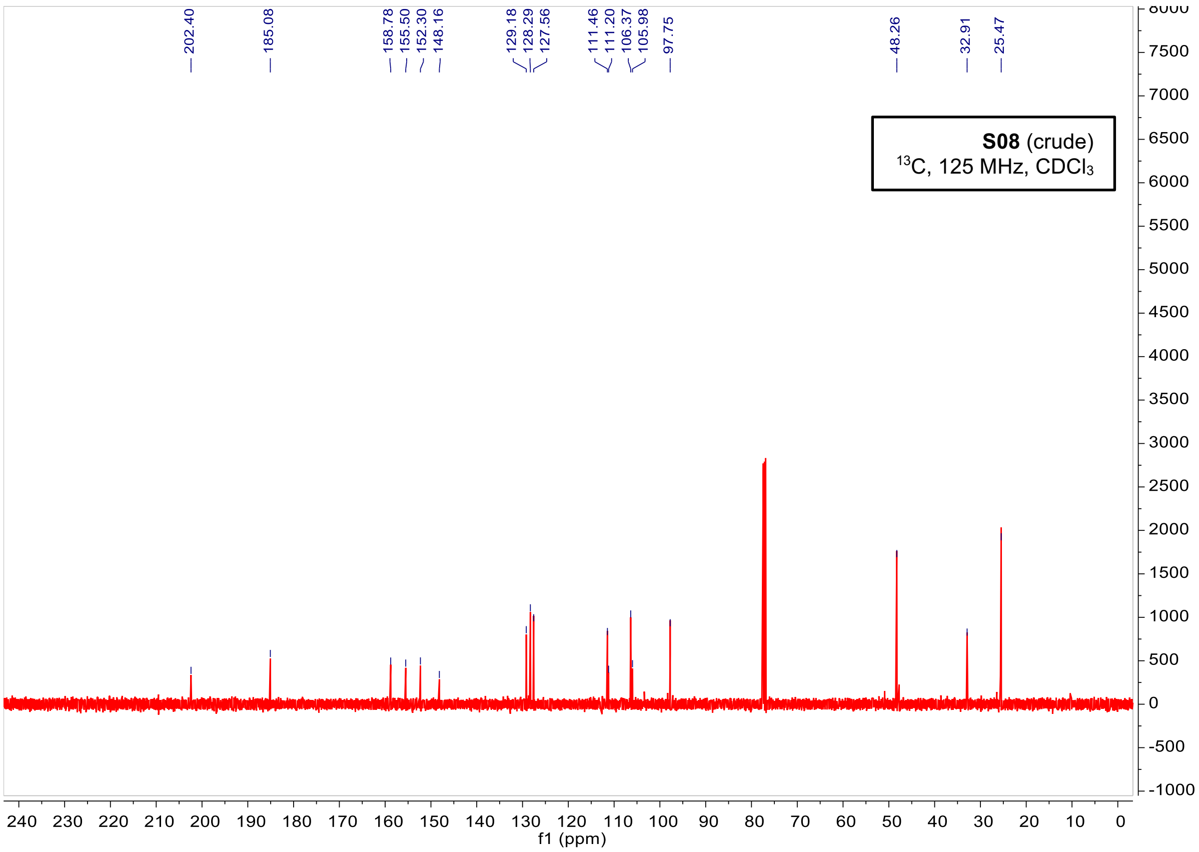
S08 (crude)
13C, 125 MHz, CDCl3
202.40
185.08
158.78
155.50
152.30
148.16
129.18
128.29
127.56
111.46
111.20
106.37
105.98
97.75
48.26
32.91
25.47
f1 (ppm)

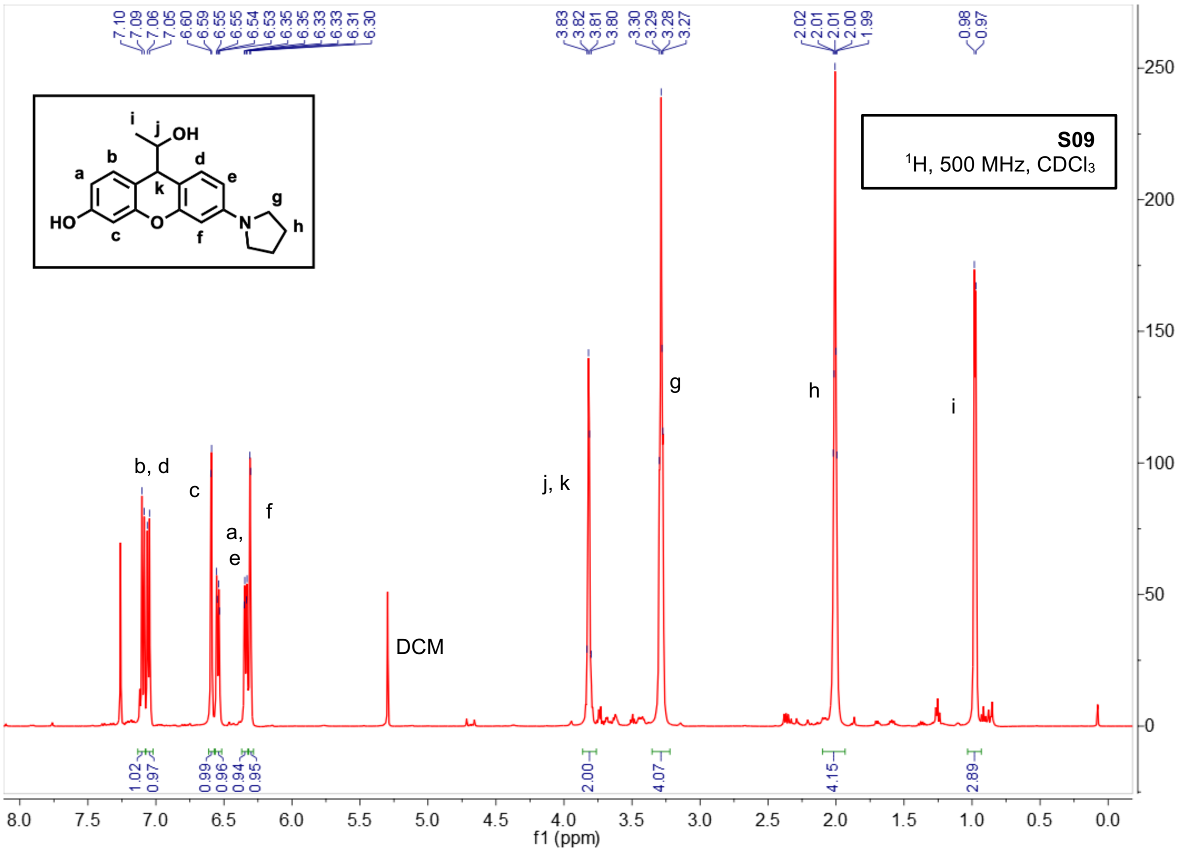
S09
1H, 500 MHz, CDCl3
HO
OH
N
a b c d e f g h i j k
b, d
c
a, e
f
DCM
j, k
g
h
i
f1 (ppm)

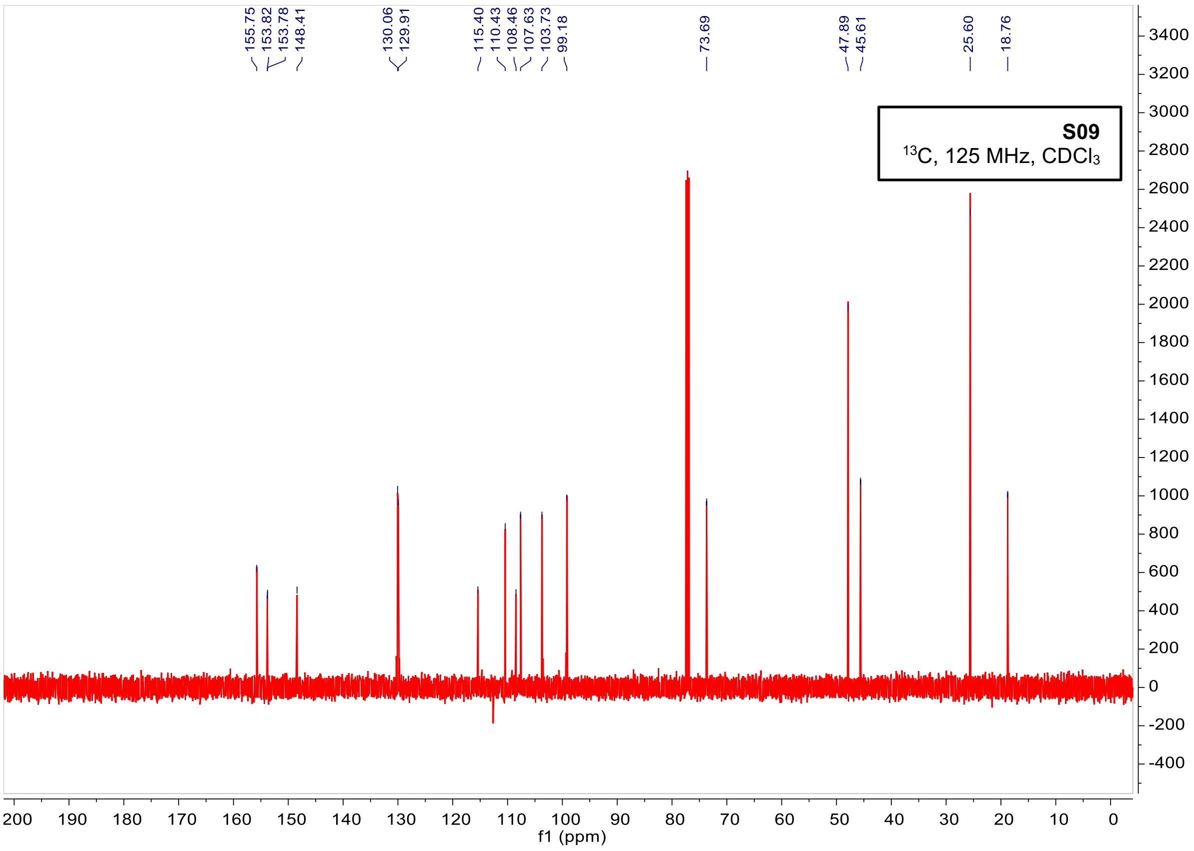
S09
13C, 125 MHz, CDCl3
155.75
153.82
153.78
148.41
130.06
129.91
115.40
110.43
108.46
107.63
103.73
99.18
73.69
47.89
45.61
25.60
18.76
f1 (ppm)

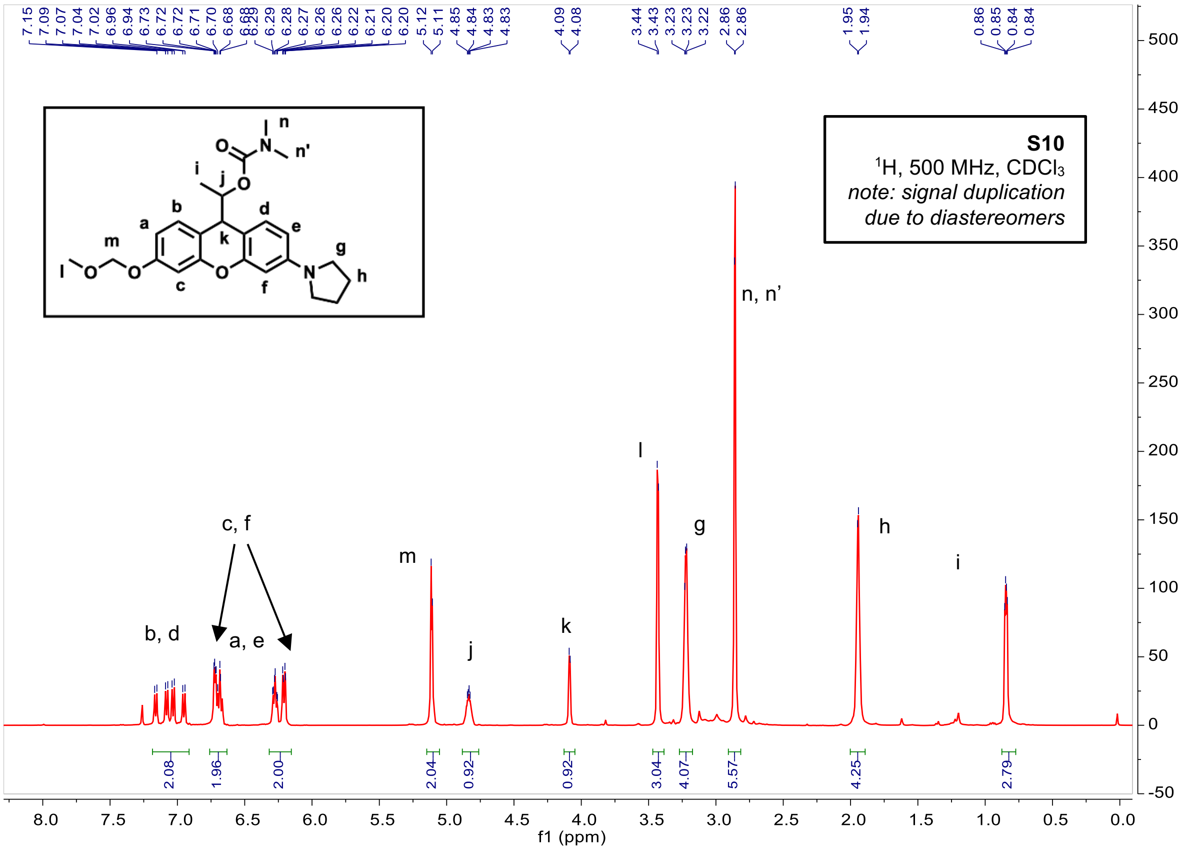
S10
^{1}H, 500 MHz, CDCl$_3$
note: signal duplication due to diastereomers
b, d
c, f
a, e
m
j
k
l
g
n, n'
h
i
f1 (ppm)

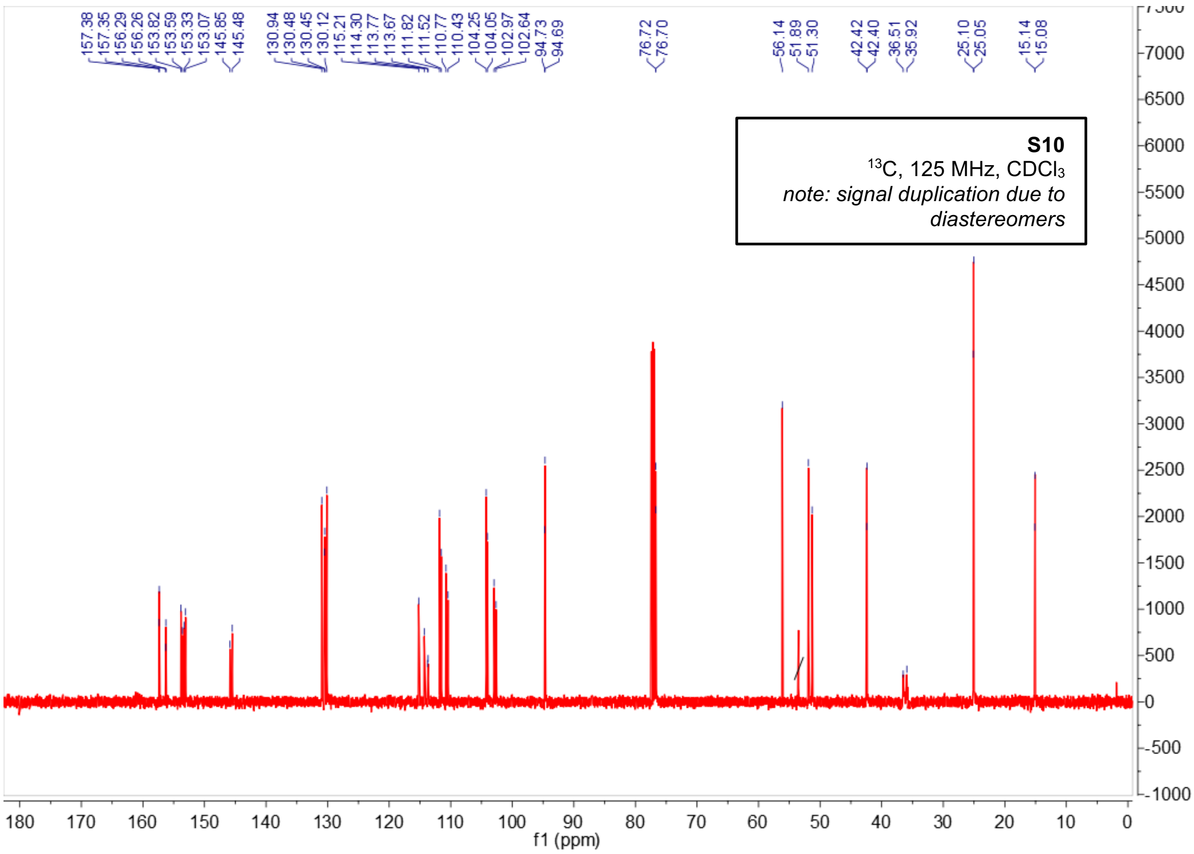
S10
^{13}C, 125 MHz, CDCl$_3$
note: signal duplication due to diastereomers
f1 (ppm)

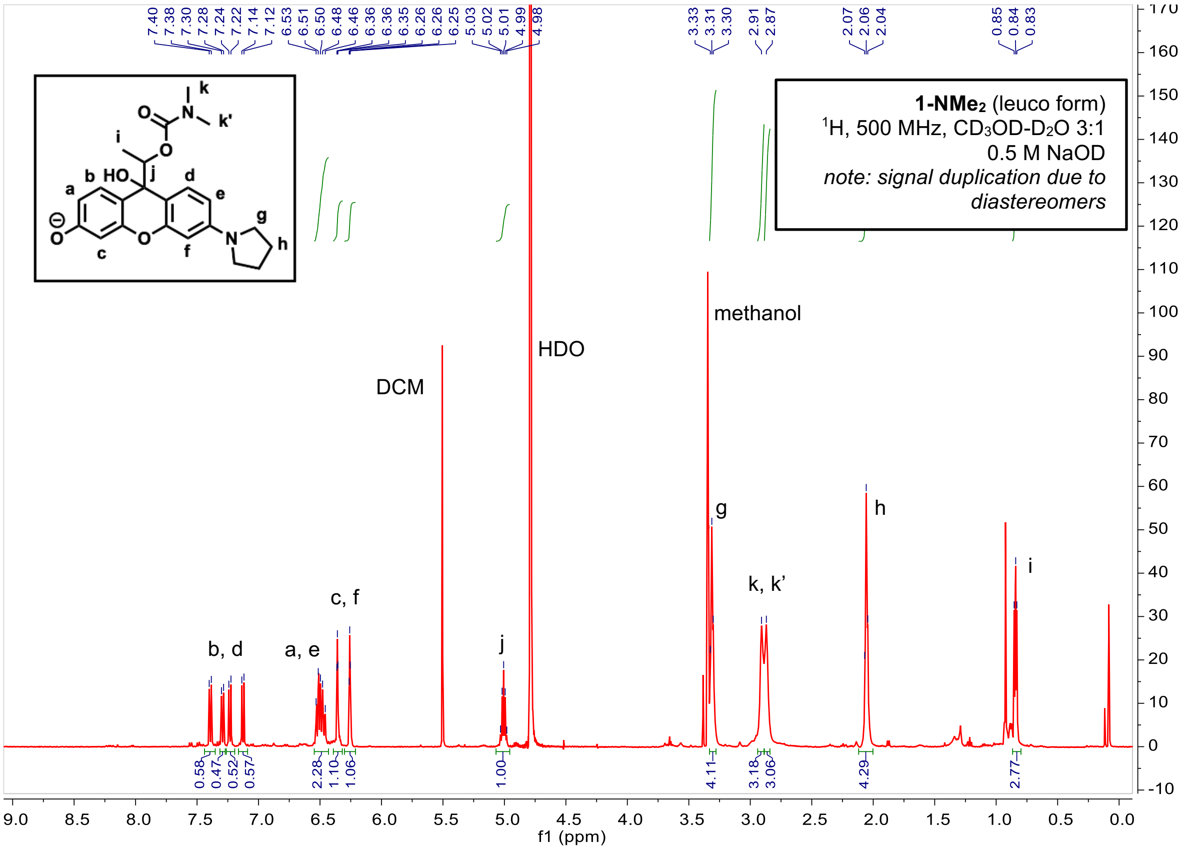
1-NMe2 (leuco form)
1H, 500 MHz, CD3OD-D2O 3:1
0.5 M NaOD
note: signal duplication due to diastereomers
DCM
HDO
methanol
g
h
i
k, k'
c, f
b, d
a, e
j
f1 (ppm)

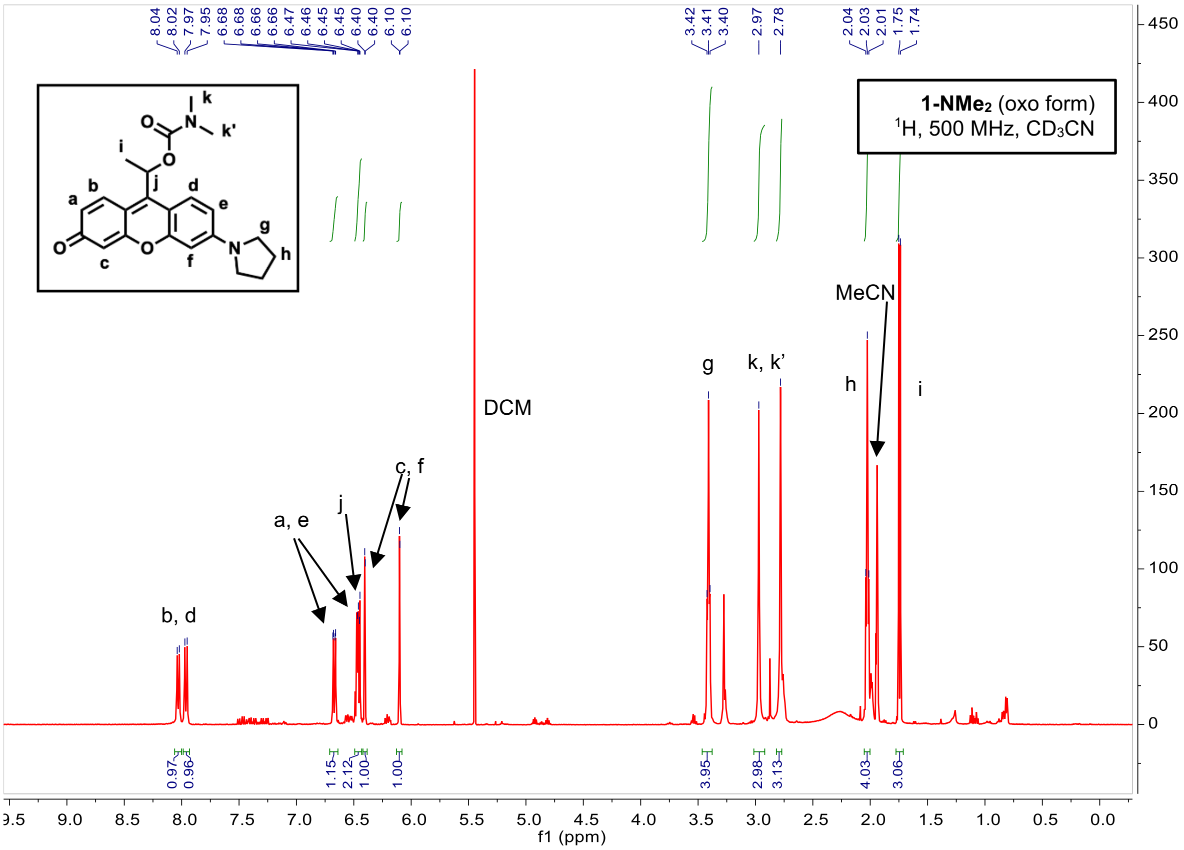
1-NMe2 (oxo form)
1H, 500 MHz, CD3CN
MeCN
DCM
g
k, k'
h
i
c, f
j
a, e
b, d
f1 (ppm)

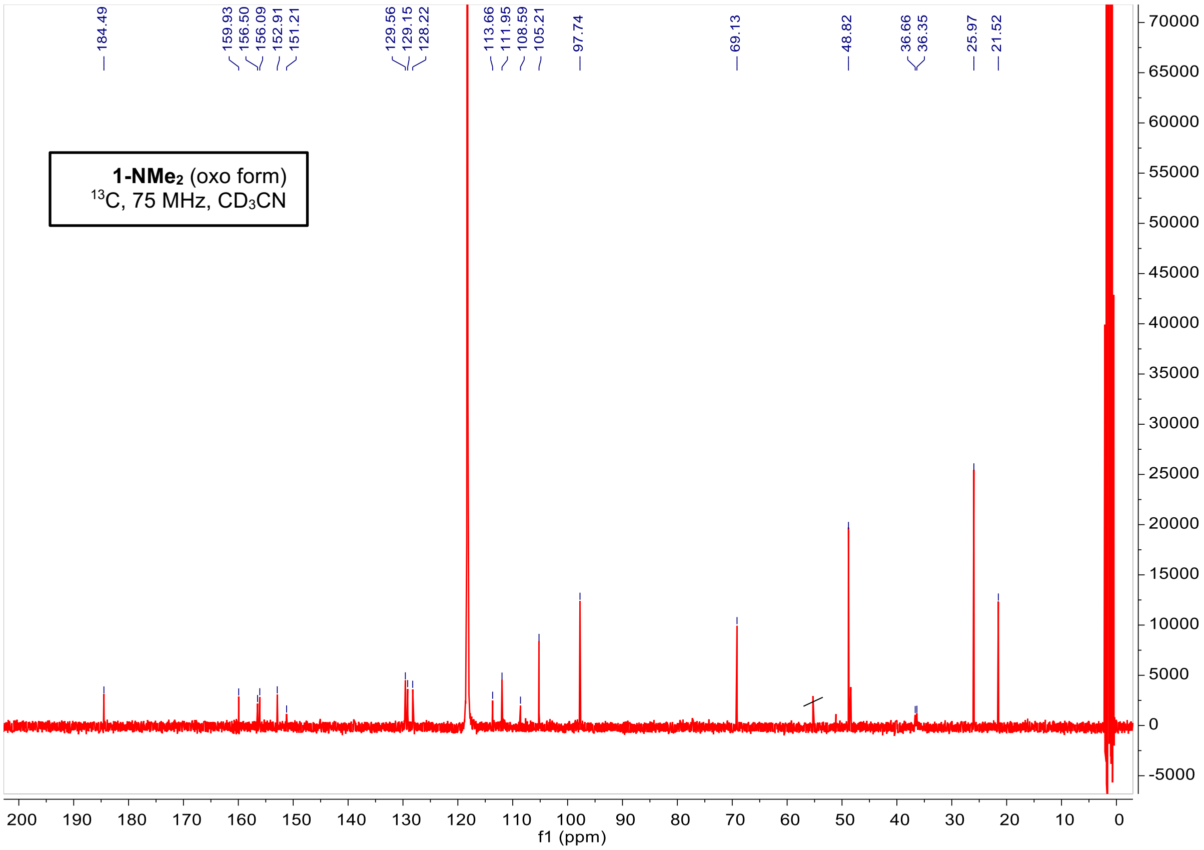
1-NMe2 (oxo form)
13C, 75 MHz, CD3CN
184.49
159.93
156.50
156.09
152.91
151.21
129.56
129.15
128.22
113.66
111.95
108.59
105.21
97.74
69.13
48.82
36.66
36.35
25.97
21.52
f1 (ppm)

1-NMe$_2$
$[M+H]^+$: 381

Chromatogram

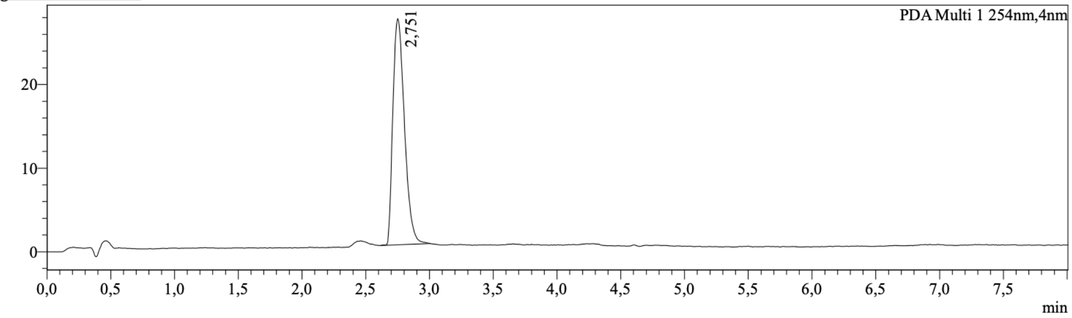

Peak Table

PDA Ch1 254nm

Peak#	Ret. Time	Peak Start	Peak End	Area	Height	Area/Height
1	2,751	2,619	3,008	171093	27075	6,319
Total				171093	27075	

Contour

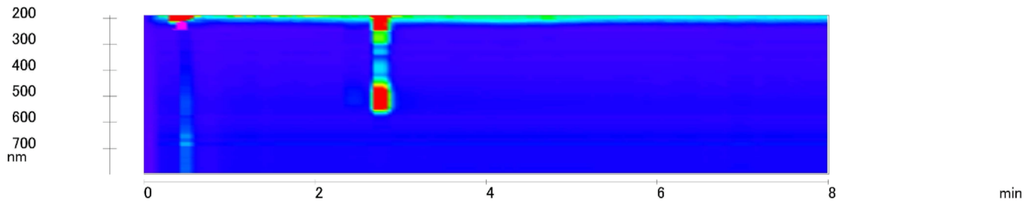

MS Chromatogram

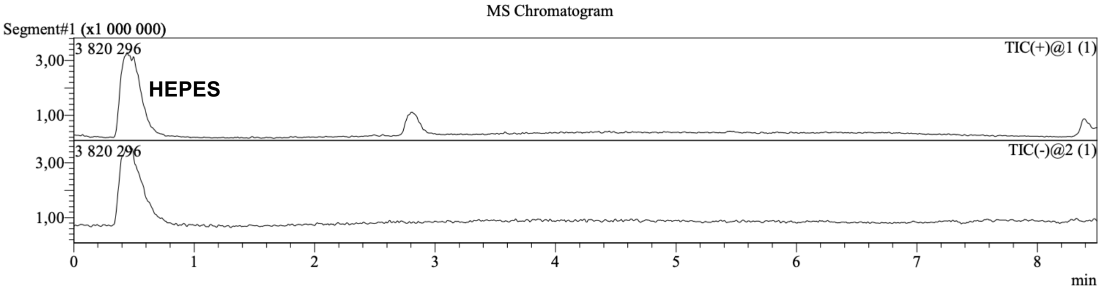

MS Spectrum

Line#:1 R.Time:----(Scan#:----)
MassPeaks:539
Spectrum Mode:Averaged 2,707-2,960(813-889) Base Peak:381(323316)
BG Mode:Averaged 1,813-2,253(545-677) Segment 1 - Event 1

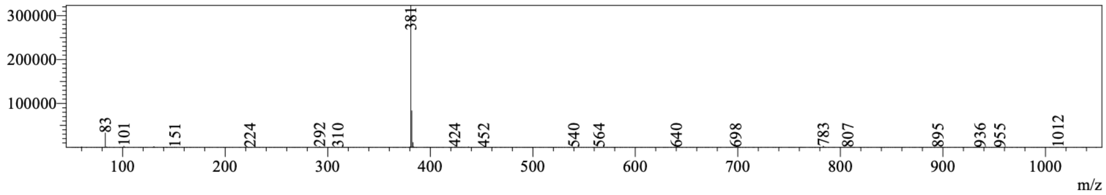

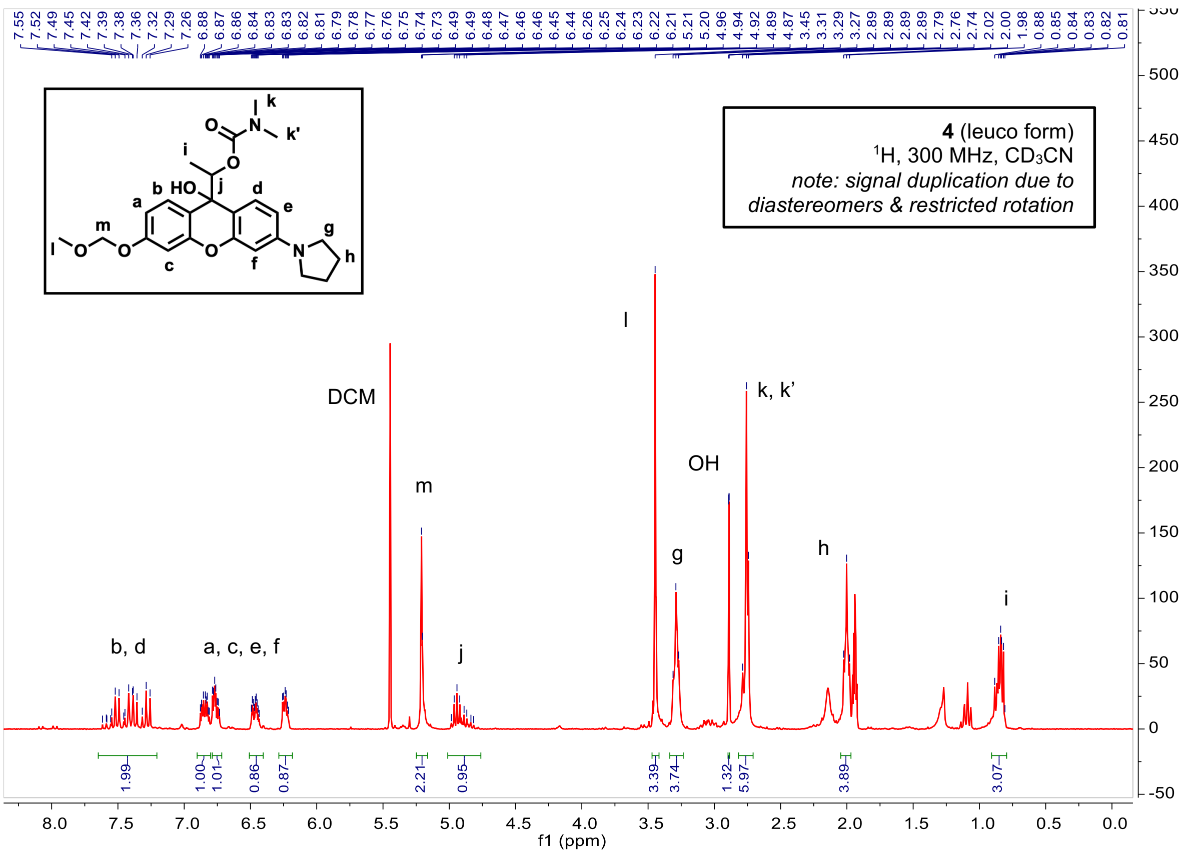
4 (leuco form)
1H, 300 MHz, CD3CN
note: signal duplication due to diastereomers & restricted rotation
DCM
m
j
l
g
OH
k, k'
h
i
b, d
a, c, e, f
f1 (ppm)

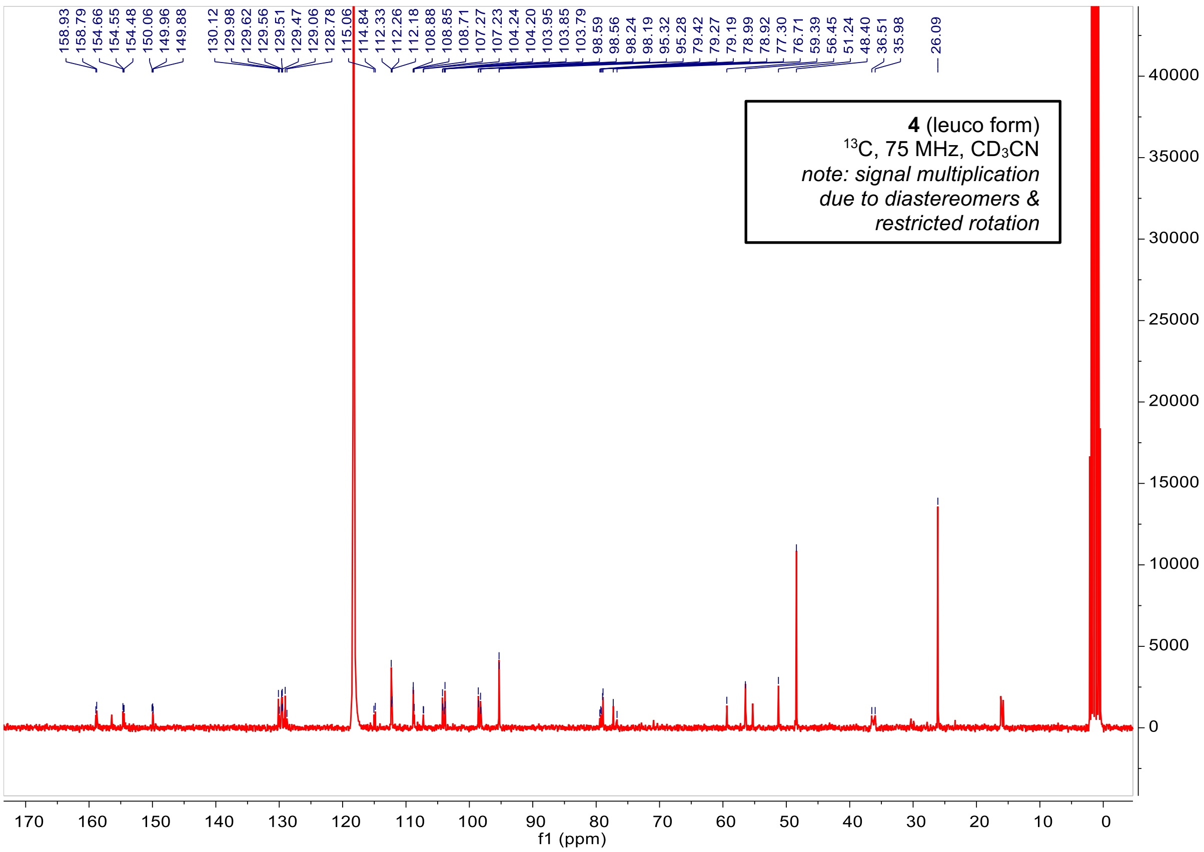
4 (leuco form)
13C, 75 MHz, CD3CN
note: signal multiplication due to diastereomers & restricted rotation
f1 (ppm)

4
$[M-H_2O+H]^+$: 425

Chromatogram

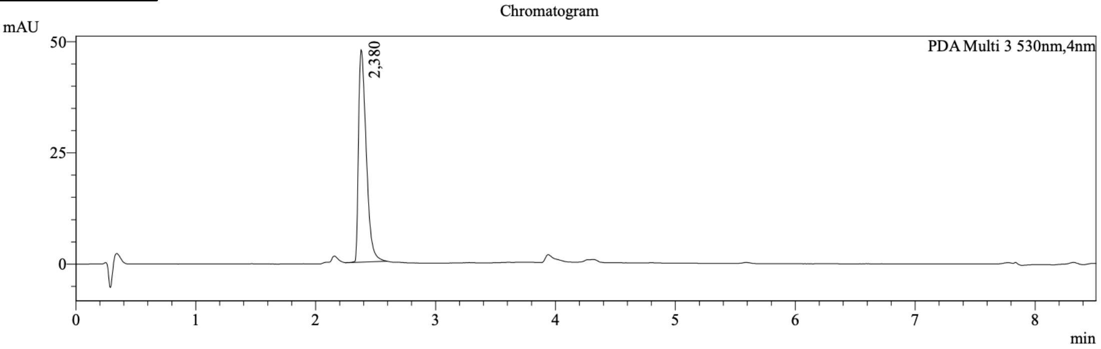

Peak Table

PDA Ch3 530nm

Peak#	Ret. Time	Peak Start	Peak End	Area	Height	Area/Height
1	2,380	2,251	2,592	212861	47798	4,453
Total				212861	47798	

Contour

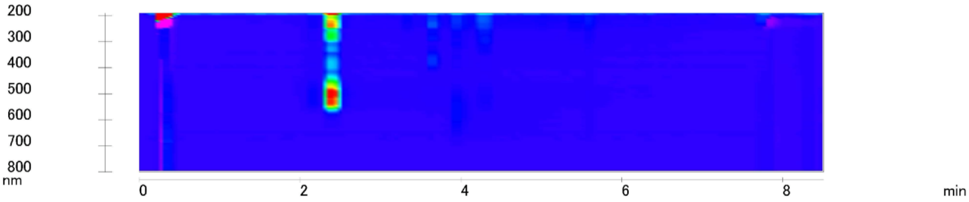

MS Chromatogram

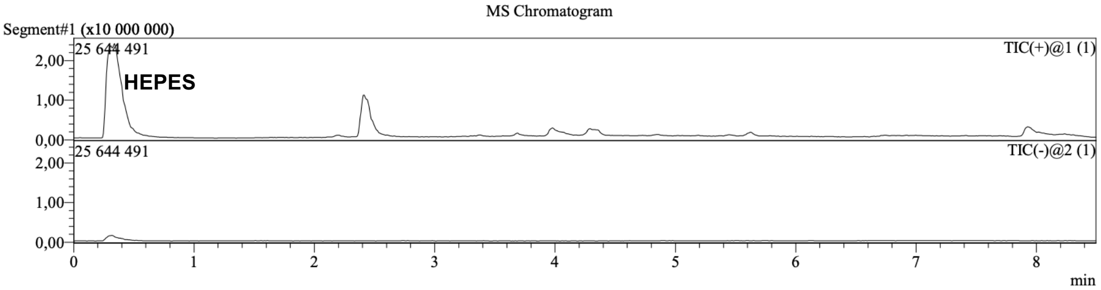

MS Spectrum

Line#:1 R.Time:----(Scan#:----)
MassPeaks:544
Spectrum Mode:Averaged 2,320-2,627(697-789) Base Peak:425(2566108)
BG Mode:Averaged 1,107-1,580(333-475) Segment 1 - Event 1

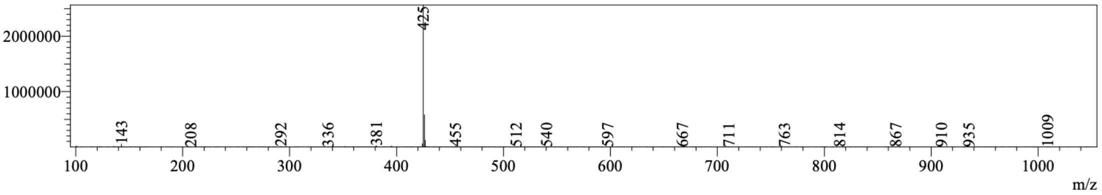

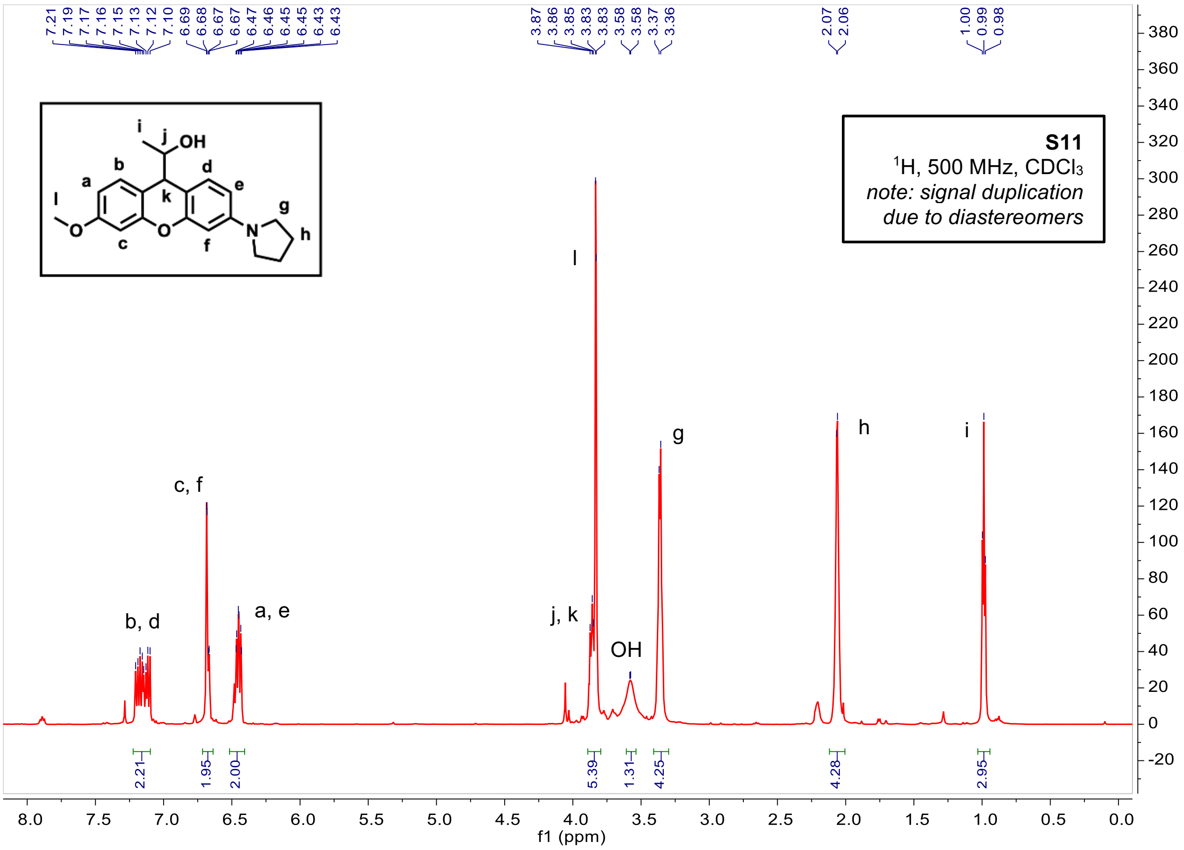

7.21 7.19 7.17 7.16 7.15 7.13 7.12 7.10 6.69 6.68 6.67 6.67 6.47 6.46 6.45 6.45 6.43 6.43
3.87 3.86 3.85 3.83 3.83 3.58 3.58 3.37 3.36
2.07 2.06
1.00 0.99 0.98
S11
^{1}H, 500 MHz, $CDCl_3$
note: signal duplication due to diastereomers
b, d
c, f
a, e
j, k
l
OH
g
h
i
2.21 1.95 2.00 5.39 1.31 4.25 4.28 2.95
f1 (ppm)

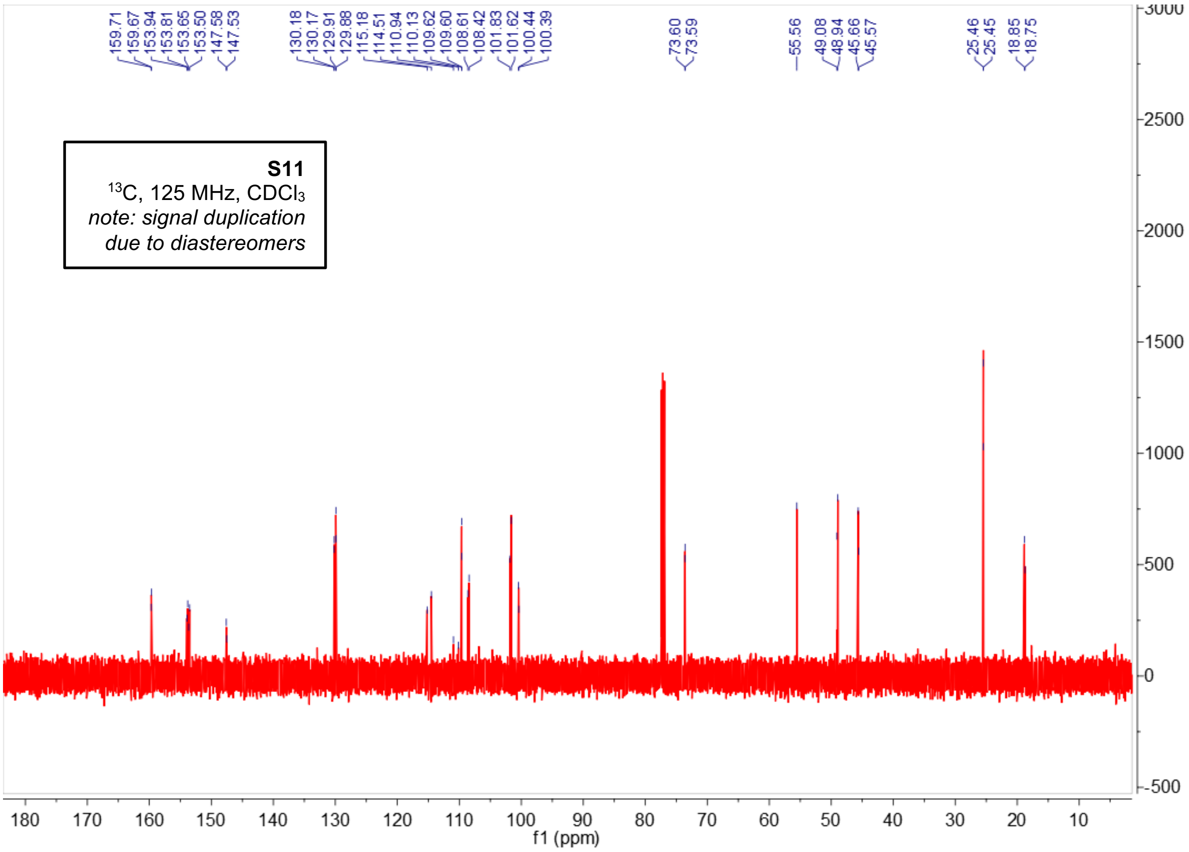

159.71 159.67 153.94 153.81 153.65 153.50 147.58 147.53
130.18 130.17 129.91 129.88 115.18 114.51 110.94 110.13 109.62 109.60 108.61 108.42 101.83 101.62 100.44 100.39
73.60 73.59
55.56 49.08 48.94 45.66 45.57
25.46 25.45 18.85 18.75
S11
^{13}C, 125 MHz, $CDCl_3$
note: signal duplication due to diastereomers
f1 (ppm)

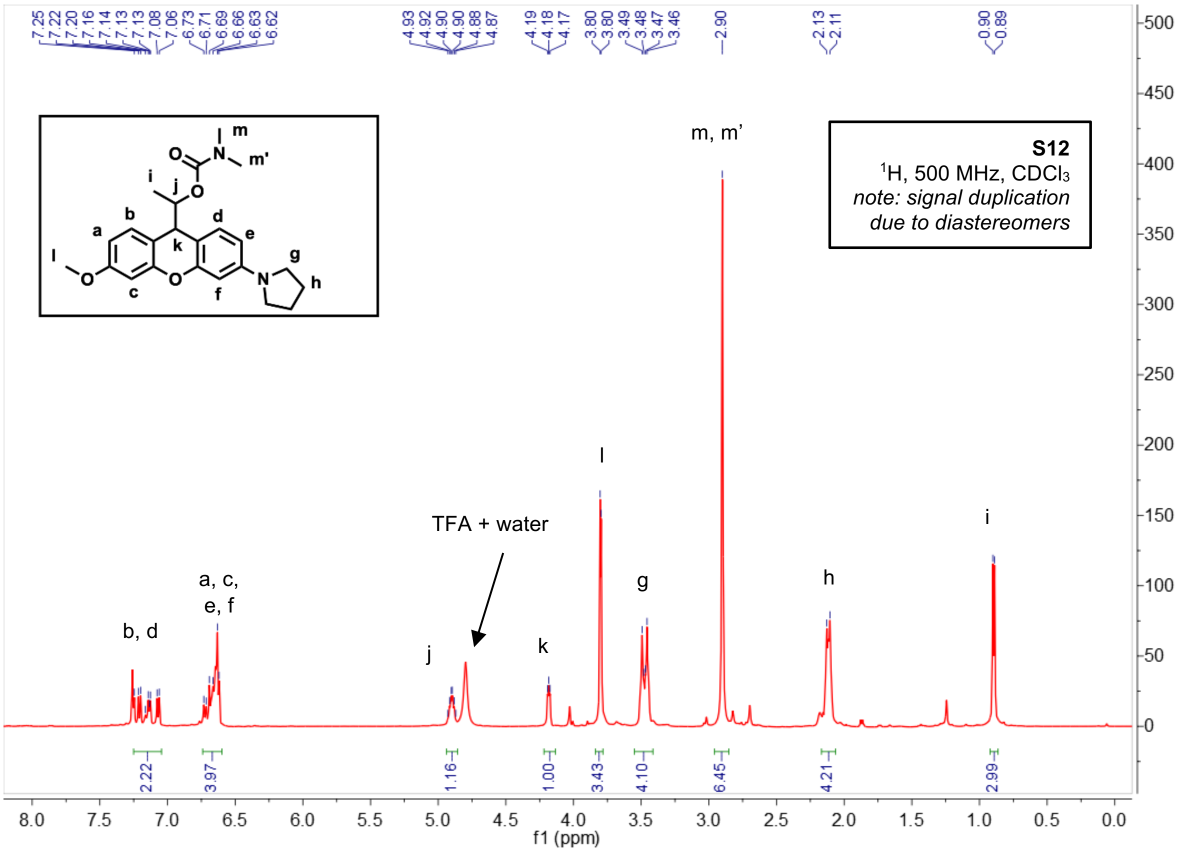
S12
^{1}H, 500 MHz, $CDCl_3$
note: signal duplication due to diastereomers
m, m'
l
TFA + water
a, c, e, f
b, d
j
k
g
h
i
f1 (ppm)

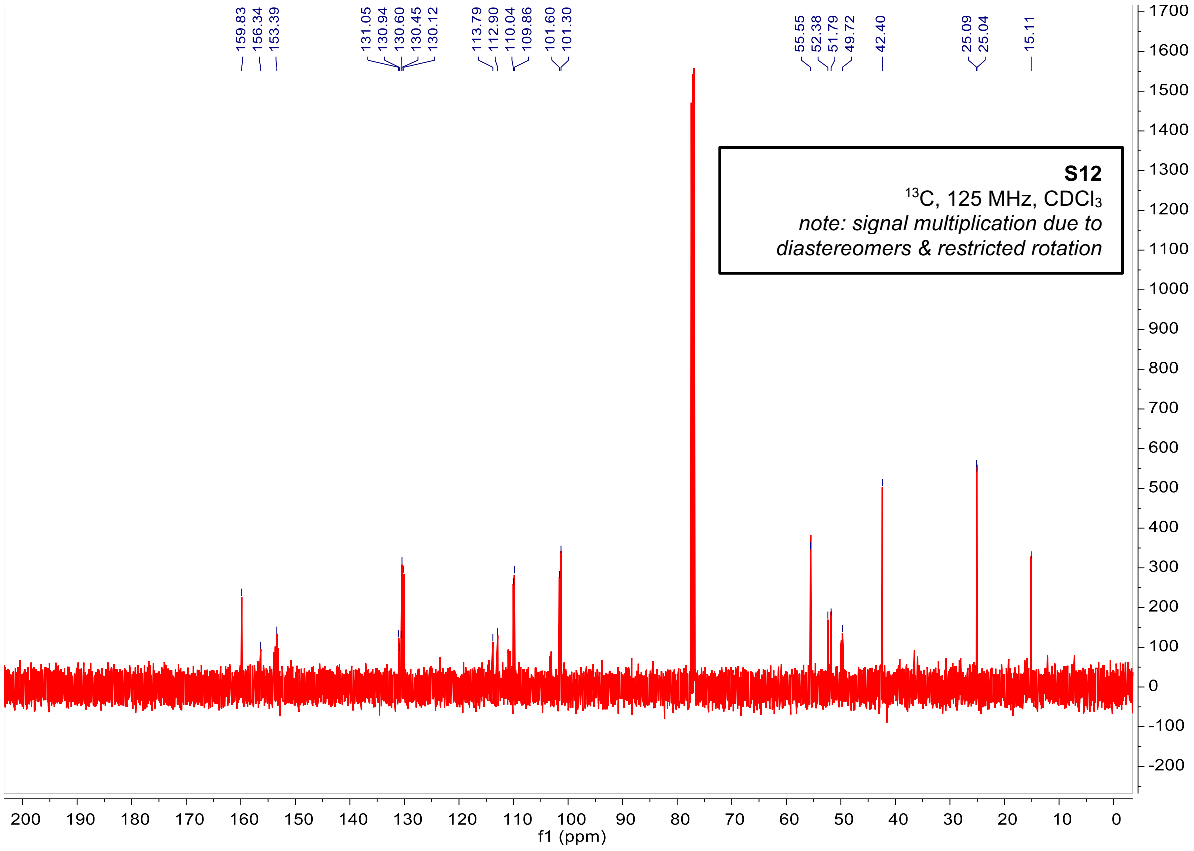
S12
^{13}C, 125 MHz, $CDCl_3$
note: signal multiplication due to diastereomers & restricted rotation
f1 (ppm)

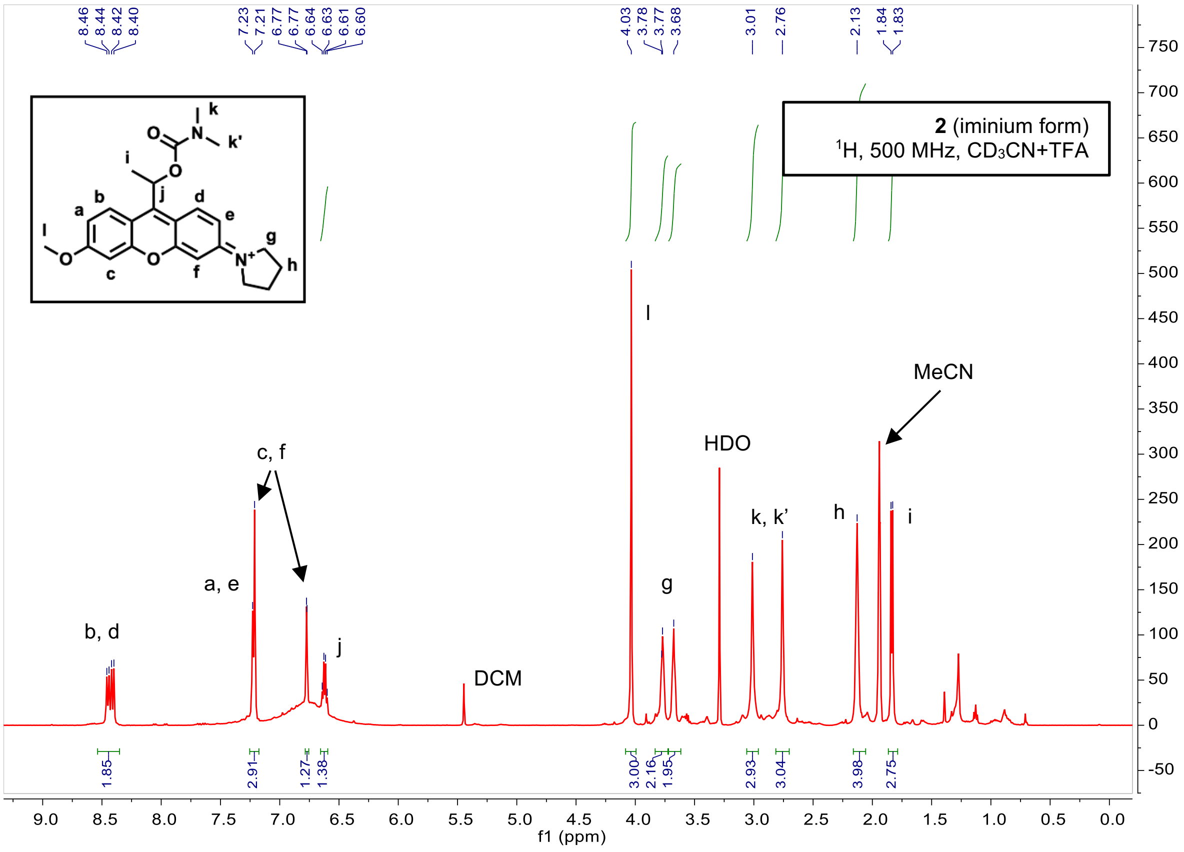
2 (iminium form)
1H, 500 MHz, CD3CN+TFA
b, d
a, e
c, f
j
DCM
l
g
HDO
k, k'
h
MeCN
i
f1 (ppm)

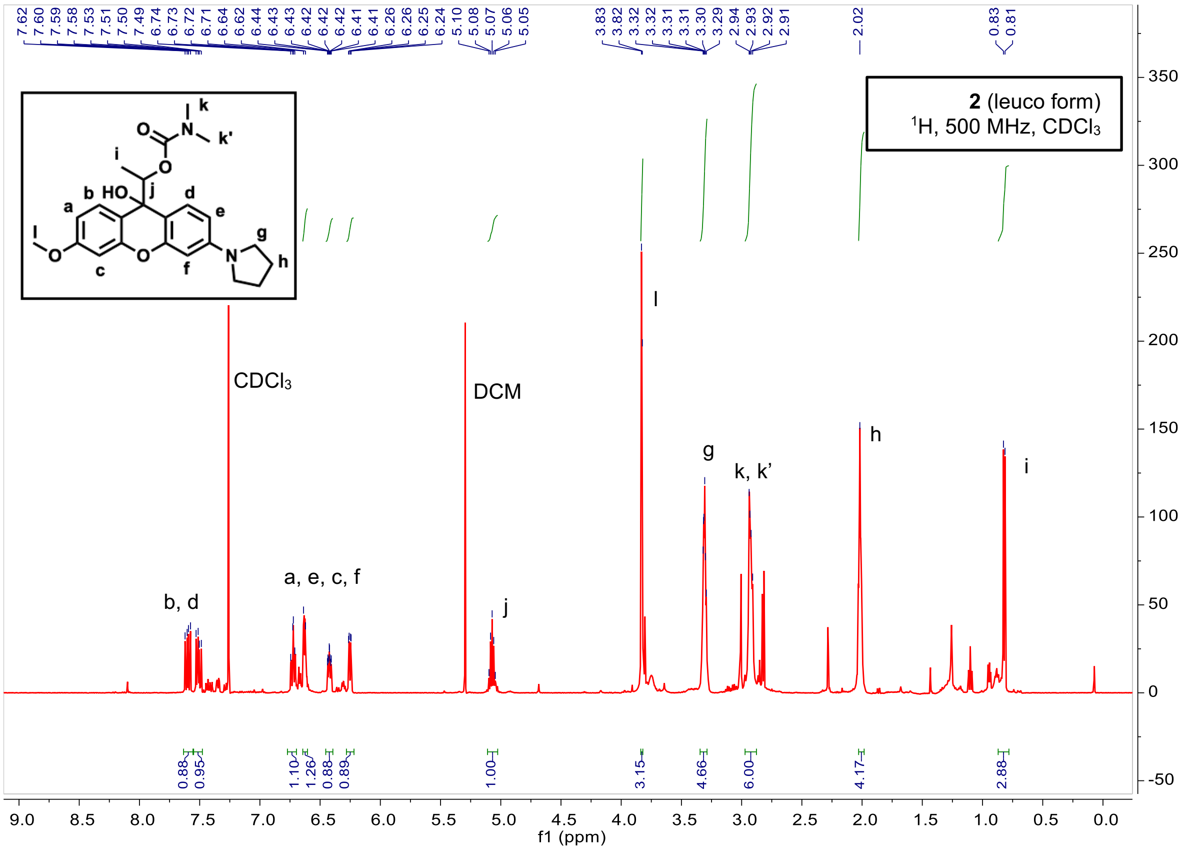
2 (leuco form)
1H, 500 MHz, CDCl3
b, d
CDCl3
a, e, c, f
DCM
j
l
g
k, k'
h
i
f1 (ppm)

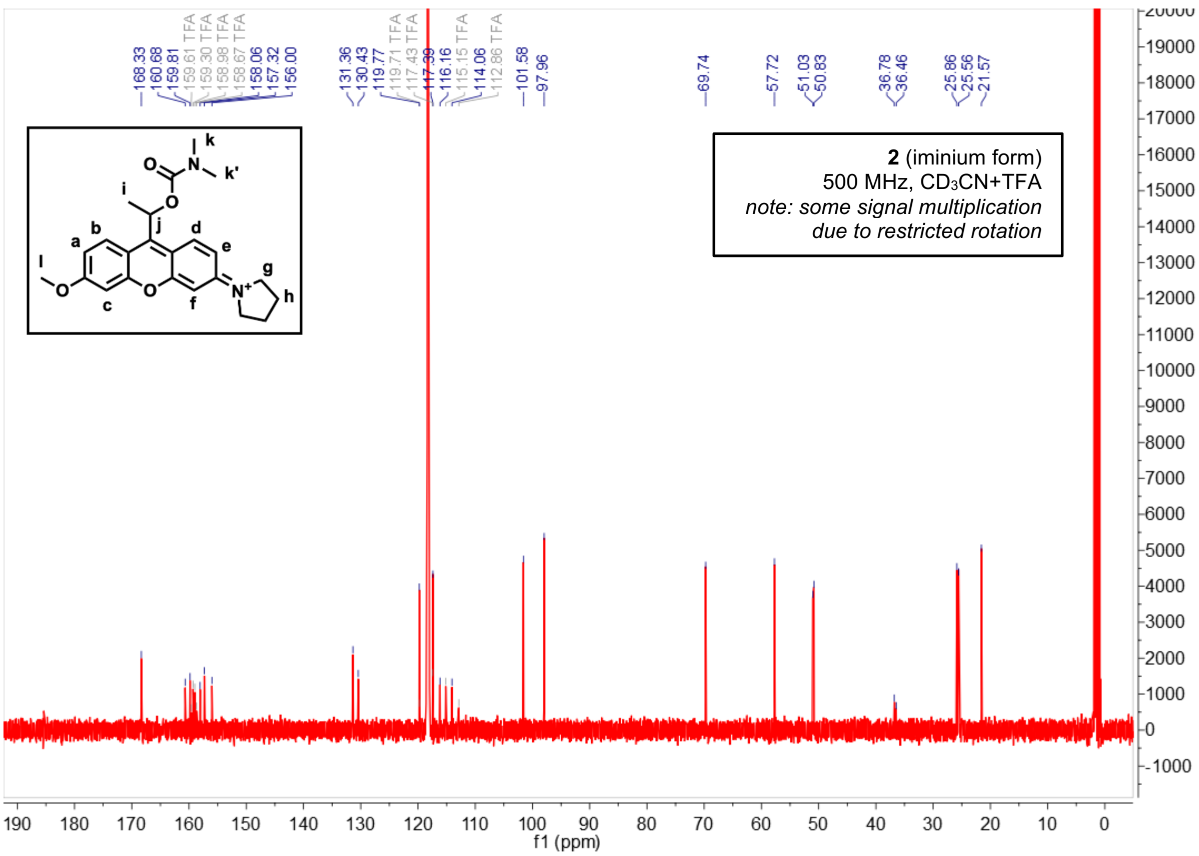
168.33
160.68
159.81
159.61 TFA
159.30 TFA
158.98 TFA
158.67 TFA
158.06
157.32
156.00
131.36
130.43
119.77
119.71 TFA
117.43 TFA
117.39
116.16
115.15 TFA
114.06
112.86 TFA
101.58
97.96
69.74
57.72
51.03
50.83
36.78
36.46
25.86
25.56
21.57
2 (iminium form)
500 MHz, CD_3CN+TFA
note: some signal multiplication due to restricted rotation
f1 (ppm)

2
$[M-H_2O+H]^+$: 395

Chromatogram

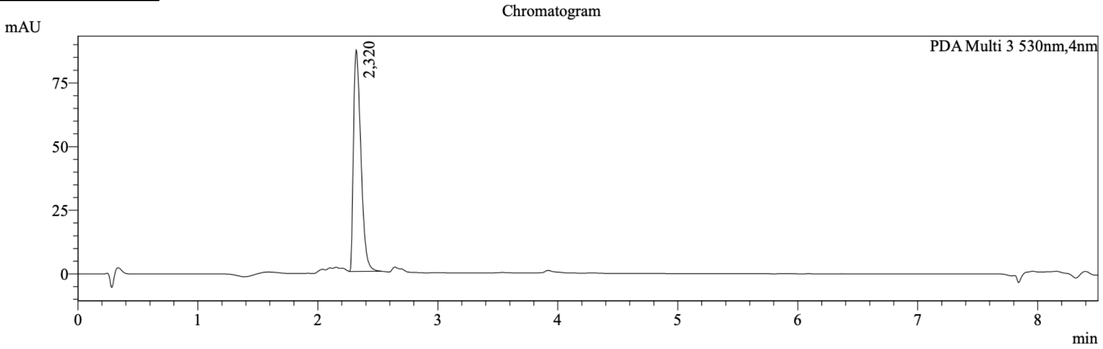

Peak Table

PDA Ch3 530nm

Peak#	Ret. Time	Peak Start	Peak End	Area	Height	Area/Height
1	2,320	2,261	2,528	373562	87121	4,288
Total				373562	87121	

Contour

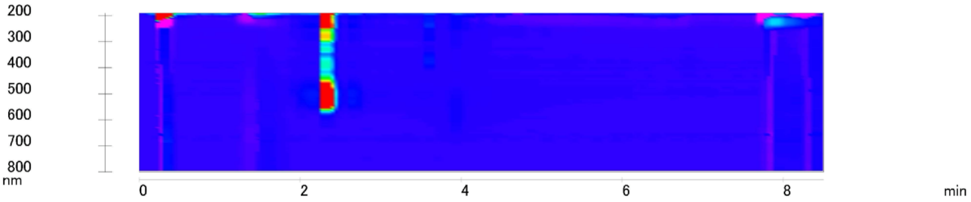

MS Chromatogram

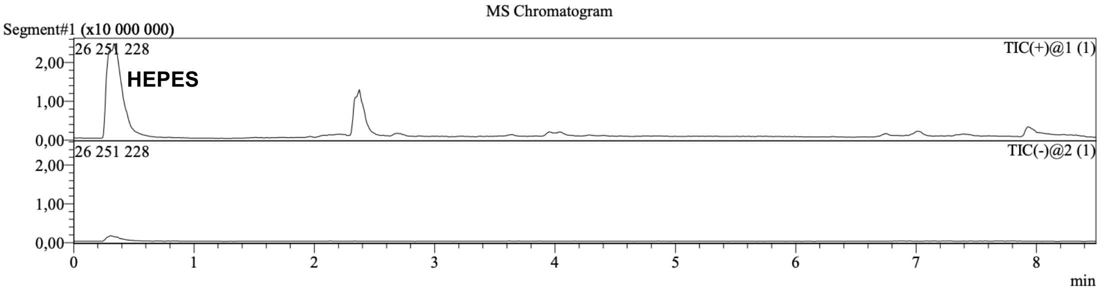

MS Spectrum

Line#:1 R.Time:----(Scan#:----)
MassPeaks:563
Spectrum Mode:Averaged 2,287-2,507(687-753) Base Peak:395(4135635)
BG Mode:Averaged 1,180-1,713(355-515) Segment 1 - Event 1

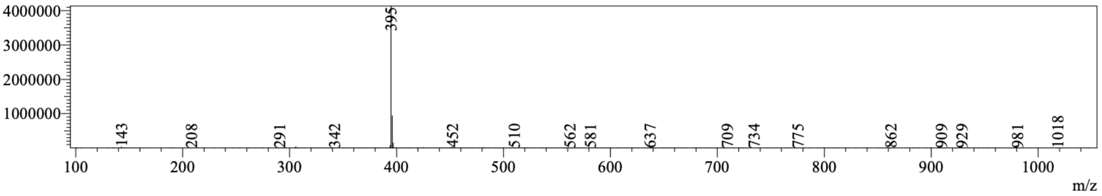

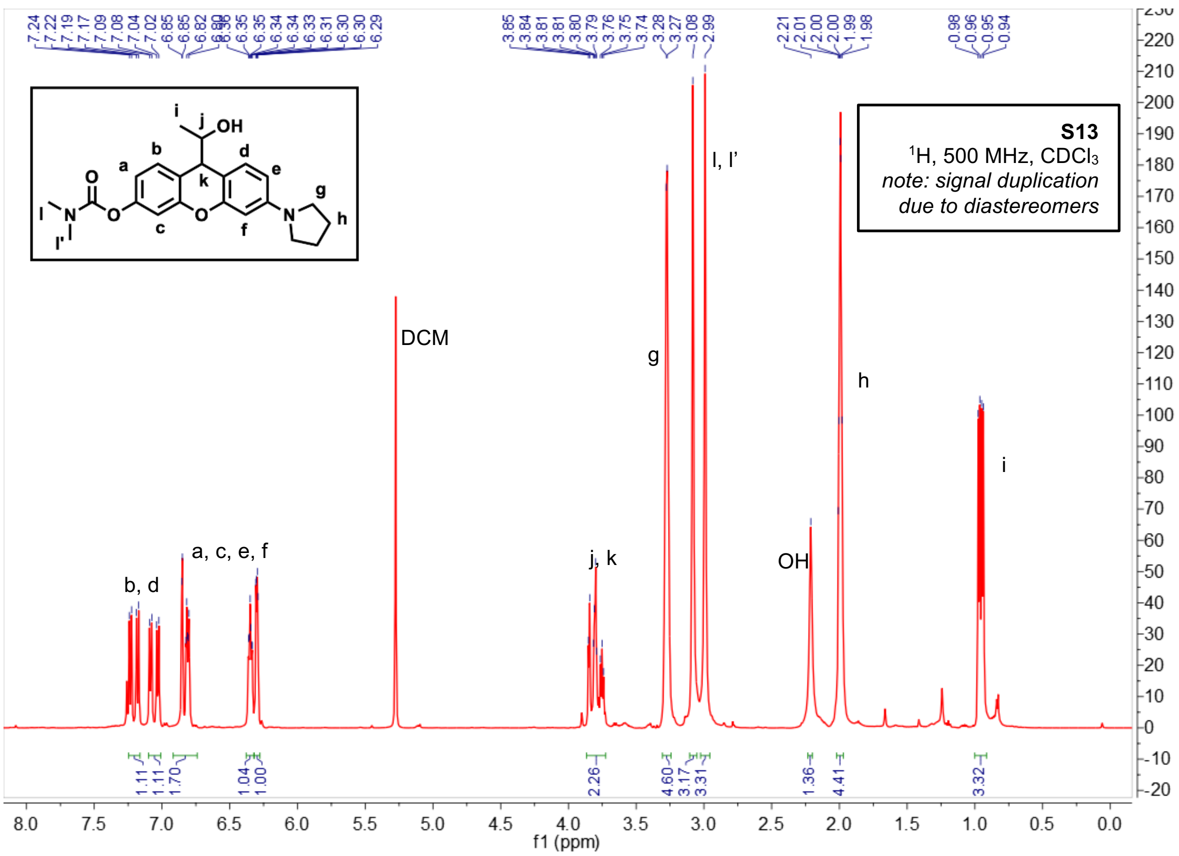
S13
^{1}H, 500 MHz, $CDCl_3$
note: signal duplication due to diastereomers
DCM
g
l, l'
h
i
OH
j, k
a, c, e, f
b, d
f1 (ppm)

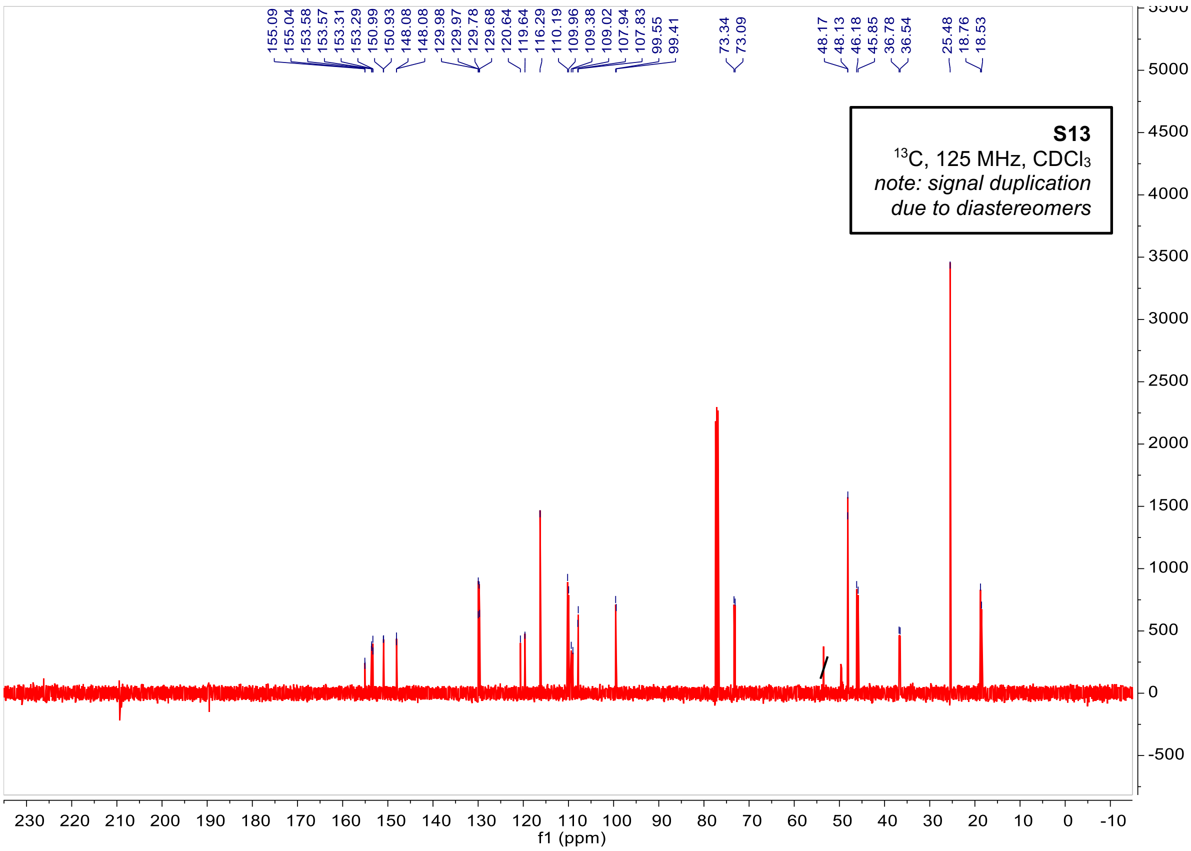
S13
^{13}C, 125 MHz, $CDCl_3$
note: signal duplication due to diastereomers
f1 (ppm)

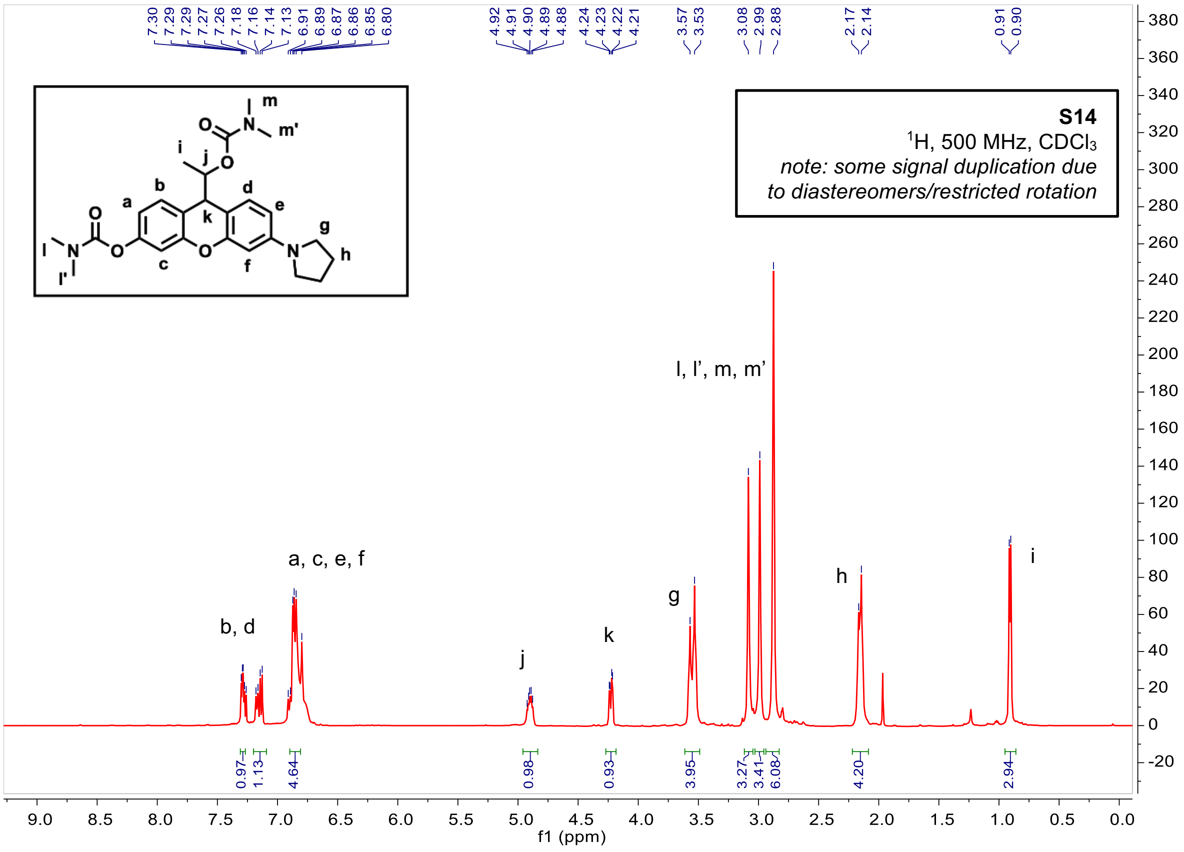
S14
1H, 500 MHz, CDCl3
note: some signal duplication due to diastereomers/restricted rotation
a, c, e, f
b, d
j
k
g
l, l', m, m'
h
i
f1 (ppm)

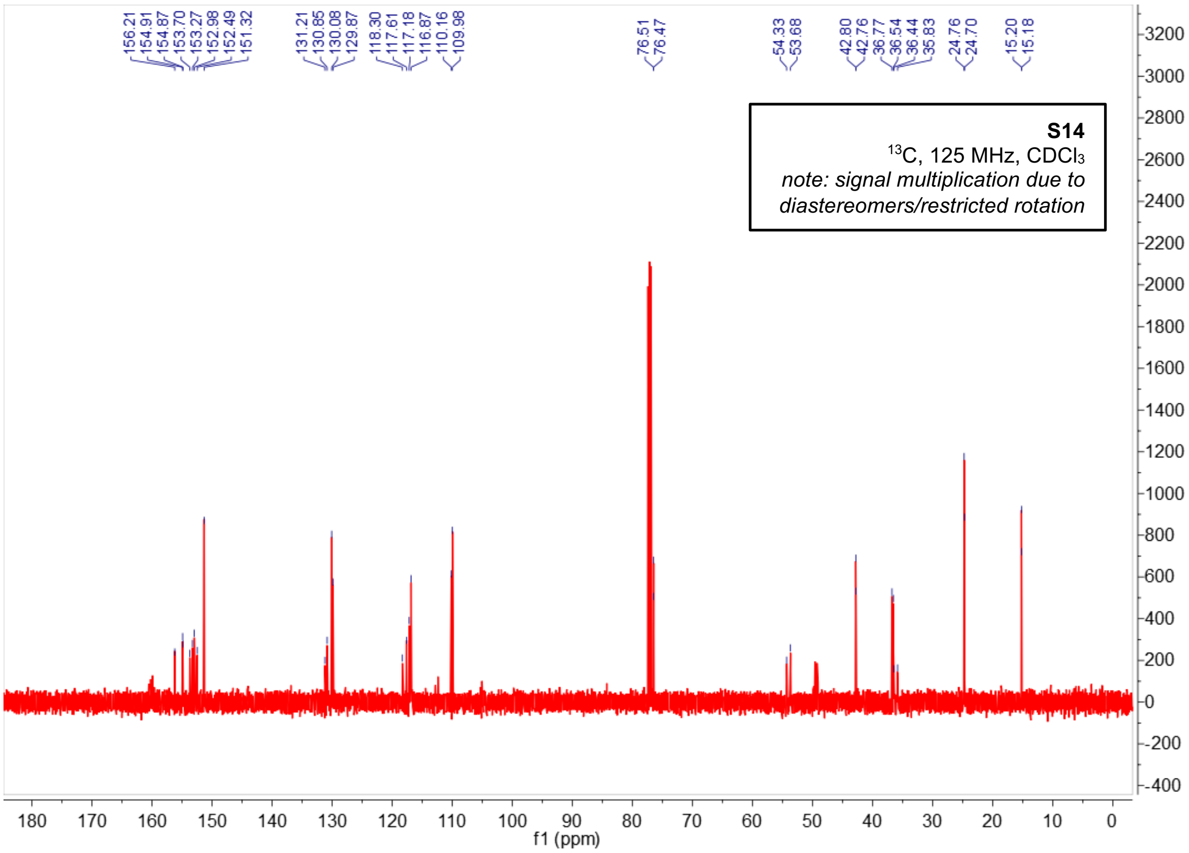
S14
13C, 125 MHz, CDCl3
note: signal multiplication due to diastereomers/restricted rotation
f1 (ppm)

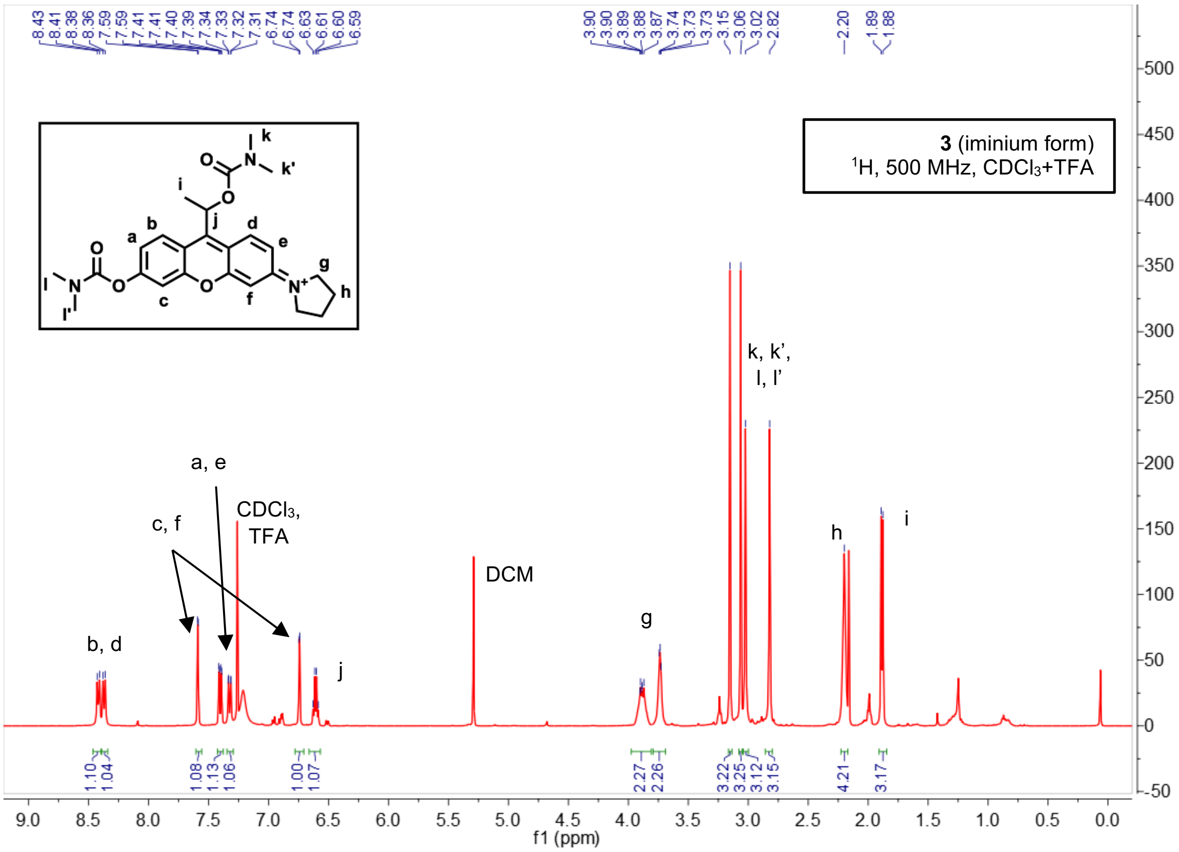

3 (iminium form)
¹H, 500 MHz, CDCl3+TFA
b, d
c, f
a, e
CDCl3,
TFA
DCM
j
g
k, k',
l, l'
h
i
f1 (ppm)

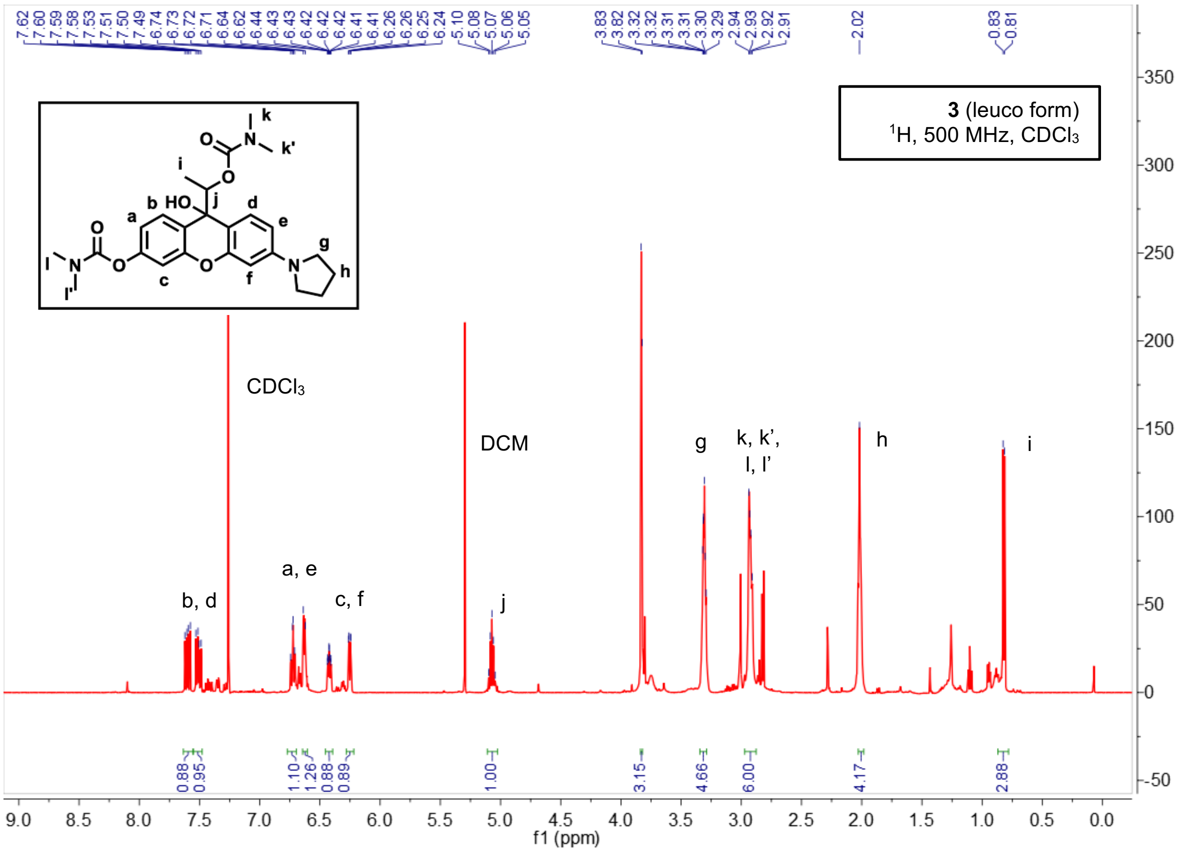

3 (leuco form)
¹H, 500 MHz, CDCl3
CDCl3
DCM
b, d
a, e
c, f
j
g
k, k',
l, l'
h
i
f1 (ppm)

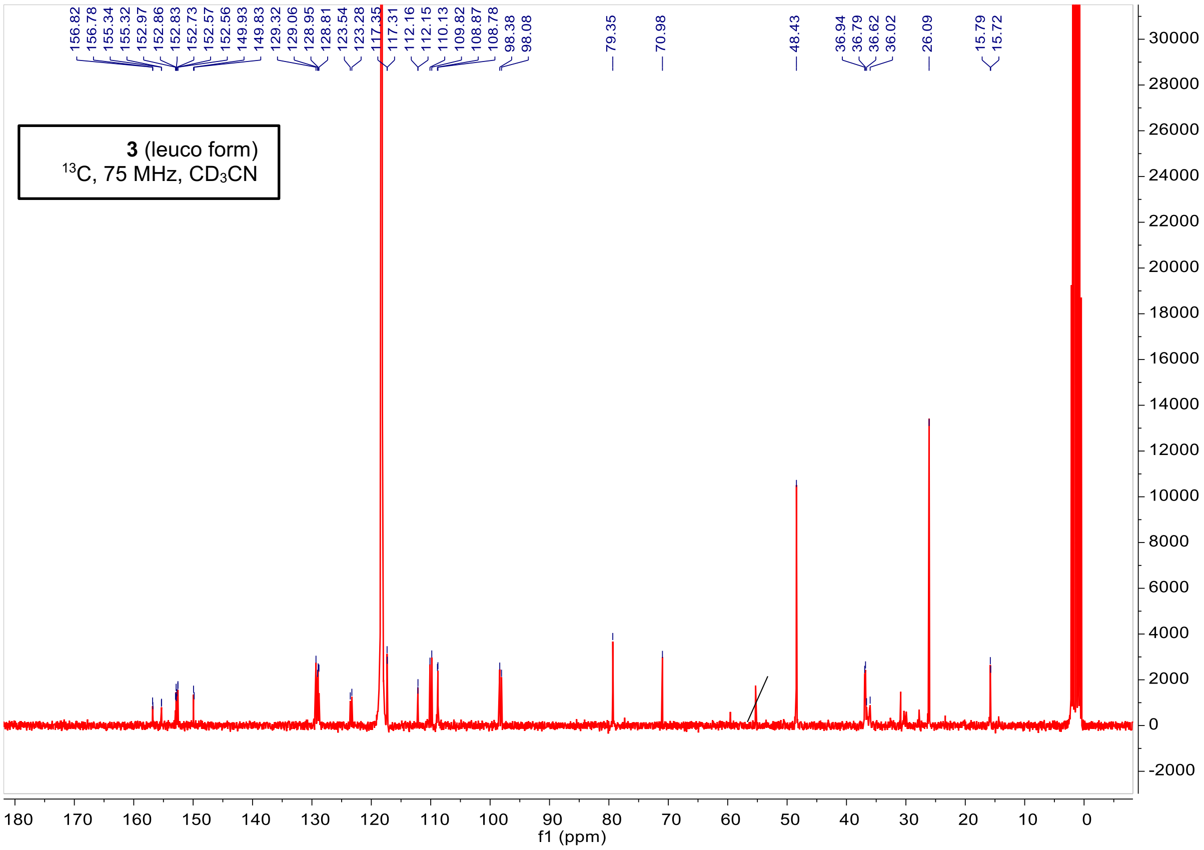
3 (leuco form)
13C, 75 MHz, CD3CN
f1 (ppm)

3
$[M-H_2O+H]^+$: 452

Chromatogram

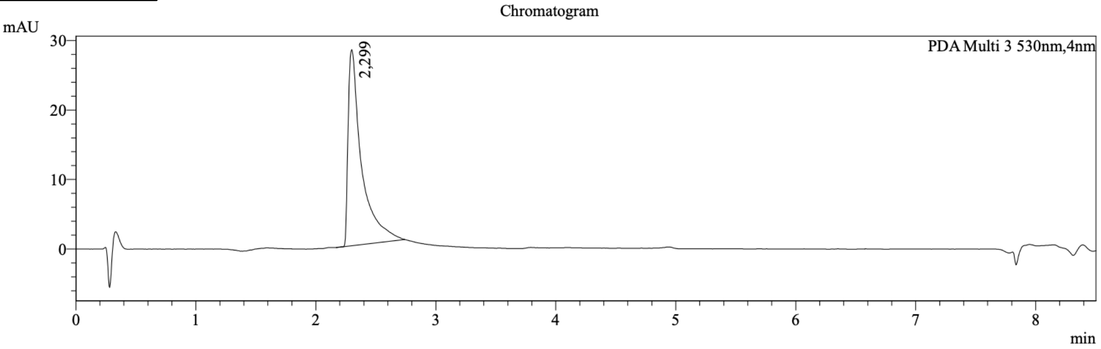

Peak Table

PDA Ch3 530nm

Peak#	Ret. Time	Peak Start	Peak End	Area	Height	Area/Height
1	2,299	2,171	2,741	219791	28218	7,789
Total				219791	28218	

Contour

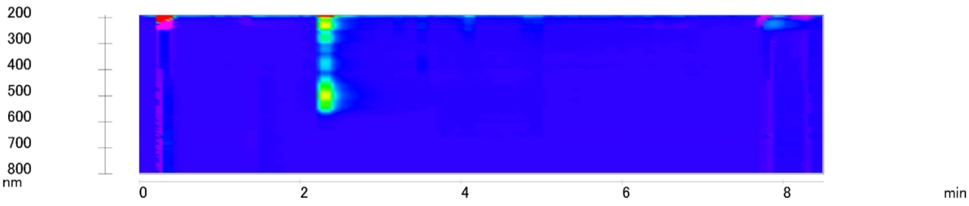

MS Chromatogram

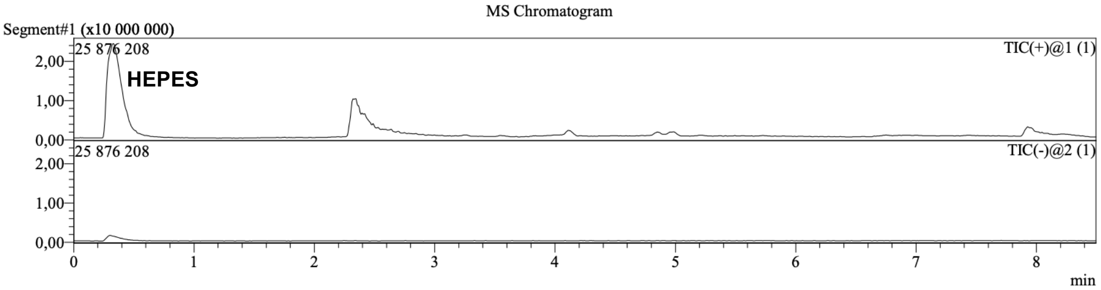

MS Spectrum

Line#:1 R.Time:----(Scan#:----)
MassPeaks:527
Spectrum Mode:Averaged 2,260-2,540(679-763) Base Peak:452(3470151)
BG Mode:Averaged 1,313-1,800(395-541) Segment 1 - Event 1

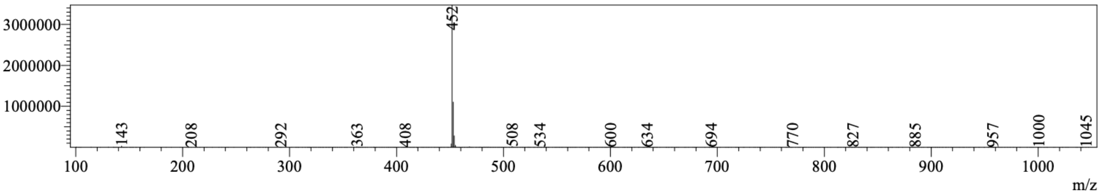

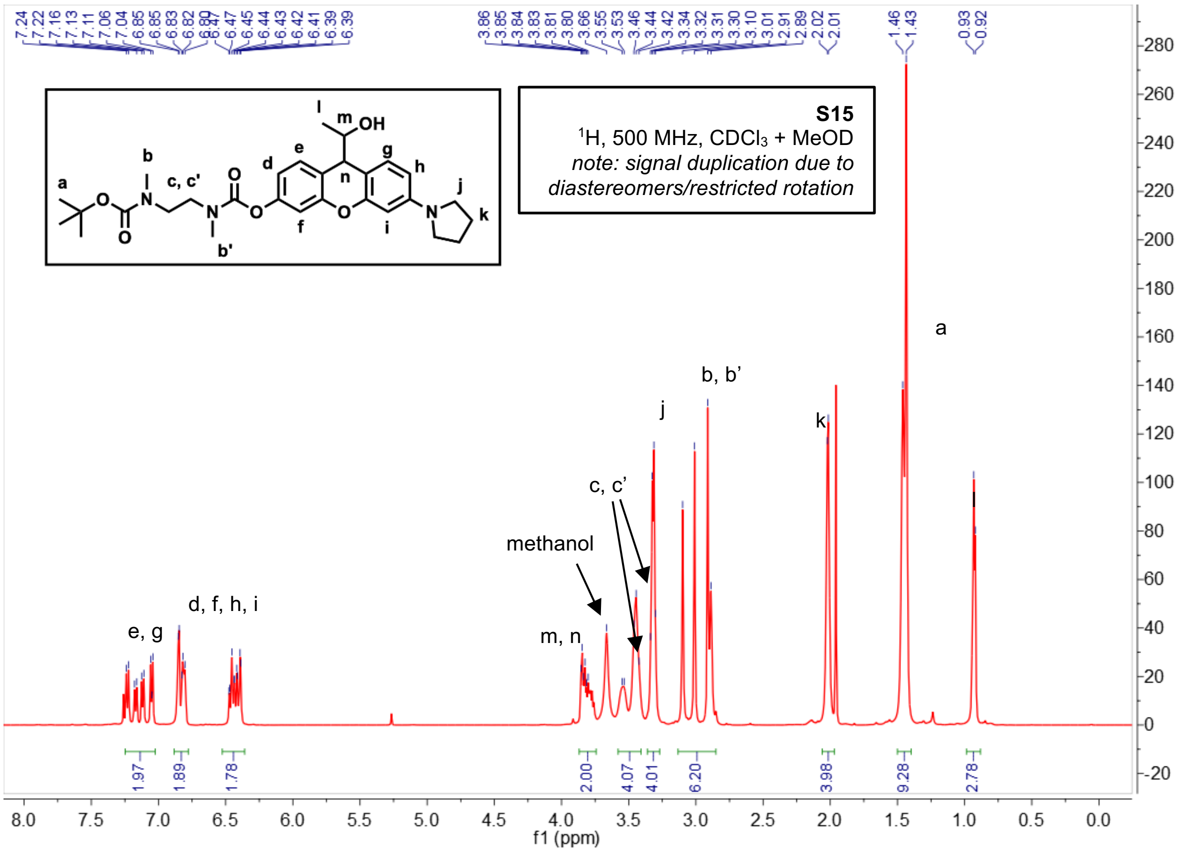
7.24 7.22 7.16 7.13 7.11 7.06 7.04 6.85 6.85 6.83 6.82 6.80 6.47 6.47 6.45 6.44 6.43 6.42 6.41 6.39 6.39
3.86 3.85 3.84 3.83 3.81 3.80 3.66 3.55 3.53 3.46 3.44 3.42 3.34 3.32 3.31 3.30 3.10 3.01 2.91 2.89 2.02 2.01
1.46 1.43
0.93 0.92
S15
^{1}H, 500 MHz, $CDCl_3$ + MeOD
note: signal duplication due to diastereomers/restricted rotation
a
b, b'
j
k
c, c'
methanol
d, f, h, i
e, g
m, n
1.97 1.89 1.78 2.00 4.07 4.01 6.20 3.98 9.28 2.78
f1 (ppm)

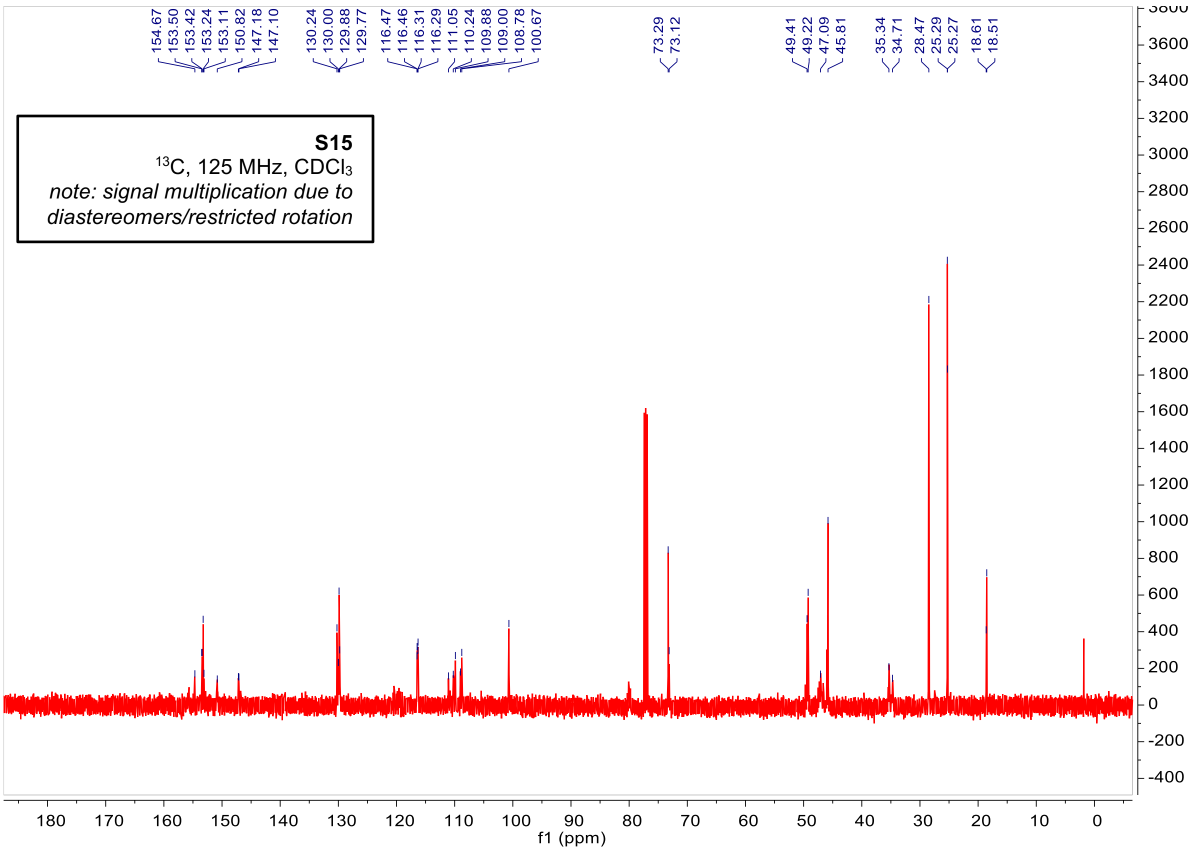
154.67 153.50 153.42 153.24 153.11 150.82 147.18 147.10
130.24 130.00 129.88 129.77
116.47 116.46 116.31 116.29 111.05 110.24 109.88 109.00 108.78 100.67
73.29 73.12
49.41 49.22 47.09 45.81
35.34 34.71 28.47 25.29 25.27 18.61 18.51
S15
^{13}C, 125 MHz, $CDCl_3$
note: signal multiplication due to diastereomers/restricted rotation
f1 (ppm)

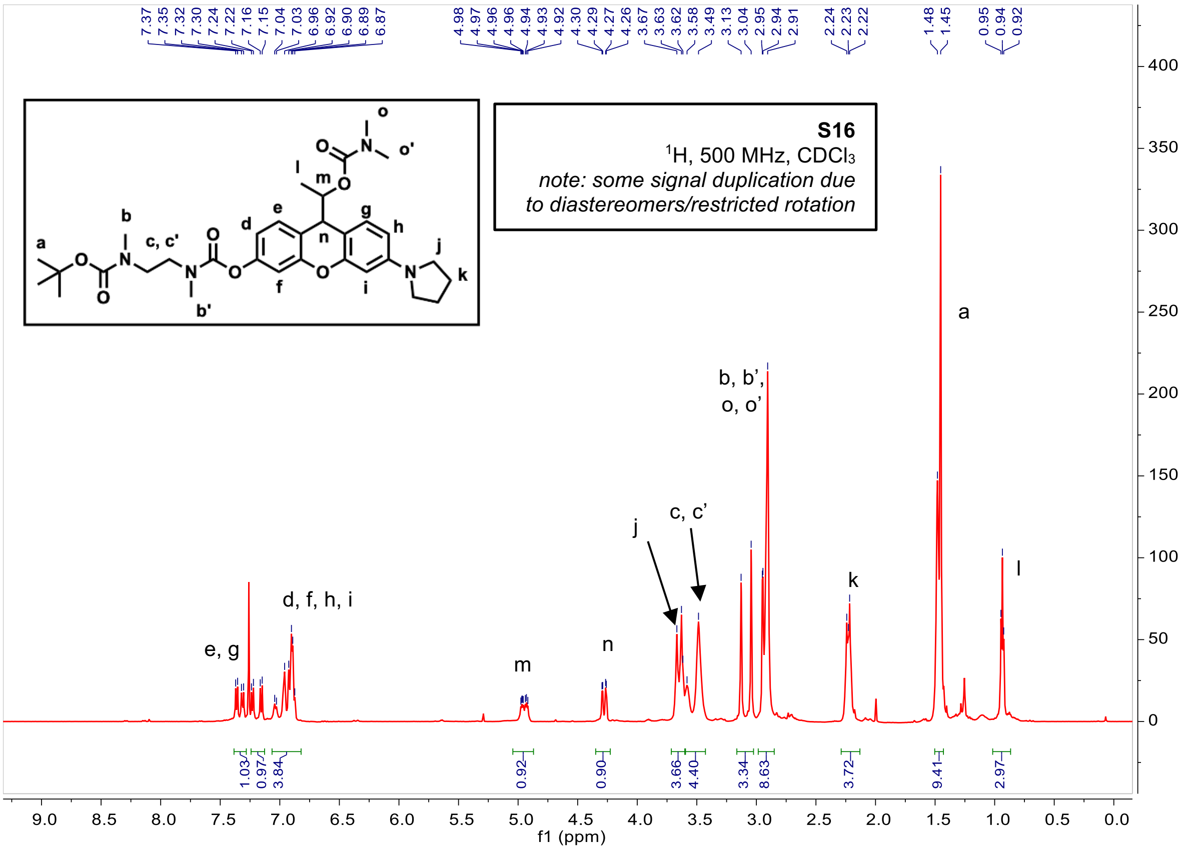
S16
¹H, 500 MHz, CDCl3
note: some signal duplication due
to diastereomers/restricted rotation
a
b, b', o, o'
c, c'
j
k
l
n
m
d, f, h, i
e, g
f1 (ppm)

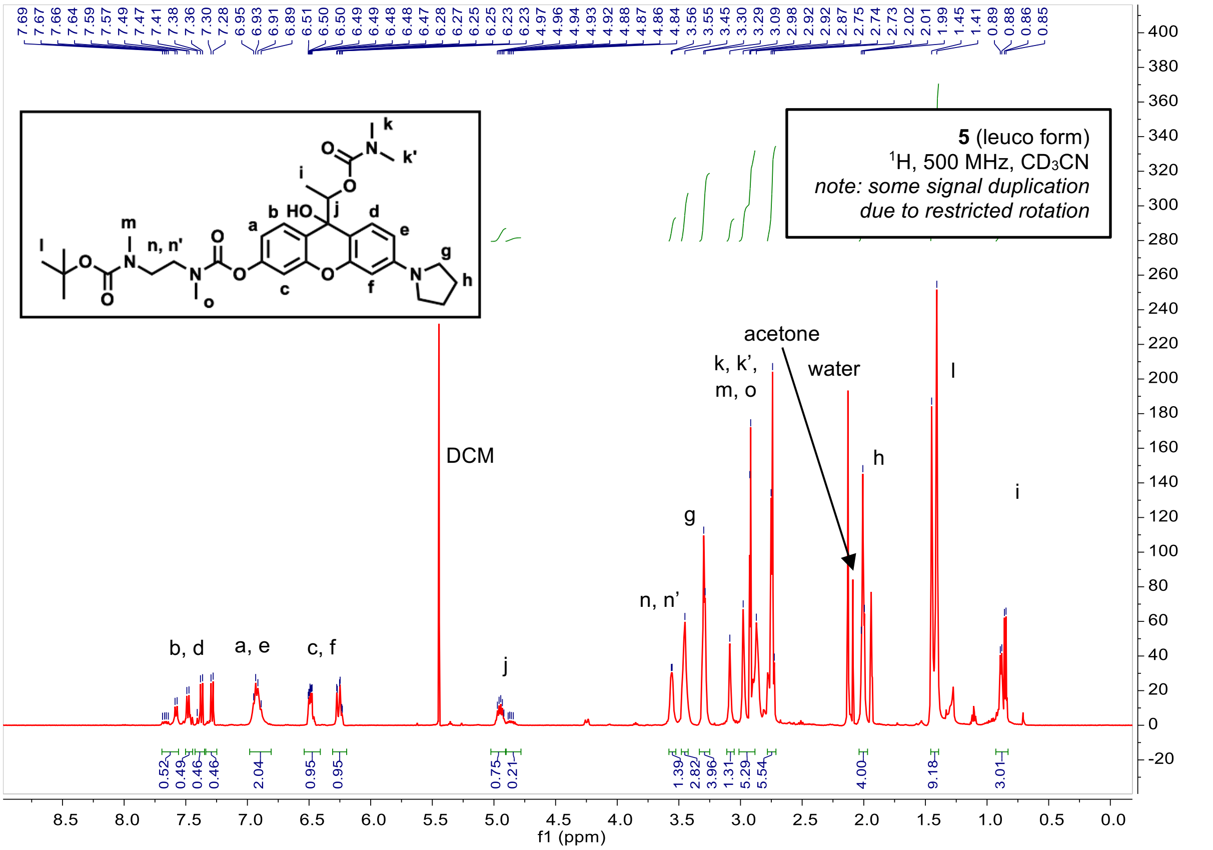
5 (leuco form)
1H, 500 MHz, CD3CN
note: some signal duplication due to restricted rotation
DCM
acetone
water
k, k', m, o
n, n'
g
h
l
i
j
b, d
a, e
c, f
f1 (ppm)

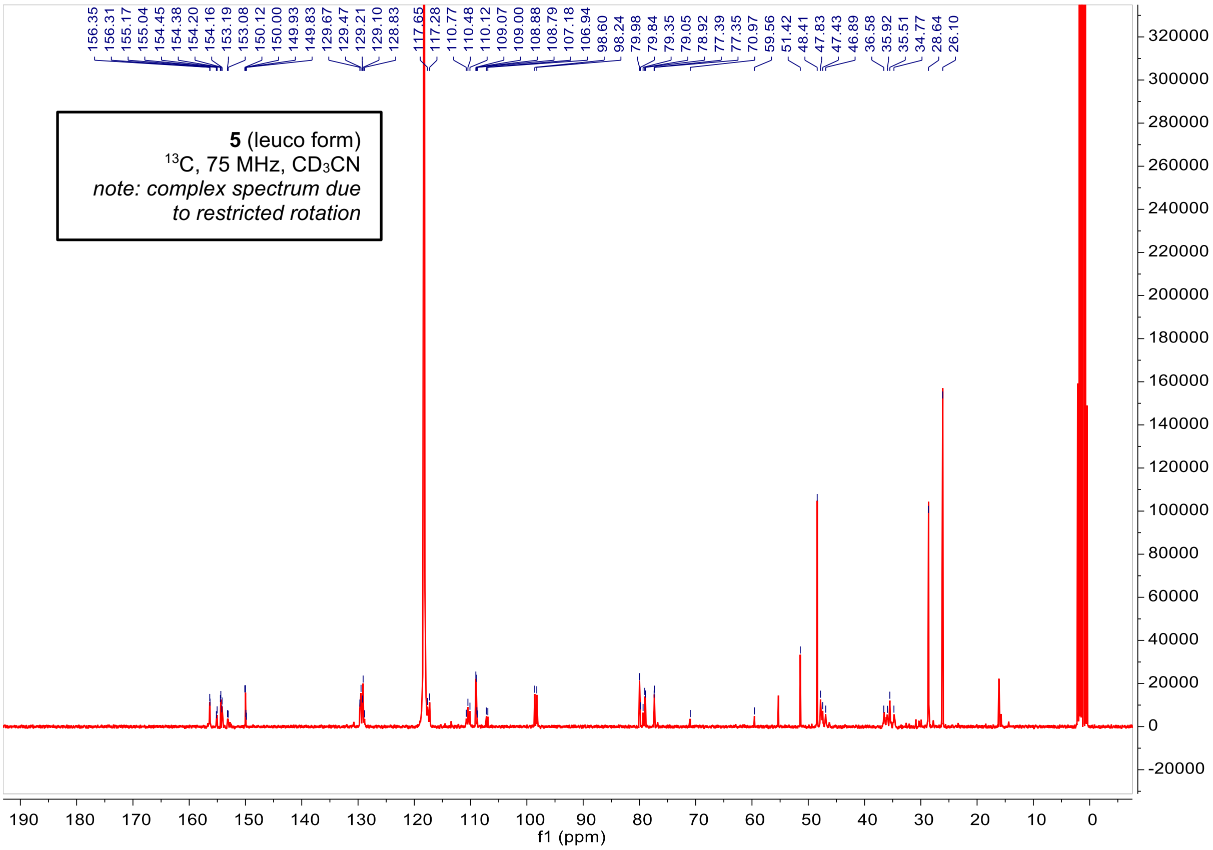
5 (leuco form)
13C, 75 MHz, CD3CN
note: complex spectrum due to restricted rotation
f1 (ppm)

5
[$M-H_2O+H]^+$: 595

Chromatogram

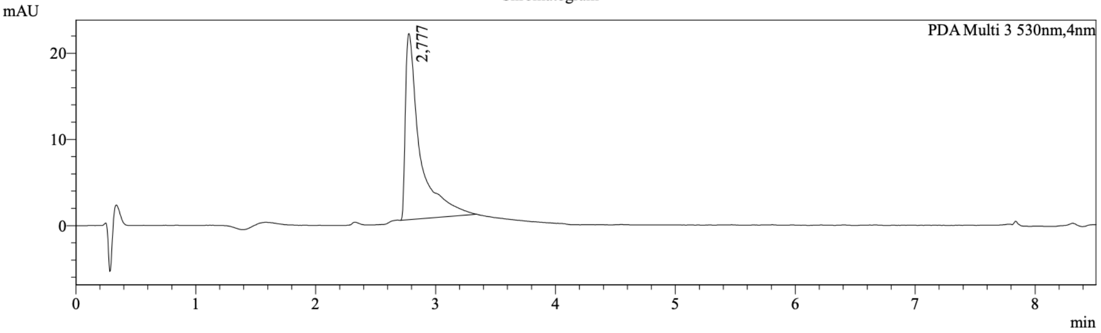

Peak Table

PDA Ch3 530nm

Peak#	Ret. Time	Peak Start	Peak End	Area	Height	Area/Height
1	2,777	2,709	3,344	182055	21604	8,427
Total				182055	21604	

Contour

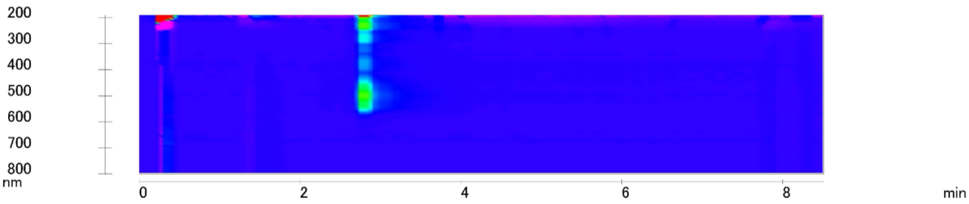

MS Chromatogram

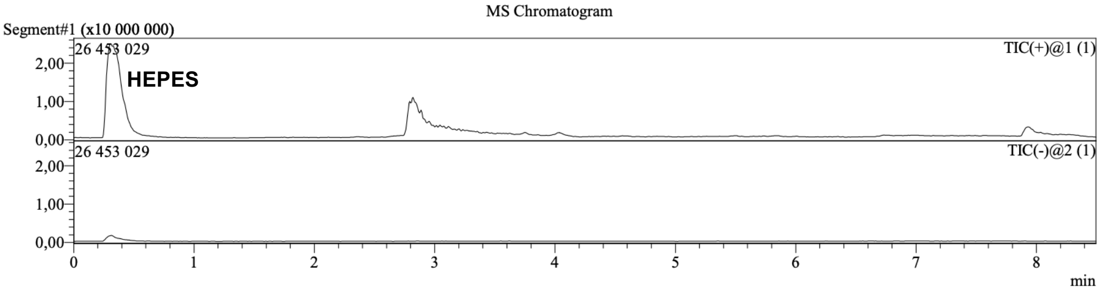

MS Spectrum

Line#:1 R.Time:----(Scan#:----)
MassPeaks:676
Spectrum Mode:Averaged 2,727-2,980(819-895) Base Peak:595(3778359)
BG Mode:Averaged 1,127-1,953(339-587) Segment 1 - Event 1

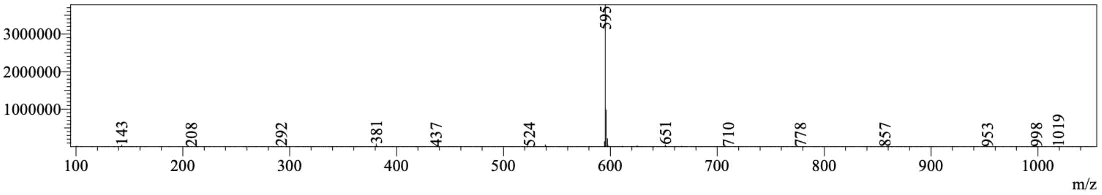

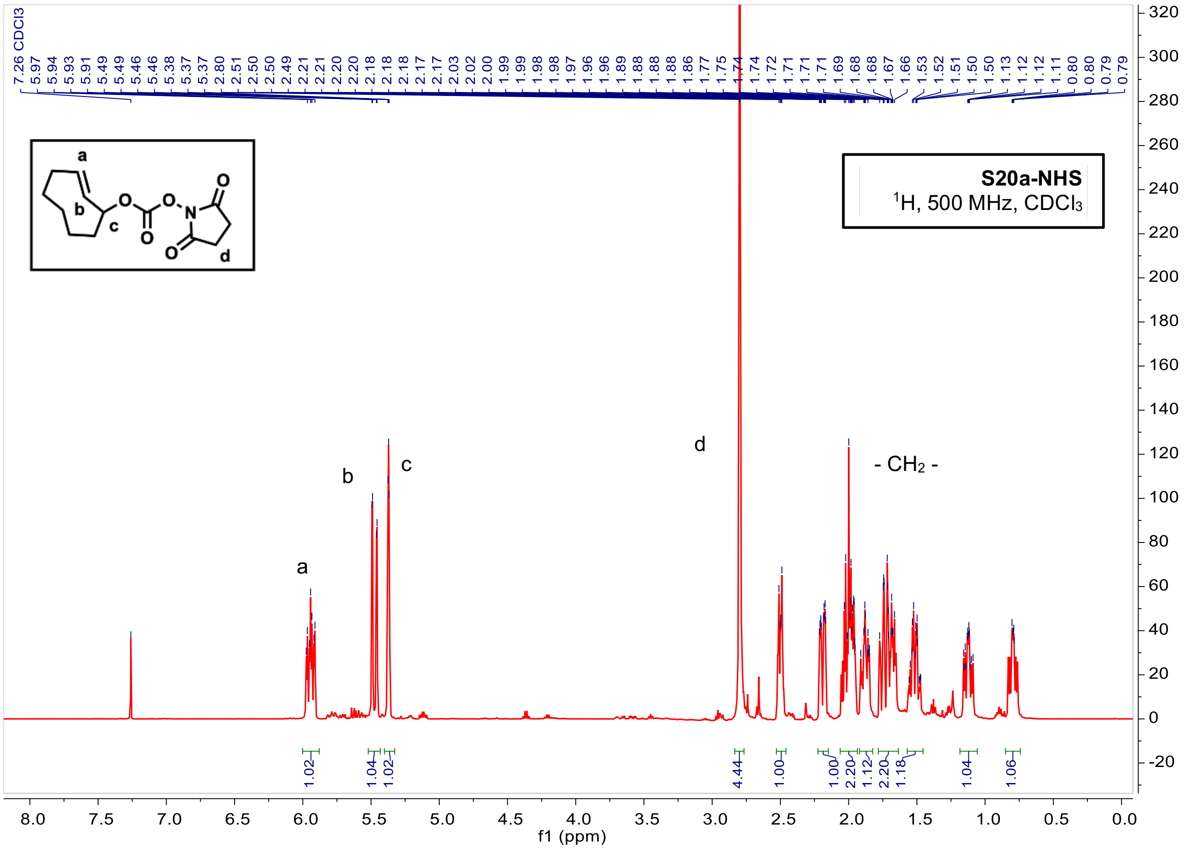
S20a-NHS
1H, 500 MHz, CDCl3
a
b
c
d
- CH2 -
f1 (ppm)

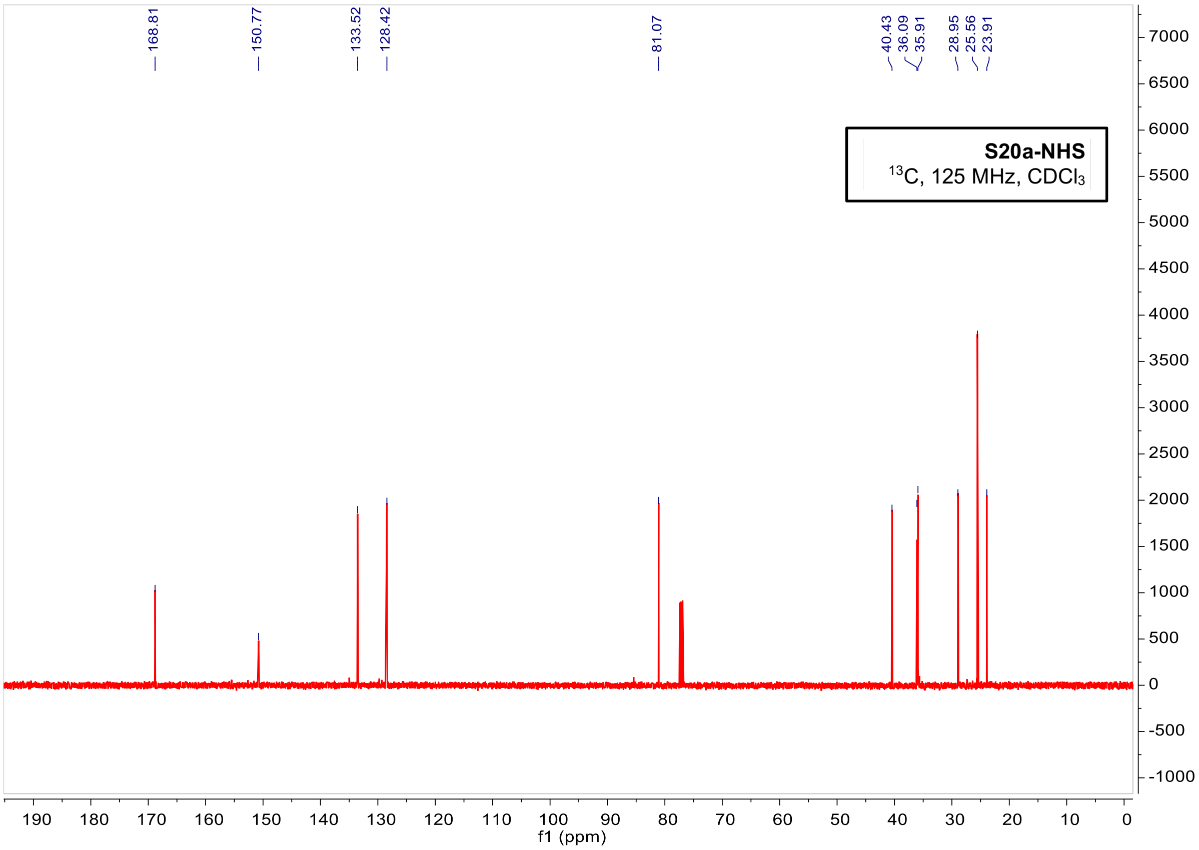
S20a-NHS
13C, 125 MHz, CDCl3
168.81
150.77
133.52
128.42
81.07
40.43
36.09
35.91
28.95
25.56
23.91
f1 (ppm)

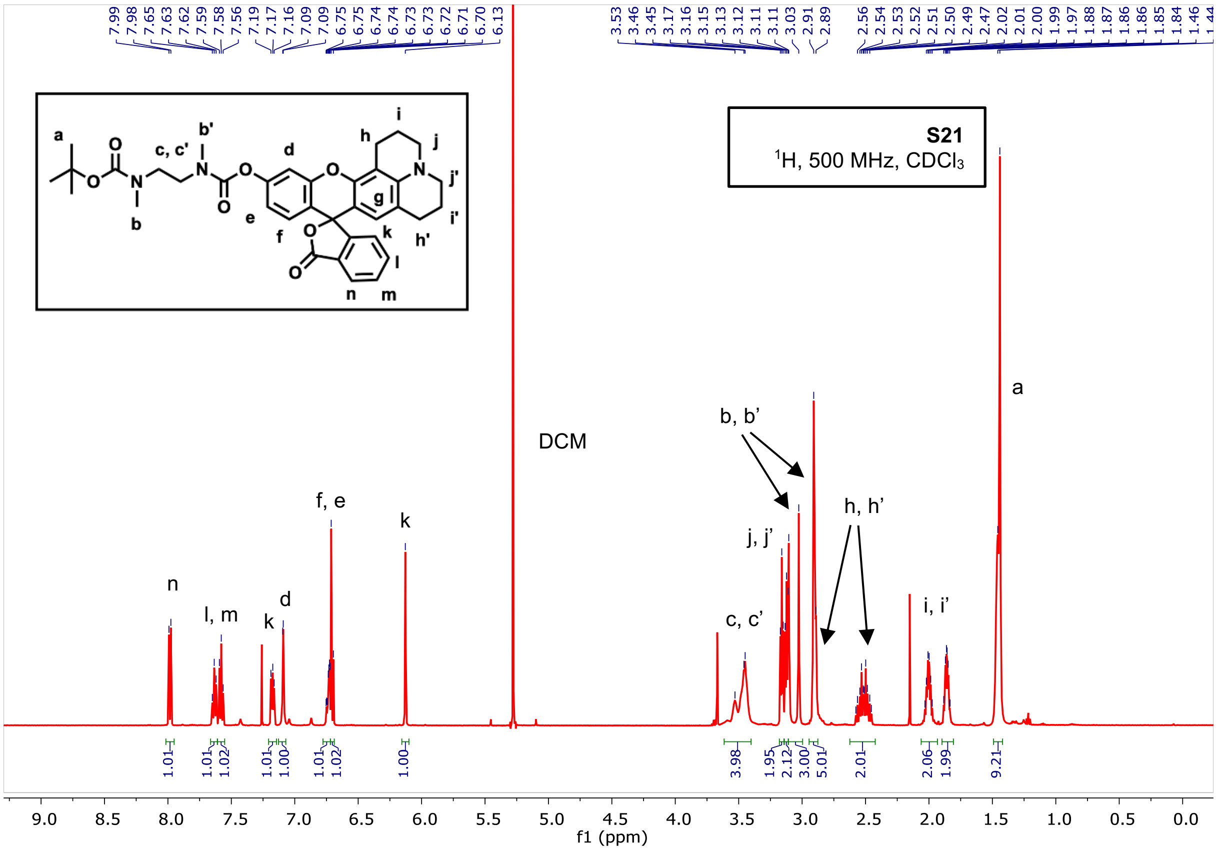
S21
1H, 500 MHz, CDCl3
DCM
a
b, b'
c, c'
j, j'
h, h'
i, i'
n
l, m
k
d
f, e
f1 (ppm)

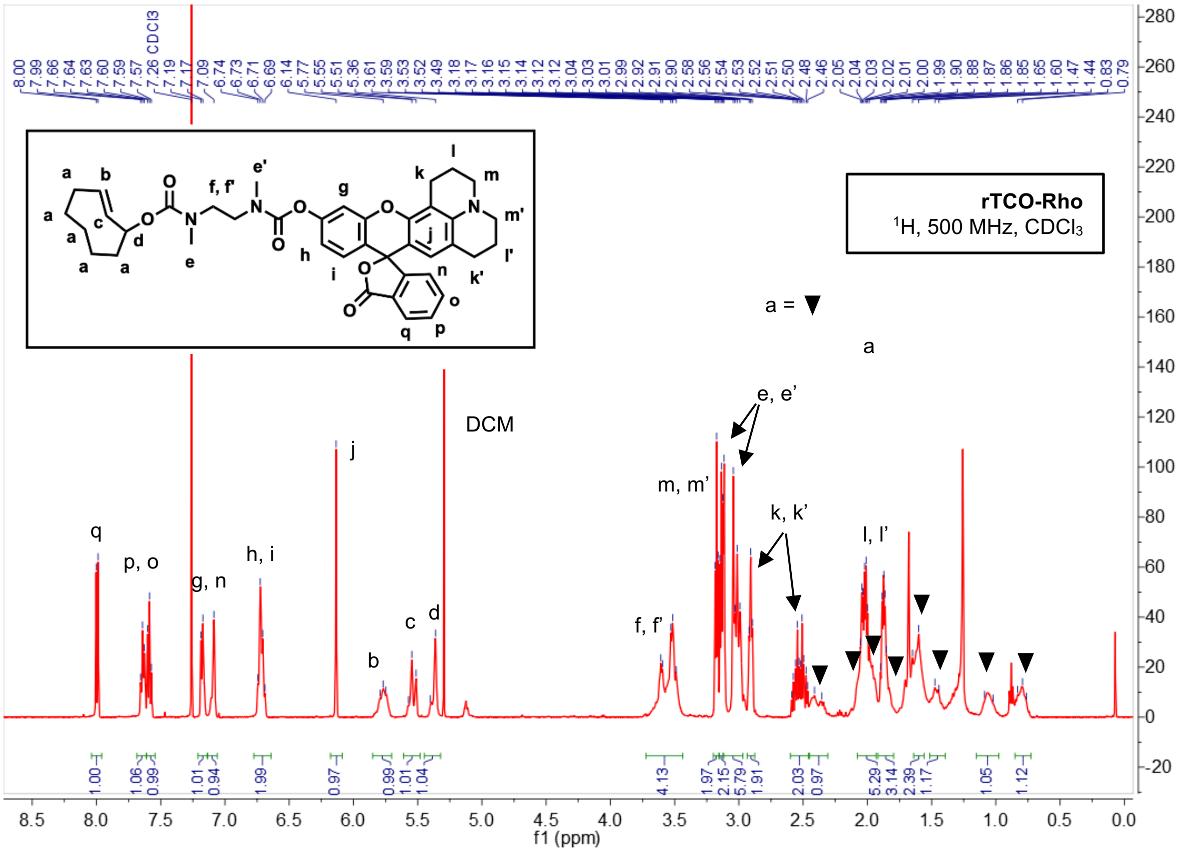
rTCO-Rho
¹H, 500 MHz, CDCl₃
DCM
a = ▼
f1 (ppm)

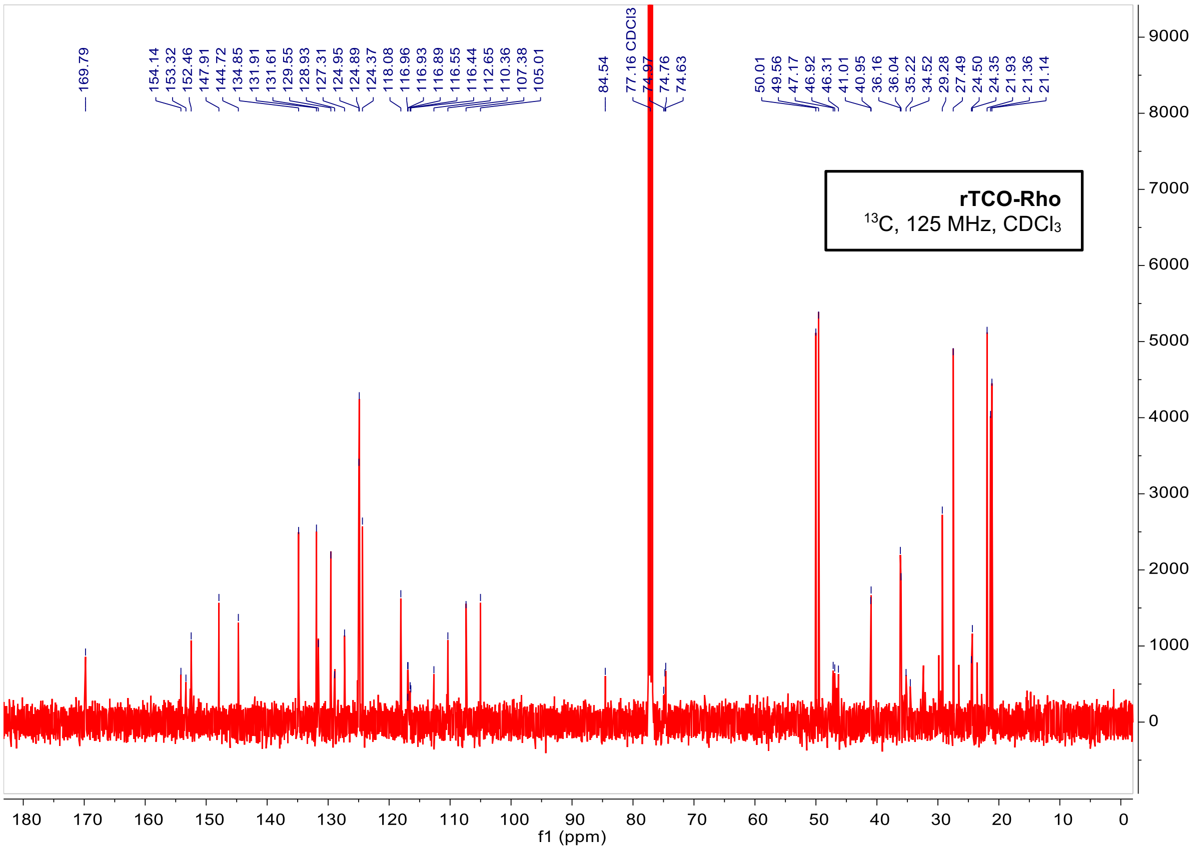
rTCO-Rho
¹³C, 125 MHz, CDCl₃
f1 (ppm)

rTCO-Rho
$[M+H]^+$: 678

Chromatogram

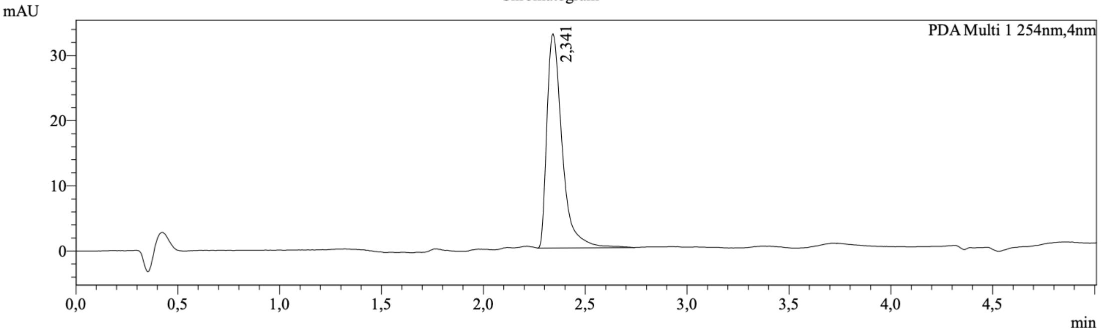

Peak Table

PDA Ch1 254nm

Peak#	Ret. Time	Peak Start	Peak End	Area	Height	Area/Height
1	2,341	2,267	2,741	170826	32899	5,192
Total				170826	32899	

Contour

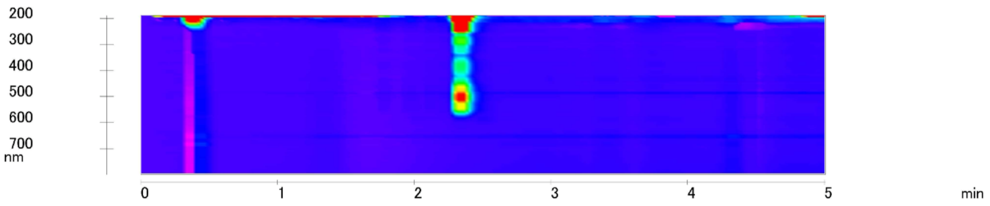

MS Chromatogram

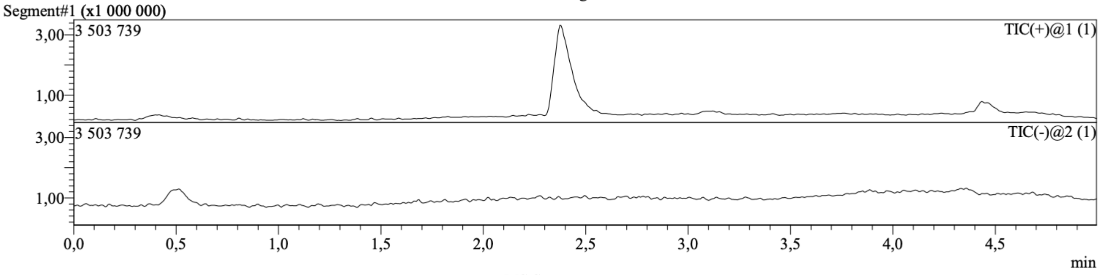

MS Spectrum

Line#:1 R.Time:----(Scan#:----)
MassPeaks:701
Spectrum Mode:Averaged 2,300-2,527(691-759) Base Peak:678(731967)
BG Mode:Averaged 1,527-1,967(459-591) Segment 1 - Event 1

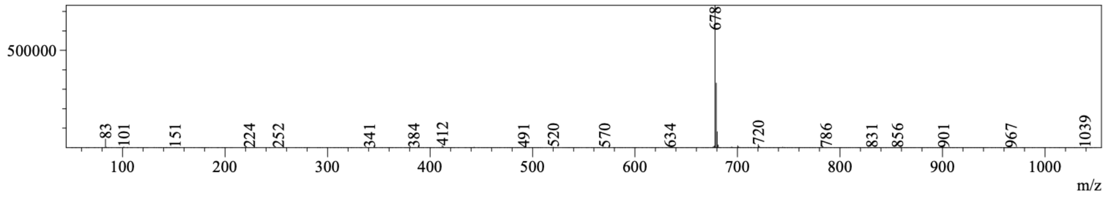

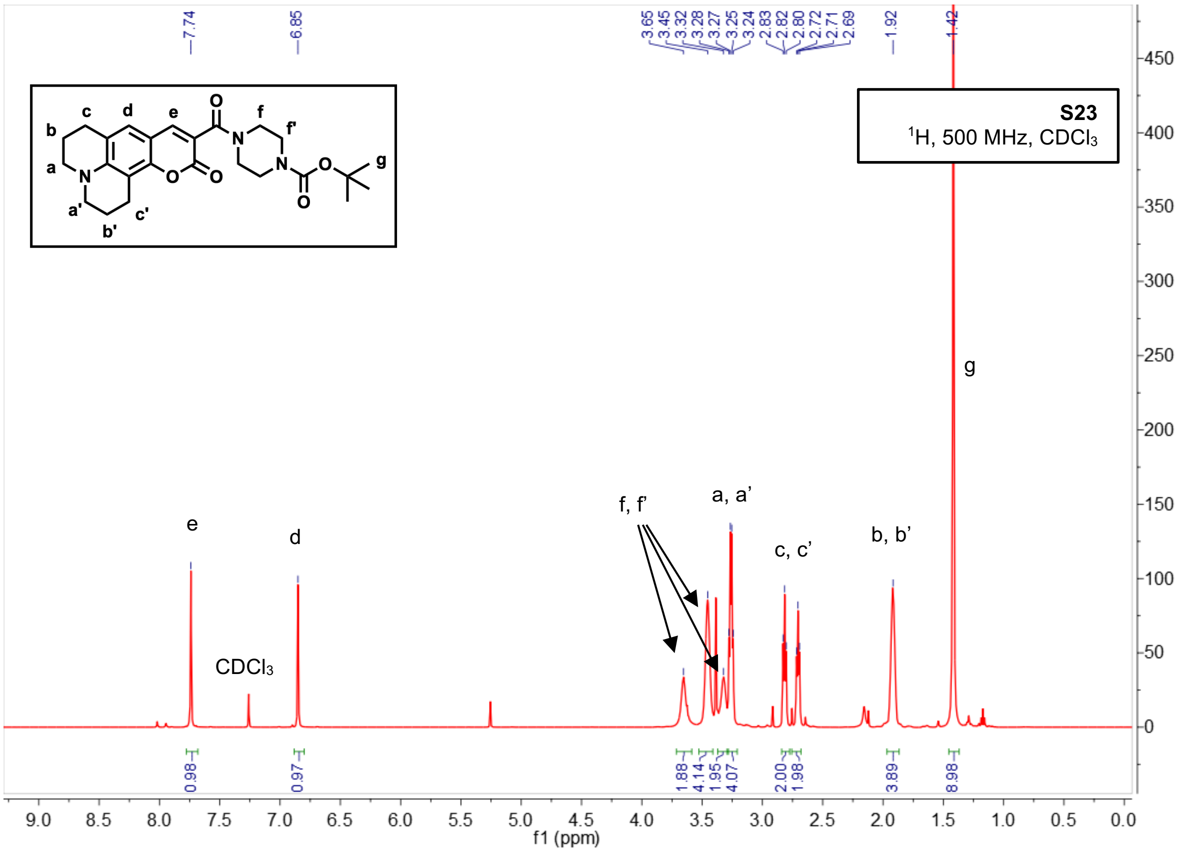

7.74
6.85
3.65
3.45
3.32
3.28
3.27
3.25
3.24
2.83
2.82
2.80
2.72
2.71
2.69
1.92
1.42
S23
^{1}H, 500 MHz, CDCl$_3$
e
d
CDCl$_3$
f, f'
a, a'
c, c'
b, b'
g
0.98
0.97
1.88
4.14
1.95
4.07
2.00
1.98
3.89
8.98
f1 (ppm)

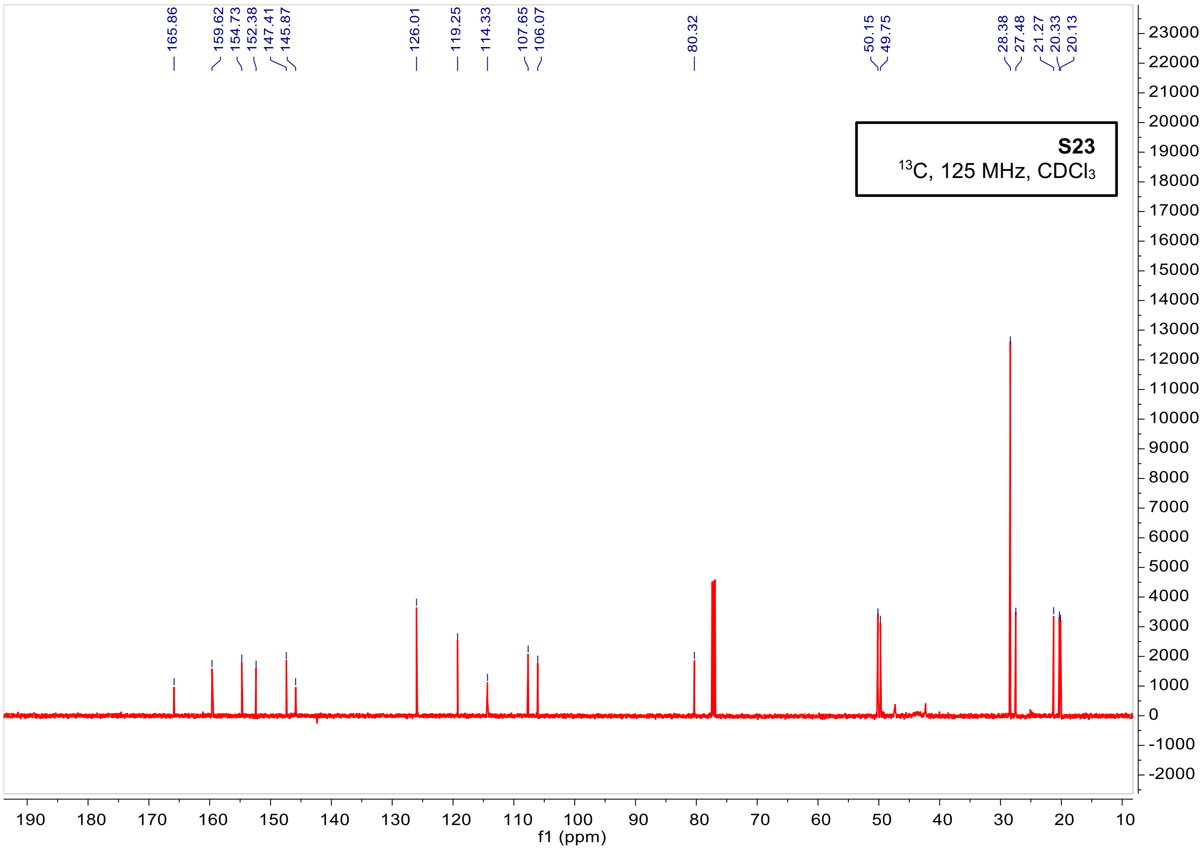

165.86
159.62
154.73
152.38
147.41
145.87
126.01
119.25
114.33
107.65
106.07
80.32
50.15
49.75
28.38
27.48
21.27
20.33
20.13
S23
^{13}C, 125 MHz, CDCl$_3$
f1 (ppm)

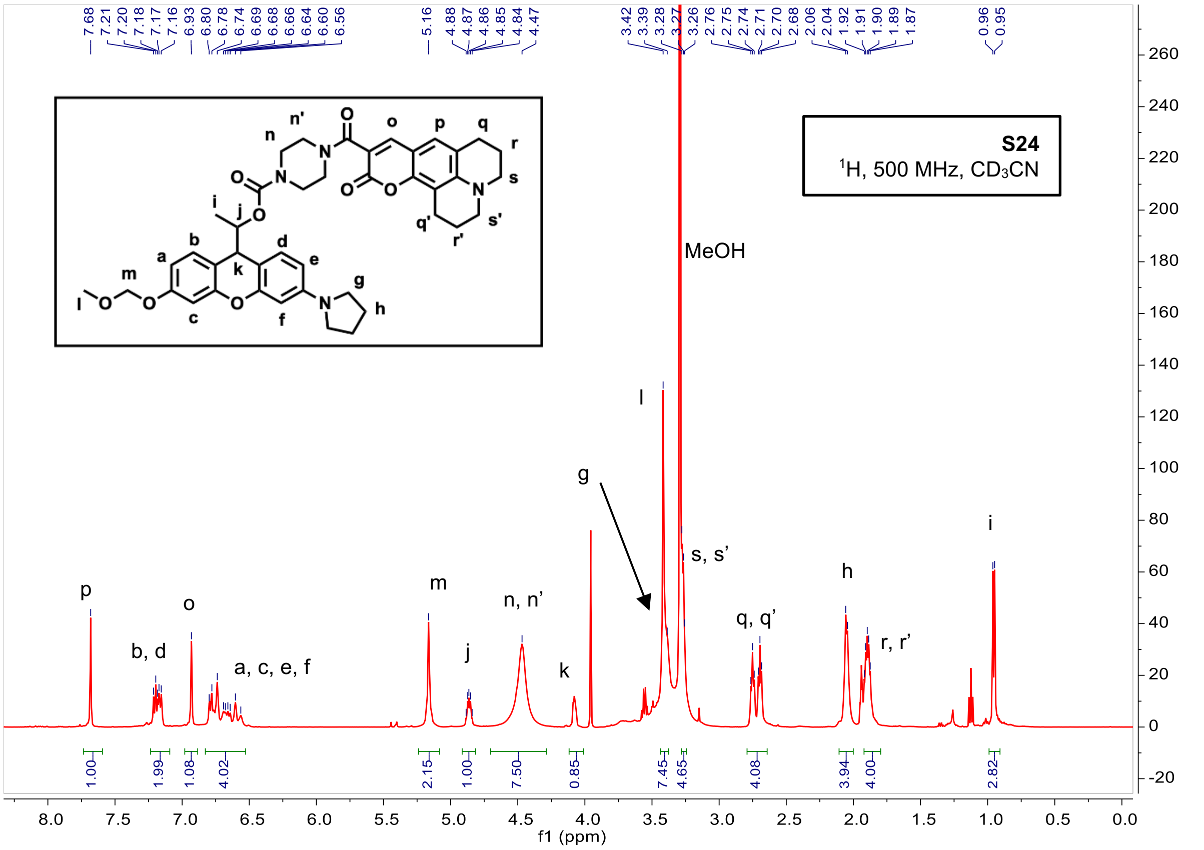
S24
^{1}H, 500 MHz, CD_3CN
MeOH
f1 (ppm)

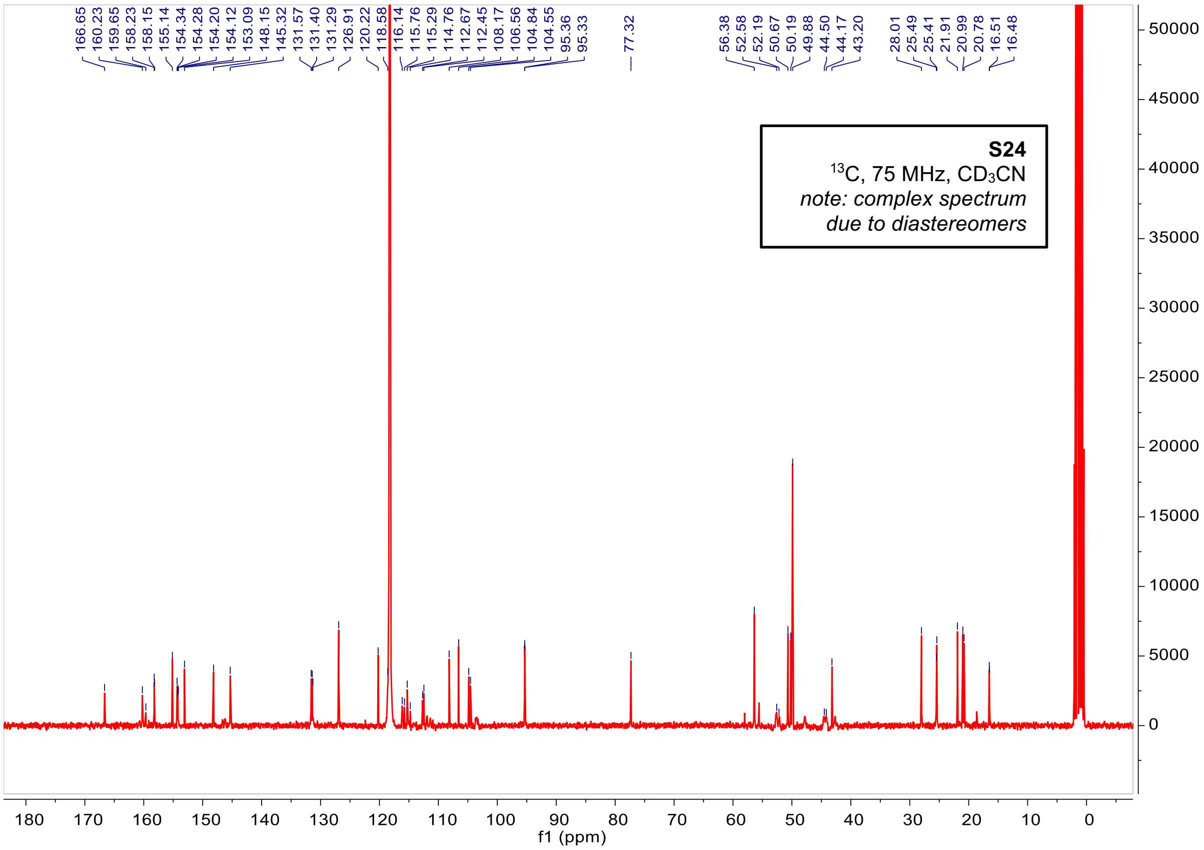
S24
^{13}C, 75 MHz, CD_3CN
note: complex spectrum due to diastereomers
f1 (ppm)

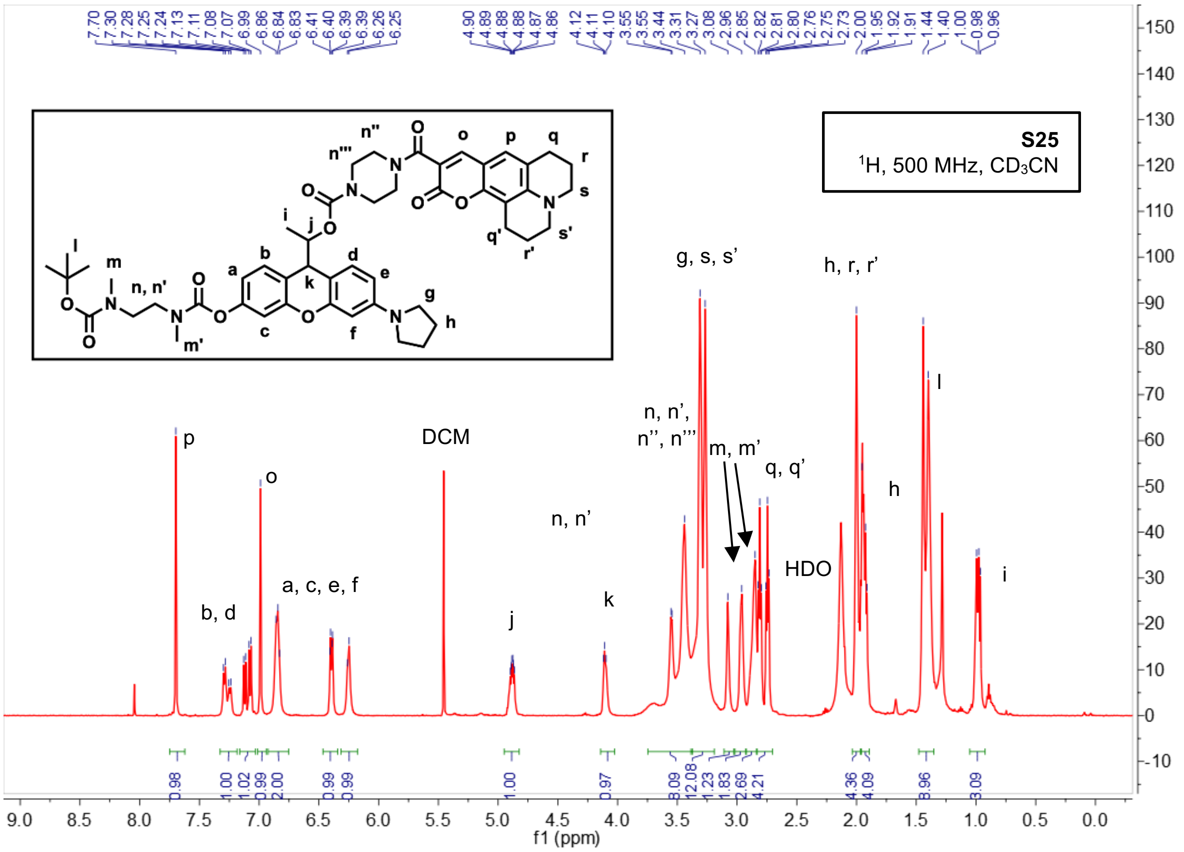
S25
¹H, 500 MHz, CD₃CN
p
o
b, d
a, c, e, f
DCM
j
n, n'
k
n, n',
n'', n'''
g, s, s'
m, m'
q, q'
HDO
h, r, r'
h
l
i
f1 (ppm)

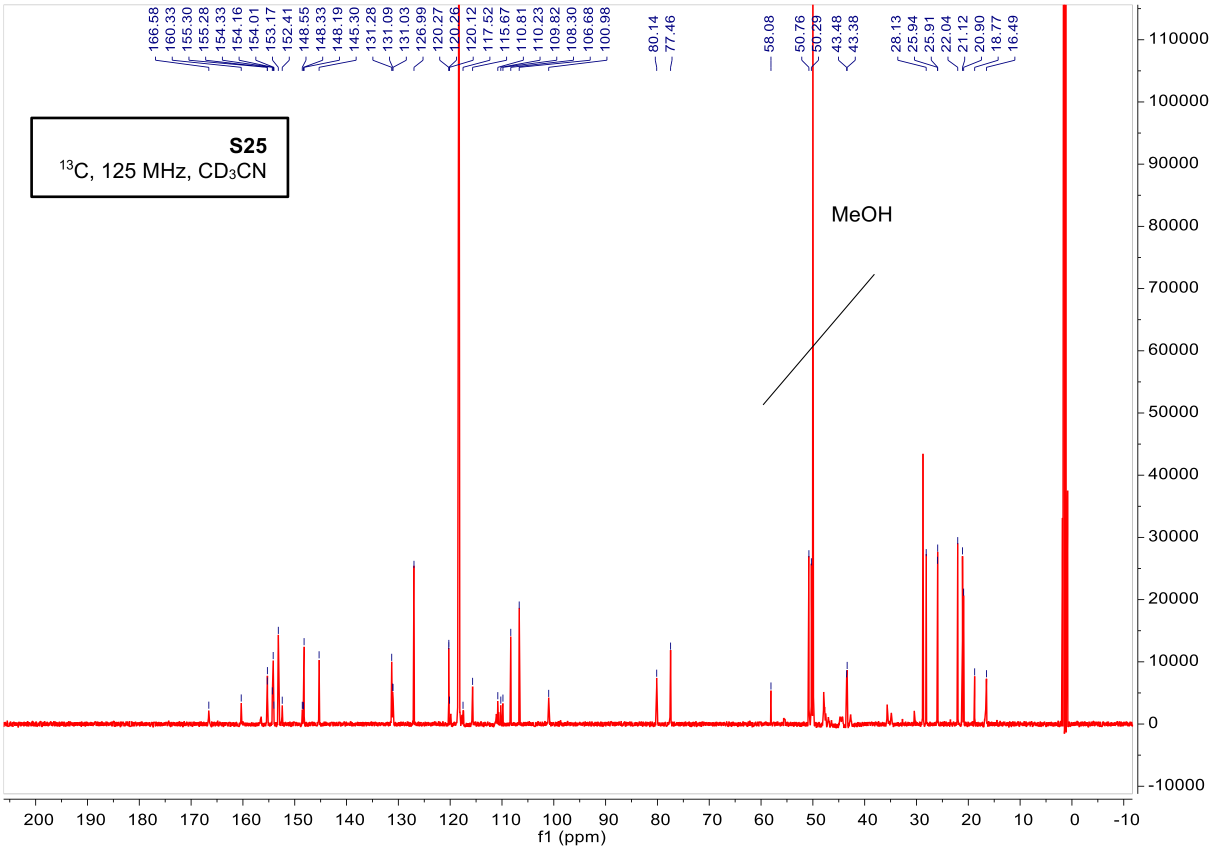
S25
¹³C, 125 MHz, CD₃CN
MeOH
f1 (ppm)

S25
$[M+H]^+$: 905

Chromatogram

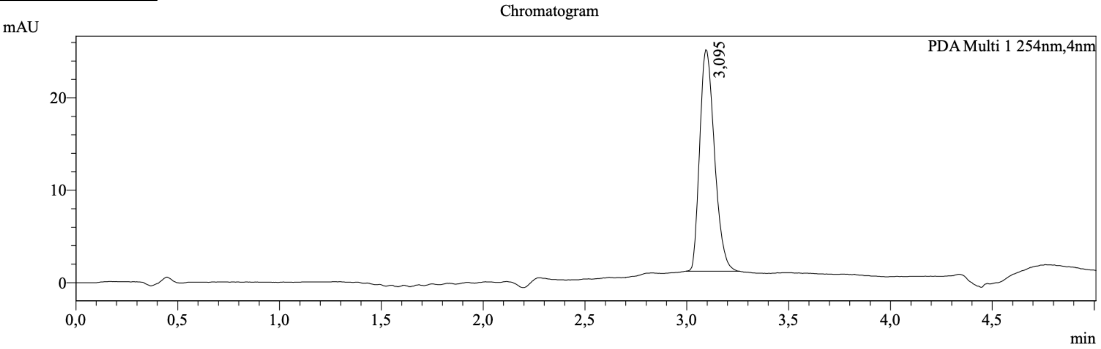

Peak Table

PDA Ch1 254nm

Peak#	Ret. Time	Peak Start	Peak End	Area	Height	Area/Height
1	3,095	2,997	3,259	121200	24007	5,049
Total				121200	24007	

Contour

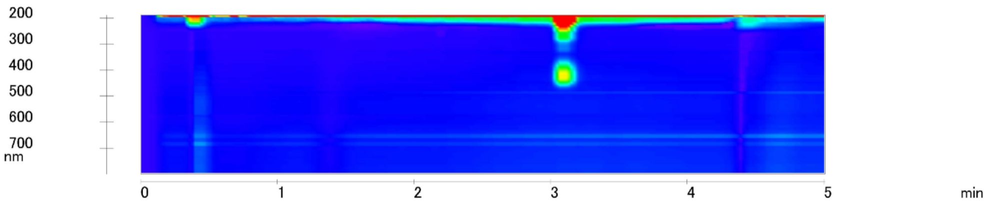

MS Chromatogram

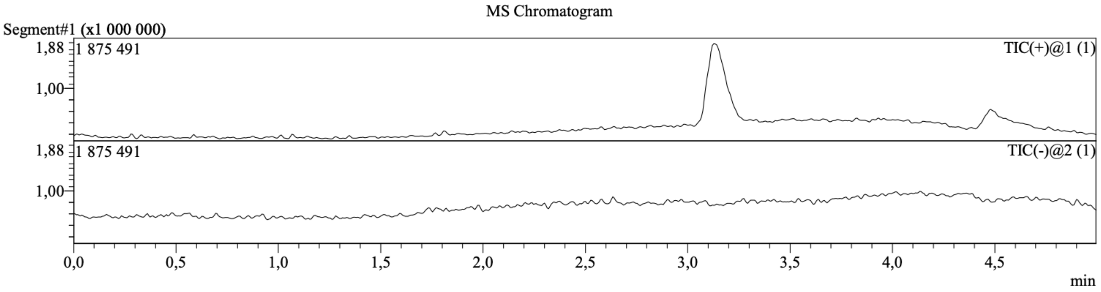

MS Spectrum

Line#:1 R.Time:----(Scan#:----)
MassPeaks:876
Spectrum Mode:Averaged 3,047-3,260(915-979) Base Peak:905(124688)
BG Mode:Averaged 1,847-2,180(555-655) Segment 1 - Event 1

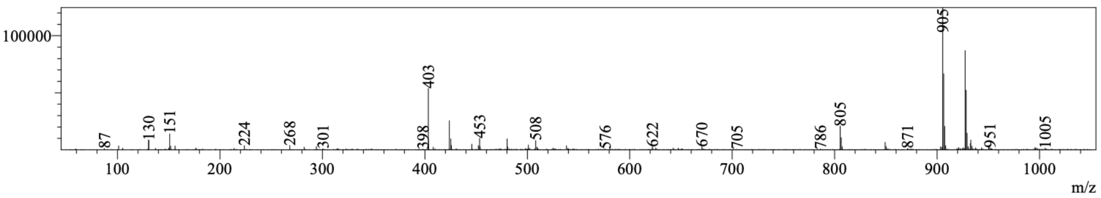

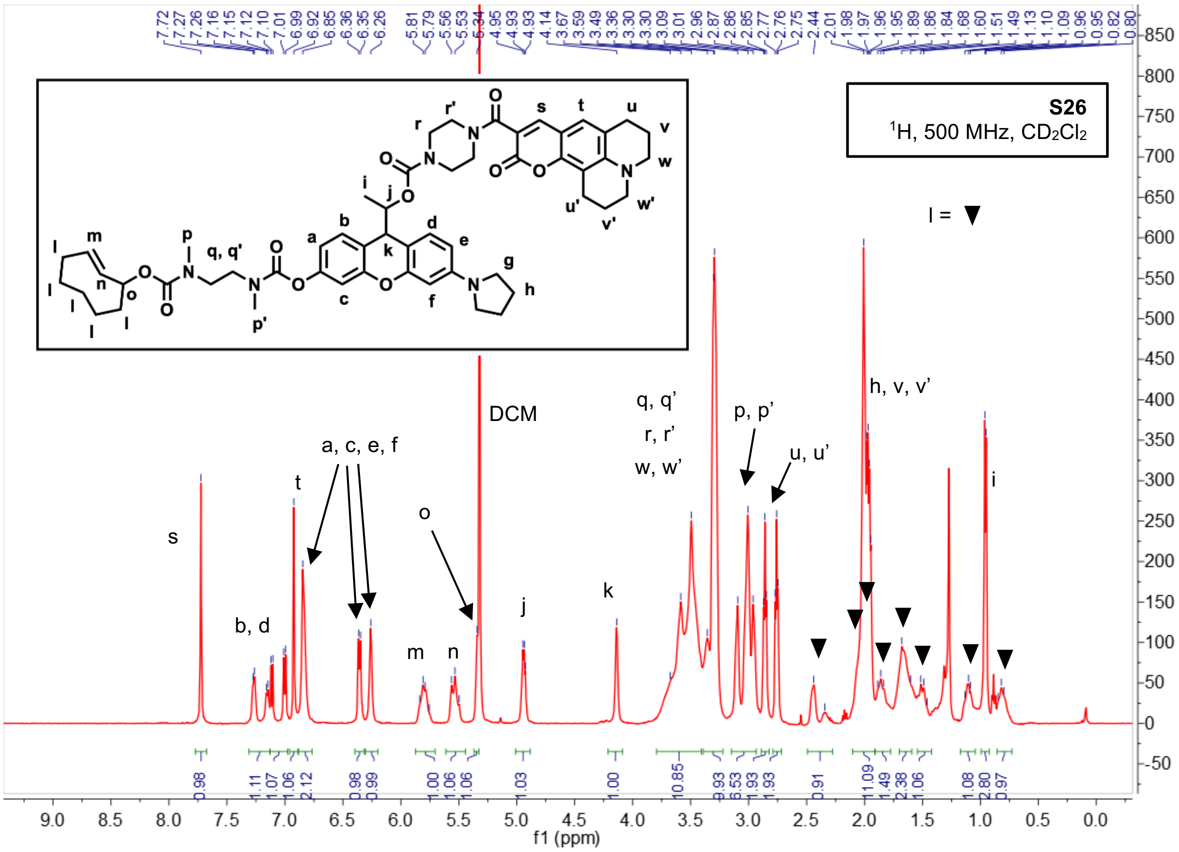
S26
¹H, 500 MHz, CD₂Cl₂
l = ▼
s
b, d
t
a, c, e, f
m
n
o
DCM
j
k
q, q'
r, r'
w, w'
p, p'
u, u'
h, v, v'
i
f1 (ppm)

S26
$[M+H]^+$: 957

Chromatogram

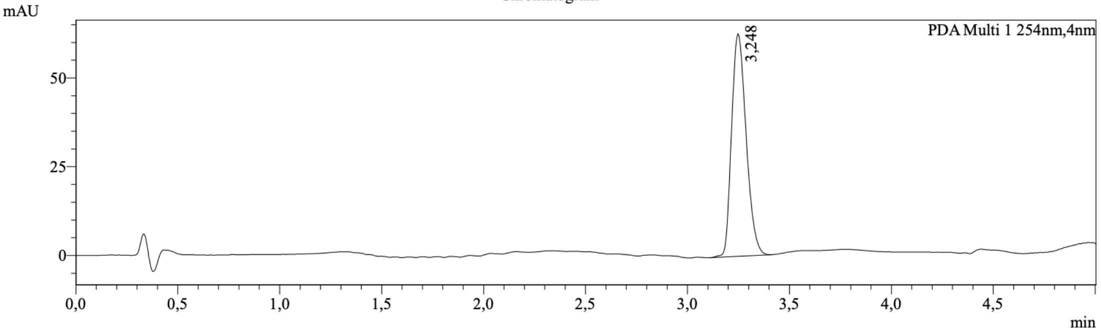

Peak Table

PDA Ch1 254nm

Peak#	Ret. Time	Peak Start	Peak End	Area	Height	Area/Height
1	3,248	3,109	3,408	311656	62769	4,965
Total				311656	62769	

Contour

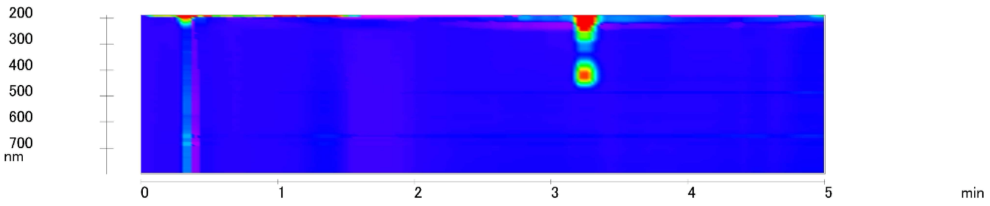

MS Chromatogram

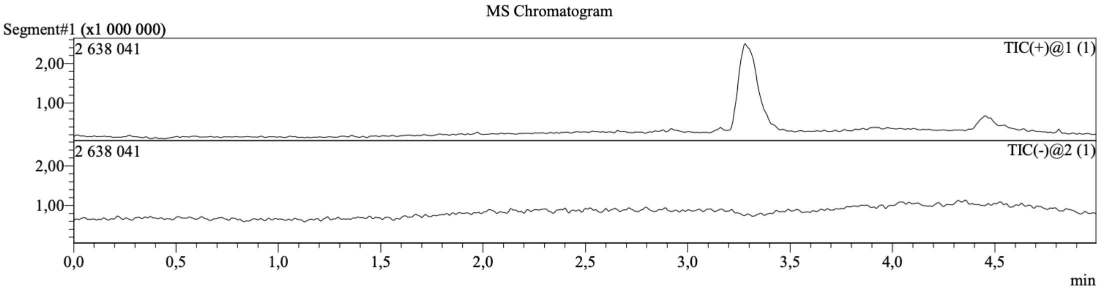

MS Spectrum

Line#:1 R.Time:----(Scan#:----)
MassPeaks:714
Spectrum Mode:Averaged 3,207-3,440(963-1033) Base Peak:957(259662)
BG Mode:Averaged 2,127-2,453(639-737) Segment 1 - Event 1

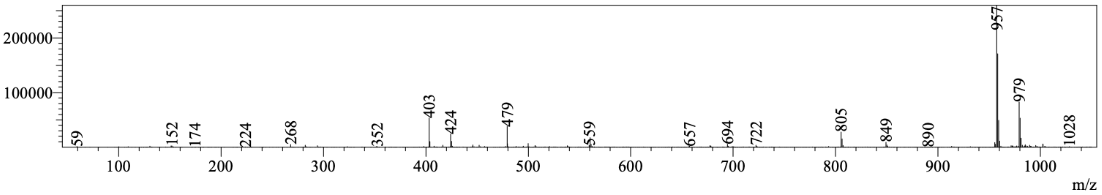

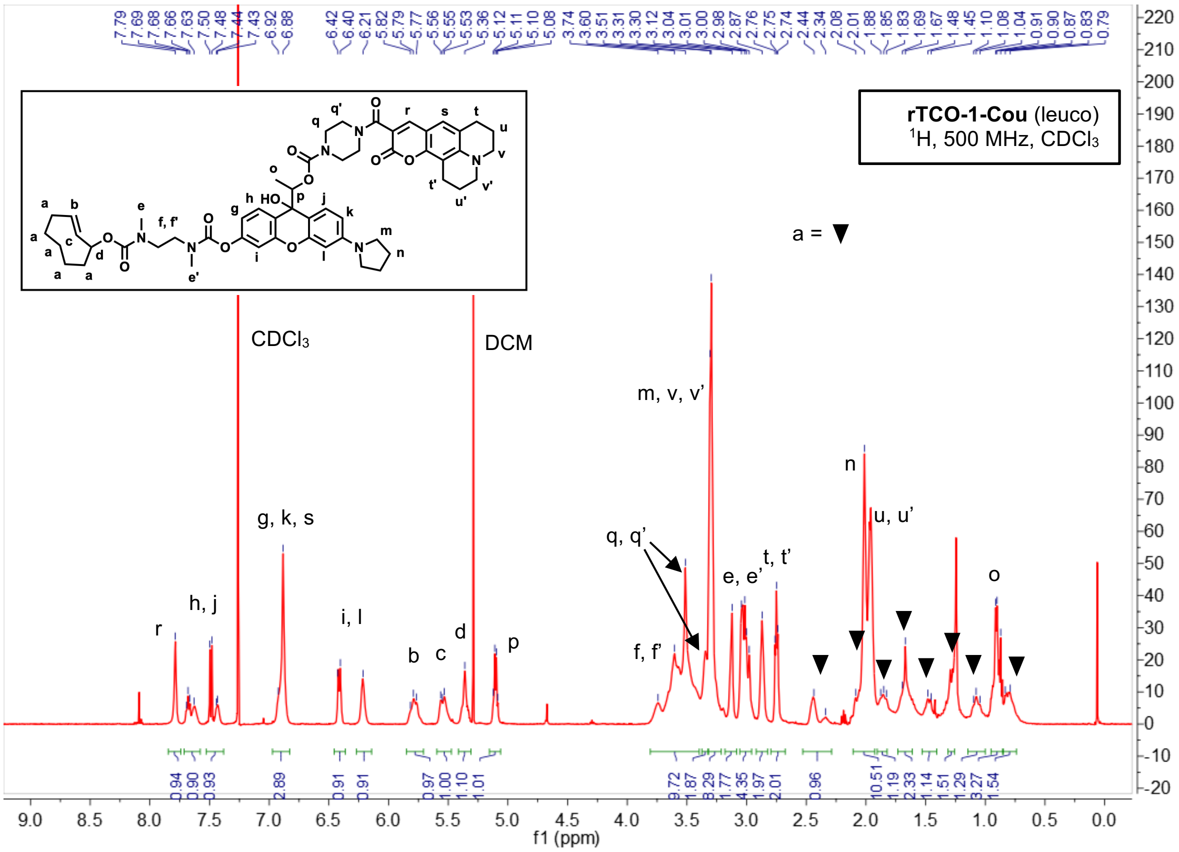
rTCO-1-Cou (leuco)
¹H, 500 MHz, CDCl3
a = ▼
CDCl3
DCM
m, v, v'
n
u, u'
g, k, s
q, q'
e, e'
t, t'
h, j
r
i, l
d
b
c
p
f, f'
o
f1 (ppm)

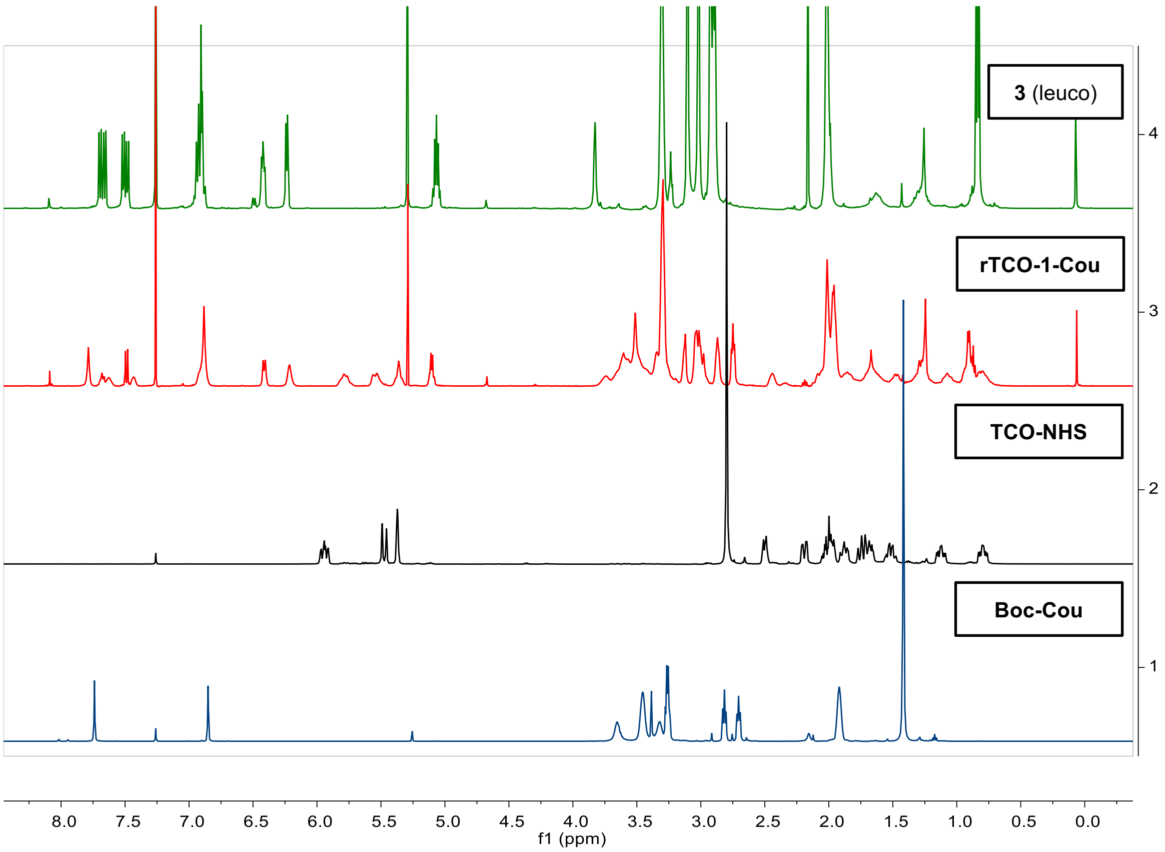
3 (leuco)
rTCO-1-Cou
TCO-NHS
Boc-Cou
f1 (ppm)

rTCO-1-Cou
$[M-H_2O+H]^+$: 955

Chromatogram

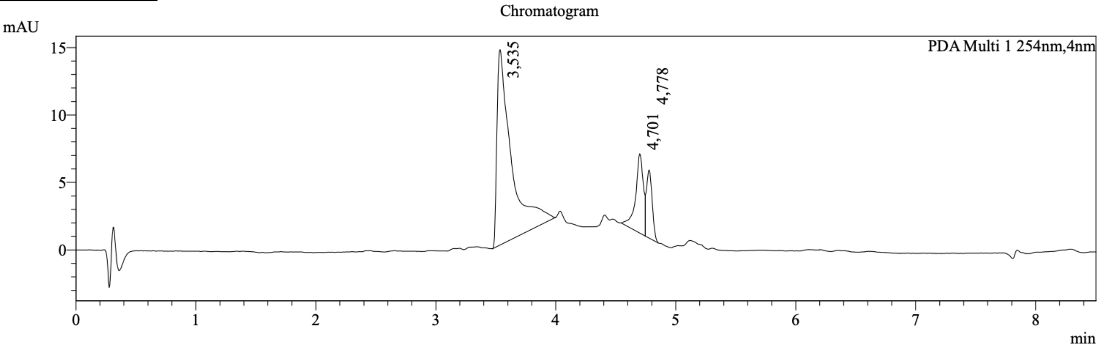

Peak Table

PDA Ch1 254nm

Peak#	Ret. Time	Peak Start	Peak End	Area	Height	Area/Height
1	3,535	3,472	3,995	120904	14489	8,344
2	4,701	4,544	4,747	27000	5877	4,594
3	4,778	4,747	4,853	16184	5042	3,210
Total				164088	25408	

Contour

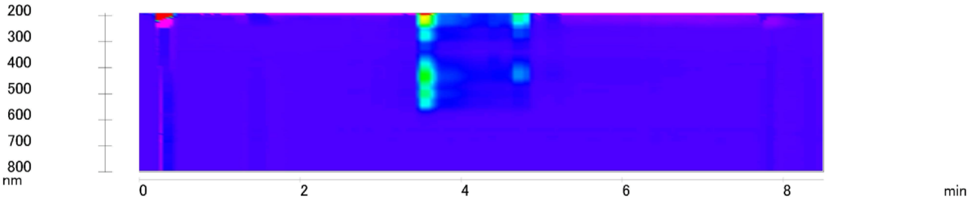

MS Chromatogram

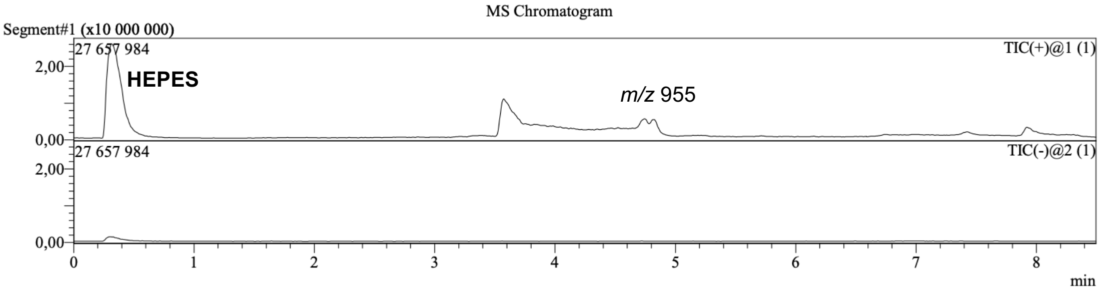

MS Spectrum

Line#:1 R.Time:----(Scan#:----)
MassPeaks:769
Spectrum Mode:Averaged 3,507-3,740(1053-1123) Base Peak:955(1299216)
BG Mode:Averaged 1,280-2,513(385-755) Segment 1 - Event 1

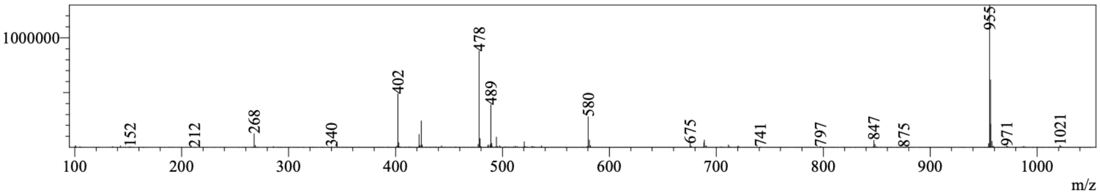

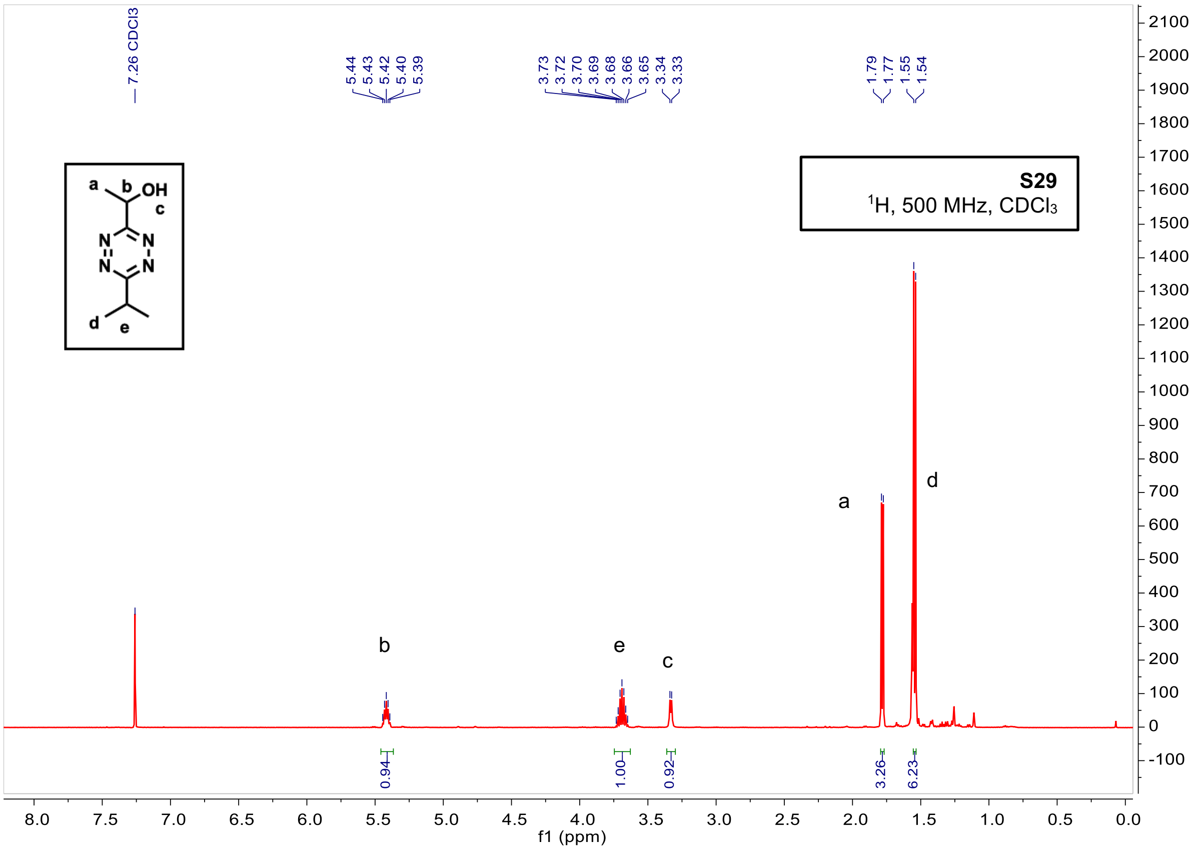

S29
1H, 500 MHz, CDCl3
7.26 CDCl3
5.44
5.43
5.42
5.40
5.39
3.73
3.72
3.70
3.69
3.68
3.66
3.65
3.34
3.33
1.79
1.77
1.55
1.54
a
b
c
d
e
OH
0.94
1.00
0.92
3.26
6.23
f1 (ppm)

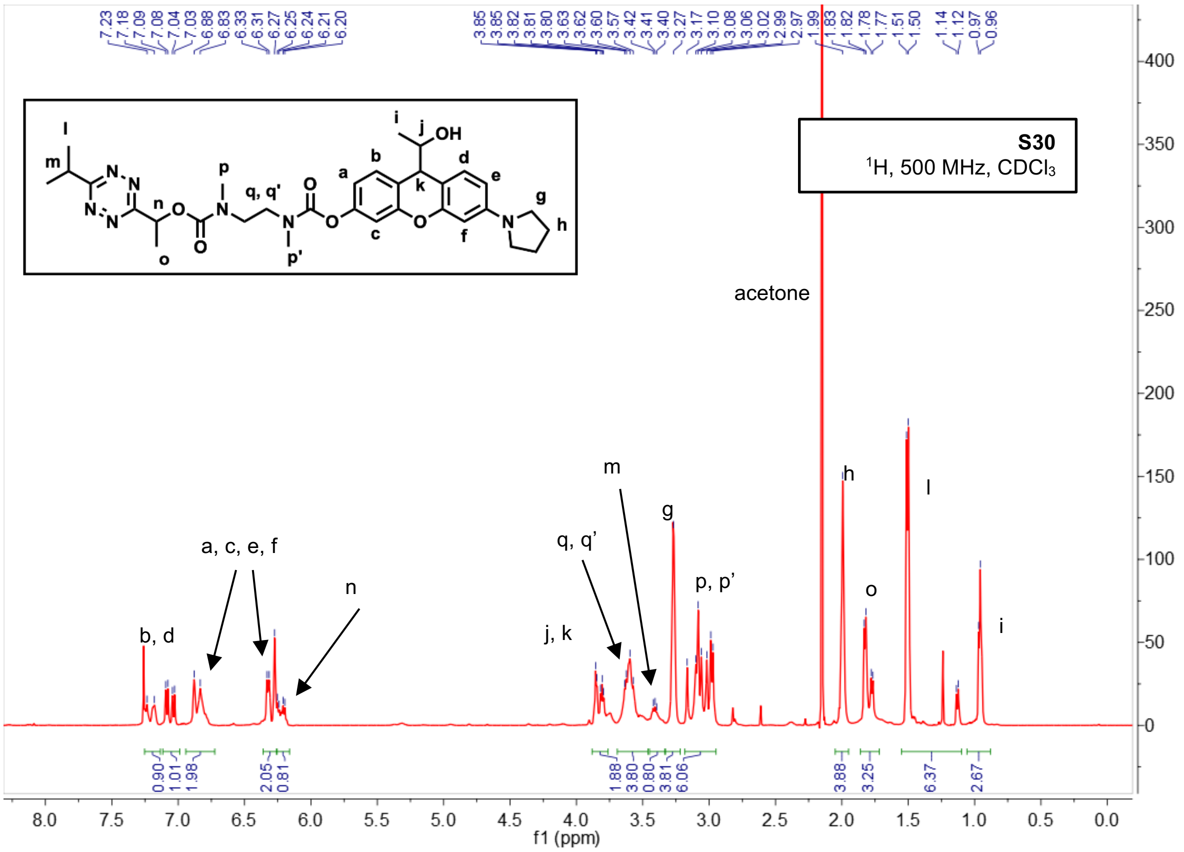

S30
1H, 500 MHz, CDCl3
acetone
b, d
a, c, e, f
n
j, k
q, q'
m
g
p, p'
h
o
l
i
f1 (ppm)

S30
$[M+H]^+$: 620

Chromatogram

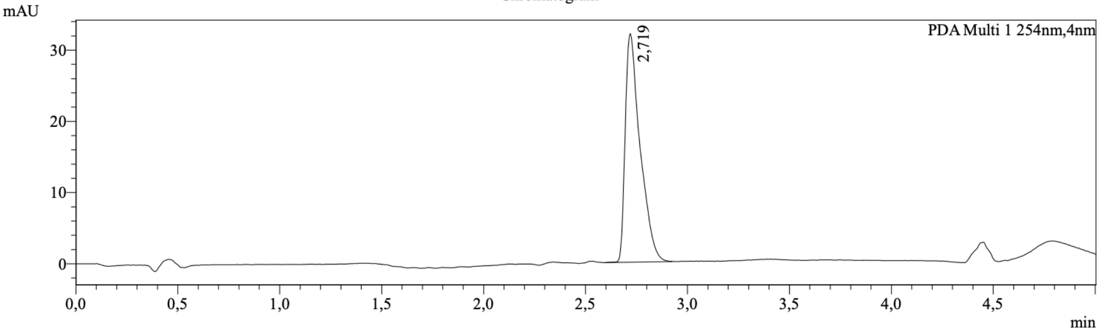

Peak Table

PDA Ch1 254nm

Peak#	Ret. Time	Peak Start	Peak End	Area	Height	Area/Height
1	2,719	2,597	2,923	162994	32127	5,073
Total				162994	32127	

Contour

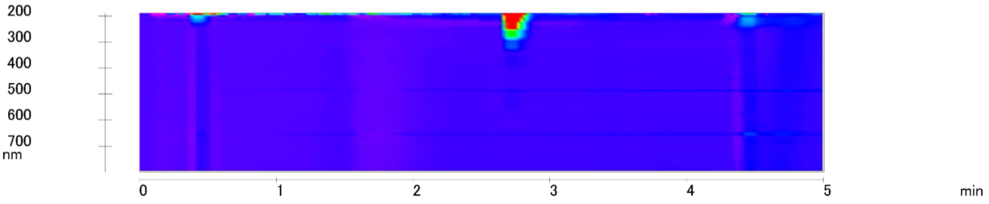

MS Chromatogram

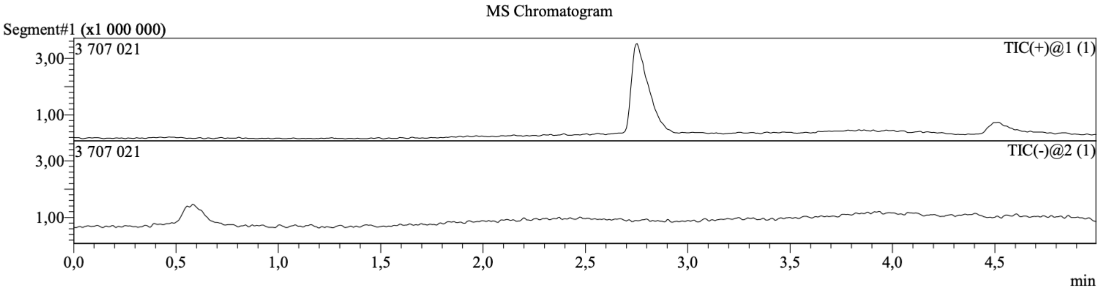

MS Spectrum

Line#:1 R.Time:----(Scan#:----)
MassPeaks:840
Spectrum Mode:Averaged 2,673-2,907(803-873) Base Peak:620(532227)
BG Mode:Averaged 2,027-2,220(609-667) Segment 1 - Event 1

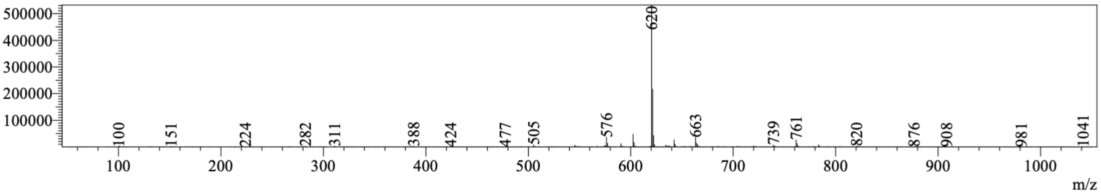

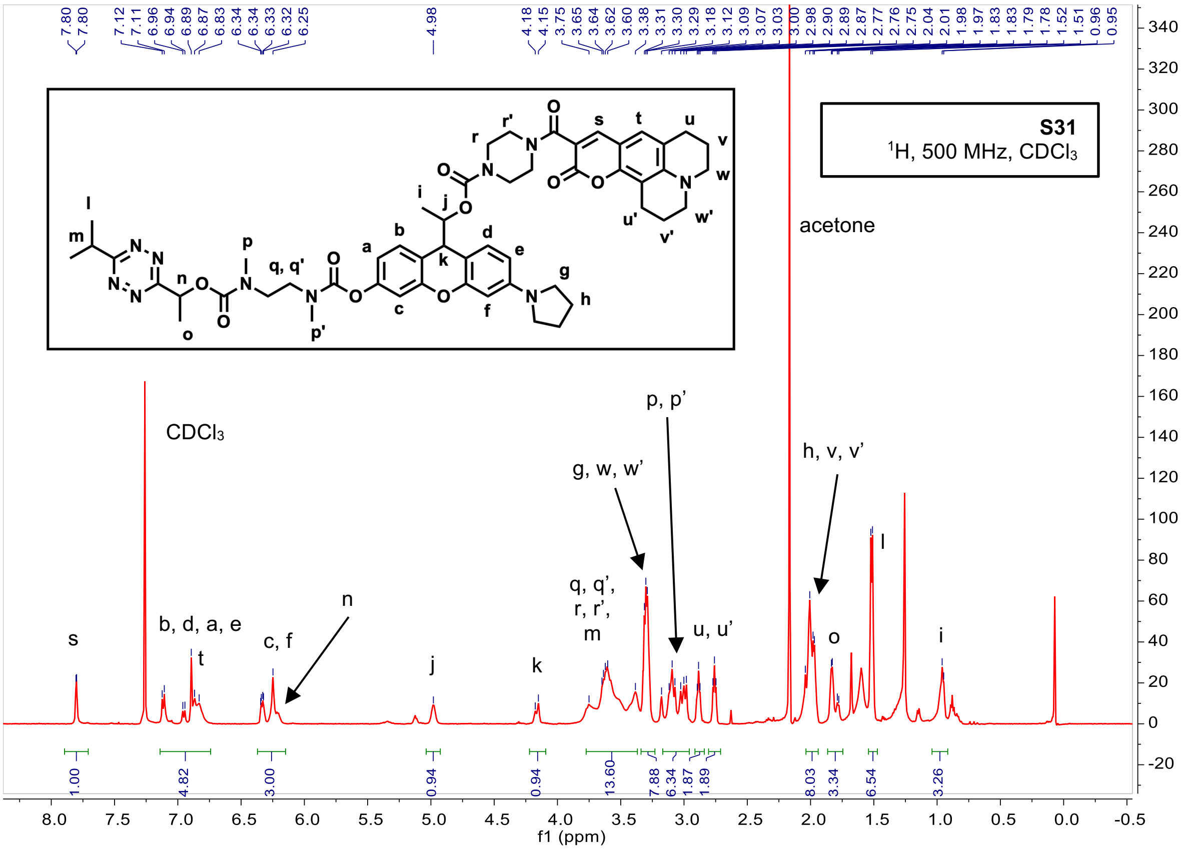

S31
^{1}H, 500 MHz, $CDCl_3$
acetone
$CDCl_3$
s
b, d, a, e
t
c, f
n
j
k
q, q', r, r', m
g, w, w'
p, p'
u, u'
h, v, v'
o
l
i
f1 (ppm)

S31
$[M+H]^+$: 999

Chromatogram

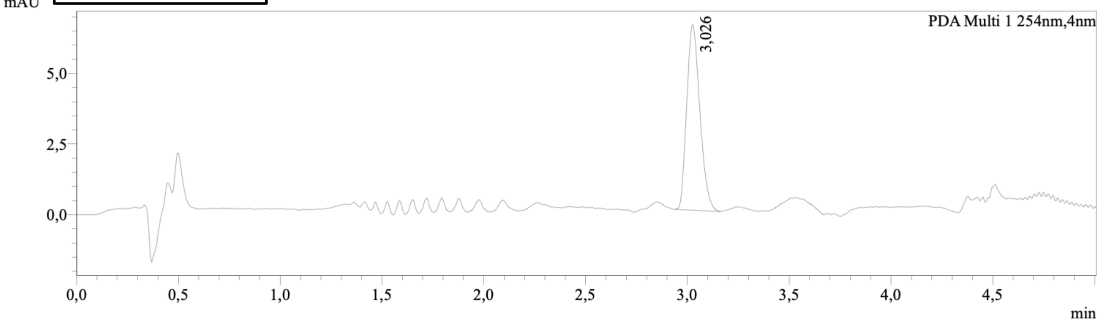

Peak Table

PDA Ch1 254nm

Peak#	Ret. Time	Peak Start	Peak End	Area	Height	Area/Height
1	3,026	2,939	3,163	29442	6570	4,481
Total				29442	6570	

Contour

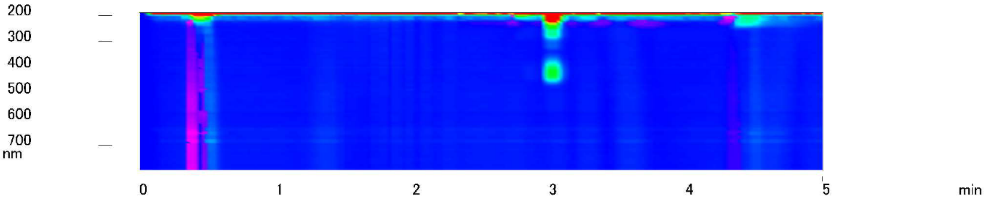

MS Chromatogram

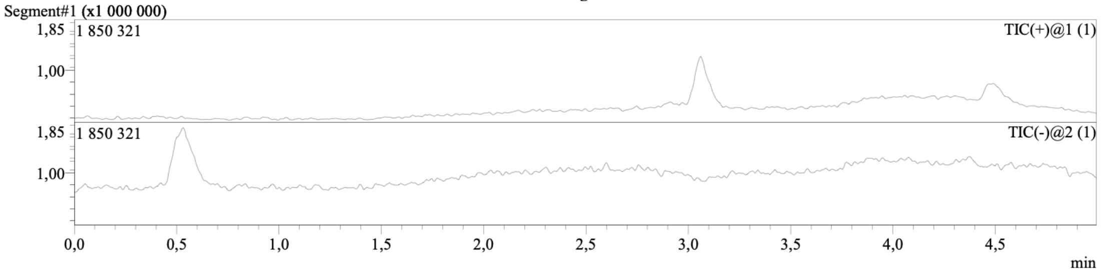

MS Spectrum

Line#:1 R.Time:----(Scan#:----)
MassPeaks:755
Spectrum Mode:Averaged 2,993-3,153(899-947) Base Peak:500(86620)
BG Mode:Averaged 2,160-2,480(649-745) Segment 1 - Event 1

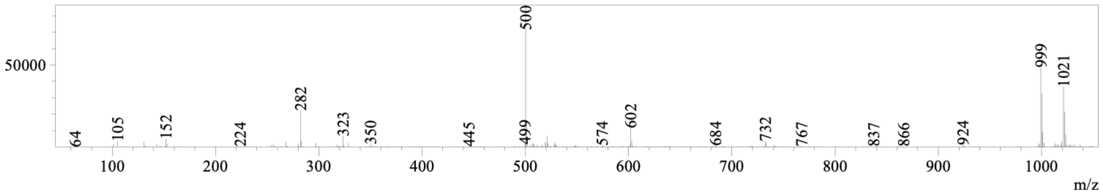

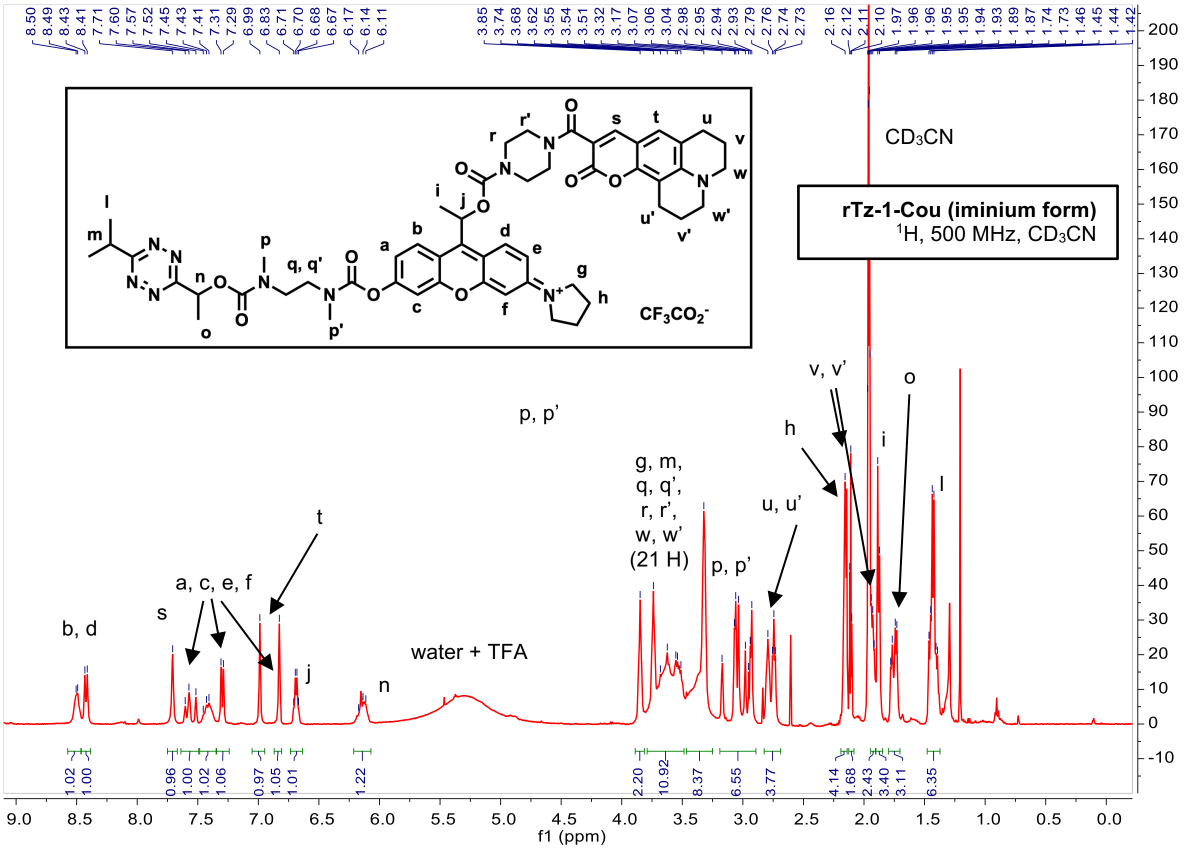
rTz-1-Cou (iminium form)
1H, 500 MHz, CD3CN
CD3CN
CF3CO2-
b, d
s
a, c, e, f
t
j
n
water + TFA
p, p'
g, m,
q, q',
r, r',
w, w'
(21 H)
p, p'
u, u'
h
v, v'
i
o
l
f1 (ppm)

rTz-1-Cou
[M]$^+$: 997

Chromatogram

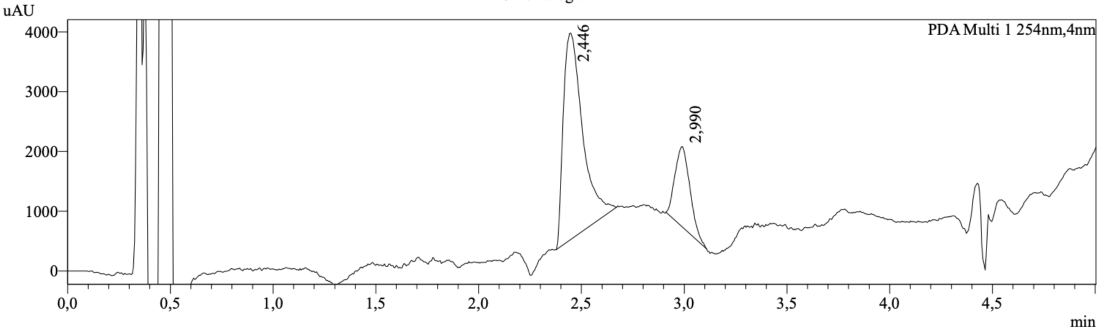

Peak Table

PDA Ch1 254nm

Peak#	Ret. Time	Peak Start	Peak End	Area	Height	Area/Height
1	2,446	2,379	2,672	22718	3459	6,568
2	2,990	2,912	3,109	6677	1338	4,990
Total				29395	4797	

Contour

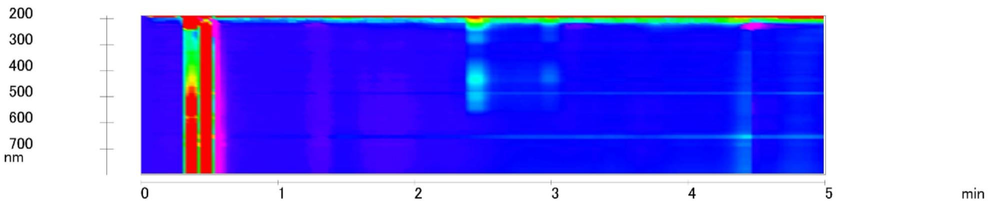

MS Chromatogram

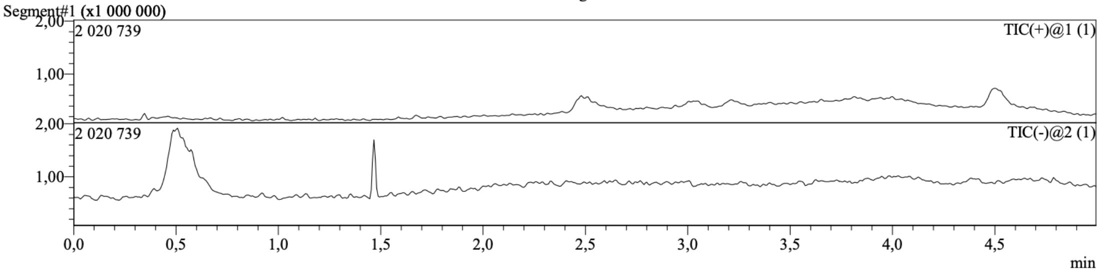

MS Spectrum

Line#:1 R.Time:----(Scan#:----)
MassPeaks:700
Spectrum Mode:Averaged 2,427-2,580(729-775) Base Peak:499(57510)
BG Mode:Averaged 1,780-2,180(535-655) Segment 1 - Event 1

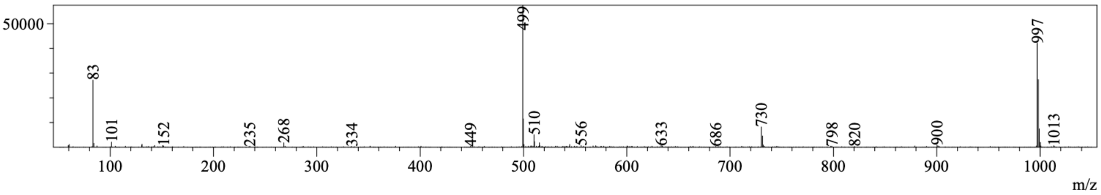

Line#:2 R.Time:----(Scan#:----)
MassPeaks:805
Spectrum Mode:Averaged 2,953-3,087(887-927) Base Peak:997(30909)
BG Mode:Averaged 1,620-2,033(487-611) Segment 1 - Event 1

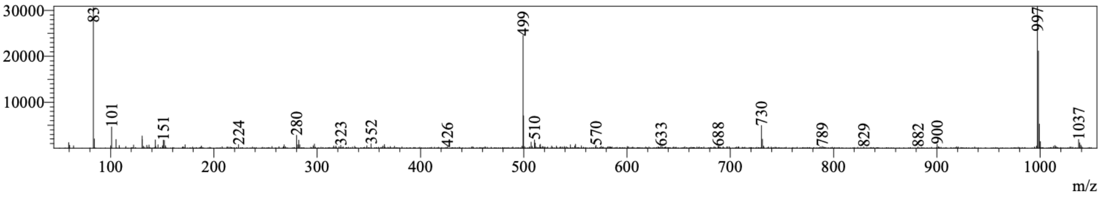

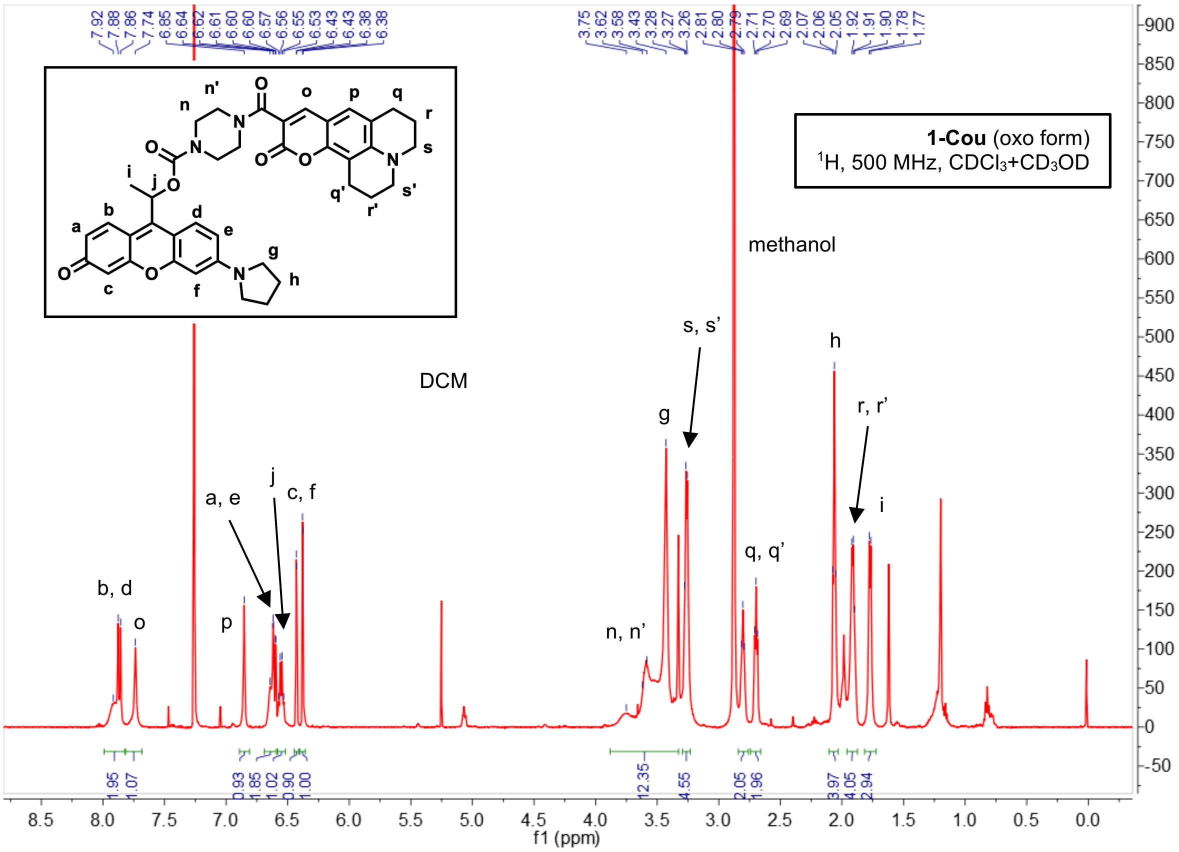
1-Cou (oxo form)
1H, 500 MHz, CDCl3+CD3OD
methanol
DCM
b, d
o
p
a, e
j
c, f
n, n’
g
s, s’
q, q’
h
r, r’
i
f1 (ppm)

1-Cou
$[M+H]^+$: 689

Chromatogram

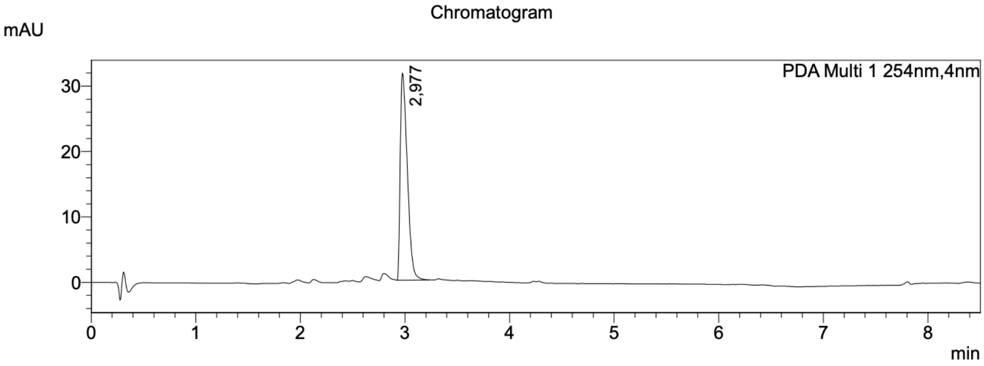

Contour

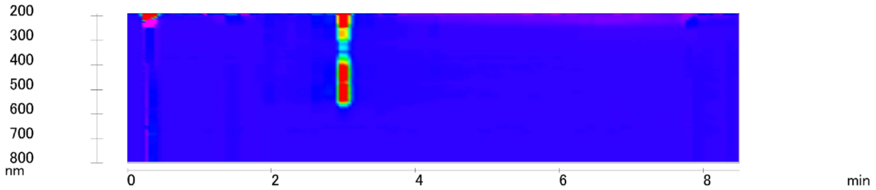

Peak Table

PDA Ch1 254nm

Peak#	Ret. Time	Peak Start	Peak End	Area	Height	Area/Height
1	2,977	2,923	3,232	148382	31662	4,686
Total				148382	31662	

MS Chromatogram

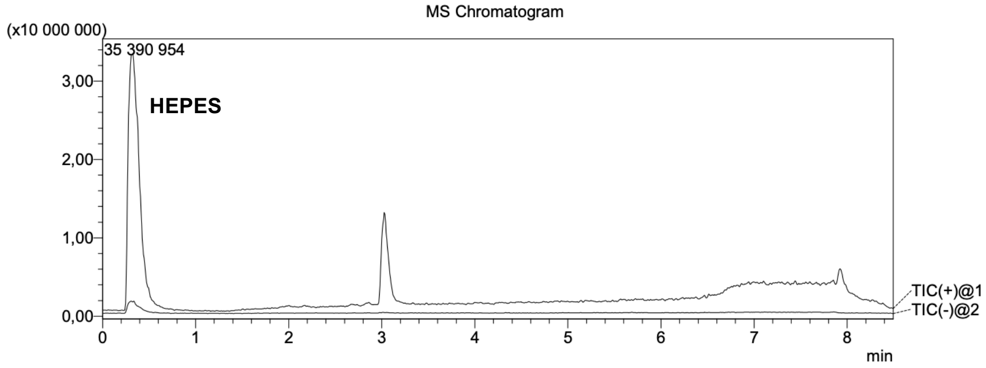

MS Spectrum

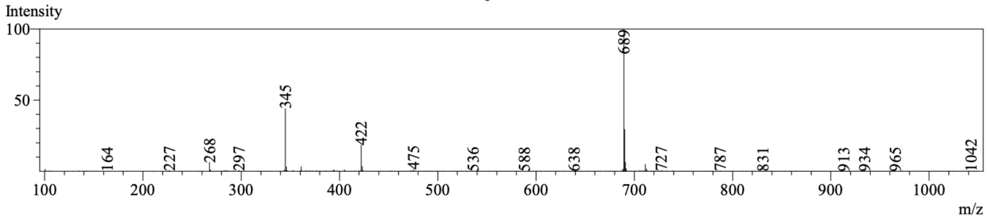

MS Spectrum

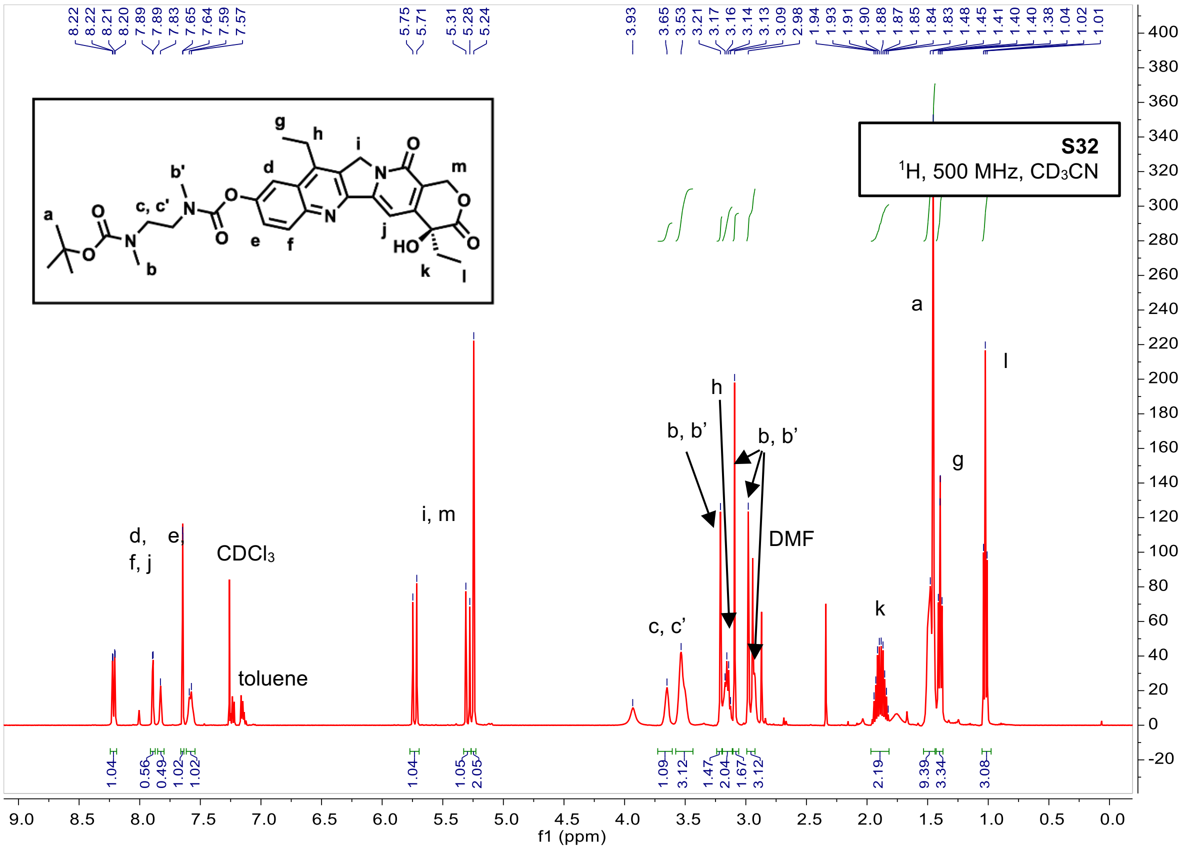
S32
¹H, 500 MHz, CD₃CN
a
b
b'
c, c'
d
e
f
g
h
i
j
k
l
m
d,
f, j
e
CDCl₃
toluene
i, m
c, c'
b, b'
h
b, b'
DMF
k
a
g
l
f1 (ppm)

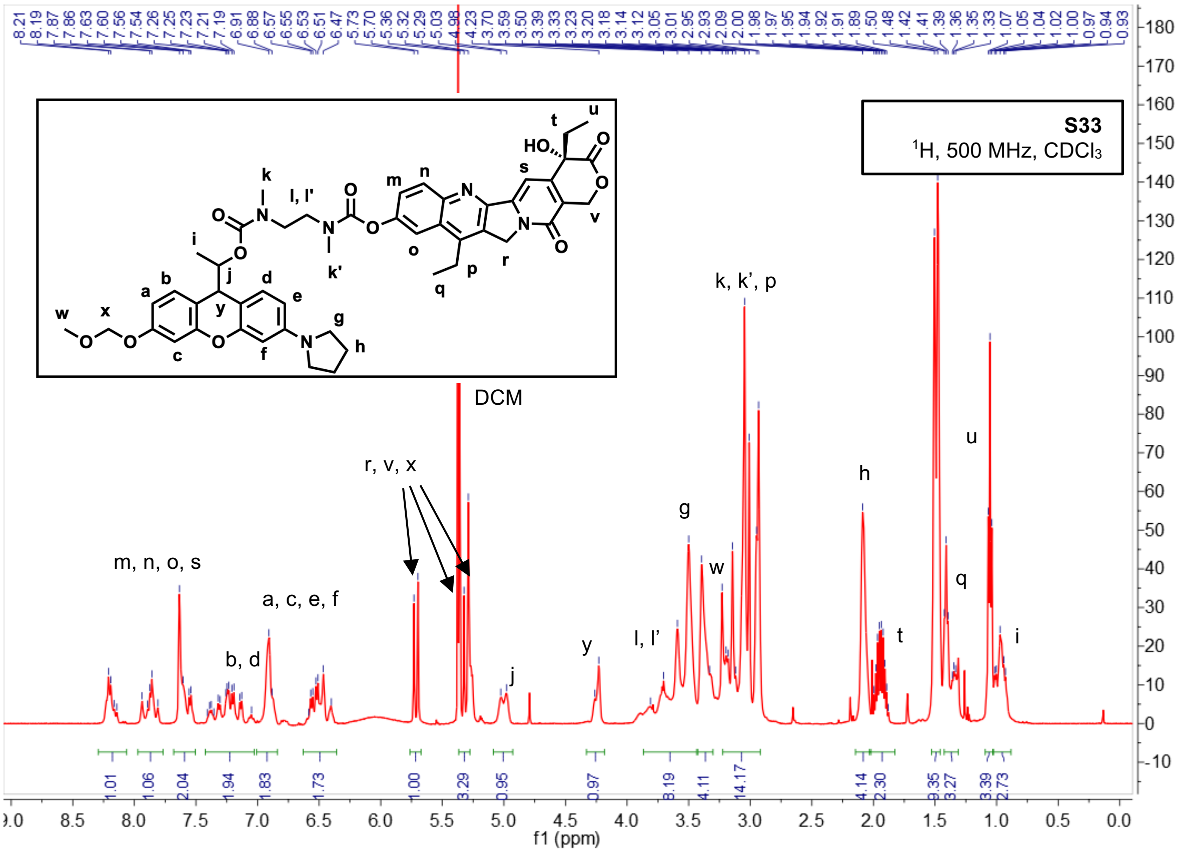

S33
1H, 500 MHz, CDCl3
DCM
m, n, o, s
b, d
a, c, e, f
r, v, x
j
y
l, l'
g
w
k, k', p
h
t
q
u
i
f1 (ppm)

S33
[M+H]+: 888

Chromatogram

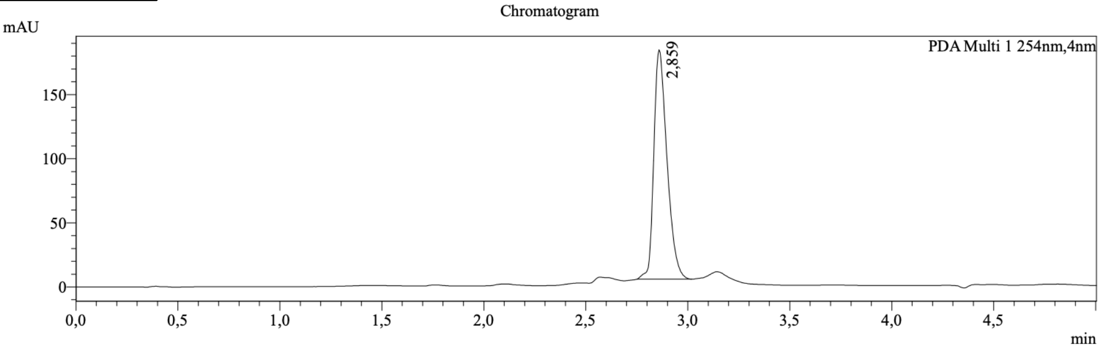

Peak Table

PDA Ch1 254nm

Peak#	Ret. Time	Peak Start	Peak End	Area	Height	Area/Height
1	2,859	2,752	3,019	803176	178970	4,488
Total				803176	178970	

Contour

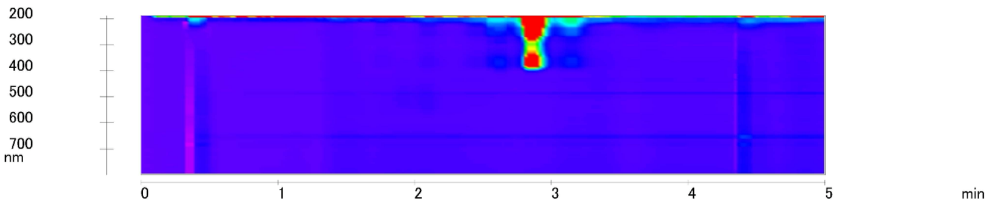

MS Chromatogram

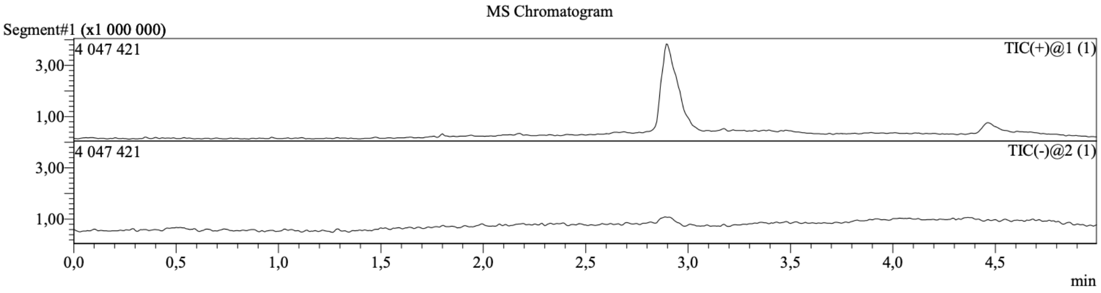

MS Spectrum

Line#:1 R.Time:----(Scan#:----)
MassPeaks:775
Spectrum Mode:Averaged 2,833-3,000(851-901) Base Peak:445(633445)
BG Mode:Averaged 1,833-2,320(551-697) Segment 1 - Event 1

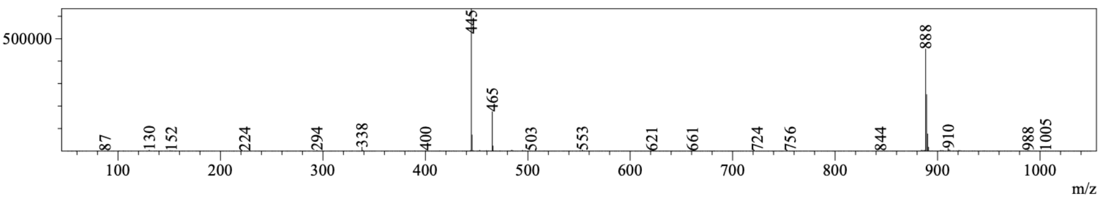

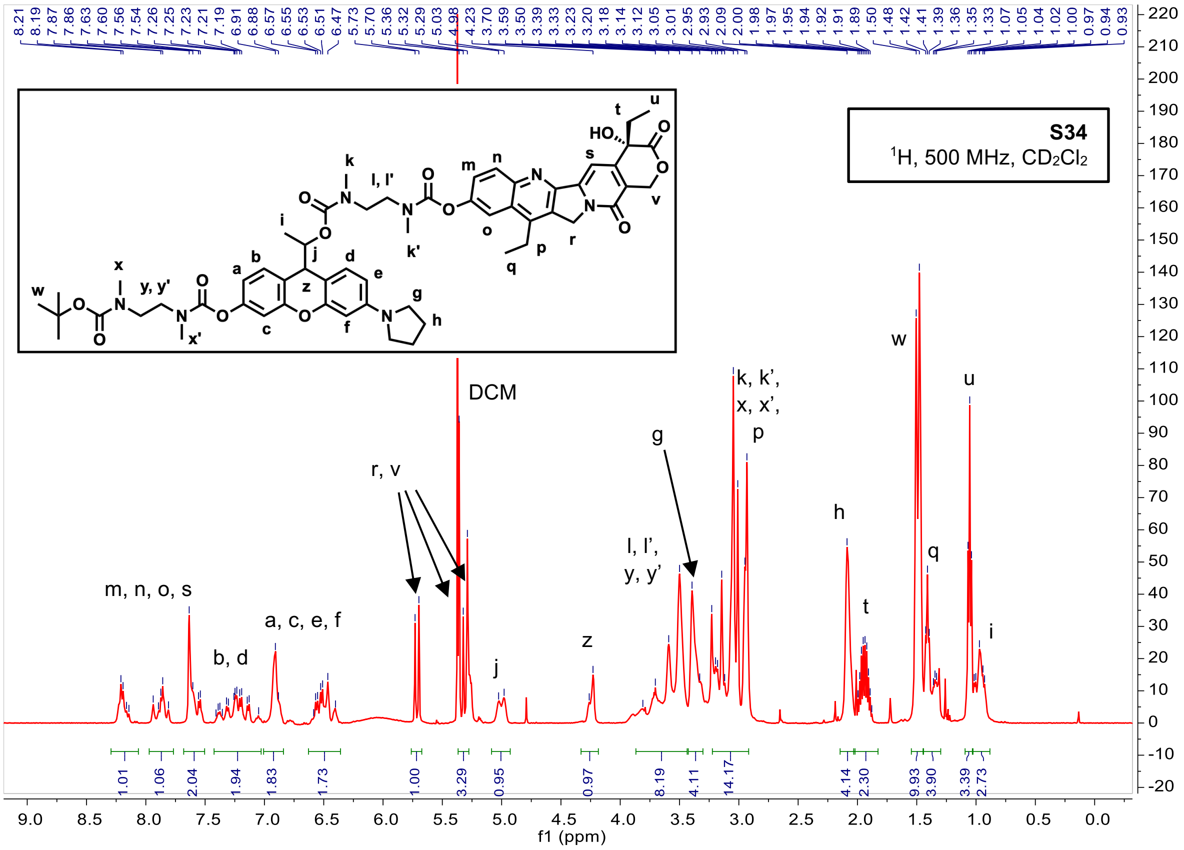
S34
1H, 500 MHz, CD2Cl2
DCM
m, n, o, s
b, d
a, c, e, f
r, v
j
z
l, l',
y, y'
g
k, k',
x, x',
p
h
t
w
q
u
i
f1 (ppm)

S34
$[M+2H]^{2+}$: 530
$[M-Boc+2H]^{2+}$: 480

Chromatogram

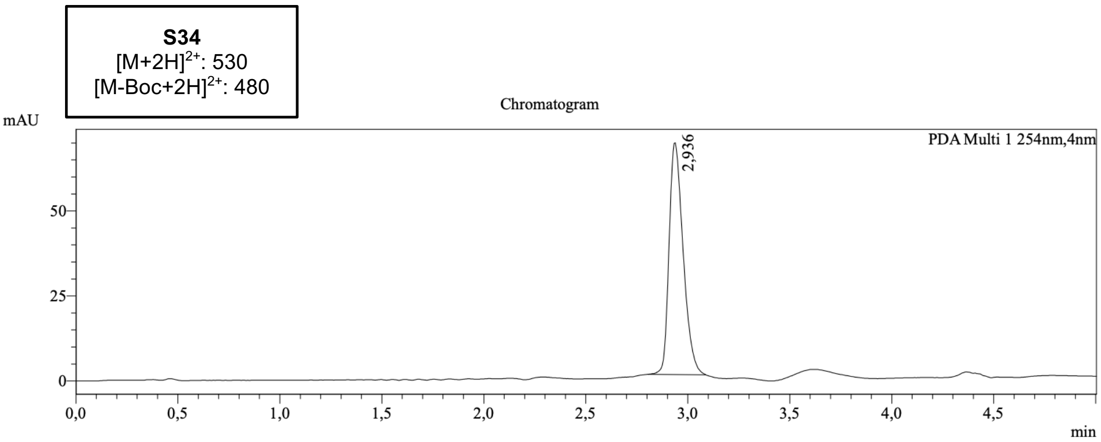

Peak Table

PDA Ch1 254nm

Peak#	Ret. Time	Peak Start	Peak End	Area	Height	Area/Height
1	2,936	2,805	3,088	327499	68162	4,805
Total				327499	68162	

Contour

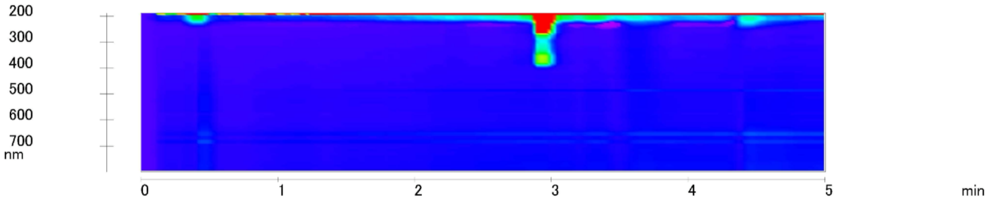

MS Chromatogram

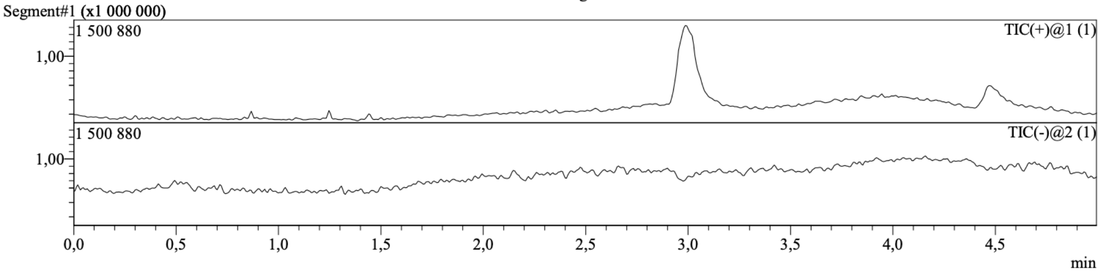

MS Spectrum

Line#:1 R.Time:----(Scan#:----)
MassPeaks:808
Spectrum Mode:Averaged 2,907-3,100(873-931) Base Peak:530(126147)
BG Mode:Averaged 1,753-2,087(527-627) Segment 1 - Event 1

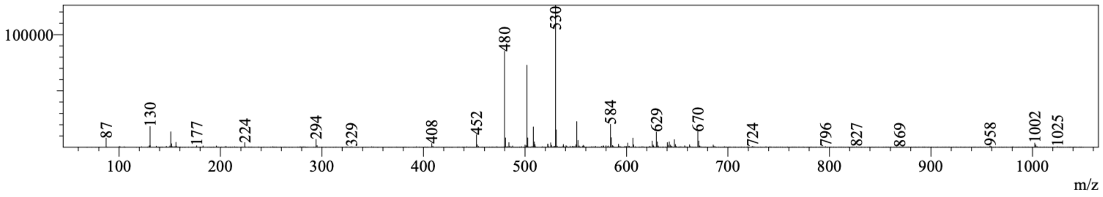

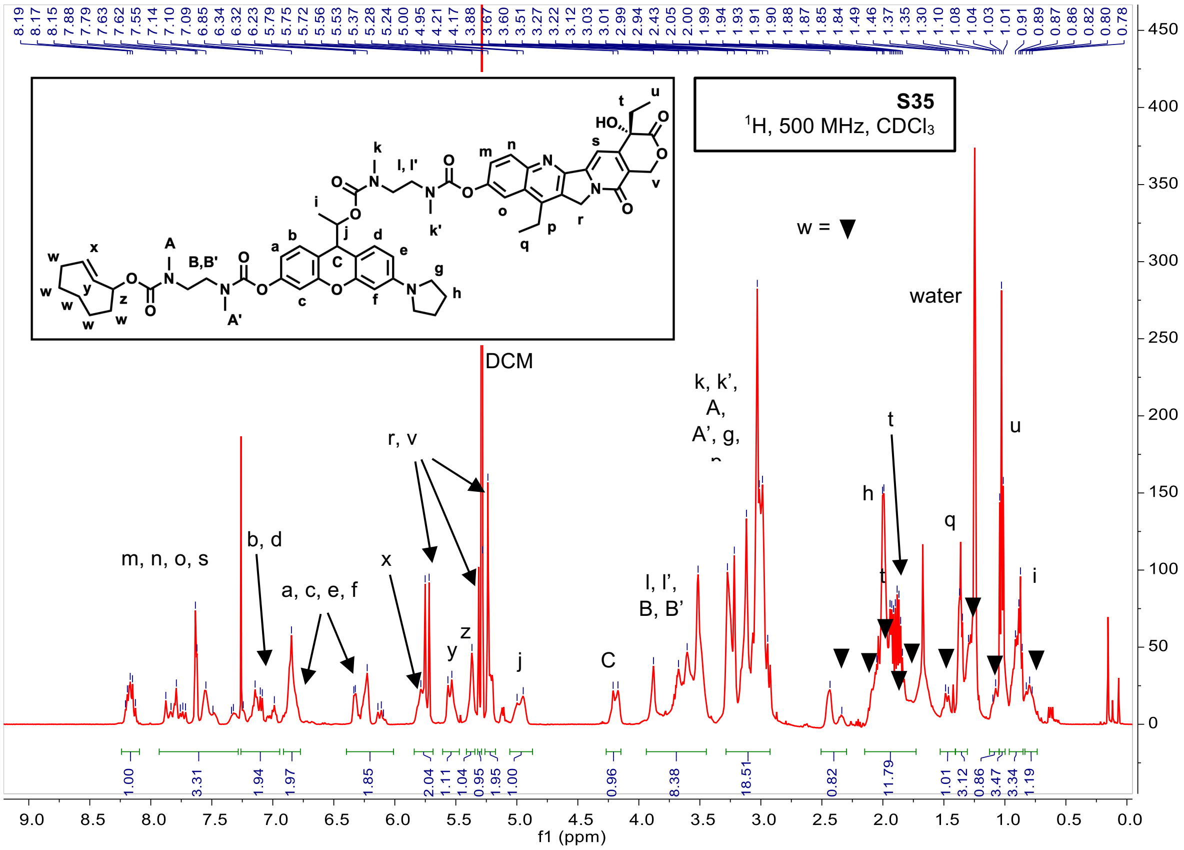

S35
^1H, 500 MHz, $CDCl_3$
w = ▼
water
DCM
m, n, o, s
b, d
a, c, e, f
x
r, v
y
z
j
C
l, l',
B, B'
k, k',
A,
A', g,
h
t
q
u
i
f1 (ppm)

S35
$[M+2H]^{2+}$: 556

Chromatogram

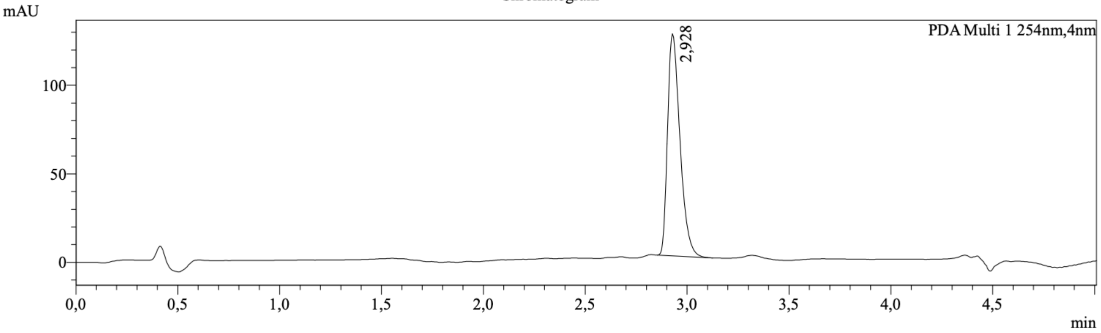

Peak Table

PDA Ch1 254nm

Peak#	Ret. Time	Peak Start	Peak End	Area	Height	Area/Height
1	2,928	2,853	3,125	531257	125560	4,231
Total				531257	125560	

Contour

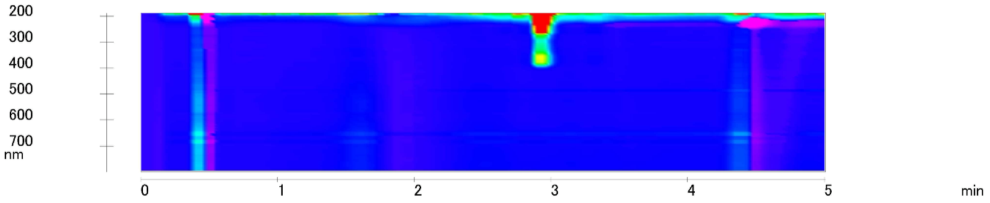

MS Chromatogram

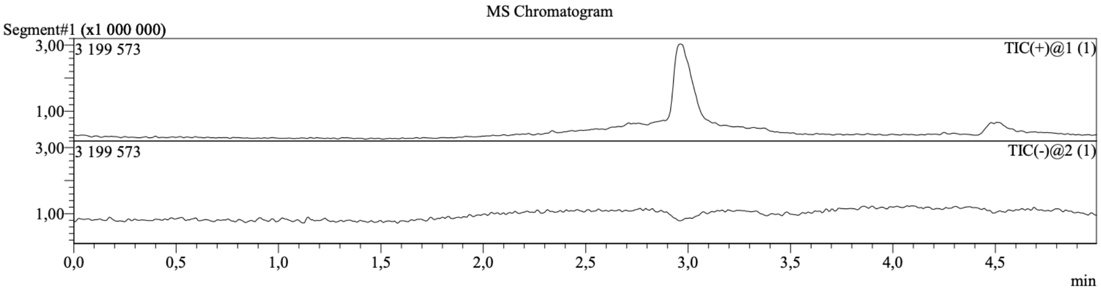

MS Spectrum

Line#:1 R.Time:----(Scan#:----)
MassPeaks:870
Spectrum Mode:Averaged 2,887-3,067(867-921) Base Peak:556(724745)
BG Mode:Averaged 1,853-2,093(557-629) Segment 1 - Event 1

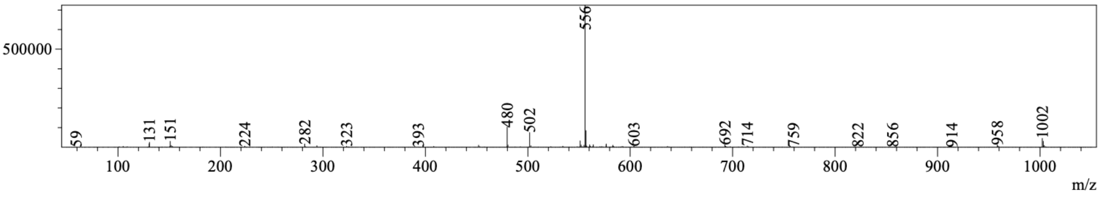

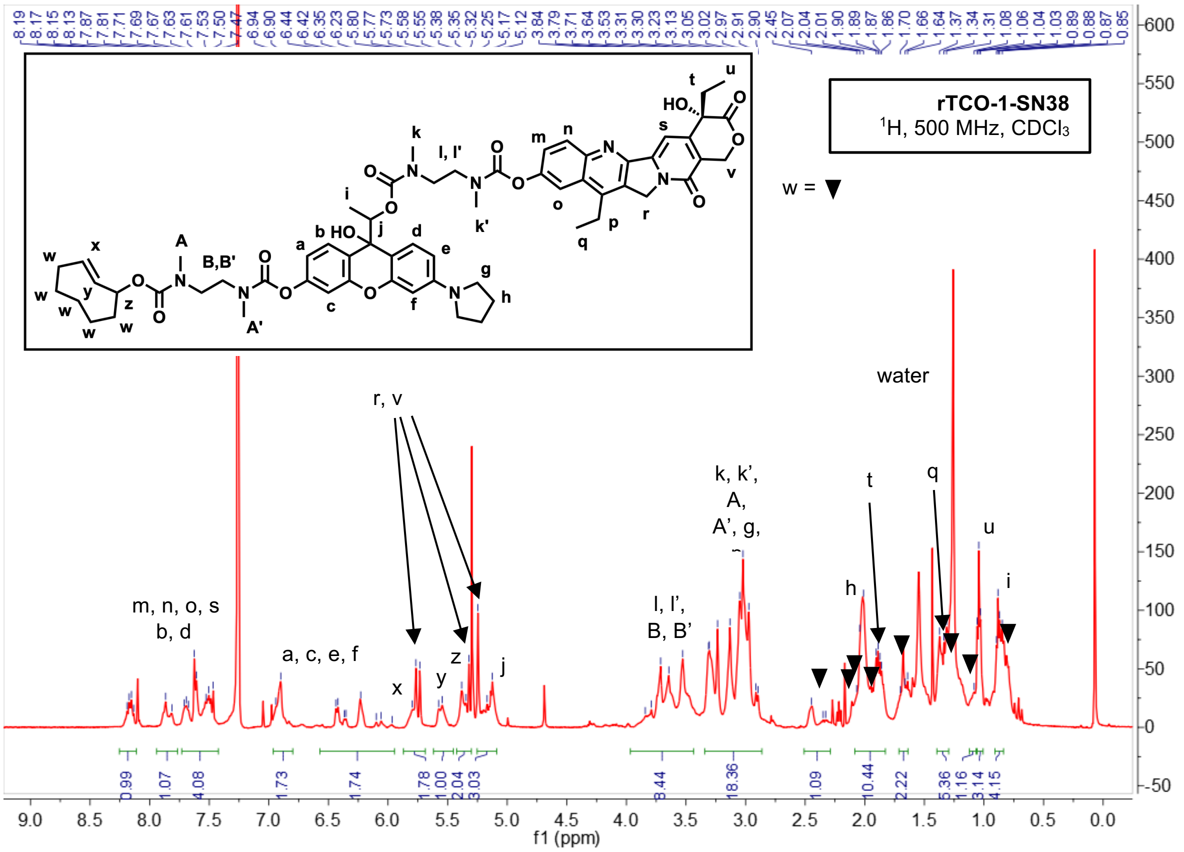
rTCO-1-SN38
1H, 500 MHz, CDCl3
w = ▼
water
m, n, o, s
b, d
a, c, e, f
r, v
x
y
z
j
l, l',
B, B'
k, k',
A,
A', g,
h
t
q
u
i
f1 (ppm)

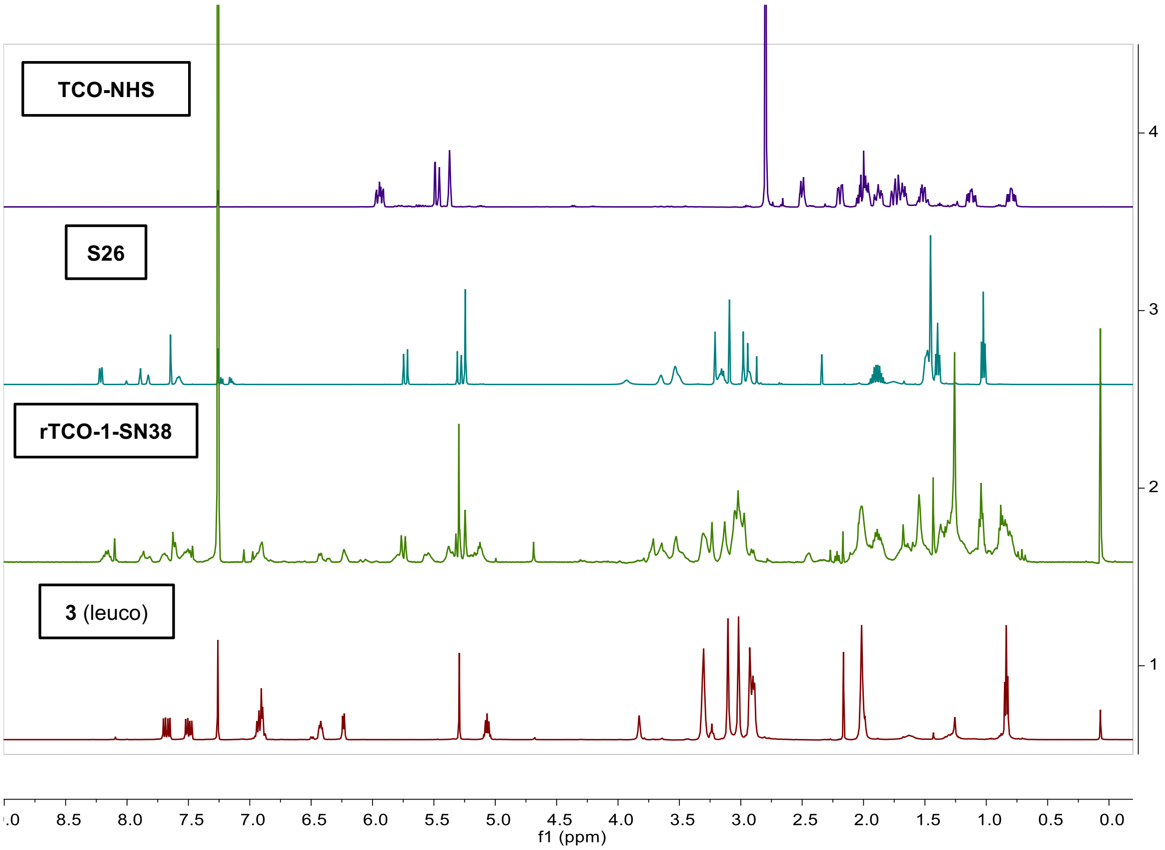
TCO-NHS
S26
rTCO-1-SN38
3 (leuco)
f1 (ppm)

rTCO-1-SN38

[M-H$_2$O+2H]$^{2+}$: 555

[M-TCO-H$_2$O+H]$^{+}$: 1000

Chromatogram

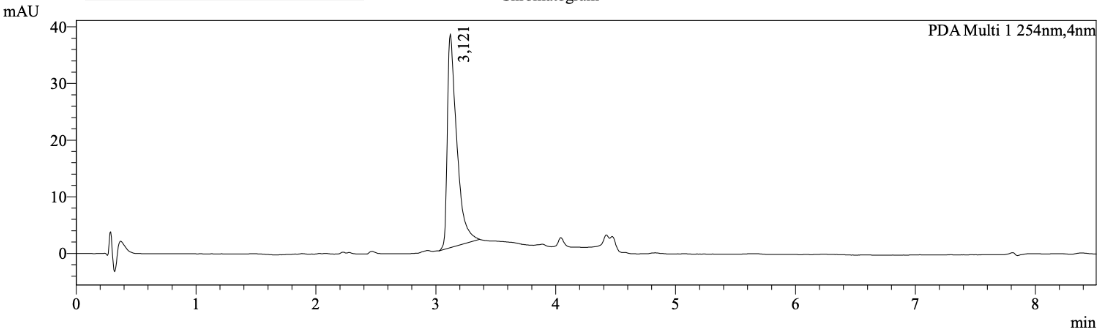

Peak Table

PDA Ch1 254nm

Peak#	Ret. Time	Peak Start	Peak End	Area	Height	Area/Height
1	3,121	3,029	3,365	206792	37754	5,477
Total				206792	37754	

Contour

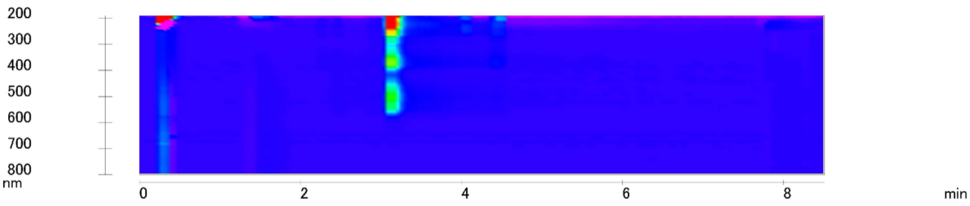

MS Chromatogram

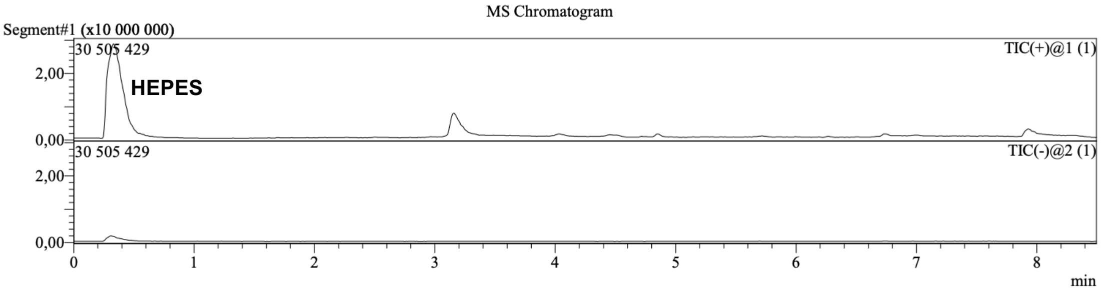

MS Spectrum

Line#:1 R.Time:----(Scan#:----)
MassPeaks:740
Spectrum Mode:Averaged 3,080-3,333(925-1001) Base Peak:555(927267)
BG Mode:Averaged 1,433-2,233(431-671) Segment 1 - Event 1

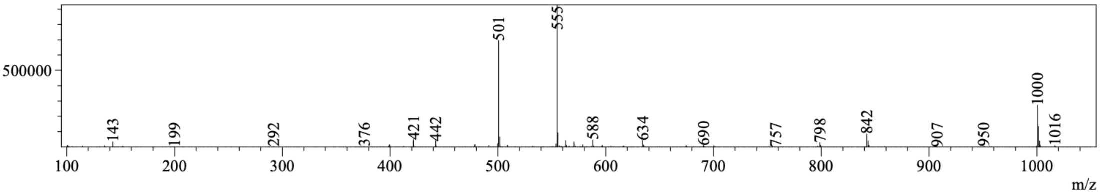

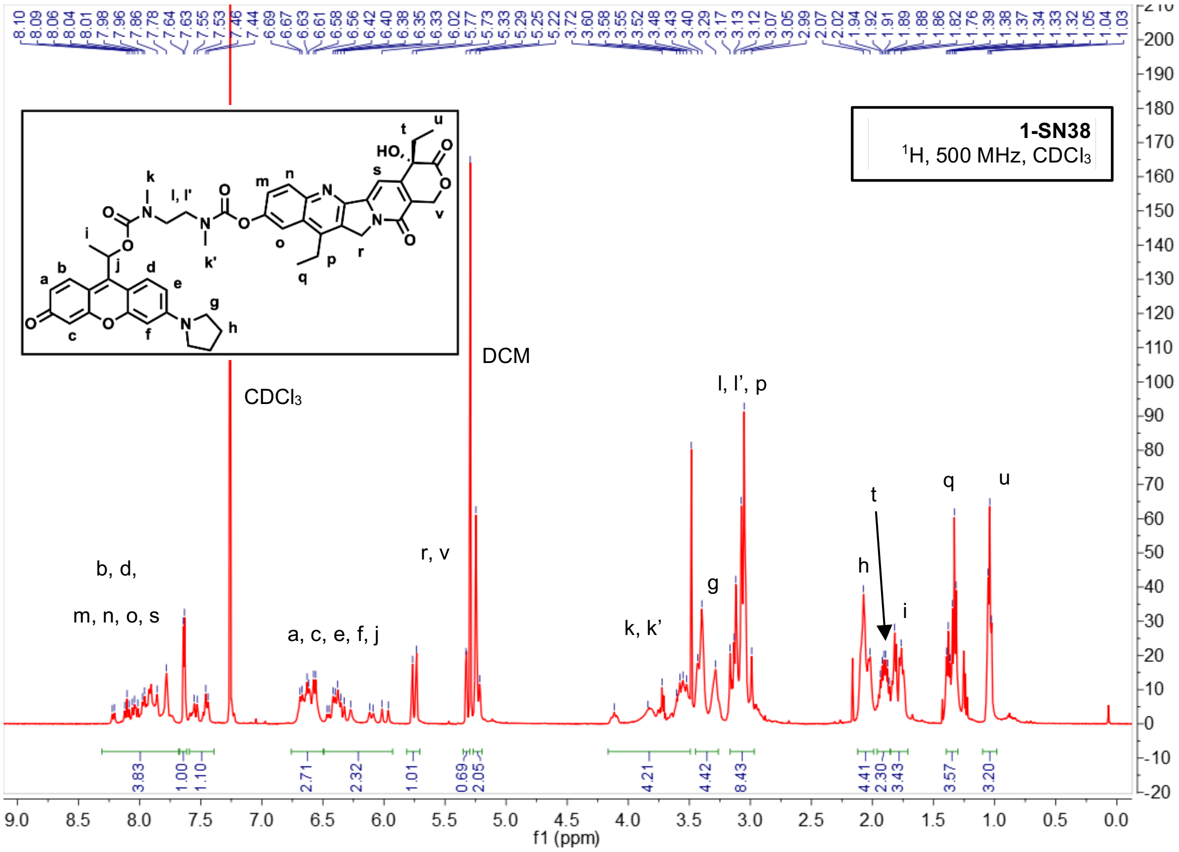
1-SN38
¹H, 500 MHz, CDCl3
CDCl3
DCM
b, d,
m, n, o, s
a, c, e, f, j
r, v
k, k'
g
l, l', p
h
t
i
q
u
3.83
1.00
1.10
2.71
2.32
1.01
0.69
2.05
4.21
4.42
8.43
4.41
2.30
3.43
3.57
3.20
f1 (ppm)

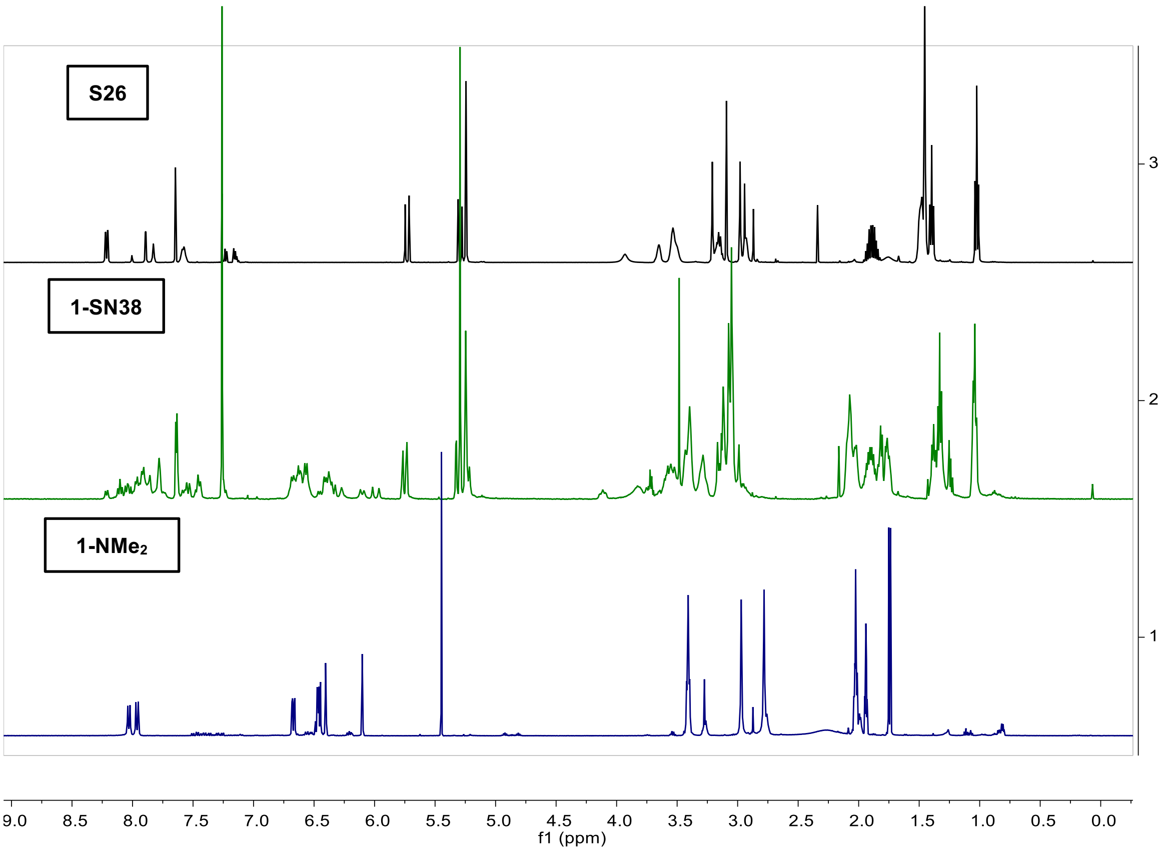
S26
1-SN38
1-NMe2
f1 (ppm)

1-SN38
$[M+H]^+$: 842

Chromatogram

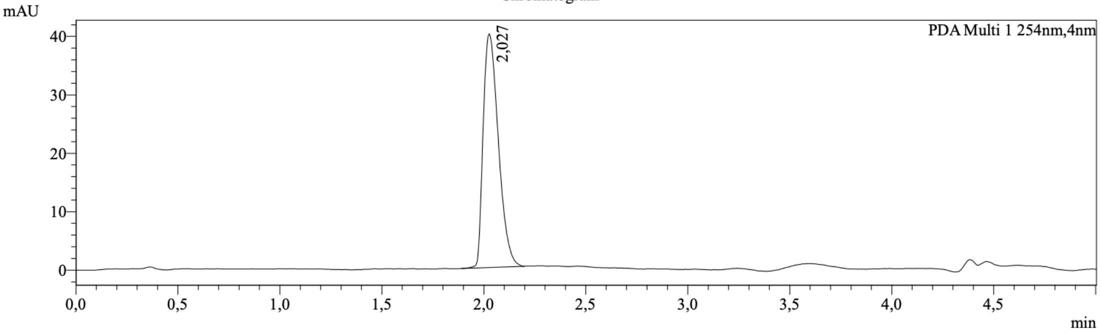

Peak Table

PDA Ch1 254nm

Peak#	Ret. Time	Peak Start	Peak End	Area	Height	Area/Height
1	2,027	1,893	2,197	207611	40041	5,185
Total				207611	40041	

Contour

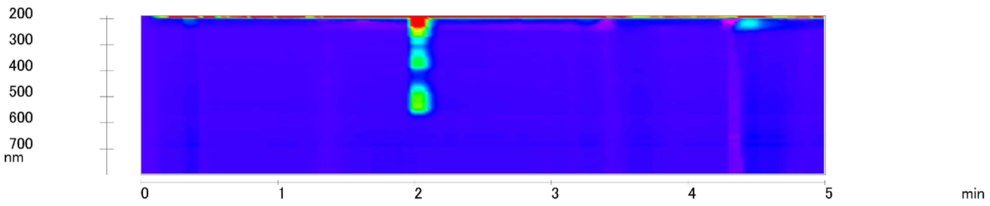

MS Chromatogram

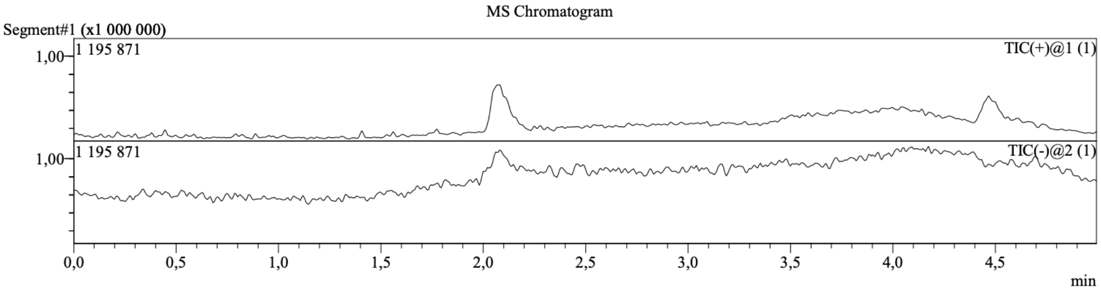

MS Spectrum

Line#:1 R.Time:----(Scan#:----)
MassPeaks:654
Spectrum Mode:Averaged 1,987-2,213(597-665) Base Peak:842(73197)
BG Mode:Averaged 1,027-1,273(309-383) Segment 1 - Event 1

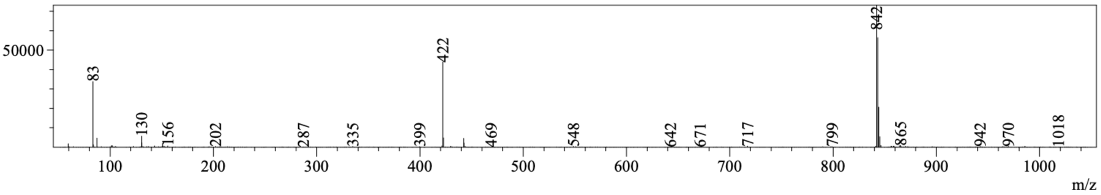

8. References

[1] Egyed, A.; Németh, K.; Molnár, T. Á.; Kállay, M.; Kele, P.; Bojtár, M. Turning Red without Feeling Embarrassed ─ Xanthenium-Based Photocages for Red-Light-Activated Phototherapeutics. *J. Am. Chem. Soc.* **2023**, *145* (7), 4026–4034. https://doi.org/10.1021/jacs.2c11499

[2] Pal, S.; Mukherjee, M.; Sen, B.; Mandal, S. K.; Lohar, S.; Chattopadhyay, P.; Dhara, K. A new fluorogenic probe for the selective detection of carbon monoxide in aqueous medium based on Pd(0) mediated reaction. *Chem. Commun.* **2015**, *51*, 4410–4413. https://doi.org/10.1039/C5CC00902B

[3] Bojtár, M.; Németh, K.; Domahidy, F.; Knorr, G.; Verkman, A.; Kállay, M.; Kele, P. Conditionally Activatable Visible-Light Photocages. *J. Am. Chem. Soc.* **2020**, *142* (35), 15164–15171. https://doi.org/10.1021/jacs.0c07508

[4] US2016/106859, 2016, A1, Location in patent: Paragraph 0284; 0285

[5] Han, Z.; Dong, L.; Sun, F.; Long, L.; Jiang, S.; Dai, X.; Zhang, M. A novel fluorescent probe with extremely low background fluorescence for sensing hypochlorite in zebrafish. *Anal. Biochem.* **2020**, *602*, 113795. https://doi.org/10.1016/j.ab.2020.113795

[6] van Onzen, A. H. A. M.; Versteegen, R. M.; Hoeben, F. J. M.; Filot, I. A. W.; Rossin, R.; Zhu, T.; Wu, J.; Hudson, P. J.; Janssen, H. M.; ten Hoeve, W.; Robillard, M. S. Bioorthogonal Tetrazine Carbamate Cleavage by Highly Reactive trans-Cyclooctene. *J. Am. Chem. Soc.* **2020**, *142* (25), 10955–10963. https://doi.org/10.1021/jacs.0c00531

[7] Kern, D., Lövei, A., Kele, P., Kormos, A. I Bind It That Way – Bioorthogonal Unmasking of Pro-Fluorescent Quinone Methides. *Eur. J. Org. Chem.* **2024**, *27* (34), e202400541. https://doi.org/10.1002/ejoc.202400541

[8] Murrey, H. E.; Judkins, J. C.; am Ende, C. W.; Ballard, T. E.; Fang, Y.; Riccardi, K.; Di, L.; Guilmette, E. R.; Schwartz, J. W.; Fox, J. M.; Johnson, D. S. Systematic Evaluation of Bioorthogonal Reactions in Live Cells with Clickable HaloTag Ligands: Implications for Intracellular Imaging *J. Am. Chem. Soc.* **2015**, *137* (35), 11461–11475. https://doi.org/10.1021/jacs.5b06847
